# Ring Expansion of 1-Indanones to 2-Halo-1-naphthols as an Entry Point to Gilvocarcin Natural Products

**DOI:** 10.1021/acs.orglett.1c03530

**Published:** 2021-11-15

**Authors:** Ivica Zamarija, Benjamin J. Marsh, Thomas Magauer

**Affiliations:** Institute of Organic Chemistry and Center for Molecular Biosciences, Leopold-Franzens-University Innsbruck, 6020 Innsbruck, Austria; Department of Chemistry and Pharmacy, Ludwig-Maximilians-University Munich, 81377 Munich, Germany

## Abstract

Herein, we describe a two-step ring expansion of 1-indanones to afford 2-chloro/bromo-1-naphthols (32 examples). The developed method shows broad functional group tolerance, benefits from mild reaction conditions, and enables rapid access to the tetracyclic core of gilvocarcin natural products. The orthogonally functionalized products allow for selective postmodifications as exemplified in the total synthesis of defucogilvocarcin M. For the selective oxidation of the chromene, a mild and regioselective oxidation protocol (DDQ and TBHP) was developed.

**Figure:**
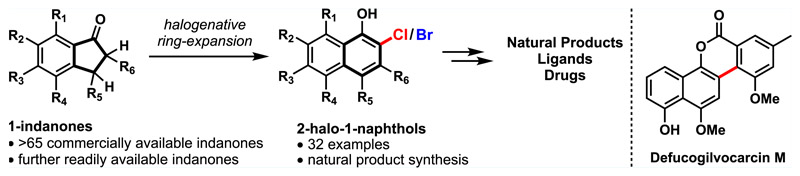

Polyfunctionalized aromatic structures that are derived from 1-naphthols are present in bioactive natural products, numerous pharmaceuticals, and chiral ligands.^1^ According to the substituents present at the *ortho*, *meta*, and *para* positions, two major classes can be identified (Scheme 1a). Class A comprises 3-carboxy-1-naphthols with variations at the *ortho* and *para* position as exemplified by chartartin^2^ (**1**), salimabromide^3^ (**2**), and diphyllin^4^ (**3**). On the contrary, parviflorene E^5^ (**5**), the VANOL ligand^6^ (**4**), and the gilvocarcin natural product ravidomycin^7^ (**6**) represent *ortho*-substituted 1-naphthols with different degrees of substitution at the *meta* and *para* position (class B). The potent biological activities associated with these structures as well as their use in asymmetric catalysis have attracted a great deal of attention for the development of efficient methods for their synthesis.^8^ Much effort has been spent to access orthogonally functionalized 1-naphthols. Despite significant progress in this area, the developed methods often involve multistep sequences,^9^ harsh reaction conditions,^10^ and the use of precious transition-metal catalysts^11^ or require a *β*-ketoester functionality to proceed.^12^ Other protocols are based on sensitive and uncommon intermediates or reagents (e.g., cyclobutenones, allenes, or nitrones).^13^ Moreover, they suffer from noncommercial starting materials, thus preventing rapid access to structurally diverse analogues. Here, we present a robust two-step protocol for the construction of orthogonally functionalized 2-halo-1-naphthols starting from 1-indanones. A plethora of 1-indanones with a broad substitution pattern are commercially available, and functionalized variations thereof are readily accessible via known literature procedures.^14^ The applicability of the developed methodology is shown for the synthesis of the natural product defucogilvocarcin M (**45**).^15^

During the course of our investigations to develop novel ring expansion reactions, we gained access to a variety of class A 1-naphthols (Scheme 1b).^16^ This protocol enabled the synthesis of chartarin (**1**) and also provided access to an advanced intermediate toward salimabromide (**2**).^17^ While a diverse set of (hetero)arenes were generated via this strategy, the inherent ester functionality restricted synthetic access to class A structures and variation of the *ortho* position was possible only at the stage of the 1-indanone (**7a**). In addition, several of the 1-indenone intermediates (**7b**) required for the cyclopropanation were unstable and prone to polymerization. We wanted to address these issues by investigating the ring expansion of gem-dihalocyclopropane^18^ **10**, readily available from indene **7c**. During our early investigations, Wang found access to 2-fluoro-1-naphthols via a related cyclo-propanation–ring expansion (CPRE) using (bromodifluoromethyl)-trimethylsilane.^10d^ The incorporation of a chlorine or bromine atom at this position was not possible via this strategy and restricted further diversification. Inspired by seminal work by Ciamician and Dennstedt and related reports on ring expansion reactions,^18,19^ we envisioned an alternative strategy that employs chloroform and bromoform as inexpensive and easy to handle halogen sources for installing the chloride and bromide, respectively. The obtained *ortho*-chlorinated and brominated 1-naphthols **11b** are more valuable substrates than their fluorinated analogues **11a**, especially when considering further postmodifications to access a large number of known bioactive 1-naphthols featuring an *ortho* substituent.^20^

We began our investigations by studying the CPRE of 1-indanone-derived trimethylsilyl enol ether **12** (Table 1). While aqueous sodium hydroxide (entry 1) or sodium methanolate (entry 2) led to only desilylation of the starting material, we were delighted to find that upon treatment of **12** with potassium tert-butoxide and chloroform in pentane at cryogenic temperatures ring expansion followed by partial *in situ* deprotection to 1-naphthol **14** was observed (entry 3). To ensure complete desilylation, hydrochloric acid (entry 4) or tetrabutylammonium fluoride (TBAF, entry 5) was added after full conversion of the starting material. While the use of acidic conditions provides slightly higher yields for **12**, TBAF proved to be superior with regard to functional group tolerance. It is noteworthy that the use of sublimed grade potassium *tert*-butoxide showed significantly higher yields compared to those of reagent grade batches. In this context, we were likewise interested in gaining access to 2-bromo-1-naphthols to expand the range of possible postmodifications of the obtained 1-naphthols (Table 2). A simple exchange of chloroform for bromoform gave the desired naphthol **17** in moderate yield (47%) accompanied by large amounts of recovered 1-indanone (entry 1). The competing desilylation was prevented by employing a more stable *tert*-butyldimethylsilyl enol ether. This allowed for the preparation of 2-bromo-1-naphthol **16** even at ambient temperature (entry 2). However, larger amounts of the base and bromoform were needed to ensure full conversion (entry 3). Although the combination of this protocol with deprotection conditions (DBU in MeCN/H_2_O or HF·pyr in THF) in a one-pot fashion afforded unprotected naphthol **17** in good yields, we observed reproducibility issues leading to varying yields between 56% and 76%. We also noticed that application of these conditions to a broader substrate scope led to significantly lower yields, not only at the stage of the ring expansion but also for the subsequent deprotection step. The inconsistencies of the subsequent TBS deprotection required another change of the protecting group. We later found that the use of a triisopropyl (TIPS) group was ideally suited as it provided good yields for the enol ethers and could be easily removed upon treatment with either TBAF or a suspension of KOAc in DMF/water (entry 4).^21^ Detailed studies showed that the reproducibility of the CPRE step was strongly dependent on the order and temperature at which the substrate and the base were combined. While addition at 23 or 0 °C immediately afforded a deep purple solution, addition at −78 °C led to the formation of a pale-yellow mixture and provided **17** in reproducible 85% yield (entry 5). Efforts to identify and characterize possible side products resulting from a competing aryne formation were unsuccessful.

With the optimized conditions in hand, we began investigating the conversion of several substrates to the corresponding 2-chloronaphthols (Scheme 2, protocol A). We found that halogens (**19Cl**–**24Cl**), acetals (**25Cl**), ethers (**26Cl**–**28Cl**), esters (**30Cl** and **31Cl**), alkyls (**33Cl**), and aryls (**32Cl**) and silyl ethers (**29Cl**) were stable under the reaction conditions to afford the corresponding 1-naphthols in yields of ≤83%. Unexpectedly, only the presence of methoxy groups led to significantly lower yields under the standard conditions (16% for **27Cl**, 57% for **28Cl**).^22^ This was attributed to the decreased stability of the transient silyl enol ether. We were able to address this issue by adapting the conditions developed for the preparation of 2-bromonaphthols (compare Table 2). Under these conditions, **27Cl** and **28Cl** were obtained in 83% and 81% yields, respectively.

When the substrates mentioned above were subjected to protocol B, comparable yields were obtained for halogenated naphthols **19Br**–**24Br**, benzyl ether **26Br**, silyl ether **28Br**, and *p*-phenyl derivative **32Br** (Scheme 2). However, the protocol was less compatible with electron-donating groups such as an acetal (**25Br**) or methoxy unit (**27Br** and **28Br**) and failed in the presence of an ester (**30Br** and **31Br**). The slightly decreased yield for *ortho,meta*-substituted naphthol **33Br** can be rationalized by steric hindrance. In the course of investigating further postmodifications to showcase the applicability of the obtained 2-halo-1-naphthols shown in Scheme 2, we observed an unusual dearomatization reaction. When 2-bromo-5-iodonaphthol **22** was treated with *N*-chlorosuccinimide (NCS) in acetonitrile, quantitative conversion to bench-stable enone **47** was observed without formation of expected naphthol **46**.^23^ We found that this rather rare dienone tautomer^24^ undergoes conjugate addition^25^ with several nucleophiles, thus representing a formal *meta*-functionalization (see the Supporting Information).

Having prepared a library of 2-halonaphthols, we turned our attention to the synthesis of defucogilvocarcin M [**45** (Scheme 3)].^15^ This natural product belongs to a family of >15 antitumor antibiotics, of which the first member was isolated in 1955.^26^ Due to their structural and biological properties, defucogilvocarcin M and its related members have become a popular synthetic target.^15b,26^ Starting from known indanone **34**,^27^ 2-bromonaphthol **35** was obtained in 70% yield over two steps on a gram scale. Two-step oxidation gave dihydroquinone **37**, which was regioselectively benzylated with **38** ^28^ in the presence of potassium carbonate to give ethers **39**. Subsequent methylation provided the key benzyl ethers **40**. Among the different known strategies for forming the gilvocarcins’ biaryl bond (e.g., Meerwein, Suzuki, Stille, Heck, and Meyers coupling), no Ar−X−Ar−X (X = halogen) coupling has been reported so far.^15b,26^ Somewhat surprisingly, all attempts to realize a Ni- or Pd-catalyzed intramolecular sp^2^−sp^2^ cross-coupling or a classical Ullmann coupling^29^ failed in our hands. After a survey of alternative methods, Lipshutz’s Cu(I)-mediated biaryl coupling protocol (t-BuLi, CuCN· 2LiX) evolved as the first solution for obtaining the full skeleton of **45** (procedure a).^30^ When an excess of *t*-BuLi (11 equiv) was used, simultaneous removal of the benzyl group took place to form **41b**, sparing an additional deprotection step (procedure b).^31^ Prolonged treatment with 1,3-dinitrobenzene (>1.5 h) led to overoxidation and thus opening of the lactone ring (not shown). Due to unsatisfactory yields, we screened further coupling conditions and were delighted to see that Stille−Kelly coupling^32^ afforded the desired tetracycle **41a** in 63% yield, with the results for **40Br** being better than those of **40I** (see the Supporting Information for a detailed screening table). Severe and unanticipated difficulties awaited us when we attempted the oxidation of chromene **41** to install the missing lactone unit. For this purpose, we initially protected the free hydroxy-chromene **41b**. Compound **41c** resisted oxidation to the corresponding chromenone **43** by several established procedures, including PCC, PDC, SeO_2_, KMnO_4_, MnO_2_, TBHP/KI, or TBHP/I_2_.^33^ In most cases, ring opening of the intermediate lactol to give the corresponding benzoquine or decomposition was observed. Progress was made when we found that treating a solution of **41c** in 1,4-dioxane with DDQ and TBHP resulted in the formation of peroxyacetal **42c** (R = *i*-Pr). When the partially purified peroxyacetal **42c** was treated with 1,8-diazabicyclo[5.4.0]-undec-7-ene (DBU) in dichloromethane, a Kornblum–DeLaMare rearrangement^34^ to the desired lactone took place. We were pleased to see that this transformation could also be applied in a one-pot fashion affording isopropylated defucogilvocarcin M (**43**) in 85% yield. Moreover, these conditions not only were completely selective for the chromene core in the presence of a benzyl group (**44**) but also tolerated the free hydroxyl group of naphthol **41b** to directly give defucogilvocarcin M (**45**) in 80% yield. Spectroscopic data (^1^H NMR and ^13^C NMR) for the synthetic material were in full agreement with reported values.^35^

In summary, we have developed a powerful protocol for converting a broad range of readily available 1-indanones into diversely substituted 2-chloro/2-bromo-1-naphthols. The halogen in the *ortho* position served as a useful handle for further functionalization as demonstrated in the synthesis of defucogilvocarcin M. In addition, a mild protocol for the selective benzylic oxidation of chromenes was developed.

## Supplementary Material

The Supporting Information is available free of charge at https://pubs.acs.org/doi/10.1021/acs.orglett.1c03530.

Experimental details and characterization data (PDF)

Supplementary Information

## Figures and Tables

**Scheme 1:**
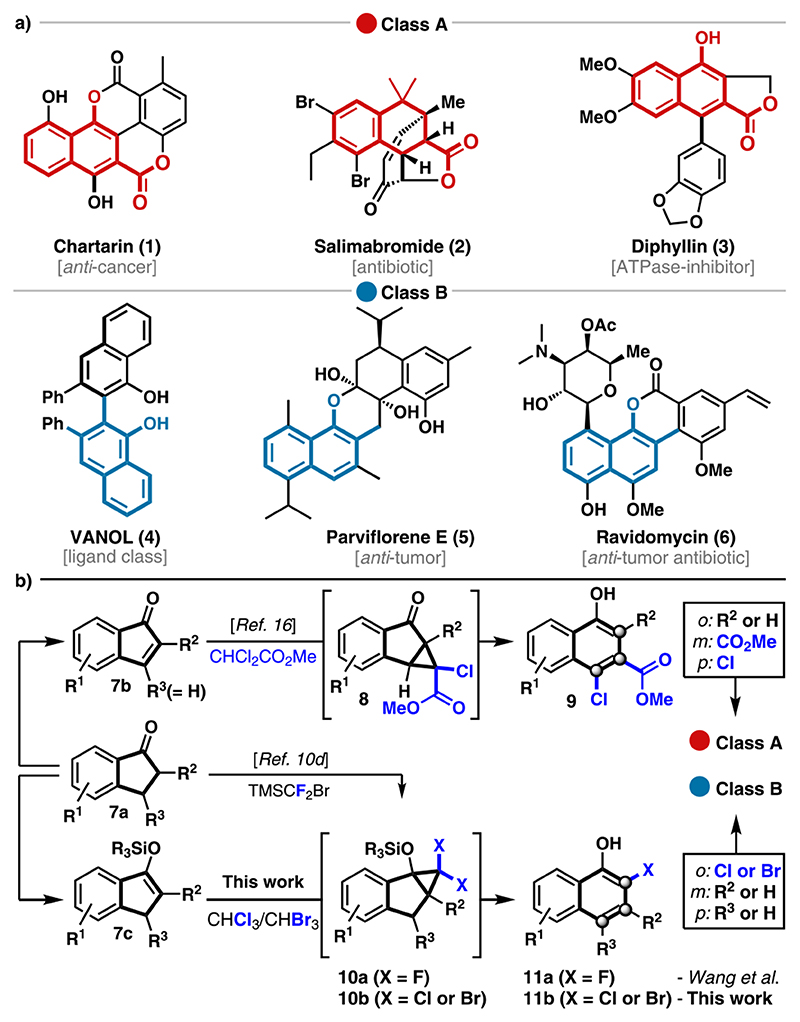
Selected Examples and Synthetic Access to Chemically and Biologically Relevant 1-Naphthols

**Scheme 2:**
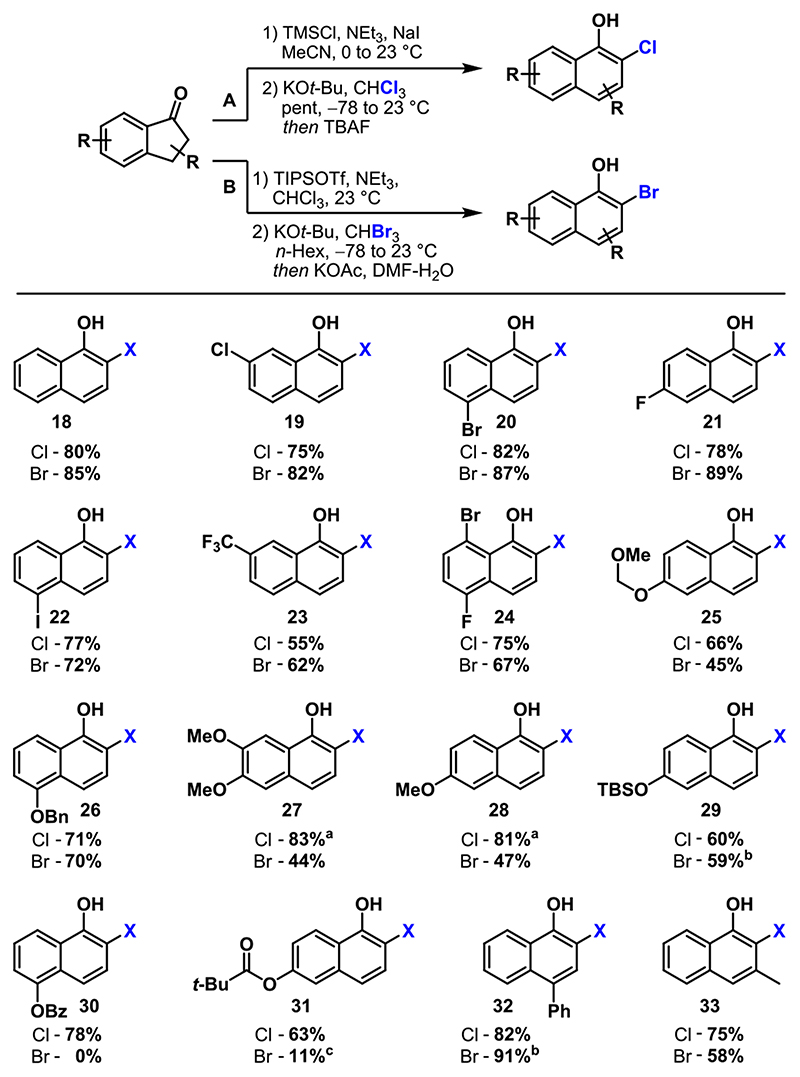
Scope of 2-Chloro- and 2-Bromo-1-naphthols Obtained via CPRE of 1-Indanones ^*a*^Via TIPS-silylenol ether. ^*b*^Yield for TIPS-protected naphthol (see the Supporting Information for details). ^*c*^TBAF deprotection.

**Scheme 3:**
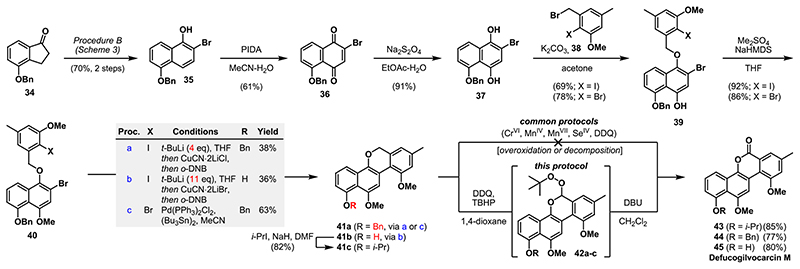
Application of the CPRE Protocol to the Synthesis of Defucogilvocarcin M

**Table 1:** Selected Screening Conditions for the Preparation of 2-Chloro-1-naphthols

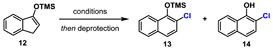							
entry	reagents	temp	time	solvent	deprotection	yield of 13 (%)	yield of 14 (%)
1	CHCI_3_, NaOH, BnEt_3_NCI	45 °C	3 days	CH_2_CI_2_, H_2_O	–	0	0
2	CCI_3_COOEt, NaOMe	0 °C	4 h	pentane	–	0	0
3	CHCI_3_, KO*t*-Bu	−78 to 23 °C	3 h	pentane (0.5 M)	–	10	55
4	CHCI_3_, KO*t*-Bu	−78 to 23 °C	3 h	pentane (0.5 M)	aqueous HCI	0	86
5	CHCI_3_, KO*t*-Bu	−78 to 23 °C	2 h	pentane (0.2 M)	TBAF	0	80

**Table 2:** Selected Screening Conditions for the Preparation of 2-Bromo-1-naphthols

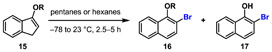								
entry	R	KO*t*-Bu (equiv)	CHBr_3_ (equiv)	base addition	deprotection	yield of 15 (%)	yield of 16 (%)	yield of 17 (%)
1	TMS	2.0	2.2	at −78 °C	TBAF, THF	0	0	47
2	TBS	2.0	2.2	at 23 °C	-	43	36	0
3	TBS	6.0	5.0	at 23 °C	DBU, MeCN/H_2_O	0	0	67
4	TIPS	4.5	2.0	at 23 °C	KOAc, DMF/H_2_O	0	0	70
5	TIPS	4.5	2.0	at −78 °C	KOAc, DMF/H_2_O	0	0	85
