# Supplementary material for: Ring Expansion of 1-Indanones to 2-Halo-1-naphthols as an Entry Point to Gilvocarcin Natural Products
Source: Org Lett. 2021 Nov 15;23(23):9221–6. doi: 10.1021/acs.orglett.1c03530 (PMC7612072; doi:10.1021/acs.orglett.1c03530)
Supplement: Supplementary file 1 — ol1c03530_si_001.pdf [file ol1c03530_si_001.pdf]

# – Supporting Information –

Ring-Expansion of 1-Indanones to 2-Halo-1-naphthols as an Entry Point to Gilvocarcin Natural Products

Ivica Zamarija, Benjamin J. Marsh†, Thomas Magauer*

[a] Institute of Organic Chemistry and Center for Molecular Biosciences, Leopold-Franzens-University Innsbruck, Innrain 80−82, 6020 Innsbruck, Austria.

† Faculty for Chemistry and Pharmacy, Ludwig-Maximilians-University Munich, Butenandtstraße 5–13, 81377 Munich, Germany.

*corresponding author: E-Mail: thomas.magauer@uibk.ac.at

Table of Contents

1 General Experimental Details S2

2 Cyclopropanation–Ring-Expansion (CPRE) of 1-Indanones S4

2.1 Optimization of the CPRE towards 2-Chloro-1-naphthols S4

2.2 Optimization of the CPRE towards 2-Bromo-1-naphthols S5

2.3 General Procedures S6

2.4 Substrate Scope (2-Chloro-1-naphthols) S8

2.5 Substrate Scope (2-Bromo-1-naphthols) S14

3 Synthesis of Defucogilvocarcin M S21

3.1 Synthesis of Linear Precursors **40-Br** and **40-I** S21

3.2 Intramolecular Biaryl Coupling S28

3.3 Mild Benzylic Oxidation of a Quinone-based Chromene using DDQ and TBHP S32

4 Meta-functionalization of 2-Bromo-5-iodo-1-naphthol S36

5 NMR-Spectra S42

6 References S94

1. General Experimental Details

All reactions were performed in flame-dried glassware fitted with rubber septa under a positive pressure of argon, unless otherwise noted. Air- and moisture-sensitive liquids were transferred via syringe or stainless-steel cannula through rubber septa. Solids were added under inert gas counter flow or were dissolved in appropriate solvents. Reactions at low temperatures were carried out in a Dewar vessel filled with a cooling agent: acetone/dry ice (−78 °C), acetonitrile/dry ice (−40 °C) or distilled water/ice (0 °C). Reaction temperatures above 23 °C conducted in a heated oil bath or a metal block. The reactions were magnetically stirred and monitored by NMR spectroscopy or analytical thin-layer chromatography (TLC), using aluminum plates precoated with silica gel (0.25 mm, 60 Å pore size, Merck or Macherey-Nagel impregnated with a fluorescent indicator (254 nm). TLC plates were visualized by exposure to ultraviolet light (UV), were stained by submersion in aqueous potassium permanganate solution ($KMnO_4$) or ceric ammonium molybdate solution (CAM) and were developed by heating with a heat gun.

Flash-Column Chromatography (FCC) was performed as described by Still et al.[1] employing silica gel (60 Å, 40–63 µm, Merck) or magnesium silicate Florisil® (60–100 mesh, Carl Roth). The yields refer to chromatographically and spectroscopically (^{1}H and ^{13}C NMR) pure material.

Solvents and Reagents as pentane (pent), acetonitrile (MeCN), chloroform ($CHCl_3$), dichloromethane (CH_2Cl_2), diethyl ether (Et_2O), cyclohexane (*c*-hex), *n*-hexane (*n*-hex), ethyl acetate (EtOAc), 1,4-dioxane, *N*,*N*-dimethylformamide (DMF), tetrahydrofuran (THF) and toluene (PhMe) were purchased from Acros Organics or Sigma Aldrich as 'extra dry' reagents and used as received. Triethylamine (Et_3N, Sigma Aldrich) and *N*-chlorosuccinimide (NCS, TCI) were used without further purification. Other reagents or solvents were purchased from chemical suppliers (Sigma-Aldrich, TCI, Fisher Scientific or others) and were used as received. Solvents for extraction, crystallization and flash column chromatography as petroleum ether (40–60 °C) were purchased in technical grade and distilled under reduced pressure prior to use. The molarity of *n*-butyllithium (*n*-BuLi) and *tert*-butyllithium (*t*-BuLi) solutions was determined by titration against diphenyl acetic acid as indicator (average of three determinations).[2]

NMR Spectra were measured on a Bruker Avance Neo 400 MHz spectrometer and a Bruker Avance II 600 MHz spectrometer at Leopold–Franzens–University Innsbruck (LFU Innsbruck). Proton chemical shifts are expressed in parts per million (ppm, δ scale) and are referenced to residual proton in the NMR solvent (CHD_2CN: δ = 1.94; CHD_2OD: δ = 3.31; $CHDCl_2$: δ = 5.32; $CHCl_3$: δ = 7.26). Carbon chemical shifts are expressed in parts per million (δ scale, assigned carbon atom) and are referenced to the carbon resonance of the NMR solvent (CD_3CN: δ = 118.26; CD_3OD: δ = 49.00; CD_2Cl_2: δ = 53.84; $CDCl_3$: δ = 77.16). Fluorine chemical shifts are expressed in parts per million (δ scale) and are referenced to $CFCl_3$ (*Bruker NMR set-up*) internal standard. ^{1}H- and ^{19}F-NMR spectroscopic data are reported as follows: Chemical shift in ppm (multiplicity, coupling constants *J* (Hz), integration intensity, assigned proton).

The multiplicities are abbreviated with s (singlet), d (doublet), t (triplet), q (quartet) and m (multiplet). In case of combined multiplicities, the multiplicity with the larger coupling constant is stated first. Except for multiplets, the chemical shift of all signals, as well for centrosymmetric multiplets, is reported as the center of the resonance range. In addition to ^{1}H- and ^{13}C-NMR measurements, 2D-NMR techniques such as homonuclear correlation spectroscopy (COSY), total correlation spectroscopy (TOCSY), heteronuclear single quantum coherence (HSQC) and heteronuclear multiple bond coherence (HMBC) were used to assist signal assignment. For further elucidation of 3D structures of the products, nuclear Overhauser enhancement spectroscopy (NOESY) was conducted. Coupling constants *J* are reported in Hz. All raw fid files were processed and the spectra analyzed using the software MestReNova 12.0.2 from Mestrelab Research.

Infrared Spectra (IR) were recorded on a Perkin Elmer Spectrum BX II FT-IR system. If required, substances were dissolved in CH_2Cl_2 or $CDCl_3$ prior to direct application on the ATR unit. Data are represented as follows: frequency of absorption (cm^{-1}) and intensity of absorption (vs = very strong, s = strong, m = medium, w = weak, br = broad).

High-Resolution Mass Spectra (HRMS) as ESI-HRMS were recorded on a Thermo Scientific™ Q Exactive™ Orbitrap Mass Spectrometer at LFU Innsbruck. DESI-HRMS as well as LTP-HRMS were recorded on a Thermo Scientific™ LTQ Orbitrap XL™ Hybrid Ion Trap-Orbitrap Mass Spectrometer equipped with a 3-in-1 ambient ionization interface.

Melting Points (MP) were measured with an SRS-MPA120 EZ-Melt Melting Point Apparatus in open glass capillaries and are uncorrected.

2. Cyclopropanation–Ring-Expansion (CPRE) of 1-Indanones

2.1 Optimization of the CPRE towards 2-Chloro-1-naphthols

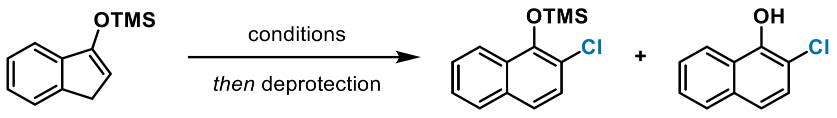

Entry	Reagents[a]	Temperature	Time	Deprotection[b]	Solvent	13	14
1	CCl_3CO_2Et, NaOMe	0 °C	4 h	-	pentane-H_2O (0.5 M)	0%	0%
2	$CHCl_3$, NaOH, $BnEt_3NCl$	45 °C	3 d	-	CH_2Cl_2-H_2O (0.5 M)	0%	0%
3	$CHCl_3$, KO*t*-Bu	0 to 23 °C	2 h	-	pentane (0.5 M)	18%	12%
4	$CHCl_3$, KO*t*-Bu	–78 to 23 °C	3 h	-	pentane (0.5 M)	10%	55%
5	$CHCl_3$, KO*t*-Bu	–78 to 23 °C	3 h	aq. HCl	pentane (0.5 M)	0%	86%
6	$CHCl_3$, KO*t*-Bu	–78 to 23 °C	2 h	aq. HCl	CH_2Cl_2 (0.5 M)	0%	40%
7	$CHCl_3$, KO*t*-Bu	–78 to 23 °C	2 h	aq. HCl	PhMe (0.5 M)	0%	40%
8	$CHCl_3$, KO*t*-Bu	–78 to 23 °C	2 h	aq. HCl	MeCN (0.5 M)	0%	5%
9	$CHCl_3$, KO*t*-Bu	–78 to 23 °C	2 h	aq. HCl	$CHCl_3$ (0.5 M)	0%	35%
10	$CHCl_3$, KO*t*-Bu	–78 to 23 °C	2 h	TBAF	pentane (0.2 M)	0%	59%
**11[c]**	**$CHCl_3$, KO*t*-Bu**	**–78 to 23 °C**	**2 h**	**TBAF**	**pentane (0.2 M)**	**0%**	**80%**

[a] 2.2 equivalents of Cl-source and 2.0 equivalents of base were used.
[b] "aq. HCl" = 4 M HCl, 40 min, 23 °C. "TBAF" = 1.1 eq TBAF (1 M in THF), 30 min, 23 °C.
[c] These conditions were preferred over the conditions from Entry 5 for substrate compatibility of a broader scope.

2.2 Optimization of the CPRE towards 2-Bromo-1-naphthols

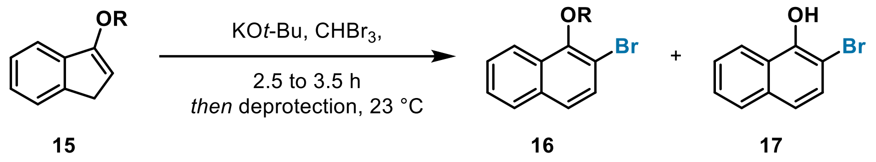

Entry	R	KO*t*-Bu	$CHBr_3$	Solvent	Temperature	Deprotection	15	16	17
1	TMS	2.0 eq	2.2 eq	pentane	–78 to 23 °C[a]	TBAF·xH_2O, THF, 0.5 h	0%	0%	33%
**2**	**TMS**	**2.0 eq**	**2.2 eq**	**pentane**	**–78 to 23 °C[b]**	**TBAF·xH_2O, THF, 0.5 h**	**0%**	**0%**	**47%**
3	TBS[c]	2.0 eq	2.2 eq	pentane	–78 to 23 °C[a]	–	43%	36%	0%
4	TBS[c]	6.0 eq	5.0 eq	pentane	–78 to 23 °C[a]	–	5%	76%	0%
5	TBS[c]	6.0 eq	5.0 eq	*n*-hexane	23 °C[a]	–	0%	79%	0%
**6**	**TBS[c]**	**6.0 eq**	**5.0 eq**	***c*-hexane**	**23 °C[a]**	**–**	**0%[d]**	**80%[d]**	**0%[d]**
7	TBS[c]	6.0 eq	5.0 eq	*c*-hexane	23 °C[a]	aq. HCl (2 M), 1 h	0%	74%	0%
8	TBS[c]	6.0 eq	5.0 eq	*c*-hexane	23 °C[a]	TBAF (1 M in THF), 0.5 h	0%[d]	0%[d]	64%[d]
9	TBS[c]	6.0 eq	5.0 eq	*c*-hexane	23 °C[a]	KF, MeCN-H_2O, 22 h	0%	12%	36%
10	TBS[c]	6.0 eq	5.0 eq	*c*-hexane	23 °C[a]	KF-Al_2O_3, MeCN-THF, 4.5 h	0%	1%	25%
**11**	**TBS[c]**	**6.0 eq**	**5.0 eq**	***c*-hexane**	**23 °C[a]**	**DBU, MeCN-H_2O, 1.5 h**	**0%[d]**	**0%[d]**	**67%[d]**
12	TIPS	6.0 eq	5.0 eq	pentane	23 °C[a]	–	0%	83%	0%
13	TIPS	6.0 eq	5.0 eq	pentane	23 °C[a]	TBAF (1 M in THF), 1 h	0%[d]	0%[d]	60%[d]
14	TIPS	4.5 eq	2.0 eq	pentane	23 °C[a]	–	0%[d]	72%[d]	0%[d]
15	TIPS	4.5 eq	2.0 eq	*n*-hexane	23 °C[a]	KOAc, DMF-H_2O, 1 h	0%[d]	0%[d]	77%[d]
16	TIPS	4.5 eq	2.0 eq	*n*-hexane	–78 to 23 °C[b]	–	0%[e]	88%[e]	0%[e]
**17**	**TIPS**	**4.5 eq**	**2.0 eq**	***n*-hexane**	**–78 to 23 °C[b]**	**KOAc, DMF-H_2O, 1 h**	**0%[e]**	**0%[e]**	**85%[e]**
18	TIPS	2.0 eq	2.2 eq	pentane	–78 to 23 °C[b]	TBAF (1 M in THF), 1 h	0%	0%	57%

[a] Base was added at 23 °C. $CHBr_3$ was added at the temperature given in the table with subsequent warming to 23 °C.
[b] Base was added at –78 °C. $CHBr_3$ was added at –78 °C with subsequent warming to 23 °C.
[c] We want to annotate, that the TBS-protection of 1-indanones is highly sensitive to the used amount of the solvent (benzene) with slightly higher dilutions already leading to significantly lower conversion, even after extended reaction times.
[d] Repeating this reaction revealed a limited reproducibility of the yield. The best yield is given in the table. For detailed information, see text.
[e] Repeating this reaction revealed a reliable reproducibility of the yield.

2.3 General Procedures

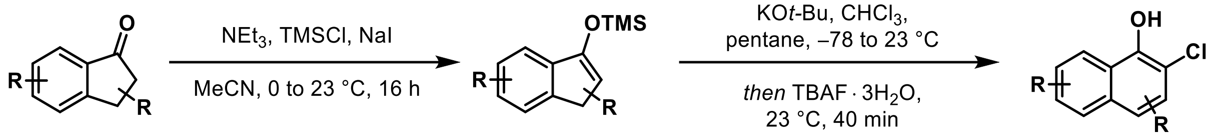

General Procedure Towards 2-Chloronaphthols (Procedure A)

Triethylamine (158 µL, 1.13 mmol, 1.50 equiv) and trimethylsilyl chloride (145 µL, 1.13 mmol, 1.50 equiv) were added in sequence to a suspension of sodium iodide (11.3 mg, 75.7 µmol, 10 mol%) and indanone (757 µmol, 1 equiv) in acetonitrile (910 µL, 0.83 M) at 0 ˚C. The resulting suspension was allowed to warm to 23 ˚C and stirred for 16 hours. After removal of the solvent under reduced pressure, the residue was dissolved in *n*-hexane (5 mL) and the suspension was filtered through a short plug of Celite®. The filtrate was then concentrated under reduced pressure to afford the crude silyl enol ether. This material was used immediately without further purification.

The crude silyl enol ether was dissolved in pentane (900 µL, 0.84 M), cooled to –78 °C and slowly added to a suspension of potassium *tert*-butoxide (sublimed grade, 170 mg, 1.51 mmol, 2.00 equiv) in pentane (1.51 mL, 1 M) at –78 ˚C. The flask of the crude silyl enol ether was rinsed for three times with pentane (3 × 800 µL) and added to the reaction in the same fashion. A solution of chloroform (133 µL, 1.67 mmol, 2.20 equiv) in pentane (1.67 mL, 1 M) was added dropwise to the mixture and the suspension was stirred at –78 ˚C for 30 minutes before allowing to warm to 23 ˚C. After stirring for 1.5 hours, water (2 mL) was added to the reaction mixture and the resulting solution was concentrated to 4 mL under reduced pressure. Tetrabutylammonium fluoride trihydrate (358 mg, 1.13 mmol, 1.50 equiv) was added in one portion and the solution was stirred vigorously for 40 minutes. Aqueous hydrochloric acid (1 M, 5 mL) was added and the resulting solution was extracted with ethyl acetate (3 × 10 mL). The organic layer was then washed with water (2 × 10 mL) and a saturated aqueous solution of sodium chloride (10 mL) and the washed solution was dried over magnesium sulfate. Filtration of the dried solution followed by concentration under reduced pressure gave the crude product, which was purified by silica gel chromatography to give the desired 2-chloronaphthols.

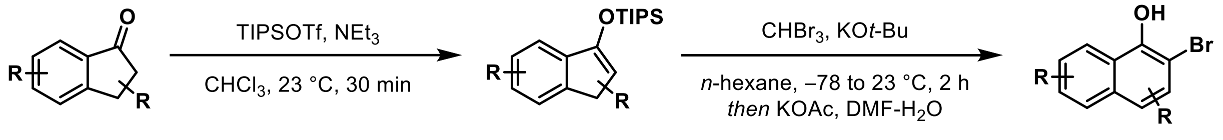

General Procedure Towards 2-Bromonaphthols (Procedure B)

Triethylamine (82.0 µL, 588 µmol, 1.40 equiv) and triisopropylsilyl trifluoromethanesulfonate (124 µL, 462 µmol, 1.10 equiv) were sequentially added to a solution of indanone (420 µmol, 1 equiv) in chloroform (5.25 mL, 0.08 M) at 23 ˚C. The reaction was stirred until full conversion (0.5 to 1.5 h). Reaction progress was visualized by basic Al_2O_3-TLC-monitoring. If remaining starting material was

indicated after two hours of stirring, additional triethylamine (199 µL, 1.43 mmol, 3.40 equiv) and triisopropylsilyl trifluoromethanesulfonate (158 µL, 588 µmol, 1.40 equiv) were added and stirred until full conversion (only needed for substrate **24-Br** in our hands). The mixture was diluted with cyclohexane (5 mL), filtered through a short plug of silica and the filtrate was concentrated under reduced pressure at 23 °C. The crude silyl enol ether was used immediately without further purification. The crude silyl enol ether was dissolved in *n*-hexane (600 µL, 0.7 M), cooled to –78 °C and added to a suspension of potassium *tert*-butoxide (sublimed grade, 212 mg, 1.89 mmol, 4.50 equiv) in *n*-hexane (859 µL, 2.2 M) at –78 ˚C. The flask of the crude silyl enol ether was rinsed with *n*-hexane (3 × 500 µL) and added to the reaction in the same fashion. After 20 minutes, a solution of freshly distilled bromoform (73.0 µL, 840 µmol, 2.00 equiv) in *n*-hexane (859 µL, 2.2 M) was added dropwise at –78 ˚C and stirred at that temperature for one hour. The reaction was then warmed to 23 °C within one hour and stirred for an additional hour. The solvent was removed under reduced pressure to give the crude silylated 2-bromonaphthol, which was dissolved in *N*,*N*-dimethylformamide-water (20:1, 1.68 mL, 0.25 M). Potassium acetate (41.0 mg, 420 µmol, 1.00 equiv) was added and the reaction was stirred for one hour at 23 ˚C. If silica-TLC-monitoring indicated remaining starting material after 1.5 hours, additional potassium acetate (41.0 mg, 420 µmol, 1.00 equiv) was added and the reaction mixture was stirred at 45 °C until full conversion (only needed for substrates **27-Br** and **28-Br** in our hands). Water (4 mL) was added to the reaction mixture and the resulting solution was extracted with diethyl ether (6 × 4 mL). The combined organic layers were washed with water (8 mL) and a saturated aqueous solution of sodium chloride (8 mL) and the washed solution was dried over magnesium sulfate. The dried solution was filtered and the filtrate was concentrated under reduced pressure. Purification by silica gel chromatography gave the desired 2-bromonaphthols.

General Procedure Towards 2-Bromonaphthols for Substrates 31-Br and 32-Br (Procedure C)

Procedure C follows the protocol of Procedure B only differing in the deprotection. Instead of dissolving the crude silylated 2-bromonaphthol in *N*,*N*-dimethylformamide-water, it was dissolved in tetrahydrofuran (10.5 mL, 0.04 M) and cooled to 0 °C, before tetrabutylammonium fluoride (1 M in tetrahydrofuran, 675 µL, 675 µmol, 1.50 equiv) was added dropwise to the mixture and stirred for one hour at that temperature. Aqueous hydrochloric acid (1 M, 4 mL) was added and the mixture was poured onto a mixture of water and ethyl acetate (1:1, 10 mL). The phases were separated and the aqueous layer was extracted with ethyl acetate (2 × 20 mL). The combined organic layers were washed with water (8 mL) and a saturated aqueous solution of sodium chloride (8 mL). The washed solution was dried over magnesium sulfate. The dried solution was filtered and the filtrate was concentrated under reduced pressure. Purification by silica gel chromatography gave the desired 2-bromonaphthols.

2.4 Substrate Scope (2-Chloro-1-naphthols)

2-Chloronaphthalen-1-ol (18-Cl)

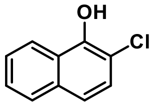

Isolated: 108 mg (80%)

Data consistent with literature: *Angew. Chem. Int. Ed.*, **2013**, *52*, 5271–5274.

2,7-Dichloronaphthalen-1-ol (19-Cl)

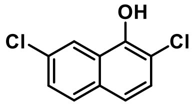

Isolated: Yellow oil, 121 mg (75%)

TLC (20% diethyl ether in pentane): R_f = 0.70 (UV, $KMnO_4$).

^{1}H NMR (400 MHz, chloroform-*d*) δ 8.20 (d, *J* = 2.1, 1H), 7.70 (d, *J* = 8.7 Hz, 1H), 7.43 (dd, *J* = 8.8, 2.1 Hz, 1H), 7.38 – 7.32 (m, 2H), 6.00 (s, 1H).

^{13}C NMR (101 MHz, chloroform-*d*) δ 146.5, 132.3, 131.5, 129.3, 127.7, 126.3, 125.1, 121.4, 120.8, 114.8.

IR (Diamond-ATR, neat) $\tilde{\upsilon}_{max}$: 3513 (m, br), 1586 (w), 1265 (s), 1090 (vs), 899 (m) cm^{-1}.

HRMS (ESI) calc. for $C_{10}H_5OCl_2$ [M-H]$^-$: 210.9723 found: 210.9724.

5-Bromo-2-chloronaphthalen-1-ol (20-Cl)

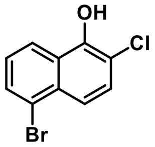

Isolated: Orange solid, 160 mg (82%)

TLC (20% diethyl ether in pentane): R_f = 0.75 (UV, $KMnO_4$).

mp: 86–90 °C.

^{1}H NMR (400 MHz, chloroform-*d*) δ 8.19 (d, *J* = 8.5 Hz, 1H), 7.76 (dd, *J* = 11.6, 8.3 Hz, 2H), 7.44 (d, *J* = 9.2 Hz, 1H), 7.32 (t, *J* = 7.9 Hz, 1H), 6.04 (s, 1H).

^{13}C NMR (101 MHz, chloroform-*d*) δ 147.1, 131.8, 130.9, 127.2, 126.5, 125.6, 122.6, 122.1, 120.3, 114.7.

IR (Diamond-ATR, neat) $\tilde{\upsilon}_{max}$: 3509 (w), 1619 (m), 1395 (w), 1237 (s), 1192 (vs), 870 (w) cm^{-1}.

HRMS (ESI) calc. for $C_{10}H_5BrClO$ [M-H]$^-$: 254.9218 found: 254.9220.

2-Chloro-6-fluoronaphthalen-1-ol (21-Cl)

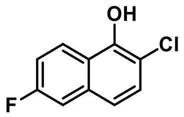

Isolated: Colorless solid, 116 mg (78%)

TLC (20% diethyl ether in pentane): R_f = 0.80 (UV, $KMnO_4$).

mp: 82–85 °C.

^{1}H NMR (400 MHz, chloroform-*d*) δ 8.10 (dd, *J* = 9.2, 5.6 Hz, 1H), 7.29 – 7.23 (m, 2H), 7.20 – 7.13 (m, 2H), 5.92 (s, 1H).

^{13}C NMR (101 MHz, chloroform-*d*) δ 161.3 (d, *J* = 247 Hz), 147.3 (d, *J* = 1.3 Hz), 134.3 (d, *J* = 9.4 Hz), 127.2, 124.9 (d, *J* = 9.2 Hz), 121.4, 120.2 (d, *J* = 5.1 Hz), 116.3 (d, *J* = 25.3 Hz), 112.9 (d, *J* = 2.8 Hz), 110.9 (d, *J* = 20.9 Hz).

IR (Diamond-ATR, neat) $\tilde{\upsilon}_{max}$: 3521 (w), 1632 (m), 1511 (vs), 1257 (s), 955 (m), 867 (w) cm^{-1}.

HRMS (ESI) calc. for $C_{10}H_5ClFO$ $[M-H]^-$: 195.0018 found: 195.0018.

5-Iodo-2-chloronaphthalen-1-ol (22-Cl)

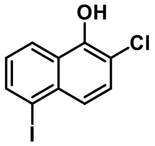

Isolated: Colorless solid, 178 mg (77%)

TLC (20% diethyl ether in pentane): R_f = 0.80 (UV, $KMnO_4$).

mp: 79–80 °C.

^{1}H NMR (400 MHz, chloroform-*d*) δ 8.24 (dt, *J* = 8.4, 1.0 Hz, 1H), 8.08 (dd, *J* = 7.3, 1.2 Hz, 1H), 7.62 (dd, *J* = 9.1, 0.9 Hz, 1H), 7.43 (d, *J* = 9.1 Hz, 1H), 7.18 (dd, *J* = 8.4, 7.3 Hz, 1H), 6.02 (s, 1H).

^{13}C NMR (101 MHz, chloroform-*d*) δ 146.9, 138.4, 134.1, 127.3, 127.0, 125.3, 124.8, 123.1, 114.6, 98.9.

IR (Diamond-ATR, neat) $\tilde{\upsilon}_{max}$: 3430 (w), 1455 (s), 1245 (vs), 1189 (m), 1147 (s), 1070 (m) cm^{-1}.

HRMS (ESI) calc. for $C_{10}H_5OICl$ $[M-H]^-$: 302.9079 found: 302.9080.

2-Chloro-7-(trifluoromethyl)naphthalen-1-ol (23-Cl)

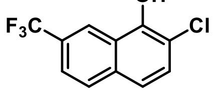

Isolated: Colorless solid, 103 mg (55%)

TLC (20% diethyl ether in pentane): R_f = 0.69 (UV, $KMnO_4$).

mp: 98–99 °C.

^{1}H NMR (400 MHz, chloroform-*d*) δ 8.54 (s, 1H), 7.88 (d, *J* = 8.6 Hz, 1H), 7.68 – 7.66 (dd, *J* = 8.7, 1.0 Hz, 1H), 7.50 (d, *J* = 8.8 Hz, 1H), 7.43 (d, *J* = 8.8 Hz, 1H), 6.11 (s, 1H).

^{13}**C NMR** (101 MHz, chloroform-*d*) δ 174.9, 134.3, 128.8, 128.3, 128.0 (q, *J* = 32.5 Hz), 124.3 (q, *J* = 272 Hz), 123.4, 122.4 (q, *J* = 3.1 Hz), 120.8, 120.4 (q, *J* = 4.6 Hz), 114.9.

IR (Diamond-ATR, neat) $\tilde{\upsilon}_{max}$: 3399 (w), 1458 (m), 1301 (m), 1243 (m), 1078 (w), 909 (w) cm^{-1}.

HRMS (ESI) calc. for $C_{11}H_5ClF_3O$ [M-H]$^-$: 244.9987 found: 244.9987.

8-Bromo-2-chloro-5-fluoronaphthalen-1-ol (24-Cl)

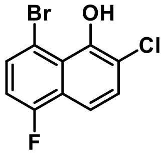

Isolated: Yellow solid, 157 mg (75%)

TLC (20% diethyl ether in pentane): R_f = 0.80 (UV, $KMnO_4$).

mp: 92–94 °C.

^{1}**H NMR** (400 MHz, chloroform-*d*) δ 7.69 – 7.64 (m, 2H), 7.55 – 7.51 (m, 2H), 6.99 (t, *J* = 8.9, Hz, 1H).

^{13}**C NMR** (101 MHz, chloroform-*d*) δ 158.4 (d, *J* = 253 Hz), 147.7 (d, *J* = 3.5 Hz), 132.6 (d, *J* = 8.5 Hz), 128.5 (d, *J* = 1.9 Hz), 125.8 (d, *J* = 17.6 Hz), 122.8 (d, *J* = 4.1 Hz), 118.9, 114.0 (d, *J* = 8.2 Hz), 110.8 (d, *J* = 21.7 Hz), 110.1 (d, *J* = 4.4 Hz).

IR (Diamond-ATR, neat) $\tilde{\upsilon}_{max}$: 3460 (m), 3089 (w), 1445 (s), 1416 (m), 1140 (m), 920 (w) cm^{-1}.

HRMS (ESI) calc. for $C_{10}H_4BrClFO$ [M-H]$^-$: 272.9124 found: 272.9124.

2-Chloro-6-(methoxymethoxy)naphthalen-1-ol (25-Cl)

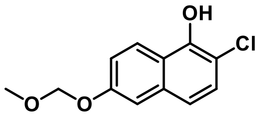

Isolated: Brown solid, 119 mg (66%)

TLC (20% diethyl ether in pentane): R_f = 0.79 (UV, $KMnO_4$).

mp: 261 °C (decomposition).

^{1}**H NMR** (400 MHz, chloroform-*d*) δ 8.14 (d, *J* = 9.1 Hz, 1H), 7.33 (d, *J* = 3.3 Hz, 1H), 7.31 (d, *J* = 10.1 Hz, 1H), 7.27 – 7.21 (m, 2H), 6.01 (s, 1H), 5.30 (s, 2H), 3.53 (s, 3H).

^{13}**C NMR** (101 MHz, chloroform-*d*) δ 155.8, 147.2, 134.7, 126.7, 123.9, 120.4, 120.1, 119.0, 112.0, 109.9, 94.5, 56.3.

IR (Diamond-ATR, neat) $\tilde{\upsilon}_{max}$: 3550 (w), 3060, (m) 1472 (m), 1320 (vs), 1068 (m), 855 (w) cm^{-1}.

HRMS (ESI) calc. for $C_{12}H_{10}ClO_3$ [M-H]$^-$: 237.0324 found: 237.0323.

5-(Benzyloxy)-2-chloronaphthalen-1-ol (26-Cl)

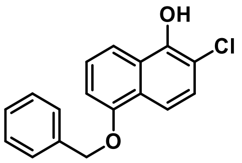

Isolated: Yellowish gum, 153 mg (71%)

TLC (20% diethyl ether in pentane): R_f = 0.50 (UV, $KMnO_4$).

^{1}H NMR (400 MHz, chloroform-*d*) δ 7.87 (dd, *J* = 9.0, 0.89 Hz, 1H), 7.80 (dt, *J* = 8.4, 0.9 Hz, 1H), 7.52 – 7.51 (m, 2H), 7.44 – 7.40 (m, 3H), 7.37 – 7.34 (m, 2H), 6.93 (dd, *J* = 7.7, 0.9 Hz, 1H), 5.97 (s, 1H), 5.24 (s, 2H).

^{13}C NMR (101 MHz, chloroform-*d*) δ 154.5, 146.9, 137.0, 128.8, 128.2, 127.5, 126.4, 125.7, 125.2, 115.6, 114.7, 114.6, 106.3, 70.4. (*Note: One ^{13}C-signal is missing due to overlap.)*

IR (Diamond-ATR, neat) $\tilde{\upsilon}_{max}$: 3550 (w), 1512 (vs), 1457 (s), 1110 (m), 1008 (w) cm^{-1}.

HRMS (EI) calc. for $C_{17}H_{13}ClO_2$ $[M]^+$: 284.0599 found: 284.0599.

2-Chloro-6,7-dimethoxynaphthalen-1-ol (27-Cl)

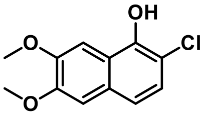

Isolated: Grey solid, 150 mg (83%)

TLC (50% dichloromethane in cyclohexane): R_f = 0.23 (UV, CAM).

mp: 99–103 °C.

^{1}H NMR (400 MHz, chloroform-*d*) δ 7.45 (s, 1H), 7.20 (d, *J* = 8.7 Hz, 1H), 7.17 (d, *J* = 8.8 Hz, 1H), 7.01 (s, 1H), 6.05 (s, 1H), 4.00 (s, 3H), 3.96 (s, 3H).

^{13}C NMR (101 MHz, chloroform-*d*) δ 150.0, 149.6, 146.0, 129.2, 124.2, 119.8, 119.4, 112.4, 106.3, 101.0, 56.0, 55.9.

IR (Diamond-ATR, neat) $\tilde{\upsilon}_{max}$: 3525 (w), 3071 (w), 1427 (vs), 1219 (m), 1163 (s), 869 (s) cm^{-1}.

HRMS (ESI) calc. for $C_{12}H_{10}ClO_3$ $[M-H]^-$: 237.0324 found: 237.0322.

Note: Higher yields were obtained following the general procedure B using chloroform instead of bromoform.

2-Chloro-6-methoxynaphthalen-1-ol (28-Cl)

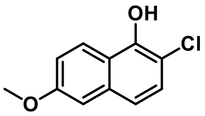

Isolated: Colorless solid, 128 mg (81%)

TLC (20% diethyl ether in pentane): R_f = 0.58 (UV, $KMnO_4$).

mp: 72–74 °C.

^{1}H NMR (400 MHz, chloroform-*d*) δ 7.97 (d, *J* = 9.2 Hz, 1H), 7.16 (d, *J* = 8.8 Hz, 1H), 7.08 (d, *J* = 8.9 Hz, 1H), 7.02 (dd, *J* = 9.2, 2.5 Hz, 1H), 6.89 (d, *J* = 2.4 Hz, 1H), 5.86 (s, 1H), 3.75 (s, 3H).

^{13}C NMR (101 MHz, chloroform-*d*) δ 158.3, 147.3, 134.8, 126.6, 123.8, 119.8, 119.7, 118.7, 111.6, 105.8, 55.4.

IR (Diamond-ATR, neat) $\tilde{\upsilon}_{max}$: 3512 (w), 3069 (m), 1431 (m), 1257 (m), 1025 (m), 871 (s) cm^{-1}.

HRMS (ESI) calc. for $C_{11}H_8ClO_2$ [M-H]$^-$: 207.0218 found: 207.0219.

Note: Higher yields were obtained following the general procedure B using chloroform instead of bromoform.

6-((*Tert*-butyldimethylsilyl)oxy)-2-chloronaphthalen-1-ol (29-Cl)

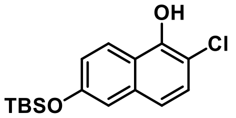

Isolated: Colorless oil, 140 mg (60%)

TLC (20% diethyl ether in pentane): R_f = 0.92 (UV, $KMnO_4$).

^{1}H NMR (400 MHz, chloroform-*d*) δ 8.13 (d, *J* = 8.9 Hz, 1H), 7.31 (d, *J* = 8.8 Hz, 1H), 7.23 (d, *J* = 8.9 Hz, 1H), 7.16 – 7.10 (m, 2H), 5.97 (s, 1H), 1.05 (s, 9H), 0.27 (s, 6H).

^{13}C NMR (101 MHz, chloroform-*d*) δ 154.4, 147.3, 134.9, 126.5, 123.9, 122.2, 120.2, 119.8, 115.0, 111.7, 25.8, 18.4, –4.2.

IR (Diamond-ATR, neat) $\tilde{\upsilon}_{max}$: 3538 (w), 2955 (m), 1629 (vs), 1431 (s), 1159 (s), 972 (s), 858 (m) cm^{-1}.

HRMS (ESI) calc. for $C_{16}H_{20}ClO_2Si$ [M-H]$^-$: 307.0926 found: 307.0927.

6-Chloro-5-hydroxynaphthalen-1-yl benzoate (30-Cl)

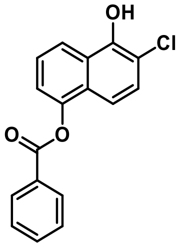

Isolated: Colorless solid, 177 mg (78%)

TLC (20% diethyl ether in pentane): R_f = 0.78 (UV, $KMnO_4$).

mp: 138–140 °C (decomposition).

^{1}H NMR (400 MHz, chloroform-*d*) δ 8.33 (d, *J* = 7.6 Hz, 2H), 8.16 (d, *J* = 8.5 Hz, 1H), 7.70 (t, *J* = 7.4 Hz, 1H), 7.57 (q, *J* = 8.0 Hz, 3H), 7.46 (d, *J* = 9.1 Hz, 1H), 7.40 (d, *J* = 7.5 Hz, 1H), 7.37 (d, *J* = 9.1 Hz, 1H), 6.09 (s, 1H).

^{13}C NMR (101 MHz, chloroform-*d*) δ 165.2, 147.4, 146.8, 134.1, 130.5, 129.3, 128.9, 127.0, 126.7, 126.1, 125.9, 120.5, 119.4, 114.6, 114.6.

IR (Diamond-ATR, neat) $\tilde{\upsilon}_{max}$: 3560 (m), 1730 (vs), 1322 (vs), 1320 (w), 1068 (m), 859 (w) cm^{-1}.

HRMS (ESI) calc. for $C_{17}H_{10}ClO_3$ [M-H]$^-$: 297.0324 found: 297.0324.

6-Chloro-5-hydroxynaphthalen-2-yl pivalate (31-Cl)

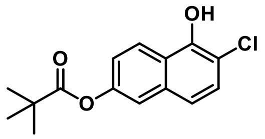

Isolated: Colorless solid, 133 mg (63%)

TLC (20% diethyl ether in pentane): R_f = 0.82 (UV, $KMnO_4$).

mp: 141–143 °C (decomposition).

^{1}H NMR (400 MHz, chloroform-*d*) δ 8.20 (d, *J* = 9.1 Hz, 1H), 7.46 (d, *J* = 2.0 Hz, 1H), 7.35 (d, *J* = 8.8 Hz, 1H), 7.29 (d, *J* = 8.9 Hz, 1H), 7.22 (dd, *J* = 9.1, 2.1 Hz, 1H), 6.05 (s, 1H), 1.41 (s, 9H).

^{13}C NMR (101 MHz, chloroform-*d*) δ 177.4, 149.6, 147.3, 133.9, 126.9, 124.0, 122.5, 121.4, 120.6, 118.4, 113.5, 39.3, 27.3.

IR (Diamond-ATR, neat) $\tilde{\upsilon}_{max}$: 3426 (w), 2956 (m), 1730 (vs), 1389 (s), 1067 (s), 869 (m) cm^{-1}.

HRMS (ESI) calc. for $C_{15}H_{14}ClO_3$ [M-H]$^-$: 277.0637 found: 277.0639.

2-Chloro-4-phenylnaphthalen-1-ol (32-Cl)

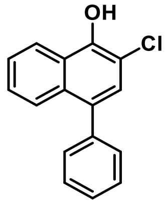

Isolated: Colorless solid, 158 mg (82%)

TLC (20% diethyl ether in pentane): R_f = 0.67 (UV, $KMnO_4$).

mp: 123–125 °C.

^{1}H NMR (400 MHz, chloroform-*d*) δ 8.32 (d, *J* = 8.3 Hz, 1H), 7.85 (d, *J* = 8.5 Hz, 1H), 7.55 (t, *J* = 7.5Hz, 1H), 7.52 – 7.41 (m, 6H), 7.36 (s, 1H), 6.04 (s, 1H).

^{13}C NMR (101 MHz, chloroform-*d*) δ 146.6, 139.6, 133.9, 131.5, 130.3, 128.5, 127.5, 126.9, 126.7, 126.2, 124.7, 122.5, 113.2. (*Note: One ^{13}C-signal is missing due to overlap.)*

IR (Diamond-ATR, neat) $\tilde{\upsilon}_{max}$: 3513 (w), 3057 (w), 1587 (m), 1220 (s), 1068 (m), 850 (s) cm^{-1}.

HRMS (ESI) calc. for $C_{16}H_{10}ClO$ [M-H]$^-$: 253.0426 found: 253.0427.

2-Chloro-3-methylnaphthalen-1-ol (33-Cl)

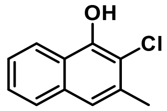

Isolated: Colorless solid, 110 mg (75%)

TLC (20% diethyl ether in pentane): R_f = 0.90 (UV, $KMnO_4$).

mp: 68–70 °C.

^{1}H NMR (400 MHz, chloroform-*d*) δ 8.32 – 8.07 (m, 1H), 7.77 – 7.64 (m, 1H), 7.53 – 7.37 (m, 2H), 7.29 (s, 1H), 6.08 (s, 1H), 2.52 (s, 3H).

^{13}C NMR (101 MHz, chloroform-*d*) δ 147.1, 133.4, 132.6, 126.9, 126.8, 125.2, 122.9, 122.1, 120.6, 115.6, 20.8.

IR (Diamond-ATR, neat) $\tilde{\upsilon}_{max}$: 3517 (w), 3056 (m), 1590 (vs), 1230 (m), 1224 (s), 1033 (w) cm^{-1}.

HRMS (ESI) calc. for $C_{11}H_{8}ClO$ [M-H]$^{-}$: 191.0269 found: 191.0269.

2.5 Substrate Scope (2-Bromo-1-naphthols)

((2-Bromonaphthalen-1-yl)oxy)(*tert*-butyl)dimethylsilane (16-TBS)

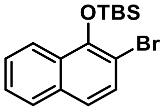

Isolated: Colorless oil, 60.9 mg (43%, see Table 2)

TLC (pentane): R_f = 0.64 (UV, CAM).

^{1}H NMR (400 MHz, chloroform-*d*) δ 8.08 (dt, *J* = 6.8, 3.3 Hz, 1H), 7.78 (dq, *J* = 6.0, 3.4 Hz, 1H), 7.55 (d, *J* = 8.8 Hz, 1H), 7.47 (dt, *J* = 6.4, 3.4 Hz, 2H), 7.36 (d, *J* = 8.7 Hz, 1H), 1.14 (s, 9H), 0.30 (s, 6H).

^{13}C NMR (101 MHz, chloroform-*d*) δ 148.9, 134.1, 130.6, 129.0, 127.9, 126.4, 125.7, 123.2, 122.7, 110.1, 26.4, 19.1, –2.5.

IR (Diamond-ATR, neat) $\tilde{\upsilon}_{max}$: 2939 (w), 1639 (vs), 1602 (m), 1431 (s), 972 (s), 858 (m) cm^{-1}.

HRMS (ESI) calc. for $C_{16}H_{22}BrOSi$ [M+H]$^{+}$: 337.0618 found: 337.0599.

2-Bromonaphthalen-1-ol (18-Br)

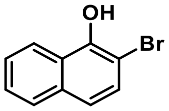

Isolated: 79.6 mg (85%)

Data consistent with literature: *J. Org. Chem.*, **2018**, *83*, 8036–8053.

2-Bromo-7-chloronaphthalen-1-ol (19-Br)

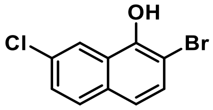

Isolated: Off-white solid, 88.9 mg (82%)

TLC (25% dichloromethane in cyclohexane): R_f = 0.50 (UV, CAM).

mp: 78–81 °C.

^{1}H NMR (400 MHz, chloroform-*d*) δ 8.22 (dd, *J* = 2.1, 0.8 Hz, 1H), 7.71 (d, *J* = 8.8 Hz, 1H), 7.48 (d, *J* = 8.8 Hz, 1H), 7.45 (dd, *J* = 8.8, 2.2 Hz, 1H), 7.29 (d, *J* = 8.8 Hz, 1H), 5.97 (s, 1H).

^{13}**C NMR** (101 MHz, chloroform-*d*) δ 147.6, 132.3, 132.1, 129.3, 128.8, 127.9, 125.1, 121.7, 121.2, 105.2.

IR (Diamond-ATR, neat) $\tilde{\upsilon}_{max}$: 3466 (m), 1589 (w), 1566 (m), 1265 (vs), 808 (w) cm^{-1}.

HRMS (ESI) calc. for $C_{10}H_5BrClO$ [M-H]$^{-}$: 254.9218 found: 254.9214.

2,5-Dibromophthalen -1-ol (20-Br)

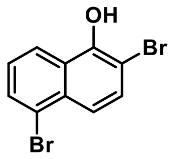

Isolated: 110 mg (87%)

Data consistent with literature: *Tetrahedron Lett.*, **2005**, *46*, 4187–4191.

2-Bromo-6-fluoronaphthalen-1-ol (21-Br)

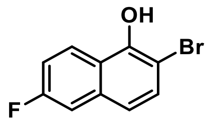

Isolated: 90.1 mg (89%)

Data consistent with literature: *Org. Biomol. Chem.*, **2004**, *2*, 3018–3025.

2-Bromo-5-iodonaphthalen-1-ol (22-Br)

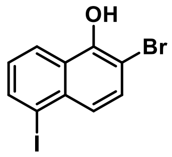

Isolated: Beige solid, 106 mg (72%)

TLC (25% ethyl acetate in cyclohexane): R_f = 0.55 (UV, CAM).

mp: 61–63 °C.

^{1}**H NMR** (400 MHz, chloroform-*d*) δ 8.25 (dt, *J* = 8.3, 0.9 Hz, 1H), 8.09 (dd, *J* = 7.3, 1.1 Hz, 1H), 7.57 (dd, *J* = 9.1, 0.7 Hz, 1H), 7.54 (d, *J* = 9.2 Hz, 1H), 7.17 (dd, *J* = 8.4, 7.3 Hz, 1H), 5.99 (s, 1H).

^{13}**C NMR** (101 MHz, chloroform-*d*) δ 148.1, 138.6, 134.7, 129.9, 127.1, 125.8, 124.8, 123.4, 105.1, 98.9.

IR (Diamond-ATR, neat) $\tilde{\upsilon}_{max}$: 3332 (w), 1447 (s), 1409 (m), 1241 (vs), 1074 (s), 999 (m) cm^{-1}.

HRMS (ESI) calc. for $C_{10}H_5BrIO$ [M-H]$^{-}$: 346.8574 found: 346.8570.

2-Bromo-7-(trifluoromethyl)naphthalen-1-ol (23-Br)

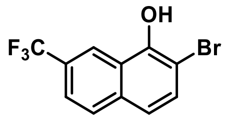

Isolated: White solid, 75.8 mg (62%)

TLC (cyclohexane): R_f = 0.27 (UV, CAM).

mp: 83 °C.

^{1}H NMR (400 MHz, chloroform-*d*) δ 8.58 – 8.54 (m, 1H), 7.88 (d, *J* = 8.4 Hz, 1H), 7.67 (dd, *J* = 8.6, 1.9 Hz, 1H), 7.61 (d, *J* = 8.8 Hz, 1H), 7.37 (d, *J* = 8.8 Hz, 1H), 6.08 (s, 1H).

^{13}C NMR (101 MHz, Chloroform-*d*) δ 149.1, 135.0 (d, *J* = 0.8 Hz), 130.9, 128.8, 128.1 (q, *J* = 32.5 Hz), 124.4 (q, *J* = 272 Hz), 123.4, 122.6 (q, *J* = 3.1 Hz), 121.2, 120.7 (q, *J* = 4.6 Hz), 105.4.

^{19}F NMR (376 MHz, chloroform-*d*, $CFCl_3$ as internal standard) –62.3 (s).

IR (Diamond-ATR, neat) $\tilde{\upsilon}_{max}$: 3359 (w), 3298 (m), 1397 (s), 1129 (vs), 1103 (m), 909 (w) cm^{-1}.

HRMS (ESI) calc. for $C_{11}H_6BrF_3O$ $[M-H]^-$: 288.9481 found: 288.9480.

2,8-Bibromo-5-fluoronaphthalen-1-ol (24-Br)

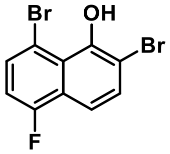

Isolated: Colorless crystals, 90.0 mg (67%)

TLC (13% ethyl acetate in cyclohexane): R_f = 0.56 (UV, CAM).

mp: 72 °C.

^{1}H NMR (400 MHz, chloroform-*d*) δ 7.68 – 7.61 (m, 3H), 7.57 (dd, *J* = 9.0, 0.8 Hz, 1H), 6.98 (dd, *J* = 9.4, 8.3 Hz, 1H).

^{13}C NMR (101 MHz, chloroform-*d*) δ 158.5 (d, *J* = 253 Hz), 148.6 (d, *J* = 3.5 Hz), 132.4 (d, *J* = 8.5 Hz), 131.2 (d, *J* = 1.8 Hz), 126.2 (d, *J* = 17.5 Hz), 122.7 (d, *J* = 4.2 Hz), 114.2 (d, *J* = 8.1 Hz), 110.9 (d, *J* = 21.7 Hz), 110.0 (d, *J* = 4.4 Hz), 109.2 (d, *J* = 0.7 Hz).

^{19}F NMR (376 MHz, chloroform-*d*, $CFCl_3$ as internal standard) δ –119.9 (dd, *J* = 9.4, 5.3 Hz).

IR (Diamond-ATR, neat) $\tilde{\upsilon}_{max}$: 3391 (w), 3069 (w), 1554 (vs), 1425 (s), 1120 (m), 880 (w) cm^{-1}.

HRMS (ESI) calc. for $C_{10}H_4Br_2FO$ $[M-H]^-$: 316.8618 found: 316.8622.

2-Bromo-6-(methoxymethoxy)naphthalen-1-ol (25-Br)

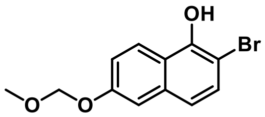

Isolated: Brownish oil, 53.5 mg (45%)

TLC (14% ethyl acetate in cyclohexane): R_f = 0.44 (UV, CAM).

^{1}H NMR (400 MHz, chloroform-*d*) δ 8.16 (dt, *J* = 9.2, 0.7 Hz, 1H), 7.42 (d, *J* = 8.8 Hz, 1H), 7.33 (d, *J* = 2.4 Hz, 1H), 7.25 – 7.19 (m, 2H), 5.95 (s, 1H), 5.29 (s, 2H), 3.53 (s, 3H).

^{13}C NMR (101 MHz, chloroform-*d*) δ 156.0, 148.4, 135.2, 129.1, 124.2, 120.7, 120.4, 119.0, 109.9, 102.2, 94.6, 56.3.

IR (Diamond-ATR, neat) $\tilde{\upsilon}_{max}$: 3552 (w), 3160, (w) 1618 (s), 1335 (m), 1230 (s), 775 (w) cm^{-1}.

HRMS (ESI) calc. for $C_{12}H_{10}BrO_3$ [M-H]$^-$: 280.9819 found: 280.9822.

5-(Benzyloxy)-2-bromonaphthalen-1-ol (26-Br)

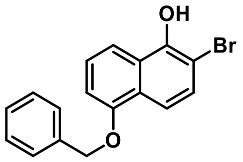

Isolated: White solid, 96.7 mg (70%)

TLC (20% diethyl ether in pentane): R_f = 0.50 (UV, $KMnO_4$).

mp: 89 °C.

^{1}H NMR (400 MHz, chloroform-*d*) δ 7.87 (dd, *J* = 9.0, 0.89 Hz, 1H), 7.80 (dt, *J* = 8.4, 0.9 Hz, 1H), 7.52 – 7.51 (m, 2H), 7.44 – 7.40 (m, 3H), 7.37 – 7.34 (m, 2H), 6.93 (dd, *J* = 7.7, 0.9 Hz, 1H), 5.97 (s, 1H), 5.24 (s, 2H).

^{13}C NMR (101 MHz, chloroform-*d*) δ 154.5, 148.0, 137.0, 128.8, 128.2, 127.7, 127.5, 126.5, 126.2, 125.7, 116.0, 114.9, 106.4, 105.3, 70.4.

IR (Diamond-ATR, neat) $\tilde{\upsilon}_{max}$: 3546 (w), 3399 (w), 1701 (s), 1461 (vs), 1343 (m), 948 (m) cm^{-1}.

HRMS (ESI) calc. for $C_{17}H_{12}BrO_2$ [M-H]$^-$: 327.0026 found: 327.0028.

2-Bromo-6,7-dimethoxynaphthalen-1-ol (27-Br)

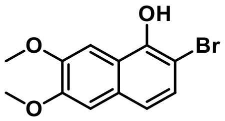

Isolated: 52.3 mg (44%)

Data consistent with literature: *Tetrahedron*, **1998**, *54*, 9875–9894.

2-Bromo-6-methoxynaphthalen-1-ol (28-Br)

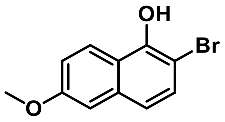

Isolated: 49.9 mg (47%)

Data consistent with literature: *Tetrahedron Lett.*, **2005**, *46*, 4187–4191.

2-Bromo-6-((tert-butyldimethylsilyl)oxy)naphthalen-1-ol (29-Br)

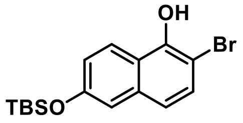

Isolated: White solid, 34.1 mg (23%)

TLC (13% dichloromethane in cyclohexane): R_f = 0.38 (UV, $KMnO_4$).

mp: 58 °C.

^{1}H NMR (400 MHz, chloroform-*d*) δ 8.12 (d, *J* = 9.0 Hz, 1H), 7.40 (d, *J* = 8.8 Hz, 1H), 7.16 (d, *J* = 8.9 Hz, 1H), 7.12 (d, *J* = 2.3 Hz, 1H), 7.09 (dd, *J* = 9.0, 2.4 Hz, 1H), 5.90 (s, 1H), 1.02 (s, 9H), 0.25 (s, 6H).

^{13}C NMR (101 MHz, chloroform-*d*) δ 154.6, 148.4, 135.5, 128.9, 124.1, 122.2, 120.3, 120.2, 115.0, 101.9, 25.8, 18.4, –4.2.

IR (Diamond-ATR, neat) $\tilde{\upsilon}_{max}$: 3499 (w), 2945 (m), 1702 (s), 1588 (vs), 1420 (s), 970 (s), 845 (m) cm^{-1}.

HRMS (ESI) calc. for $C_{16}H_{20}BrO_2Si$ [M-H]$^{-}$: 351.0421 found: 351.0424.

*Note: **29-Br** was obtained in 23% along with 30% of unprotected 6-hydroxynaphthol S1-Br (see next) following protocol B.*

2-Bromonaphthalene-1,6-diol (S1-Br)

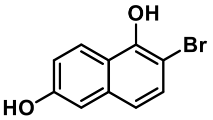

Isolated: White solid, 30.1 mg (30%)

TLC (57% dichloromethane in cyclohexane): R_f = 0.12 (UV, $KMnO_4$).

mp: 90 °C.

^{1}H NMR (400 MHz, chloroform-*d*) δ 8.14 (d, *J* = 8.9 Hz, 1H), 7.41 (d, *J* = 8.8 Hz, 1H), 7.14 (d, *J* = 8.9 Hz, 1H), 7.12 – 7.06 (m, 2H), 5.93 (s, 1H), 5.19 (s, 1H).

^{13}C NMR (101 MHz, chloroform-*d*) δ 154.4, 148.5, 135.4, 129.3, 124.7, 120.0, 119.9, 117.8, 109.6, 101.7.

IR (Diamond-ATR, neat) $\tilde{\upsilon}_{max}$: 3451 (w), 1624 (s), 1587 (vs), 1385 (m), 1230 (s), 972 (s) cm^{-1}.

HRMS (ESI) calc. for $C_{10}H_6BrO_2$ [M-H]$^{-}$: 236.9557 found: 236.9561.

((6-bromo-5-((triisopropylsilyl)oxy)naphthalen-2-yl)oxy)(tert-butyl)dimethylsilane (S2-Br)

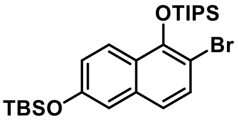

Isolated: Yellow oil, 126 mg (59%)

TLC (13% dichloromethane in cyclohexane): R_f = 0.63 (UV, CAM).

^{1}H NMR (400 MHz, chloroform-*d*) δ 7.99 (d, *J* = 9.1 Hz, 1H), 7.46 (d, *J* = 8.8 Hz, 1H), 7.18 (d, *J* = 8.8 Hz, 1H), 7.12 (d, *J* = 2.4 Hz, 1H), 7.07 (dd, *J* = 9.0, 2.4 Hz, 1H), 1.53 (h, *J* = 7.4 Hz, 3H), 1.14 (d, *J* = 7.5 Hz, 18H), 1.02 (s, 9H), 0.25 (s, 6H).

^{13}C NMR (101 MHz, $CDCl_3$) δ 154.0, 150.1, 135.5, 131.0, 124.8, 124.5, 121.9, 121.2, 115.1, 107.4, 25.8, 18.4, 18.2, 14.6, -4.2.

IR (Diamond-ATR, neat) $\tilde{\upsilon}_{max}$: 3318 (w), 2850 (m), 1601 (s), 1331 (s), 841 (w) cm^{-1}.

HRMS (ESI) calc. for $C_{25}H_{42}BrO_2Si_2$ [M+H]$^{+}$: 509.1901 found: 509.1903.

*Note: **S2-Br** was obtained in 59% following protocol B omitting the deprotection step.*

6-Bromo-5-hydroxynaphthalen-2-yl pivalate (31-Br)

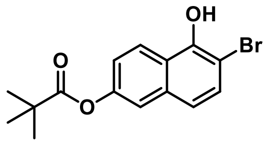

Isolated: Colorless solid, 14.9 mg (11%)

TLC (50% dichloromethane in cyclohexane): R_f = 0.51 (UV, CAM).

mp: 122–124 °C.

^{1}H NMR (400 MHz, chloroform-*d*) δ 8.01 (d, *J* = 9.1 Hz, 1H), 7.25 – 7.22 (m, 1H), 7.05 – 6.96 (m, 3H), 5.77 (s, 1H), 1.18 (s, 9H).

^{13}C NMR (101 MHz, chloroform-*d*) δ 177.3, 149.8, 148.5, 134.5, 129.4, 124.2, 122.4, 121.4, 121.1, 118.4, 103.8, 39.3, 27.3.

IR (Diamond-ATR, neat) $\tilde{\upsilon}_{max}$: 3420 (w), 1831 (s), 1670 (vs), 1393 (s), 1212 (m), 1067 (s), 889 (s) cm^{-1}.

HRMS (ESI) calc. for $C_{15}H_{14}BrO_3$ [M-H]$^-$: 321.0132 found: 321.0133.

((2-Bromo-4-phenylnaphthalen-1-yl)oxy)triisopropylsilane (S3-Br)

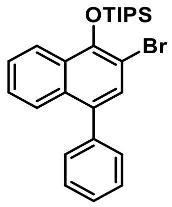

Isolated: Colorless oil, 174 mg (91%)

TLC (12% dichloromethane in cyclohexane): R_f = 0.79 (UV, CAM).

^{1}H NMR (400 MHz, chloroform-*d*) δ 8.21 (d, *J* = 8.4 Hz, 1H), 7.84 (d, *J* = 8.2 Hz, 1H), 7.57 – 7.39 (m, 8H), 1.60 (h, *J* = 7.5 Hz, 3H), 1.20 (d, *J* = 7.6 Hz, 18H).

^{13}C NMR (101 MHz, chloroform-*d*) δ 149.5, 139.6, 134.9, 132.1, 131.2, 130.3, 129.2, 128.4, 127.5, 126.5, 126.4, 125.9, 123.1, 109.0, 18.3, 14.6.

IR (Diamond-ATR, neat) $\tilde{\upsilon}_{max}$: 3518 (w), 2957 (m), 1587 (vs), 1456 (s), 1340 (vs), 850 (w) cm^{-1}.

HRMS (ESI) calc. for $C_{25}H_{32}BrOSi$ [M+H]$^+$: 455.1400 found: 455.1407.

*Note: **S3-Br** was obtained in 91% following protocol B omitting the deprotection step.*

2-Bromo-4-phenylnaphthalen-1-ol (32-Br)

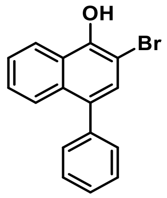

Isolated: yellow-brown solid in oily film (crude mixture)

TLC (12% dichloromethane in cyclohexane): R_f = 0.45 (UV, CAM).

mp: 118 °C.

^{1}H NMR (400 MHz, chloroform-*d*) δ 8.33 (d, *J* = 8.2 Hz, 1H), 7.83 (d, *J* = 8.5 Hz, 1H), 7.59 – 7.38 (m, 8H), 6.01 (s, 1H).

^{13}C NMR (101 MHz, chloroform-*d*) δ 147.8, 139.5, 134.3, 132.1, 130.3, 129.1, 128.5, 127.5, 127.0, 126.2, 126.2, 124.7, 122.7, 103.7.

IR (Diamond-ATR, neat) $\tilde{\upsilon}_{max}$: 3522 (w), 3157 (w), 1534 (s), 1457 (vs), 1113 (m), 797 (m) cm^{-1}.

HRMS (ESI) calc. for $C_{16}H_{10}BrO$ $[M-H]^-$: 296.9921 found: 296.9924.

*Note: **32-Br** turned out to be highly sensitive to oxygen and silica, thus only partial purification was possible.*

2-Bromo-3-methylnaphthalen-1-ol (33-Br)

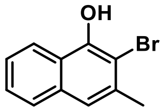

Isolated: Orange crystals, 57.7 mg (58%)

TLC (25% dichloromethane in cyclohexane): R_f = 0.50 (UV, CAM).

mp: 60–63 °C.

^{1}H NMR (400 MHz, chloroform-*d*) δ 8.19 (d, *J* = 7.9 Hz, 1H), 7.70 (d, *J* = 7.5 Hz, 1H), 7.54 – 7.40 (m, 2H), 7.31 (t, *J* = 1.1 Hz, 1H), 6.09 (s, 1H), 2.54 (d, *J* = 1.0 Hz, 3H).

^{13}C NMR (101 MHz, chloroform-*d*) δ 148.3, 134.7, 133.2, 127.1, 126.9, 125.3, 122.7, 122.4, 120.6, 108.4, 23.7.

IR (Diamond-ATR, neat) $\tilde{\upsilon}_{max}$: 3243 (w), 3021 (m), 1691 (s), 1321 (vs), 1124 (m), 903 (s) cm^{-1}.

HRMS (ESI) calc. for $C_{11}H_8BrO$ $[M-H]^-$: 234.9764 found: 234.9759.

3. Synthesis of Defucogilvocarcin M

3.1 Synthesis of linear precursor 40

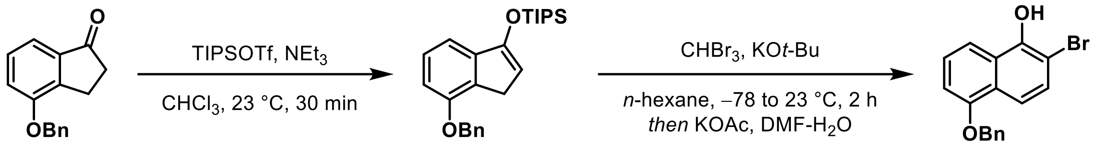

34 **15-TIPS** **35**

5-(Benzyloxy)-2-bromonaphthalen-1-ol (26-Br)

Triethylamine (833 µL, 6.01 mmol, 1.40 equiv) and triisopropylsilyl trifluoromethanesulfonate (1.27 mL, 4.72 mmol, 1.10 equiv) were added in sequence to a solution of indanone **34** (1.02 g, 4.29 mmol, 1 equiv) in chloroform (53.6 mL, 0.08 M) at 23 ˚C. The reaction was stirred for two hours. The mixture was diluted with 30 mL of cyclohexane, filtered through a short plug of silica and the filtrate was evaporated under reduced pressure at 23 °C to afford the crude silyl enol ether. This material was used immediately without further purification.

The crude intermediate **15-TIPS** was dissolved in *n*-hexane (6.13 mL, 0.7 M), cooled to –78 °C and slowly added to a suspension of potassium *tert*-butoxide (sublimed grade, 2.17 g, 19.3 mmol, 4.50 equiv) in *n*-hexane (8.78 mL, 2.2 M) at –78 ˚C. The flask of the crude intermediate was rinsed for three times (3 × 5.11 mL) and added to the reaction in the same fashion. After 20 minutes, a solution of freshly distilled bromoform (751 µL, 8.58 mmol, 2.00 equiv) in *n*-hexane (3.90 mL, 2.2 M) was added dropwise to the mixture and stirred for one hour at –78 ˚C. The reaction was warmed to 23 °C within one hour and stirred for an additional hour at that temperature. The solvent was removed under reduced pressure to give the crude silylated 2-bromonaphthol, which was dissolved in *N*,*N*-dimethylformamide-water (20:1, 17.2 mL, 0.25 M). Potassium acetate (548 mg, 5.58 mmol, 1.30 equiv) was added and the reaction was stirred for two hours at 23 ˚C. Water (40 mL) was added to the reaction mixture and the resulting solution was extracted with diethyl ether (6 × 40 mL). The combined organic layers were washed with water (80 mL) and a saturated aqueous solution of sodium chloride (80 mL) and the washed solution was dried over magnesium sulfate. The dried solution was filtered and the filtrate was evaporated under reduced pressure. Purification by silica gel chromatography (30% grading to 70% dichloromethane in cyclohexane) gave bromonaphthol **35** (987 mg, 3.00 mmol, 70%) as a beige solid.

For analytical data: see **26-Br**.

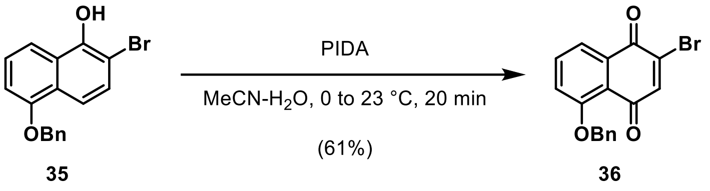

5-(Benzyloxy)-2-bromonaphthalene-1,4-dione (36)

5-(Benzyloxy)-2-bromonaphthalen-1-ol **35** (4.00 g, 12.2 mmol, 1 equiv) was dissolved in acetonitrile (203 mL, 0.061 M) upon gentle heating. Then the solution was cooled to 0 °C and a suspension of (diacetoxyiodo)benzene (8.61 g, 26.7 mmol, 2.20 equiv) in water (100 mL, 0.27 M) was slowly added to the mixture. The cooling bath was removed and the reaction was stirred for 20 minutes at 23 °C before a saturated aqueous solution of sodium hydrogen carbonate (150 mL) was added. The mixture was extracted with dichloromethane (3 × 300 mL) and the combined organic layers were washed with a saturated aqueous solution of sodium chloride (150 mL) and the washed solution was dried over magnesium sulfate. The dried solution was filtered and the filtrate was evaporated under reduced pressure. Purification by silica gel chromatography (30% grading to 80% dichloromethane in cyclohexane) gave the desired benzoquinone **36** (2.54 g, 7.41 mmol, 61%) as yellow needles.

Data consistent with literature: *J. Chem. Soc., Perkin Trans. 1*, **2001**, 1612–1623.

We observed one significant difference:

Data from Literature: **^{1}H NMR** (400 MHz, chloroform-*d*) δ 7.73 (dd, *J* = 8.5, 1.1 Hz, 1H).
Our obtained data: **^{1}H NMR** (400 MHz, chloroform-*d*) δ 7.84 (dd, *J* = 7.7, 1.0 Hz, 1H).

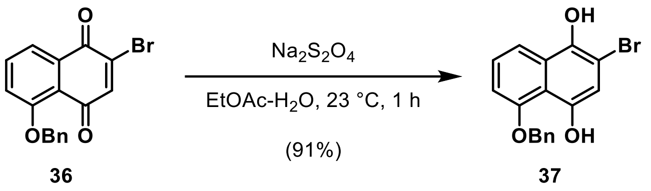

5-(Benzyloxy)-2-bromonaphthalene-1,4-diol (37)

A solution of sodium dithionite (11.5 g, 65.9 mmol, 5.22 equiv) in water (165 mL, 0.4 M) was slowly added to a solution of 5-(benzyloxy)-2-bromonaphthalene-1,4-dione **36** (4.33 g, 12.6 mmol, 1 equiv) in ethyl acetate (126 mL, 0.1 M) within ten minutes via a dropping funnel and then vigorously stirred for one hour at 23 °C. The solution was diluted with water (100 mL) and extracted with ethyl acetate (3 × 100 mL). The combined organic layers were washed with a saturated aqueous solution of sodium chloride (100 mL) and the washed solution was dried over magnesium sulfate. The dried solution was filtered and the filtrate was evaporated under reduced pressure to give the crude product. Purification was performed by silica gel chromatography (20% grading to 50% dichloromethane in cyclohexane) to afford air-sensitive dihydroquinone **37** (3.97 g, 11.5 mmol, 91%) as a brown solid.

TLC (50% dichloromethane in cyclohexane): R_f = 0.41 (CAM).

mp: 132–134 °C.

^{1}H NMR (400 MHz, chloroform-*d*) δ 9.02 (s, 1H), 7.83 (dd, *J* = 8.6, 1.0 Hz, 1H), 7.51 – 7.39 (m, 5H), 7.36 (dd, *J* = 8.6, 7.7 Hz, 1H), 6.94 (dd, *J* = 7.8, 1.0 Hz, 1H), 6.91 (s, 1H), 5.51 (s, 1H), 5.27 (s, 2H).
^{13}C NMR (101 MHz, chloroform-*d*) δ 155.3, 148.3, 141.1, 135.1, 129.3, 129.1, 128.2, 126.4, 126.3, 116.7, 115.3, 112.1, 106.7, 105.3, 72.0.
IR (Diamond-ATR, neat) $\tilde{\upsilon}_{max}$: 3203 (w), 3117 (m), 1720 (s), 1523 (s), 1222 (vs), 921 (m) cm^{-1}.
HRMS (ESI) calc. for $C_{17}H_{12}BrO_3$ [M-H]$^-$: 342.9975 found: 342.9976.

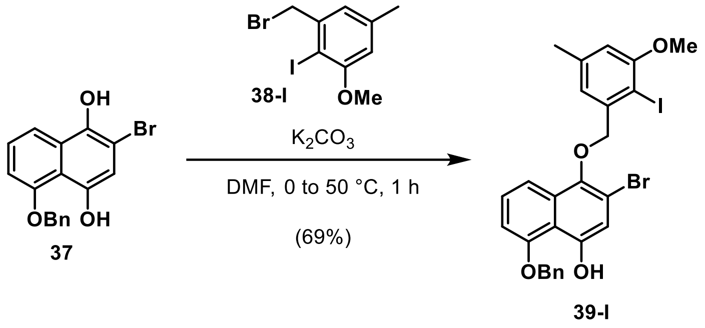

8-(Benzyloxy)-3-bromo-4-((2-iodo-3-methoxy-5-methylbenzyl)oxy)naphthalen-1-ol (39-I)

5-(Benzyloxy)-2-bromonaphthalene-1,4-diol **37** (349 mg, 1.01 mmol, 1.15 equiv) was dissolved in degassed *N,N*-dimethylformamide (760 µL, 1.33 M) and cooled to 0 °C. Potassium carbonate (140 mg, 1.01 mmol, 1.15 equiv) was added and the mixture was stirred for five minutes at 0 °C. A solution of 1-(bromomethyl)-2-iodo-3-methoxy-5-methylbenzene **38-I** (300 mg, 0.880 mmol, 1 equiv) in degassed *N,N*-dimethylformamide (700 µL, 1.26 M) was added dropwise to the reaction and rinsed with additional *N, N*-dimethylformamide (700 µL, 1.26 M). The mixture was heated to 50 °C and stirred for one hour. The mixture was allowed to cool to 23 °C and diluted with a solution of lithium chloride (4 M, 10 mL) and then extracted with ethyl acetate (3 × 50). The combined organic layers were washed with a saturated aqueous solution of sodium chloride (50 mL) and the washed solution was dried over sodium sulfate. The dried solution was filtered and the filtrate was evaporated under reduced pressure to give the crude product. Purification was performed by silica gel chromatography (33 % dichloromethane in cyclohexane) to afford quinone **39-I** (366 mg, 605 µmol, 69%) as an orange foam, accompanied by recovered benzyl bromide **38-I** (54.0 mg, 158 µmol, 18%).

TLC (50% dichloromethane in cyclohexane): R_f = 0.54 (CAM).
^{1}H NMR (400 MHz, chloroform-*d*) δ 9.29 (s, 1H), 7.75 (dd, *J* = 8.5, 0.9 Hz, 1H), 7.51 – 7.40 (m, 5H), 7.40 – 7.38 (m, 1H), 7.35 (dd, *J* = 8.6, 7.7 Hz, 1H), 7.03 (s, 1H), 6.93 (dd, *J* = 7.8, 0.9 Hz, 1H), 6.67 (s, 1H), 5.28 (s, 2H), 5.04 (s, 2H), 3.91 (s, 3H), 2.43 (s, 3H).
^{13}C NMR (101 MHz, chloroform-*d*) δ 157.8, 155.8, 151.6, 144.2, 141.2, 139.9, 135.0, 131.4, 129.3, 129.2, 128.2, 127.1, 122.1, 116.5, 115.3, 114.8, 113.9, 111.4, 106.4, 85.0, 79.3, 72.0, 56.7, 21.7.
IR (Diamond-ATR, neat) $\tilde{\upsilon}_{max}$: 3498 (m), 3390 (w), 3104 (w), 1780 (m), 1495 (s), 1394 (vs), 1123 (s), 909 (m) cm^{-1}.

HRMS (ESI) calc. for $C_{26}H_{21}BrIO_4$ $[M-H]^-$: 602.9673 found: 602.9668.

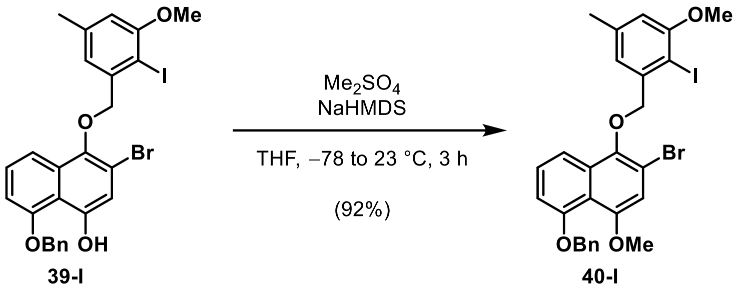

5-(Benzyloxy)-2-bromo-1-((2-iodo-3-methoxy-5-methylbenzyl)oxy)-4-methoxynaphthalene (40-I) Sodium bi(trimethylsilyl)amide (1 M in tetrahydrofuran, 1.55 mL, 1.59 mmol, 1.05 equiv) was added to a solution of naphthol **39-I** (919 mg, 1.52 mmol, 1 equiv) in tetrahydrofuran (2.17 mL, 0.7 M) at –78 °C followed by dimethyl sulfate (160 µL, 1.69 mmol, 1.11 equiv). The reaction was allowed to warm to 23 °C and stirred for three hours at that temperature. A saturated aqueous solution of sodium hydrogen carbonate (10 mL) was added, and the solution was extracted with ethyl acetate (4 × 20 mL). The combined organic layers were washed with a saturated aqueous solution of sodium chloride (15 mL) and the washed solution was dried over sodium sulfate. The dried solution was filtered, and the filtrate was evaporated under reduced pressure to give the crude product. Purification was performed by silica gel chromatography (20% grading to 70% dichloromethane in cyclohexane) to afford quinone **40-I** (864 mg, 1.40 mmol, 92%) as a white solid.

TLC (30% dichloromethane in cyclohexane): R_f = 0.54 (CAM).
mp: 94–96 °C.
^{1}H NMR (400 MHz, chloroform-*d*) δ 7.75 (dd, *J* = 8.5, 1.0 Hz, 1H), 7.59 (d, *J* = 7.4 Hz, 2H), 7.45 – 7.37 (m, 4H), 7.34 (t, *J* = 7.3 Hz, 1H), 6.98 (dd, *J* = 7.8, 1.0 Hz, 1H), 6.97 (s, 1H), 6.68 (d, *J* = 1.9 Hz, 1H), 5.21 (s, 2H), 5.07 (s, 2H), 3.95 (s, 3H), 3.92 (s, 3H), 2.44 (s, 3H).
^{13}C NMR (101 MHz, chloroform-*d*) δ 157.8, 156.7, 154.5, 145.3, 141.3, 139.9, 137.5, 132.2, 128.6, 127.8, 127.8, 127.1, 122.0, 118.2, 115.4, 113.3, 111.4, 109.8, 109.4, 84.9, 79.2, 71.6, 56.8, 56.7, 21.7.
IR (Diamond-ATR, neat) $\tilde{\upsilon}_{max}$: 2193 (w), 1790 (m), 1623 (w), 1473 (s), 1374 (vs), 1223 (s), 909 (m), 712 (w) cm^{-1}.
HRMS (ESI) calc. for $C_{27}H_{25}BrIO_4$ $[M+H]^+$: 618.9975 found: 618.9970.

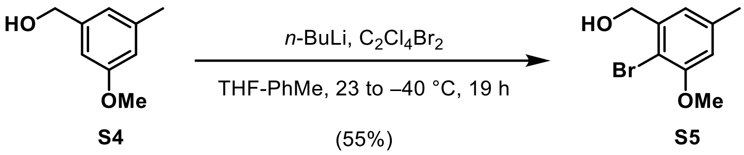

(2-Bromo-3-methoxy-5-methylphenyl)methanol (S5)

To a solution of benzyl alcohol **S4**[3] (1.14 g, 7.49 mmol, 1 equiv) in toluene (74.9 mL, 0.1 M) *n*-butyllithium (2.40 M in *n*-hexane, 6.71 mL, 16.1 mmol, 2.15 equiv) was added dropwise over ten minutes at –40 °C. After stirring for four hours at 23 °C, the reaction was cooled to –40 °C before tetrahydrofuran (15 mL, 0.5 M) was added. To this mixture a solution of 1,2-dibromo-1,1,2,2-tetrachloroethane (4.15 g, 12.7 mmol, 1.70 equiv) in tetrahydrofuran (2 mL, 6.35 M) was added dropwise and stirred for 15 hours at 23 °C. A saturated solution of ammonium chloride (120 mL) was added and the mixture was extracted with diethyl ether (3 × 200 mL). The combined organic layers were washed with a saturated aqueous solution of sodium chloride (100 mL) and the washed solution was dried over magnesium sulfate. The dried solution was filtered, and the filtrate was evaporated under reduced pressure. Purification by silica gel chromatography (20% diethyl ether in pentane) gave the title compound **S5** (945 mg, 4.09 mmol, 55%) as a white solid.

TLC (50% diethyl ether in pentane): R_f = 0.38 (UV, CAM)

mp: 99–101 °C.

^{1}H NMR (400 MHz, CDCl3) δ 6.92 (d, *J* = 2.0 Hz, 1H), 6.68 (d, *J* = 2.1 Hz, 1H), 4.73 (d, *J* = 6.5 Hz, 2H), 3.89 (s, 3H), 2.34 (s, 3H), 2.05 – 1.96 (m, 1H).

^{13}C NMR (101 MHz, $CDCl_3$) δ 155.7, 140.9, 138.5, 121.7, 112.1, 108.5, 65.3, 56.4, 21.5.

IR (Diamond-ATR, neat) $\tilde{\upsilon}_{max}$: 3255 (s), 2899 (w), 1603 (m), 1302 (vs), 1031 (m), 842 (w) cm^{-1}.

HRMS (ESI) calc. for $C_9H_{11}NaBrO_2$ [M+Na]$^+$: 252.9835 found: 252.9833.

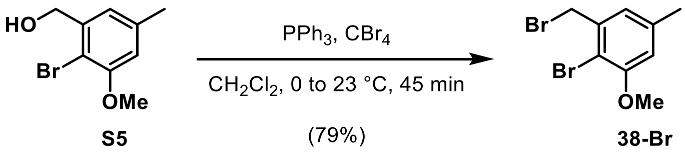

2-Bromo-1-(bromomethyl)-3-methoxy-5-methylbenzene (38-Br)

Alcohol **S5** (0.786 g , 3.40 mmol, 1 equiv) and tetrabromomethane (2.26 g, 6.802 mmol, 2.00 equiv) were dissolved in dichloromethane (34.0 mL, 0.1 M) and cooled to 0 °C. Triphenylphosphine (1.78 g, 6.80 mmol, 2.00 equiv) was added and the reaction was stirred for 45 min while slowly warming to 23 °C. The solvent was removed under reduced pressure and the crude product was purified by silica gel chromatography (5% diethyl ether in pentane) to give benzyl bromide **38-Br** (792 mg, 2.69 mmol, 79%) as a white solid.

NOTE: Addition of triphenylphosphine prior to tetrabromomethane led to significant dimerization of ***S5*** *forming the corresponding dibenzylether (not shown).*

TLC (66% diethyl ether in pentane) R_f = 0.76 (UV, CAM).

mp: 81–82 °C.

^{1}H NMR (400 MHz, CDCl3) δ 6.90 (d, J = 2.0 Hz, 1H), 6.66 (d, J = 2.1 Hz, 1H), 4.60 (s, 2H), 3.89 (s, 3H), 2.32 (s, 3H).

^{13}C NMR (101 MHz, CDCl3) δ 156.1, 141.3, 139.0, 122.1, 112.5, 108.9, 65.8, 56.8, 21.9.

IR (Diamond-ATR, neat) $\tilde{\upsilon}_{max}$: 1565 (w), 1349 (s), 1261 (w), 1004 (s), 957 (m), 714 (w) cm^{-1}.

HRMS (ESI) calc. for $C_9H_{11}Br_2O$ $[M+H]^+$: 292.9171 found: 292.9178.

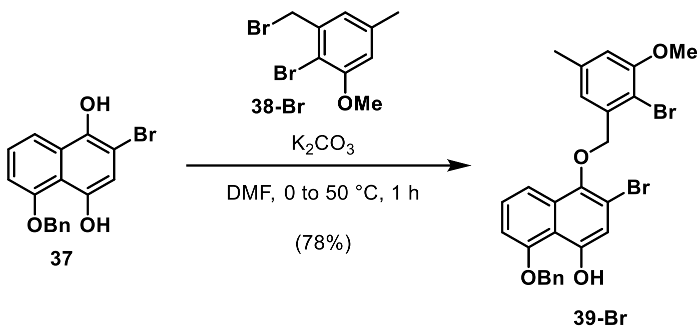

8-(Benzyloxy)-3-bromo-4-((2-bromo-3-methoxy-5-methylbenzyl)oxy)naphthalen-1-ol (39-Br)

5-(Benzyloxy)-2-bromonaphthalene-1,4-diol **37** (194 mg, 561 µmol, 1.1 equiv) was dissolved in degassed *N,N*-dimethylformamide (350 µL, 1.60 M) and cooled to 0 °C. Potassium carbonate (81 mg, 587 µmol, 1.15 equiv) was added and the mixture was stirred for five minutes at 0 °C. A solution of 1-(bromomethyl)-2-bromo-3-methoxy-5-methylbenzene **38-Br** (150 mg, 510 µmol, 1 equiv) in degassed *N,N*-dimethylformamide (350 µL, 1.46 M) was added dropwise to the reaction and rinsed with additional *N,N*-dimethylformamide (300 µL, 1.70 M). The mixture was heated to 50 °C and stirred for one hour. The mixture was allowed to cool to 23 °C and diluted with a solution of lithium chloride (4 M, 5 mL) and then extracted with ethyl acetate (3 × 30). The combined organic layers were washed with a saturated aqueous solution of sodium chloride (40 mL) and the washed solution was dried over sodium sulfate. The dried solution was filtered and the filtrate was evaporated under reduced pressure to give the crude product. Purification was performed by silica gel chromatography (33 % dichloromethane in cyclohexane) to afford quinone **39-Br** (223 mg, 400 µmol, 78%) as a yellow foam, accompanied by recovered benzyl bromide **38-Br** (21.0 mg, 71.4 µmol, 14%).

TLC (33 % dichloromethane in cyclohexane) R_f = 0.17 (UV, CAM).

^{1}H NMR (400 MHz, CDCl3) δ 9.29 (s, 1H), 7.75 (dd, J = 8.6, 0.9 Hz, 1H), 7.51 – 7.40 (m, 5H), 7.39 – 7.32 (m, 2H), 7.02 (s, 1H), 6.93 (dd, J = 7.8, 0.9 Hz, 1H), 6.74 (d, J = 1.9 Hz, 1H), 5.28 (s, 2H), 5.09 (s, 2H), 3.92 (s, 3H), 2.42 (s, 3H).

^{13}C NMR (101 MHz, CDCl3) δ 155.7, 155.7, 151.6, 144.1, 138.6, 137.9, 135.0, 131.3, 129.2, 129.1, 128.1, 127.1, 121.9, 116.4, 115.2, 114.7, 113.8, 112.3, 108.2, 106.3, 74.7, 72.0, 56.5, 21.8.

IR (Diamond-ATR, neat) $\tilde{\upsilon}_{max}$: 3442 (m), 3381 (w), 1733 (m), 1603 (m), 1478 (s), 1392 (s), 1120 (s), 913 (w) cm^{-1}.

HRMS (ESI) calc. for $C_{26}H_{21}Br_2O_4$ [M-H]$^-$: 554.9812 found: 554.9817.

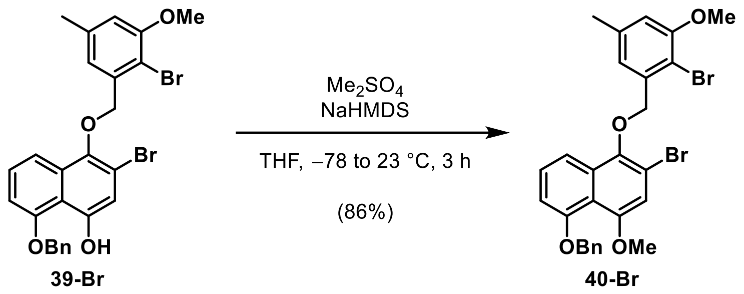

5-(Benzyloxy)-2-bromo-1-((2-bromo-3-methoxy-5-methylbenzyl)oxy)-4-methoxynaphthalene (40-Br)
Sodium bi(trimethylsilyl)amide (1 M in tetrahydrofuran, 200 µL, 200 µmol, 1.05 equiv) was added to a solution of naphthol **39-Br** (106 mg, 190 µmol, 1 equiv) in tetrahydrofuran (571 µL, 0.40 M) at –78 °C followed by dimethyl sulfate (20 µL, 211 µmol, 1.11 equiv). The reaction was allowed to warm to 23 °C and stirred for three hours at that temperature. (*Note: After one hour, 1.50 mL tetrahydrofuran were added to prevent gel-formation.*) A saturated aqueous solution of sodium hydrogen carbonate (10 mL) was added and the solution was extracted with ethyl acetate (4 × 15 mL). The combined organic layers were washed with a saturated aqueous solution of sodium chloride (15 mL) and the washed solution was dried over sodium sulfate. The dried solution was filtered and the filtrate was evaporated under reduced pressure to give the crude product. Purification was performed by silica gel chromatography (50% grading to 70% dichloromethane in cyclohexane) to afford quinone **40-Br** (93 mg, 163 µmol, 86%) as a white solid.

TLC (50 % dichloromethane in cyclohexane) R_f = 0.44 (UV, CAM).

mp: 84–86 °C.

^{1}H NMR (300 MHz, $CDCl_3$) δ 7.77 (dd, *J* = 8.4, 1.0 Hz, 1H), 7.64 – 7.55 (m, 2H), 7.48 – 7.31 (m, 5H), 6.99 (d, *J* = 8.0 Hz, 2H), 6.76 (d, *J* = 1.9 Hz, 1H), 5.21 (s, 2H), 5.14 (s, 2H), 3.95 (s, 3H), 3.93 (s, 3H), 2.43 (s, 3H).

^{13}C NMR (101 MHz, chloroform-*d*) δ 156.7, 155.7, 154.4, 145.4, 138.6, 138.1, 137.5, 132.2, 128.6, 127.8, 127.8, 127.1, 121.9, 118.2, 115.3, 113.3, 112.3, 109.8, 109.4, 108.2, 74.6, 71.6, 56.8, 56.6, 21.8.

IR (Diamond-ATR, neat) $\tilde{\upsilon}_{max}$: 2223 (m), 1797 (m), 1601 (m), 1470 (vs), 1424 (s), 1221 (s), 937 (m), 710 (w) cm^{-1}.

HRMS (ESI) calc. for $C_{27}H_{25}Br_2O_4$ [M+H]$^+$: 571.0114 found: 571.0116.

3.2 Intramolecular Biaryl Coupling

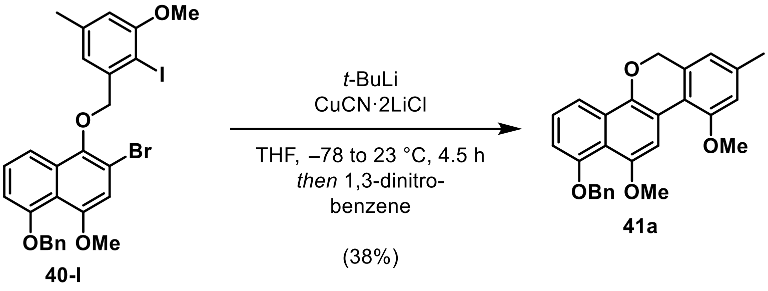

1-(Benzyloxy)-10,12-dimethoxy-8-methyl-6H-dibenzo[*c*,*h*]chromene (41a)

Tert-butyllithium (1.48 M in pentane, 630 µL, 930 µmol, 4.00 equiv) was added dropwise to a solution of ether **40-I** (144 mg, 232.5 µmol, 1 equiv) in tetrahydrofuran (2.33 mL, 0.1 M) at –78 °C and stirred for one hour at that temperature. A freshly prepared solution of CuCN·2LiCl (41.0 mg, 232.5 µmol, 1 equiv) in tetrahydrofuran (233 µL, 1 M) was added dropwise to the reaction and stirred for two hours while warming to –40 °C. A solution of 1,3-dinitrobenzene (156 mg, 930 µmol, 4.00 equiv) in tetrahydrofuran (930 µL, 1 M) was added and the reaction was stirred for 1.5 hours while warming to 10 °C. The reaction was stopped by the addition of methanolic hydrochloric acid (5 mL) and stirred for 30 minutes, before a saturated aqueous solution of ammonium chloride (5 mL) was added. The mixture was extracted with diethyl ether (3 × 20 mL). The combined organic layers were washed with a saturated aqueous solution of sodium chloride (20 mL) and the washed solution was dried over sodium sulfate. The dried solution was filtered and the filtrate was evaporated under reduced pressure to give the crude product. The remaining crude mixture was purified by two consecutive applications of silica gel chromatography (A: 10% dichloromethane in cyclohexane; B: 3% diethyl ether in pentane) to afford dibenzochromene **41a** (36.4 mg, 88.4 µmol, 38%) as a white solid.

TLC (57% dichloromethane in cyclohexane): R_f = 0.37 (UV, CAM).
mp: 167–169 °C.
^{1}H NMR (400 MHz, chloroform-*d*) δ 8.00 (s, 1H), 7.91 (dd, *J* = 8.5, 1.1 Hz, 1H), 7.66 – 7.59 (m, 2H), 7.44 – 7.40 (m, 2H), 7.35 (dd, *J* = 8.3, 7.8 Hz, 1H), 7.35 – 7.31 (m, 1H), 6.95 (dd, *J* = 7.8, 1.1 Hz, 1H), 6.79 (s, 1H), 6.69 (s, 1H), 5.22 (s, 2H), 5.09 (s, 2H), 3.96 (s, 3H), 3.95 (s, 3H), 2.40 (s, 3H).
^{13}C NMR (101 MHz, chloroform-*d*) δ 156.4, 155.8, 150.8, 144.8, 139.0, 137.9, 134.6, 128.5, 128.3, 127.6, 127.2, 126.0, 118.3, 118.2, 117.7, 116.7, 115.6, 112.9, 109.7, 107.8, 71.8, 69.5, 57.5, 56.0, 21.7.
IR (Diamond-ATR, neat) $\tilde{\upsilon}_{max}$: 2104 (w), 1590 (m), 1377 (m), 1346 (s), 1286 (s), 1133 (vs), 1051 (s), 843 (m), 756 (w) cm^{-1}.
HRMS (ESI) calc. for $C_{27}H_{25}O_4$ [M+H]$^+$: 413.1747 found: 413.1746.

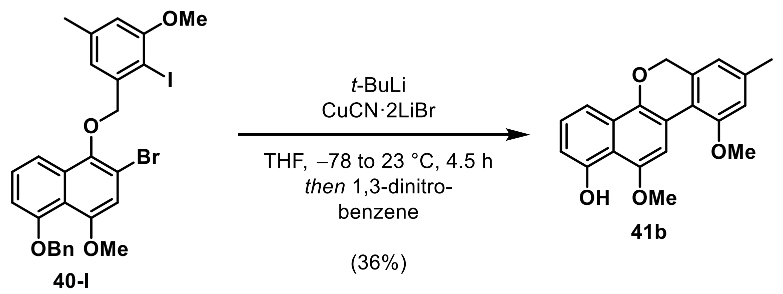

10,12-Dimethoxy-8-methyl-6*H*-dibenzo[*c*,*h*]chromen-1-ol (41b)

Tert-butyllithium (1 M in pentane, 9.70 mL, 14.7 mmol, 11.0 equiv) was added dropwise to a solution of ether **40-I** (825 mg, 1.33 mmol, 1 equiv) in tetrahydrofuran (13.3 mL, 0.1 M) at –78 °C and stirred for 30 minutes. A freshly prepared solution of CuCN·2LiBr (351 mg, 1.33 mmol, 1.00 equiv) in tetrahydrofuran (3.51 mL, 0.38 M) was added dropwise to the reaction and stirred for two hours while warming to –40 °C. A solution of 1,3-dinitrobenzene (896 mg, 5.33 mmol, 4.00 equiv) in tetrahydrofuran (8.96 mL, 0.59 M) was added and the reaction was warmed to 23 °C over a period of 30 minutes and stirred for two hours at that temperature. The reaction was stopped by the addition of methanolic hydrochloric acid (15 mL) and stirred for 30 minutes, before a saturated aqueous solution of ammonium chloride (15 mL) was added. The mixture was extracted with diethyl ether (3 × 60 mL). The combined organic layers were washed with a saturated aqueous solution of sodium chloride (50 mL) and the washed solution was dried over sodium sulfate. The dried solution was filtered and the filtrate was evaporated under reduced pressure to give the crude product. Residual dinitrobenzene was largely removed by sublimation at 0.1 mbar and 40 °C for 20 hours. The remaining crude mixture was purified by two consecutive applications of silica gel chromatography (A: 25% dichloromethane in cyclohexane; B: 5% diethyl ether in pentane) to afford dibenzochromene **41b** (155 mg, 0.480 mmol, 36%) as a yellow solid.

TLC (50% dichloromethane in cyclohexane): R_f = 0.26 (CAM).

mp: 174 °C.

^{1}H NMR (400 MHz, chloroform-*d*) δ 9.44 (s, 1H), 7.91 (s, 1H), 7.73 (dd, *J* = 8.4, 1.1 Hz, 1H), 7.35 (dd, *J* = 8.4, 7.6 Hz, 1H), 6.88 (dd, *J* = 7.7, 1.1 Hz, 1H), 6.79 (s, 1H), 6.69 (dt, *J* = 1.7, 0.8 Hz, 1H), 5.08 (s, 2H), 4.09 (s, 3H), 3.95 (s, 3H), 2.40 (s, 3H).

^{13}C NMR (101 MHz, chloroform-*d*) δ 156.2, 154.4, 149.9, 145.4, 139.1, 134.4, 127.8, 127.4, 118.4, 116.8, 116.5, 114.8, 113.4, 112.8, 111.1, 104.3, 69.4, 56.4, 56.0, 21.7.

IR (Diamond-ATR, neat) $\tilde{\nu}_{max}$: 3553 (m), 3092 (m), 2004 (m), 1741 (w), 1652 (w), 1223 (s), 1103 (vs), 1030 (m), 972 (s) cm^{-1}.

HRMS (ESI) calc. for $C_{20}H_{17}O_4$ [M-H]$^-$: 321.1132 found: 321.1137.

Optimization Studies for the anticipated Stille–Kelly coupling

All solids (dihalogen **40**, palladium catalyst and additives) were placed into a 20-mL-pressure tube in a glove box. All liquids (solvent and stannane) were added outside of the glove box via canula under an argon counterflow. The pressure tube was sealed with a Teflon® screw-cap and then put into a pre-heated oil bath under light exclusion. After the given reaction time, the oil bath was removed to allow the reaction to cool to 23 °C.

For reactions using DMF or NMP as solvent (Entries 7, 20, 28 and 29): The mixture was diluted with a solution of lithium chloride (4 M, 5 mL) and then extracted with ethyl acetate (3 × 10). The combined organic layers were washed with a saturated aqueous solution of sodium chloride (10 mL) and the washed solution was dried over magnesium sulfate. The dried solution was filtered and the filtrate was evaporated under reduced pressure to give the crude product.

For reactions using 1,4-dioxane, acetonitrile (MeCN), toluene (PhMe) or tetrahydrofuran (THF) (Entries 1–6, 8–19, 21–27 and 30–41: The suspension was filtered through a short plug of Celite® and the solvent was removed. The crude residue was taken up with $CDCl_3$ for a ^1H NMR measurement. For Entries 1, 19, 39, 40 and 41: Then, the crude product was purified via flash column chromatography on silica gel (50% dichloromethane in cyclohexane) to afford product **41a** as a white solid.

Table 1: Screening conditions for the Stille–Kelly coupling

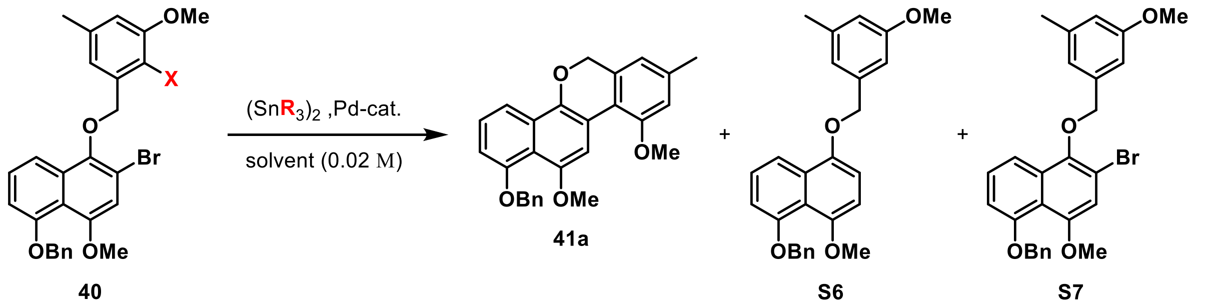

Entry[a]	X	R (eq.)	Pd-cat. (eq.)	Additive (eq.)	Solvent[b]	Temp.	Time	41a[c]	Result[d] 41a	40	S6	S7	Z[e]
1	I	Bu (1.1)	$Pd(PPh_3)_2Cl_2$ (0.1)	-	1,4-dioxane	140 °C	23 h	31%	**1.7**	0	0.8	1	0.2
2	Br	Bu (1.1)	$Pd(PPh_3)_2Cl_2$ (0.1)	-	1,4-dioxane	140 °C	23 h	n.i.	**1**	0	**1**	0	0
3	I	Me (1.1)	$Pd(PPh_3)_2Cl_2$ (0.1)	-	1,4-dioxane	140 °C	16 h	n.i.	complex mixture				
4	Br	Me (1.1)	$Pd(PPh_3)_2Cl_2$ (0.1)	-	1,4-dioxane	140 °C	16 h	n.i.	complex mixture				
5	I	Bu (1.1)	$Pd(PPh_3)_2Cl_2$ (0.1)	-	1,4-dioxane	170 °C	2 h	n.i.	0.5	**3**	0	1	0.7
6	I	Bu (1.1)	$Pd(PPh_3)_2Cl_2$ (0.1)	CuI (0.4)	1,4-dioxane	130 °C	12 h	n.i.	0.1	0	0	**1**	0.1
7	I	Bu (1.1)	$Pd(PPh_3)_2Cl_2$ (0.1)	-	DMF	130 °C	12 h	n.i.	0.1	0	0	**1**	0.1
8	I	Bu (1.1)	$Pd(PPh_3)_2Cl_2$ (0.1)	-	PhMe	130 °C	12 h	n.i.	**1**	0	0.6	**1**	1
9	I	Bu (2.5)	$Pd(PPh_3)_2Cl_2$ (0.1)	-	1,4-dioxane	130 °C	12 h	n.i.	0.6	0	0.1	1	**1.3**
10	I	Bu (1.1)	$Pd(PPh_3)_4$ (0.1)	-	1,4-dioxane	130 °C	12 h	n.i.	**2**	0	0.5	1	**1.5**
11	I	Bu (1.1)	$Pd(P\textit{t}\text{-}Bu_3)_2$ (0.1)	-	1,4-dioxane	130 °C	12 h	n.i.	**0.3**	0	**0.3**	1	0.2
12	I	Bu (2.0)	$Pd(dppe)_2$ (0.1)	-	1,4-dioxane	130 °C	12 h	n.i.	0.1	**2**	0	1	0.8
13	I	Bu (0.5)	$Pd(PPh_3)_2Cl_2$ (0.1)	-	1,4-dioxane	140 °C	16 h	n.i.	0.3	**0.8**	0.1	1	0.1
14	I	Bu (1.1)	$Pd(PPh_3)_2Cl_2$ (0.1)	LiCl (3.2)	1,4-dioxane	140 °C	16 h	n.i.	0.7	0	0.2	**1**	0.4
15	I	Bu (1.1)	$Pd(PPh_3)_2Cl_2$ (0.1)	CsF (2.2)	1,4-dioxane	140 °C	16 h	n.i.	0.2	0.1	0.2	**1**	0.5
16	I	Bu (1.1)	$Pd(PPh_3)_2Cl_2$ (0.1)	-	MeCN	140 °C	16 h	n.i.	**1**	0	0.2	**1**	0.1
17	I	Bu (1.1)	$Pd(dppf)_2Cl_2$ (0.1)	-	1,4-dioxane	140 °C	16 h	n.i.	**0.9**	0	**1**	**1**	0
18	I	Bu (1.1)	$Pd_2(dba)_3$, $AsPh_3$[f] (0.1)		1,4-dioxane	140 °C	16 h	n.i.	0.4	**1.3**	0	**1**	**1.1**
19	Br	Bu (1.1)	$Pd(PPh_3)_4$ (0.1)	-	1,4-dioxane	140 °C	16 h	44%	**1**	0	0.7	0	0
20	Br	Bu (1.1)	$Pd(PPh_3)_2Cl_2$ (0.1)	-	DMF	140 °C	16 h	n.i.	complex mixture				
21	Br	Bu (1.1)	$Pd(PPh_3)_4$ (0.1)	LiCl (3.2)	1,4-dioxane	140 °C	16 h	n.i.	**1**	0	**1**	0	0
22	Br	Bu (1.1)	$Pd(PPh_3)_4$ (0.1)	CsF (2.2)	1,4-dioxane	140 °C	16 h	n.i.	**1**	0.1	0.6	0	0
23	Br	Bu (1.1)	$Pd(PPh_3)_4$ (0.1)	CuI (1.5)	1,4-dioxane	140 °C	16 h	n.i.	mainly **40** (+ minor unidentified SP)				
24	Br	Bu (1.1)	$Pd(PPh_3)_4$ (0.1)	-	PhMe	140 °C	16 h	n.i.	0	**1**	0.2	0	0.2
25	Br	Bu (1.1)	$Pd(PPh_3)_4$ (0.1)	NBu_4I (1.4) Li_2CO_3 (1.4)	PhMe	140 °C	16 h	n.i.	complex mixture (including **41a**, **40**, **S6** and **S7**)				
26	Br	Bu (1.1)	$Pd(PPh_3)_4$ (0.1)	-	MeCN	140 °C	16 h	n.i.	0	**1**	0.1	0	0
27	Br	Bu (1.1)	$Pd(PPh_3)_4$ (0.1)	CuI (1.5)	MeCN	140 °C	16 h	n.i.	mainly **40** (+ minor unidentified SP)				
28	Br	Bu (1.1)	$Pd(PPh_3)_4$ (0.1)	-	NMP	140 °C	16 h	n.i.	0	**1**	**0.8**	0	0.4
29	Br	Bu (1.1)	$Pd(PPh_3)_4$ (0.1)	CuI (1.5)	NMP	140 °C	16 h	n.i.	mainly **40** (+ minor unidentified SP)				
30	Br	Bu (1.1)	$Pd(PPh_3)_4$ (0.1)	-	THF	140 °C	16 h	n.i.	0	**1**	0.2	0	0
31	Br	Bu (1.1)	$Pd(PPh_3)_4$ (0.1)	-	MeCN	80 °C	16 h	n.i.	mainly **40** (+ minor **41a** and **S7**)				
32	Br	Bu (1.1)	$Pd(PPh_3)_4$ (0.1)	-	MeCN	100 °C	16 h	n.i.	complex mixture (including **41a**, **40**, **S6** and **S7**)				
33	Br	Bu (1.1)	$Pd(PPh_3)_4$ (0.1)	-	MeCN	120 °C	16 h	n.i.	**2.4**	1	0	**1.8**	0
34	Br	Bu (1.1)	$Pd(PPh_3)_4$ (0.1)	-	THF	100 °C	16 h	n.i.	**6.2**	1	0	**1.9**	1.3
35	Br	Bu (1.1)	$Pd(PPh_3)_4$ (0.1)	-	THF	120 °C	16 h	n.i.	**10**	1	0	0.2	**2.6**
36	Br	Me (1.1)	$Pd(PPh_3)_4$ (0.17)	-	MeCN	140 °C	13 h	n.i.	mainly P (+ several minor unidentif. SP)				
37	Br	Me (1.1)	$Pd(PPh_3)_4$ (0.17)	-	THF	140 °C	13 h	n.i.	0.4	1	0.8	0	**1.9**
38	Br	Bu (1.1)	$Pd(PPh_3)_4$ (0.17)	-	MeCN[g]	140 °C	13 h	n.i.	mainly **41a** + partial decomposition				
39[h]	Br	Bu (1.1)	$Pd(PPh_3)_4$ (0.3)	-	MeCN	140 °C	14 h	61%	mainly **41a** + partial decomposition				
40[h]	Br	Bu (1.1)	$Pd(PPh_3)_4$ (0.4)	-	MeCN	140 °C	14 h	74%	mainly **41a** + partial decomposition				
41[i]	Br	Bu (1.1)	$Pd(PPh_3)_4$ (0.4)	-	MeCN	140 °C	14 h	63%	mainly **41a** + partial decomposition				

[a] reactions have been performed on a 23.6 µmol scale, [b] degassed, [c] isolated yield (n.i. = no isolation was performed), [d] crude-^{1}H-NMR: ratios of integrals of the main-signals (bold numbers highlight the most prominent signals), [e] unidentified side-product, [f] 0.4 equiv. of $AsPh_3$, [g] 0.04 M, [h] Scale: 87.4 µmol, [i] Scale: 175 µmol. (SP = side-product).

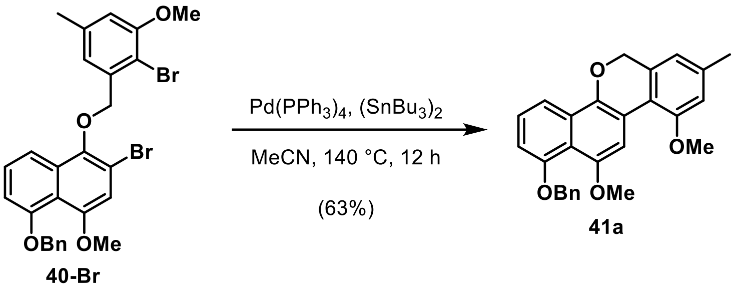

1-(benzyloxy)-10,12-dimethoxy-8-methyl-6H-dibenzo[*c*,*h*]chromene (41a)

Dibromide **40-Br** (100 mg, 175 µmol, 1 equiv) and tetrakis(triphenylphosphine)palladium(0) (80.8 mg, 69.9 µmol, 0.40 equiv) were placed in a 20-mL-pressure tube inside a glovebox before bis(tributyltin) (94 µL, 187 µmol, 1.07 equiv) and degassed acetonitrile (8.74 mL, 0.02 M) were added to the pressure tube via syringe under a argon counterflow outside of the glove box. The pressure tube was sealed, covered with aluminum foil and heated to 140 °C for twelve hours under constant stirring. After cooling to 23 °C the solvent of the reaction mixture was removed under reduced pressure and the crude product was purified by flash column chromatography on silica gel (50 % cyclohexane in dichloromethane) to yield tetracycle **41a** (45 mg, 63 %) as a white solid.

For analytical data of compound **41a**: *see above.*

3.3 Mild benzylic Oxidation of a quinone-based Chromene using DDQ and THP

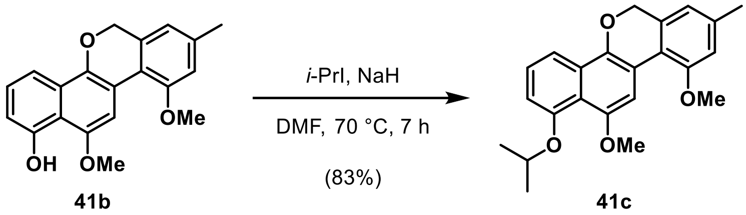

1-Isopropoxy-10,12-dimethoxy-8-methyl-6*H*-dibenzo[*c*,*h*]chromene (41c)

Sodium hydride (60% dispersion in mineral oil, 5.04 mg, 129 µmol, 1.30 equiv) was added to a solution of naphtol **41b** (32.0 mg, 99.3 µmol, 1 equiv) in *N*,*N*-dimethylformamide (764 µL, 0.13 M) at 0 ˚C. After 30 minutes, 2-iodopropane (20 µL, 200 µmol, 2.00 equiv) was added in one portion to the mixture and then stirred for seven hours at 70 °C. The reaction was allowed to cool to 23 ˚C before water (2 mL) was added and then extracted with dichloromethane (3 × 10 mL). The combined organic layers were washed with a saturated aqueous solution of sodium chloride (10 mL) and the washed solution was dried over magnesium sulfate. The dried solution was filtered and the filtrate was evaporated under reduced pressure. Purification by silica gel chromatography (5% ethyl acetate in cyclohexane) gave the title compound **41c** (30.0 mg, 82.4 µmol, 83%) as a white solid.

TLC (10% ethyl acetate in cyclohexane): R_f = 0.34 (UV, CAM, $KMnO_4$).

mp: 179 °C.

^{1}H NMR (400 MHz, chloroform-*d*) δ 7.96 (s, 1H), 7.91 (dd, *J* = 8.4, 1.1 Hz, 1H), 7.34 (t, *J* = 8.0 Hz, 1H), 6.95 (dd, *J* = 7.6, 1.2 Hz, 1H), 6.78 (s, 1H), 6.68 (s, 1H), 5.08 (s, 2H), 4.56 (hept, *J* = 5.8 Hz, 1H), 3.96 (s, 3H), 3.94 (s, 3H), 2.39 (s, 3H), 1.41 (d, *J* = 6.0 Hz, 6H).

^{13}C NMR (101 MHz, chloroform-*d*) δ 156.4, 154.5, 150.6, 145.1, 138.9, 134.5, 128.5, 125.8, 119.8, 118.3, 117.4, 116.8, 115.9, 114.1, 112.8, 108.6, 73.2, 69.5, 57.9, 56.0, 22.2, 21.7.

IR (Diamond-ATR, neat) $\tilde{\upsilon}_{max}$: 3031 (m), 2970 (m), 1534 (m), 1302 (m), 1274 (m), 1249 (w) cm^{-1}.

HRMS (ESI) calc. for $C_{23}H_{25}O_4$ $[M+H]^+$: 365.1747 found: 365.1742.

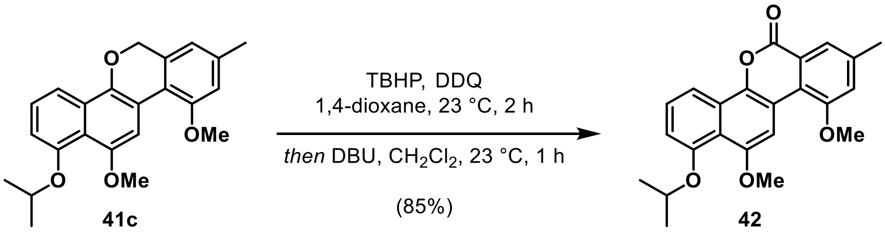

1-Isopropoxy-10,12-dimethoxy-8-methyl-6*H*-dibenzo[*c*,*h*]chromen-6-one (42)

Tert-butyl hydroperoxide (5.5 M in nonane, 15.0 µL, 82.4 µmol, 2.00 equiv) followed by 2,3-dichloro-5,6-dicyano-1,4-benzoquinone (14.0 mg, 61.8 µmol, 1.50 equiv) were added to a solution of tetracycle **41c** (15 mg, 41.2 µmol, 1 equiv) in 1,4-dioxane (458 µL, 0.09 M) at 23 ˚C and stirred for two hours at that temperature. The solvent was removed under reduced pressure and the crude solid was re-dissolved in dichloromethane (458 µL, 0.09 M). 1,8-Diazabicyclo(5.4.0)undec-7-ene (30.7 µL, 206 µmol, 5.00 equiv) was added and the mixture was stirred for one hour at 23 °C. The solvent was removed and the crude mixture was directly subjected to silica gel chromatography (10% cyclohexane in dichloromethane) to afford isopropylated defucogilvocarcin M **42** (13.2 mg, 35.0 µmol, 85%) as a yellowish solid.

Data consistent with literature: *J. Org. Chem.*, **2009**, *74*, 4080–4093.

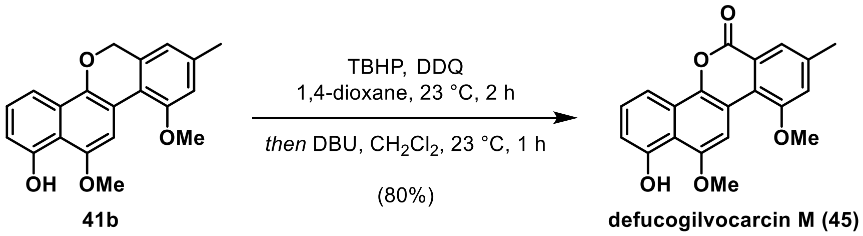

Defucogilvocarcin M (45)

Tert-butyl hydroperoxide (5.5 M in nonane, 56.4 µL, 310 µmol, 2.00 equiv) followed by 2,3-dichloro-5,6-dicyano-1,4-benzoquinone (44.0 mg, 194 µmol, 1.25 equiv) were added to a solution of tetracycle **41b** (50.0 mg, 155 µmol, 1 equiv) in 1,4-dioxane (1.72 mL, 0.09 M) at 23 ˚C and stirred for two hours at that temperature. The solvent was removed under reduced pressure and the crude solid was re-dissolved in dichloromethane (3.10 mL, 0.09 M). 1,8-Diazabicyclo(5.4.0)undec-7-ene (69.5 µL, 465 µmol, 3.00 equiv) was added and the mixture was stirred for one hour at 23 °C. The solvent was

removed under reduced pressure and the crude mixture was directly subjected to silica gel chromatography (60% diethyl ether in cyclohexane) to afford defucogilvocarcin M **45** (41.7 mg, 124.1 µmol, 80%) as a white solid.

TLC (25% diethyl ether in pentane): R_f = 0.17 (UV, $KMnO_4$).
HRMS (ESI) calc. for $C_{20}H_{17}O_5$ $[M+H]^+$: 337.1071 found: 337.1066.

Comparison with the reported spectral Data of Defucogilvocarcin M in $CDCl_3$:

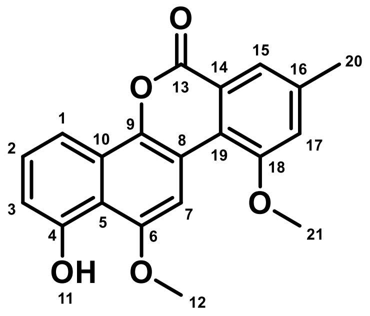

defucogilvocarcin M (45)

Atom Number	This work (400 MHz, 101 MHz)		Suzuki's work[1] (–)[2]	
	^{1}H	^{13}C	^{1}H	^{13}C
1	8.04 (dd, *J* = 8.4, 1.1 Hz, 1H)	113.5	8.05 (dd, *J* = 7.6, 0.7 Hz, 1H)	113.4
2	7.48 (t, *J* = 8.1 Hz, 1H)	128.5	7.48 (dd, *J* = 7.6, 7.6 Hz, 1H)	128.4
3	6.99 (dd, *J* = 7.7, 1.1 Hz, 1H)	112.5	6.99 (dd, *J* = 7.6, 0.7 Hz, 1H)	112.4
4		154.3		154.2
5		113.1		112.9
6		151.9		151.8
7	8.23 (s, 1H)	101.8	8.23 (s, 1H)	101.8
8		114.7		114.6
9		141.3		141.3
10		126.3		126.2
11	9.33 (s, 1H)		9.34 (s, 1H)	
12	4.07 (s, 3H)	56.3	4.12 (s, 3H)	56.2
13		161.4		161.2
14		123.1		123.0
15	7.88 (dd, *J* = 1.7, 0.9 Hz, 1H)	122.8	7.89 (s, 1H)	122.8
16		140.0		139.9
17	7.06 (d, *J* = 1.7 Hz, 1H)	118.1	7.07 (s, 1H)	118.1
18		157.0		157.0
19		121.8		121.8
20	2.47 (s, 3H)	21.8	2.48 (s, 3H)	21.6
21	4.03 (s, 3H)	56.1	4.07 (s, 3H)	56.0

[1]I. Takemura, K. Imura, T. Matsumoto, K. Suzuki. Concise Three-Component Synthesis of Defucogilvocarcin M. *Org. Lett.*, **2004**, *6*, 2503–2505.
[2] frequency was not mentioned by the authors.

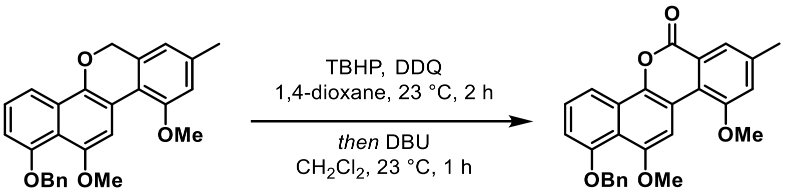

41a 44

1-(Benzyloxy)-10,12-dimethoxy-8-methyl-6*H*-dibenzo[*c*,*h*]chromen-6-one (44)

Cyclic ether **41a** (1.02 g, 2.46 mmol, 1 equiv) was dissolved in 1,4-dioxane (27.4 mL, 0.09 M). 2,3-dichloro-5,6-dicyano-*p*-benzoquinone (839 mg, 3.70 mmol, 1.50 equiv) was added to the solution, followed by *tert*-butyl hydroperoxide (5.5 M in nonane, 896 µL, 2.93 mmol, 2.00 equiv) and stirred for two hours at 23 °C. The solvent was removed under reduced pressure and the residual crude solid was dissolved in dichloromethane (49.3, 0.05 M). 1,8-Diazabicyclo(5.4.0)undec-7-ene (1.47 mL, 9.84 mmol, 4.00 equiv) was added and the mixture was stirred for one hour at 23 °C. The solvent was removed and the crude mixture was directly subjected to silica gel chromatography (10% grading to 1% cyclohexane in dichloromethane) to afford aglycone **44** (807 mg, 1.89 mmol, 77%) as a white solid.

TLC (25% ethyl acetate in cyclohexane): R_f = 0.27 (UV, CAM).

mp: 221 °C.

^{1}H NMR (400 MHz, chloroform-*d*) δ 8.36 (s, 1H), 8.22 (dd, *J* = 8.5, 1.0 Hz, 1H), 7.92 (s, 1H), 7.64 – 7.59 (m, 2H), 7.50 (t, *J* = 8.1 Hz, 1H), 7.43 (dd, *J* = 8.5, 7.8 Hz, 2H), 7.38 – 7.32 (m, 1H), 7.11 (s, 1H), 7.04 (dd, *J* = 7.8, 1.0 Hz, 1H), 5.22 (s, 2H), 4.06 (s, 3H), 3.99 (s, 3H), 2.48 (s, 3H).

^{13}C NMR (101 MHz, $CDCl_3$) δ 161.6, 157.3, 155.8, 153.0, 140.6, 139.9, 137.6, 128.5, 127.7, 127.2, 127.1, 126.9, 123.3, 122.9, 122.1, 118.2, 118.1, 115.4, 113.9, 110.3, 104.4, 71.6, 56.6, 56.3, 21.8.

IR (Diamond-ATR, neat) $\tilde{\upsilon}_{max}$: 1716 (m), 1592 (s), 1398 (m), 1336 (s), 1297 (vs), 1122 (w), 1062 (m), 852 (m), 746 (w) cm^{-1}.

HRMS (ESI) calc. for $C_{27}H_{23}O_5$ $[M+H]^+$: 427.1540 found: 427.1547.

4. Meta-functionalization of 2-Bromo-5-iodo-1-naphthol

Treatment of 2-bromo-5-iodonaphthol **22-Br** with *N*-chlorosuccinimide (NCS) in acetonitrile affords quantitative conversion to bench-stable enone **47**. The succinimide could be removed by filtration of **47** through a short plug of silica with only little decomposition (less than 10%), while aqueous basic work-up results in partial decomposition accompanied with significant enolization. Interestingly, in a first attempt the use of Nagata's reagent led to nucleophilic attack of mainly the ethyl group (40%) and only minor amounts of the cyanide (25%), while in any other attempt to reproduce this result the cyanide **S8-CN** was isolated as main product (Entry 1). Similarly, $AlEt_3$ underwent conjugate addition in 85% overall (Entry 2), while a one-pot transformation starting from naphthol **22-Br** showed almost quantitative overall yield (Entry 5). The use of $SnCl_4$ allowed for the application of Mukaiyama-Aldol conditions (Entry 3) as well as for the installation of nonactivated anisole (Entry 4). It is noteworthy, that both products have been isolated as a mixture of 2-chloro- and 2-bromonaphthols, indicating a $SnCl_4$-induced halogen shuffle. For all entries, naphthol **22** was isolated as minor side-product (between 3 and 15%). Since several of the known stable phenols and naphthols in their keto-form are decorated with halogens (especially in the *ortho*-position)[4] we conclude that the bromine and the iodine might contribute to the stability of the dienone-form **47** by their electronegativity. However, similar dearomatization reactions are known for less-substituted systems (compare reference 24a).

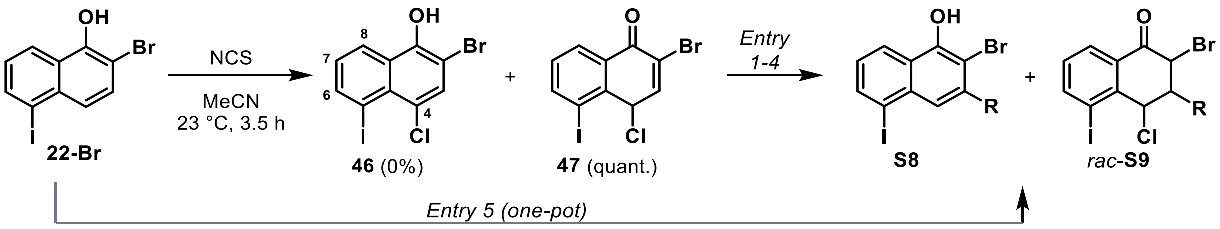

Entry	Nucleophile	Conditions	R	S8	*rac*-S9
1	Et_2AlCN	PhMe, –78 to 23 °C, 2.5 h	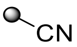	**58%**[a]	**–**[b]
2	Et_3Al	PhMe, –78 °C, 5 min	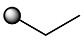	**60%**	**25%**
3	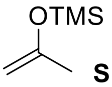 **S10**	$SnCl_4$, CH_2Cl_2, –78 to 23 °C, 8 h	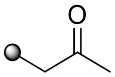	**36%**[c]	**–**
4	PhOMe	$SnCl_4$, CH_2Cl_2, 23 °C, 2 h	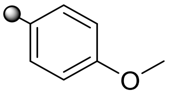	**38%**[d]	**–**
5[e]	Et_3Al	NCS, MeCN, 23 °C, 3 h *then* $AlEt_3$, PhMe, –78 °C, 5 min	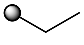	**41%**	**56%**

[a]along with 8% of R = Et. [b]according to the crude ^{1}H-NMR the cyano-tetralone is mainly formed, which decomposes to **S8-CN** during purification over silica. [c]2:1-mix of 2-Br/2-Cl-naphthol. [d]1:1-mix of 2-Br/2-Cl-naphthol. [e]in one-pot from **22-Br**.

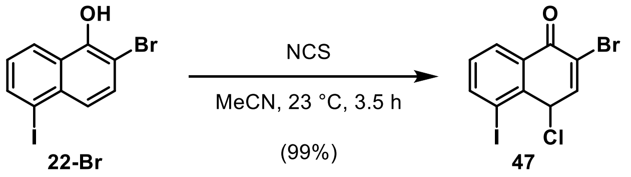

2-Bromo-4-chloro-5-iodonaphthalen-1(4*H*)-one (47)

N-Chlorosuccinimide (66.0 mg, 495 µmol, 1.10 equiv) was added to a solution of naphthol **22-Br** (157 mg, 450 µmol, 1 equiv) in acetonitrile (3.00 mL, 0.15 M) in three portions over 30 minutes at 23 °C. After three hours, the mixture was diluted with a mixture of cyclohexane and ethyl acetate (1:1) and quickly filtered over a short plug of silica to obtain the desired product **47** in approximately 95% purity (171 mg, 446 µmol, 99%).

TLC (14% ethyl acetate in cyclohexane): R_f = 0.38 (UV, CAM, $KMnO_4$).
mp: 75 °C (decomposition).
^{1}H NMR (400 MHz, chloroform-*d*) δ 8.22 (dd, *J* = 7.8, 1.3 Hz, 1H), 8.16 (dd, *J* = 7.8, 1.3 Hz, 1H), 7.62 (d, *J* = 5.3 Hz, 1H), 7.30 – 7.22 (m, 1H), 5.67 (d, *J* = 5.3 Hz, 1H).
^{13}C NMR (101 MHz, chloroform-*d*) δ 177.2, 145.1, 144.0, 141.2, 131.9, 131.2, 128.8, 126.4, 100.5, 56.9.
IR (Diamond-ATR, neat) $\tilde{\upsilon}_{max}$: 3093 (w), 2949 (m), 1732 (m), 1613 (m), 1423 (w), 871 (s), 793 (m), 742 (m) cm^{-1}.
HRMS (ESI) calc. for $C_{10}H_4BrClIO$ $[M-H]^-$: 380.8184 found: 380.8188.

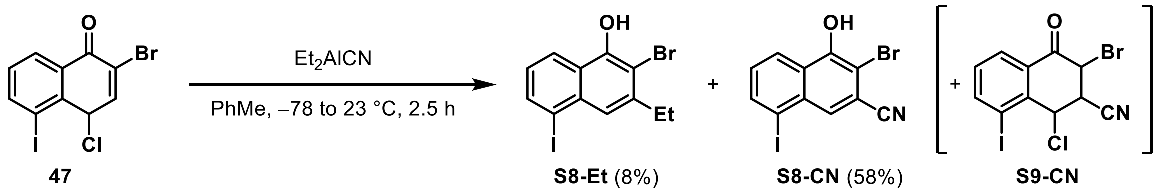

2-Bromo-3-ethyl-5-iodonaphthalen-1-ol (S8-Et)
3-Bromo-4-hydroxy-8-iodo-2-naphthonitrile (S8-CN)

Diethylaluminium cyanide (Nagata's reagent) (1 M in PhMe, 138 µL, 138 µmol, 1.20 equiv) was added dropwise to a solution of enone **47** (44.0 mg, 115 µmol, 1 equiv) in toluene (1.15 mL, 0.1 M) at –78 °C. After two hours of stirring at that temperature, the residual solid dry-ice was removed from the external acetone bath to initiate slow warming to 23 °C within 50 minutes. After stirring for ten minutes at 23 °C, the red solution was diluted with water (3 mL) and extracted with diethyl ether (3 × 5 mL). The combined organic layers were washed with a saturated aqueous solution of sodium chloride (5 mL) and the washed solution was dried over magnesium sulfate. The dried solution was filtered and the filtrate was concentrated under reduced pressure to give a crude mixture containing 9% of **S8-Et** and 63% of presumably **S9-CN** as indicated by internal-standard-^{1}H-NMR. Purification by silica gel chromatography (11% ethyl acetate in cyclohexane + 2% acetic acid) afforded compound **S8-Et**

(3.50 mg, 9.20 µmol, 8%) as a white solid and compound **S8-CN** (24.9 mg, 66.7 µmol, 58%) as a yellow solid.

Compound **S8-Et**

TLC (14% diethyl acetate in cyclohexane): R_f = 0.59 (UV, CAM, $KMnO_4$).

mp: 69 °C.

^{1}H NMR (400 MHz, chloroform-*d*) δ 8.25 (d, *J* = 8.4 Hz, 1H), 8.11 (dd, *J* = 7.4, 1.1 Hz, 1H), 7.57 (s, 1H), 7.15 (dd, *J* = 8.4, 7.3 Hz, 1H), 6.19 (s, 1H), 2.95 (q, *J* = 7.5 Hz, 2H), 1.38 (t, *J* = 7.5 Hz, 3H).

^{13}C NMR (101 MHz, chloroform-*d*) δ 148.3, 142.0, 138.7, 134.0, 126.3, 123.8, 123.5, 123.4, 108.9, 98.5, 30.3, 14.4.

IR (Diamond-ATR, neat) $\tilde{\upsilon}_{max}$: 3104 (w), 1521 (s), 1434 (vs), 1209 (s), 1144 (s), 798 (w) cm^{-1}.

HRMS (ESI) calc. for $C_{12}H_9BrIO$ $[M-H]^-$: 374.8887 found: 374.8891.

Compound **S8-CN**

TLC (14% diethyl acetate in cyclohexane): R_f = 0.17 (UV, CAM, $KMnO_4$).

mp: 70–72 °C.

^{1}H NMR (400 MHz, dichloromethane-*d₂*) δ 8.63 (dd, *J* = 7.4, 1.3 Hz, 1H), 8.50 (dd, *J* = 8.4, 1.3 Hz, 1H), 7.37 (dd, *J* = 8.4, 7.4 Hz, 1H), 7.22 (s, 1H).

^{13}C NMR (101 MHz, dichloromethane-d_2) δ 154.3, 147.5, 130.7, 130.7, 130.0, 126.1, 125.2, 117.5, 114.9, 110.8, 89.8.

IR (Diamond-ATR, neat) $\tilde{\upsilon}_{max}$: 3527 (w), 2248 (s), 1599 (s), 1203 (s), 1123 (vs), 982 (w) cm^{-1}.

HRMS (ESI) calc. for $C_{11}H_4BrINO$ $[M-H]^-$: 371.8526 found: 371.8529.

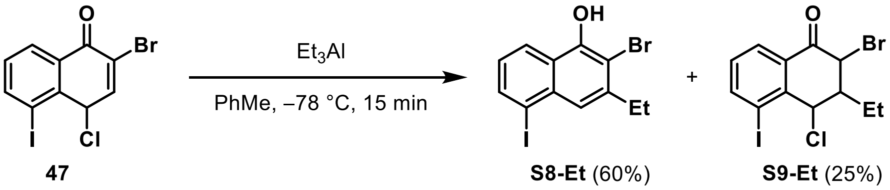

2-Bromo-3-ethyl-5-iodonaphthalen-1-ol (S8-Et)
2-Bromo-4-chloro-3-ethyl-5-iodo-3,4-dihydronaphthalen-1(2*H*)-one (S9-Et)

Triethylaluminium (25 wt% in PhMe, 23.0 µL, 51.3 µmol, 1.20 equiv) was added dropwise to a solution of enone **47** (16.4 mg, 42.8 µmol, 1 equiv) in toluene (428 µL, 0.1 M) at –78 ˚C. After 15 minutes of stirring at that temperature, the red solution was diluted with water (2 mL) and extracted with diethyl ether (3 × 3 mL). The combined organic layers were washed with a saturated aqueous solution of sodium chloride (4 mL) and the washed solution was dried over magnesium sulfate. The dried solution was filtered, and the filtrate was evaporated under reduced pressure. Purification by silica gel chromatography (3% ethyl acetate in cyclohexane) afforded compound **S8-Et** (9.70 mg, 25.7 µmol, 60%) as a while solid and compound **S9-Et** (4.40 mg, 10.7 µmol, 25%) as a reddish solid.

For analytical data of compound **S8-Et**: *see above.*

Compound **S9-Et**

TLC (20% dichloromethane in pentane): R_f = 0.24 (UV, CAM, $KMnO_4$).

mp: 69 °C.

^{1}H NMR (400 MHz, chloroform-*d*) δ 8.14 (dd, *J* = 7.9, 1.3 Hz, 1H), 8.10 (dd, *J* = 7.8, 1.4 Hz, 1H), 7.18 (t, *J* = 7.8 Hz, 1H), 5.88 (d, *J* = 4.3 Hz, 1H), 5.51 (d, *J* = 3.0 Hz, 1H), 2.95 – 2.82 (m, 1H), 2.12 – 1.94 (m, 1H), 1.07 – 0.95 (m, 4H).

^{13}C NMR (151 MHz, chloroform-*d*) δ 188.2, 146.2, 141.1, 131.3, 131.2, 128.7, 101.5, 64.8, 55.2, 52.6, 21.5, 12.2.

IR (Diamond-ATR, neat) $\tilde{\upsilon}_{max}$: 3012 (w), 1732 (s), 1637 (m), 1584 (s), 1429 (s), 1192 (m), 1038 (vs), 721 (w) $^{-1}$.

HRMS (ESI) calc. for $C_{12}H_{12}BrClIO$ $[M+H]^+$: 412.8799 found: 412.8797.

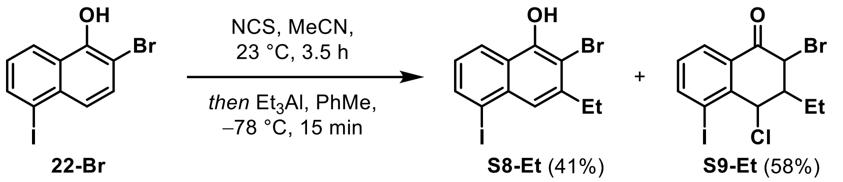

2-Bromo-3-ethyl-5-iodonaphthalen-1-ol (S8-Et)
2-Bromo-4-chloro-3-ethyl-5-iodo-3,4-dihydronaphthalen-1(2*H*)-one (S9-Et)

N-Chlorosuccinimide (8.40 mg, 63.0 µmol, 1.10 equiv) was added to a solution of naphthol **22-Br** (20.0 mg, 57.3 µmol, 1 equiv) in acetonitrile (600 µL, 0.10 M) in two portions over 15 minutes at 23 °C. After three hours, the solvent was removed under reduced pressure. The obtained crude solid was re-dissolved in toluene (800 µL, 0.07 M) and cooled to –78 °C before triethylaluminium (25 wt% in PhMe, 31.4 µL, 68.8 µmol, 1.20 equiv) was added dropwise. After ten minutes of stirring at that temperature, the solution was diluted with water (2 mL) and extracted with diethyl ether (3 × 3 mL). The combined organic layers were washed with a saturated aqueous solution of sodium chloride (4 mL) and the washed solution was dried over magnesium sulfate. The dried solution was filtered, and the filtrate was concentrated under reduced pressure. Purification by silica gel chromatography (3% ethyl acetate in cyclohexane) to afford compound **S8-Et** (8.90 mg, 23.5 µmol, 41%) as a while solid and compound **S9-Et** (13.7 mg, 33.2 µmol, 58%) as a reddish solid.

For analytical data: *see above.*

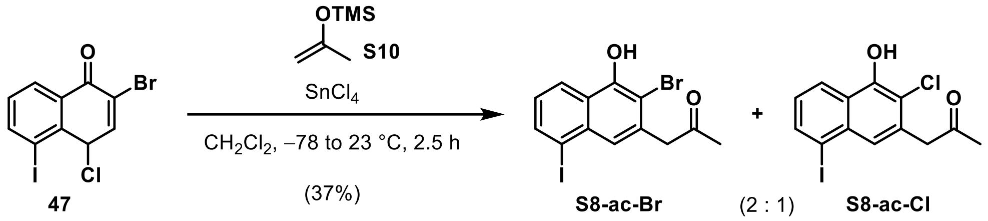

1-(3-Bromo-4-hydroxy-8-iodonaphthalen-2-yl)propan-2-one (S8-ac-Br)
1-(3-Chloro-4-hydroxy-8-iodonaphthalen-2-yl)propan-2-one (S8-ac-Cl)

Tin(IV)-chloride (1 M in dichloromethane, 42.2 µL, 42.2 µmol, 1.10 equiv) was added dropwise to a solution of enone **47** (14.7 mg, 38.3 µmol, 1 equiv) and silyl enol ether **S10** (85% purity, 8.00 µL, 42.2 µmol, 1.10 equiv) in dichloromethane (500 µL, 0.08 M) at –78 ˚C. The reaction was stirred for two hours while slowly warming to 23 °C. Additional silyl enol ether **S10** (85% purity, 50.0 µL, 264 µmol, 6.88 equiv) was added dropwise and the reaction was stirred at 23 °C for 30 minutes. After addition of water (4 mL), the crude mixture was extracted with ethyl acetate (3 × 10 mL). The combined organic layers were washed with a saturated aqueous solution of sodium chloride (5 mL) and the washed solution was dried over magnesium sulfate. The dried solution was filtered. The filtrate was evaporated under reduced pressure and purified by silica gel chromatography (15% dichloromethane in cyclohexane) to give the title compounds **S8-ac-Br** and **S8-ac-Cl** (5.40 mg, 13.3 mmol, 37%) as an inseparable mixture in a ratio of 2:1.

Mixture (2:1) of **S8-ac-Br** and **S8-ac-Cl**

TLC (80% dichloromethane in cyclohexane): R_f = 0.32 (UV, CAM, $KMnO_4$).

^{1}H NMR (400 MHz, chloroform-*d*) δ 8.25 (dt, *J* = 8.4, 1.0 Hz, 1H), 8.22 (dt, *J* = 8.4, 1.0 Hz, 0.5H), 8.10 (dd, *J* = 7.4, 1.2 Hz, 1H), 8.09 (dd, *J* = 7.4, 1.1 Hz, 0.5H), 7.56 (s, 1H), 7.55 (s, 0.5H), 7.19 – 7.15 (m, 1.5H), 6.13 (s, 1H), 6.12 (s, 0.5H), 4.05 (s, 2H), 4.03 (s, 1H), 2.28 (s, 4.5H).

^{13}C NMR (101 MHz, chloroform-*d*) δ 204.7, 204.7, 148.8, 147.6, 139.1, 138.9, 134.0, 133.5, 133.4, 132.0, 127.0, 127.0, 126.8, 126.7, 124.1, 123.9, 123.5, 123.3, 115.9, 108.6, 98.5, 98.5, 51.8, 49.4, 29.9, 29.8.

IR (Diamond-ATR, neat) $\tilde{\upsilon}_{max}$: 3432 (w), 1761 (s), 1620 (s), 1229 (s), 1093 (s), 938 (m), 798 (w) cm^{-1}.

HRMS (ESI) calc. for $C_{13}H_8BrIO_2$ $[M–H]^-$: 402.8836 found: 402.8831.

HRMS (ESI) calc. for $C_{13}H_8ClIO_2$ $[M–H]^-$: 358.9341 found: 358.9337.

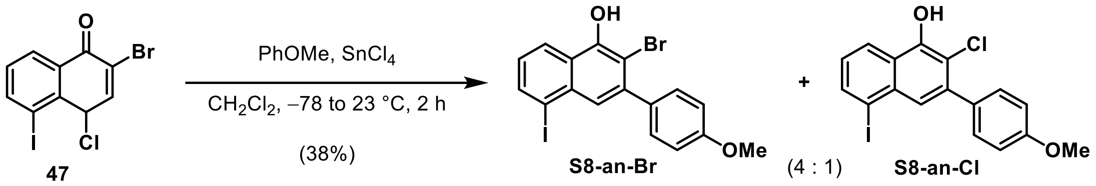

2-Bromo-5-iodo-3-(4-methoxyphenyl)naphthalen-1-ol (S8-an-Br)
2-Chloro-5-iodo-3-(4-methoxyphenyl)naphthalen-1-ol (S8-an-Cl)

Tin(IV)-chloride (1 M in dichloromethane, 242 µL, 242 µmol, 1.20 equiv) was added dropwise to a solution of ketone **47** (42.0 mg, 110 µmol, 1 equiv) and anisole (239 µL, 2.19 mmol, 20.0 equiv) in dichloromethane (1.38 mL, 0.08 M) at –30 ˚C. The reaction was slowly warmed to 23 °C within 1.5 hours and then stirred for further 30 minutes at that temperature. The solvent was removed under reduced pressure to give an orange-brown solid. The sensitive crude product was dissolved in a mixture of cyclohexane and ethyl acetate (19:1) and immediately subjected to flash-column-chromatography on silica gel under a nitrogen-stream to give an inseparable mixture of compounds **S8-an-Br** and **S8-an-Cl** (19.0 mg, 41.8 µmol, 38%) in a ratio of 4:1.

Mixture (4:1) of **S8-an-Br** and **S8-an-Cl**

TLC (14% ethyl acetate in cyclohexane): R_f = 0.45 (UV, CAM, $KMnO_4$).

^{1}H NMR (400 MHz, chloroform-*d*) δ 8.40 (dd, *J* = 8.4, 1.3 Hz, 1.25H), δ 8.31 – 8.21 (m, 0.5H), 8.25 (dd, *J* = 7.3, 1.3 Hz, 1H), 8.11 (td, *J* = 7.3, 1.2 Hz, 0.5H), 7.50 (s, 1.25H), 7.21 – 7.16 (m, 2.5H), 7.12 (dd, *J* = 8.4, 7.3 Hz, 1.25H), 7.00 – 6.94 (m, 2.5H), 5.98 (s, 1.25H), 3.89 (s, 0.75H), 3.89 (s, 3H).

^{13}C NMR (101 MHz, chloroform-*d*)[1] δ 159.7, 147.8, 143.2, 135.2, 133.0, 132.7, 132.7, 131.7, 126.8, 126.4, 123.6, 113.5, 103.7, 92.4, 55.5.

IR (Diamond-ATR, neat) $\tilde{\upsilon}_{max}$: 3131 (w), 1689 (m), 1513 (vs), 1074 (s), 999 (m), 783 (w) cm^{-1}.

HRMS (ESI) calc. for $C_{17}H_{11}BrIO_2$ $[M\text{-}H]^-$: 452.8993 found: 452.8995.

HRMS (ESI) calc. for $C_{17}H_{11}ClIO_2$ $[M\text{-}H]^-$: 408.9498 found: 408.9492.

[1]*Only the set of signals for the 2-bromo-naphthol* ***S8-an-Br*** *is given due to the low intensity of the 2-chloro-naphtho-signals.*

5. NMR-Spectra

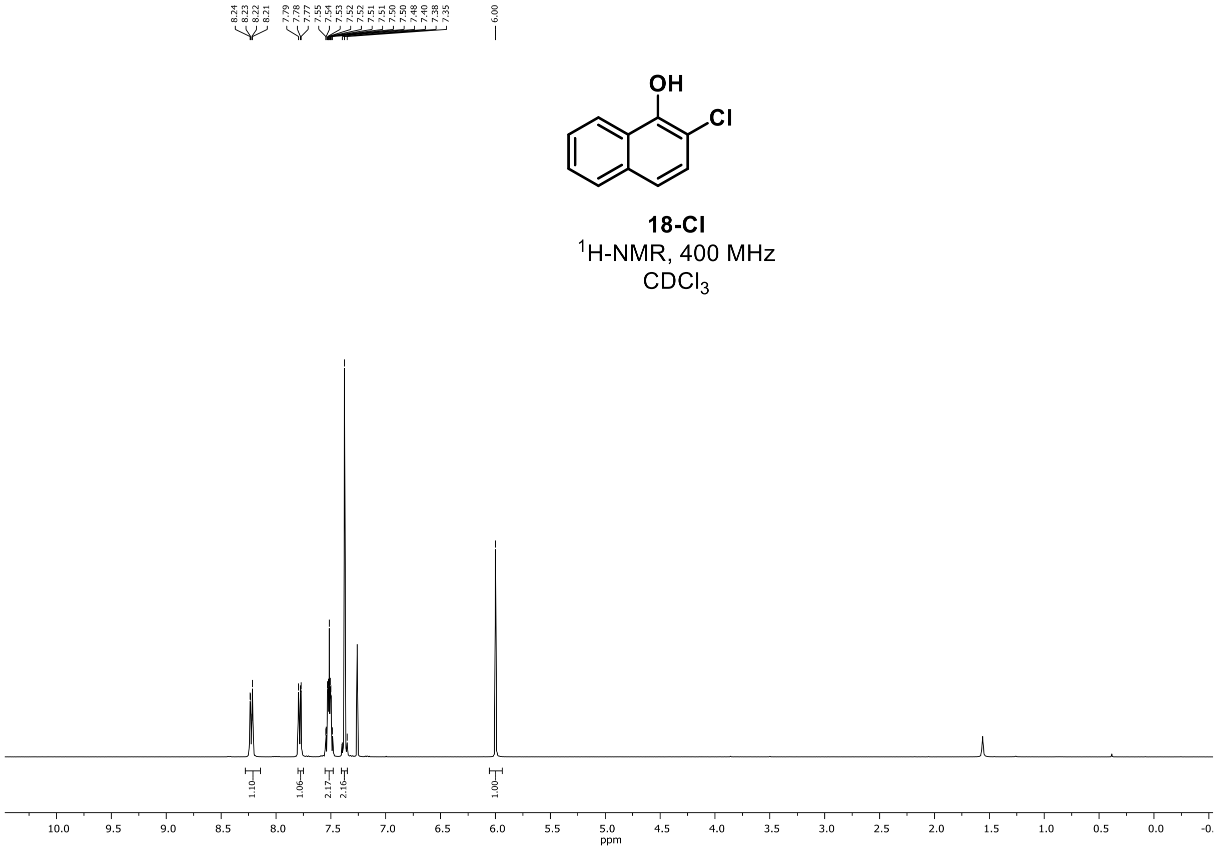

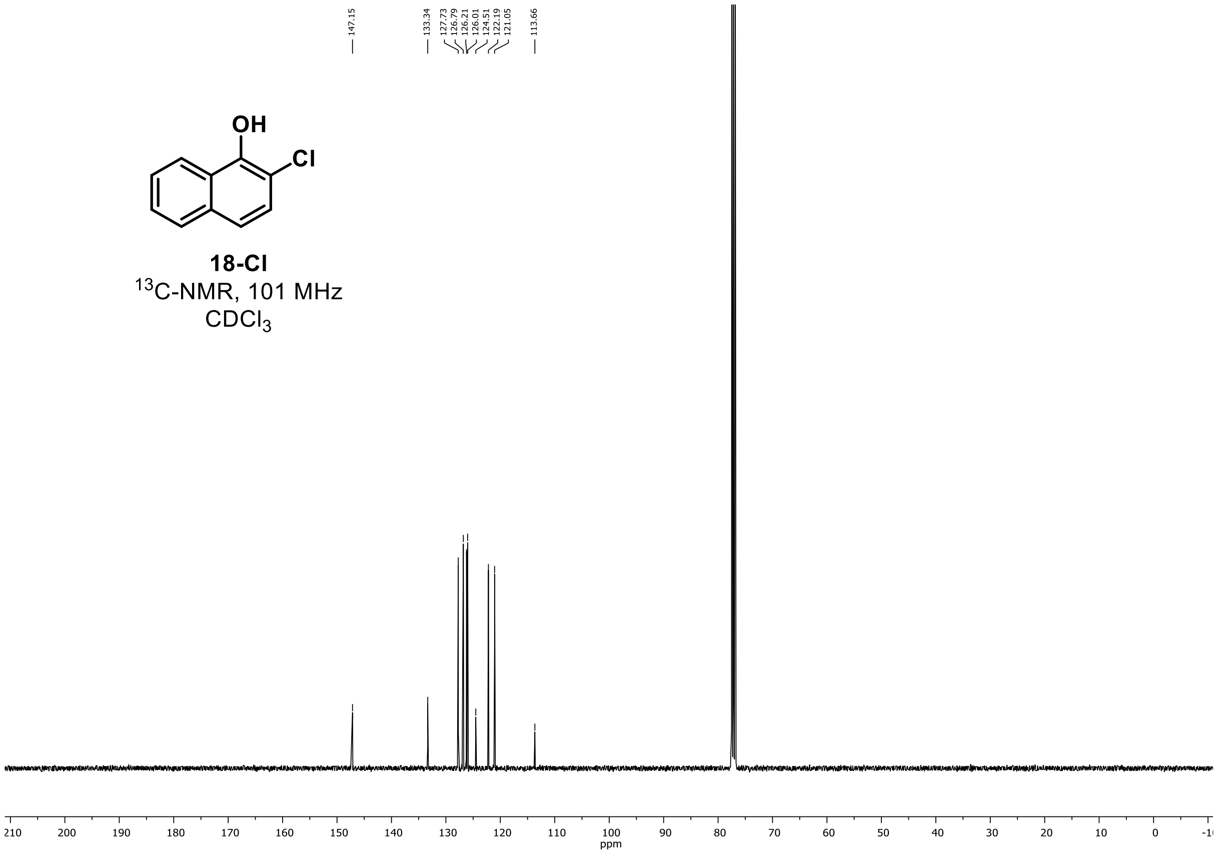

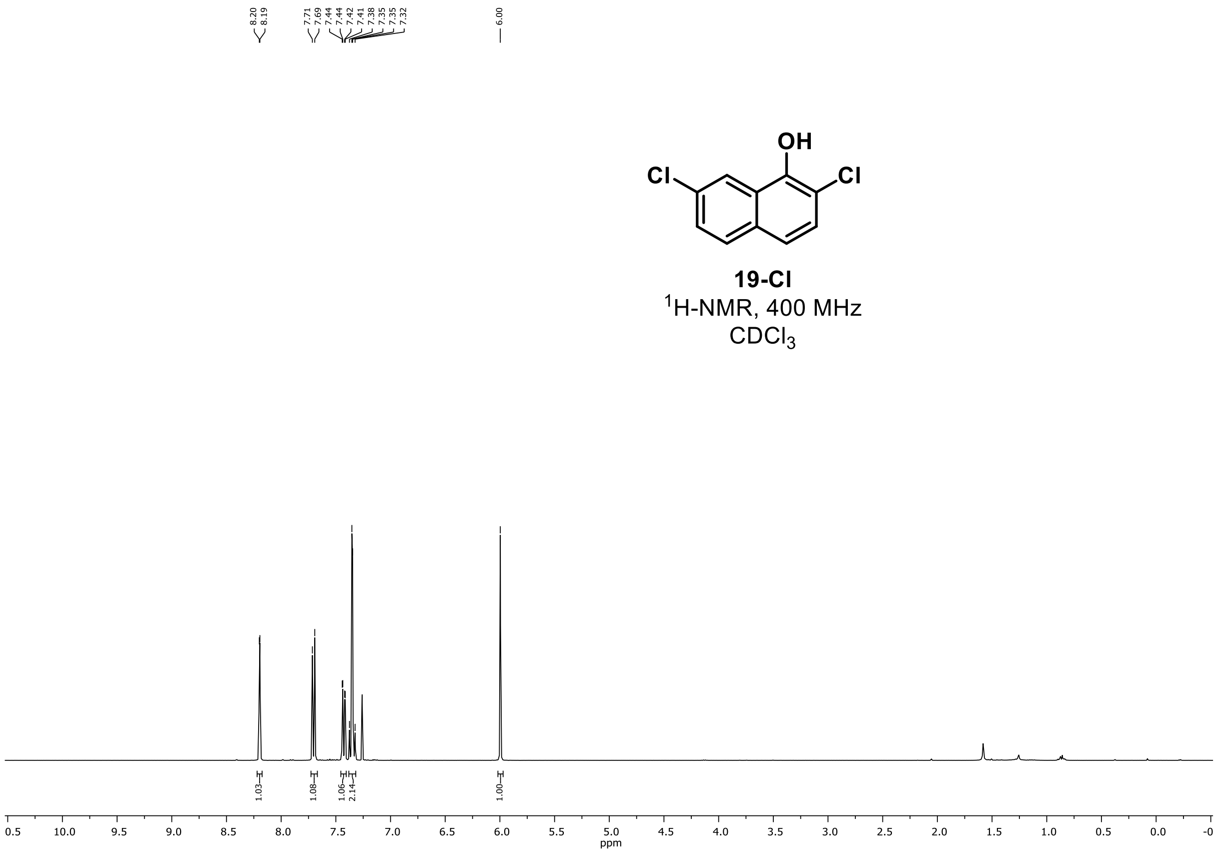
8.20
8.19
7.71
7.69
7.44
7.44
7.42
7.41
7.38
7.35
7.35
7.32
6.00
OH
Cl
Cl
19-Cl
1H-NMR, 400 MHz
CDCl3
1.03
1.08
1.06
2.14
1.00
ppm

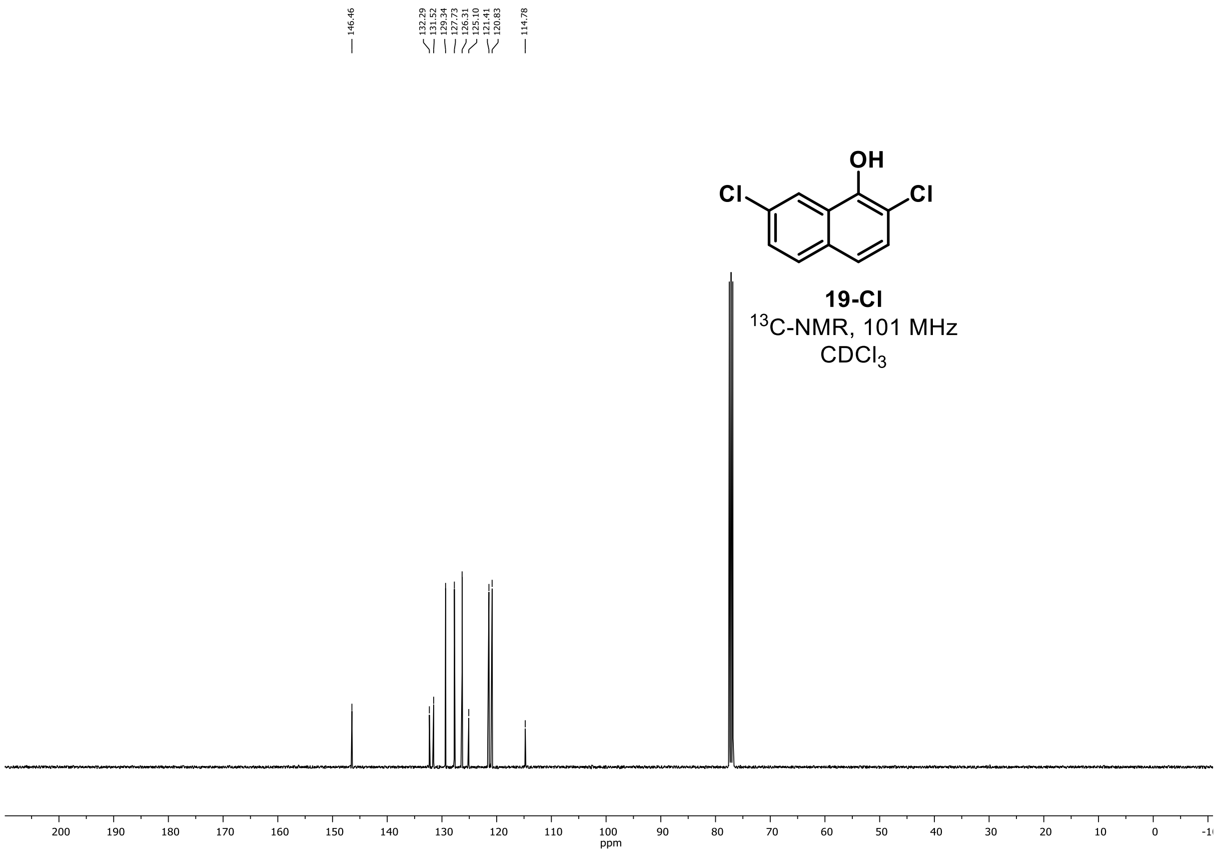
146.46
132.29
131.52
129.34
127.73
126.31
125.10
121.41
120.83
114.78
OH
Cl
Cl
19-Cl
13C-NMR, 101 MHz
CDCl3
ppm

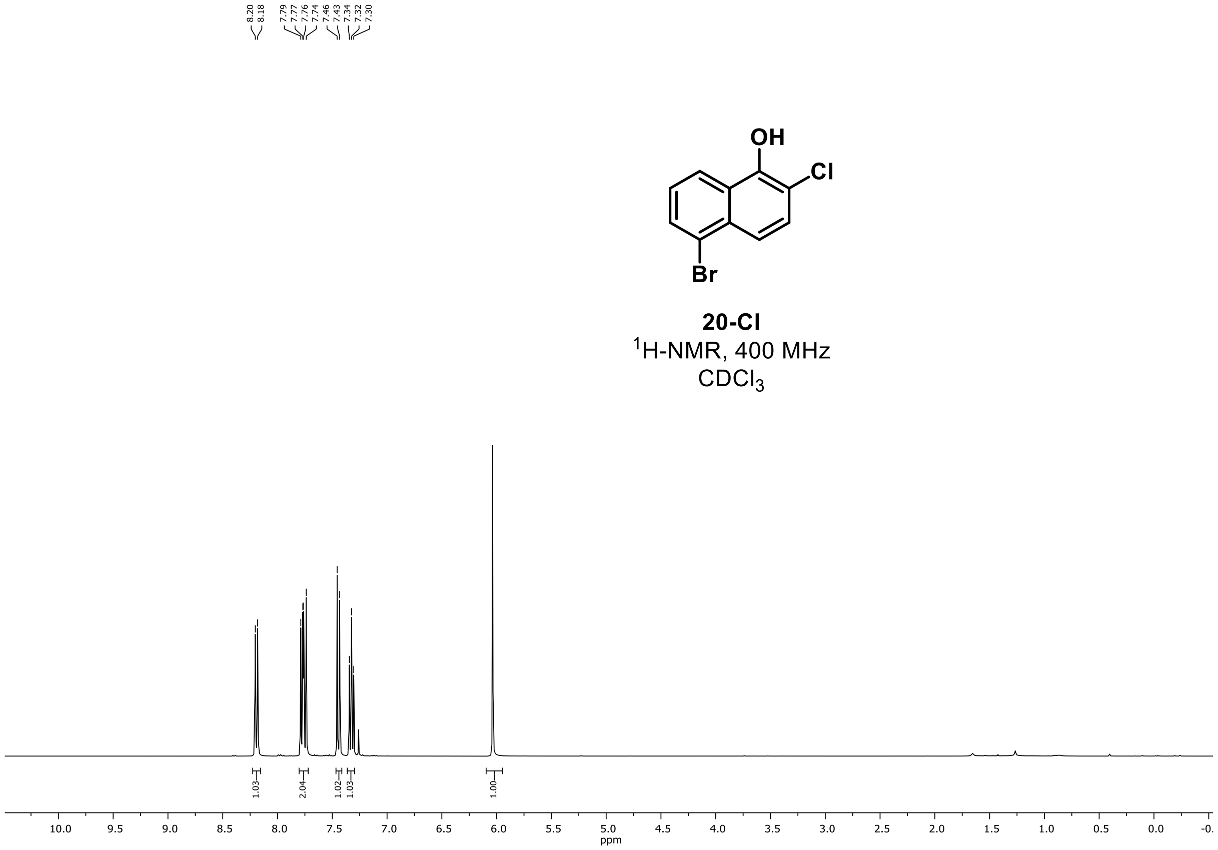

20-Cl
^{1}H-NMR, 400 MHz
$CDCl_3$

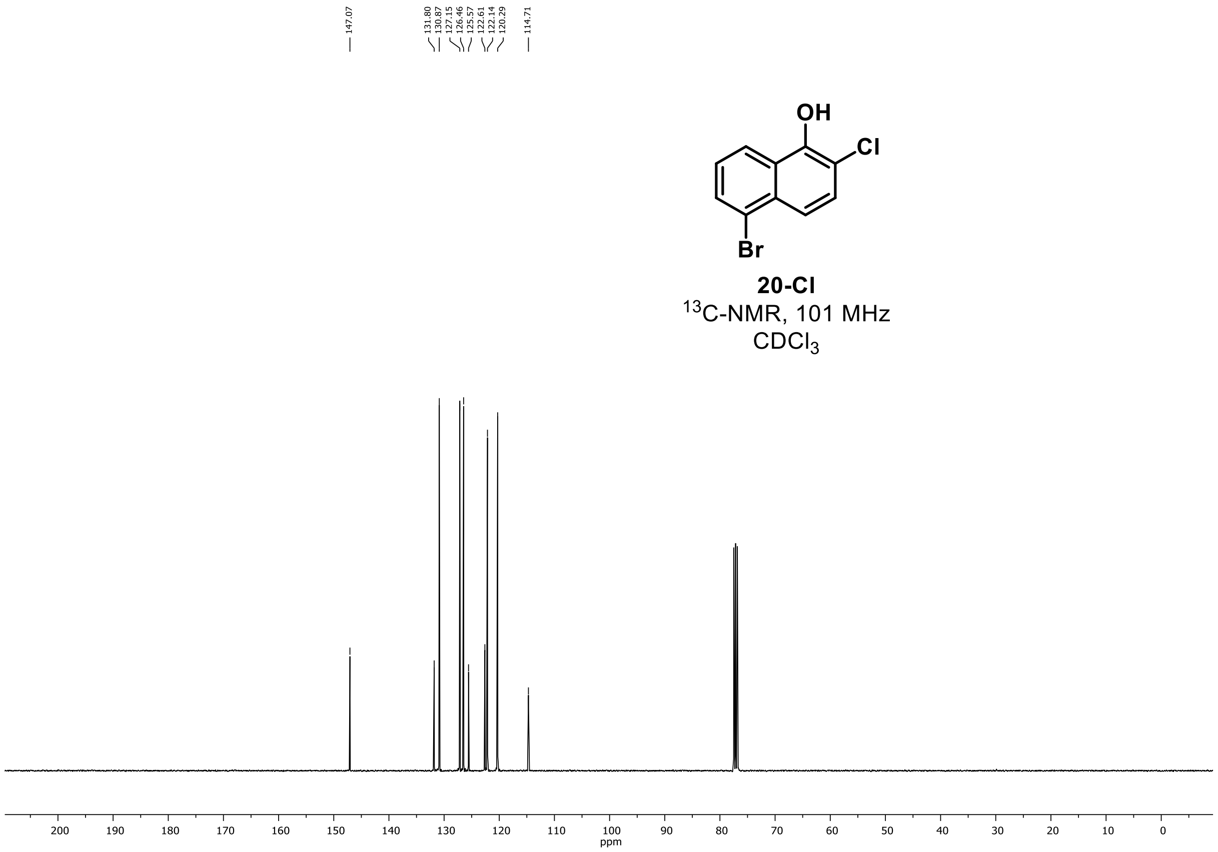

20-Cl
^{13}C-NMR, 101 MHz
$CDCl_3$

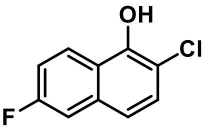

21-Cl
^{1}H-NMR, 400 MHz
$CDCl_3$

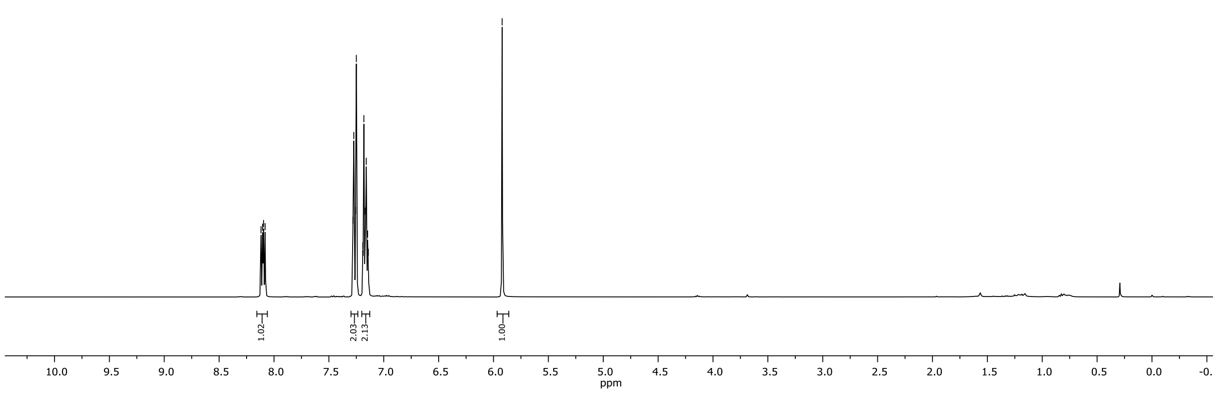

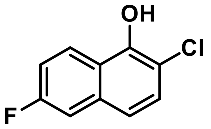

21-Cl
^{13}C-NMR, 101 MHz
$CDCl_3$

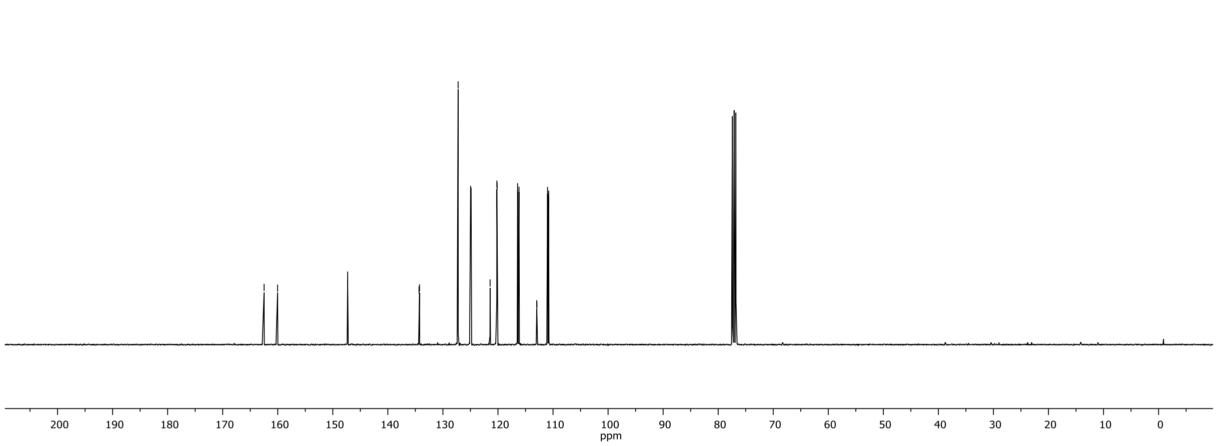

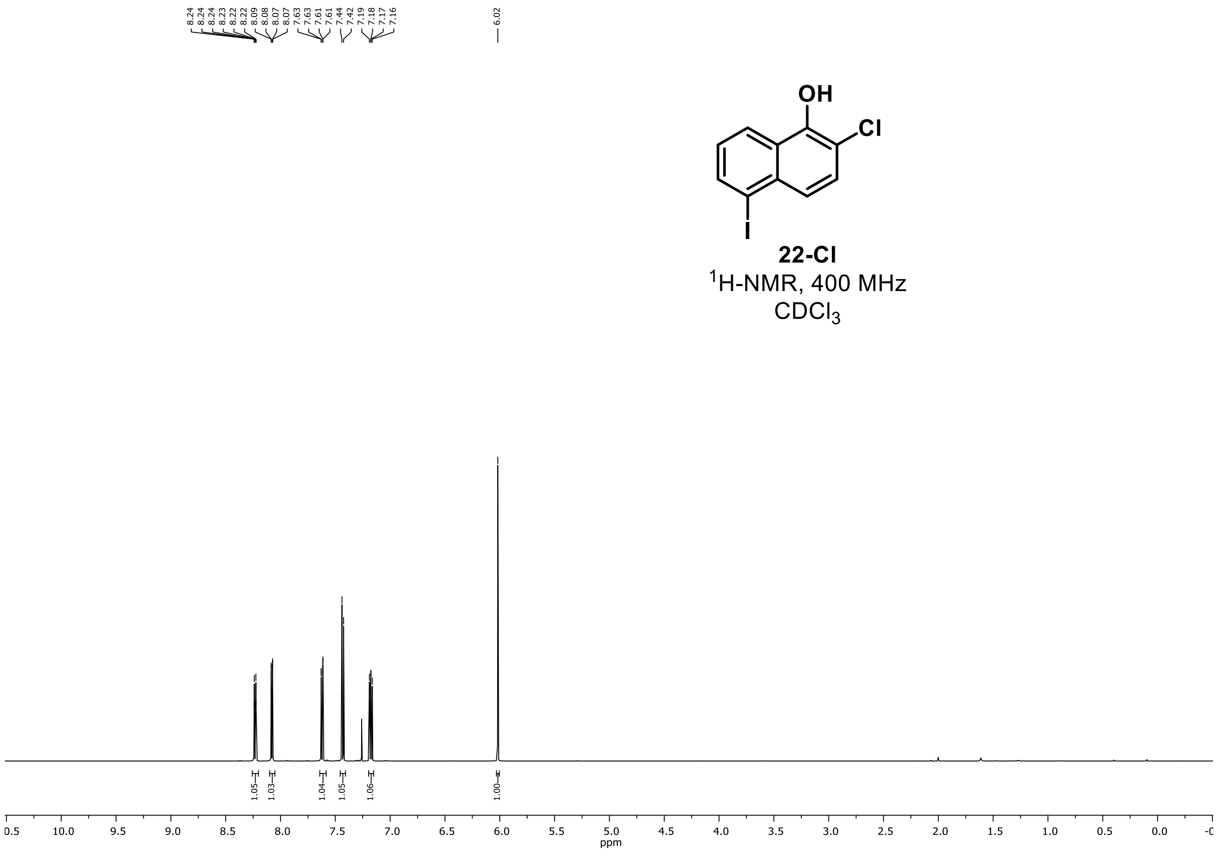

22-Cl
^{1}H-NMR, 400 MHz
$CDCl_3$

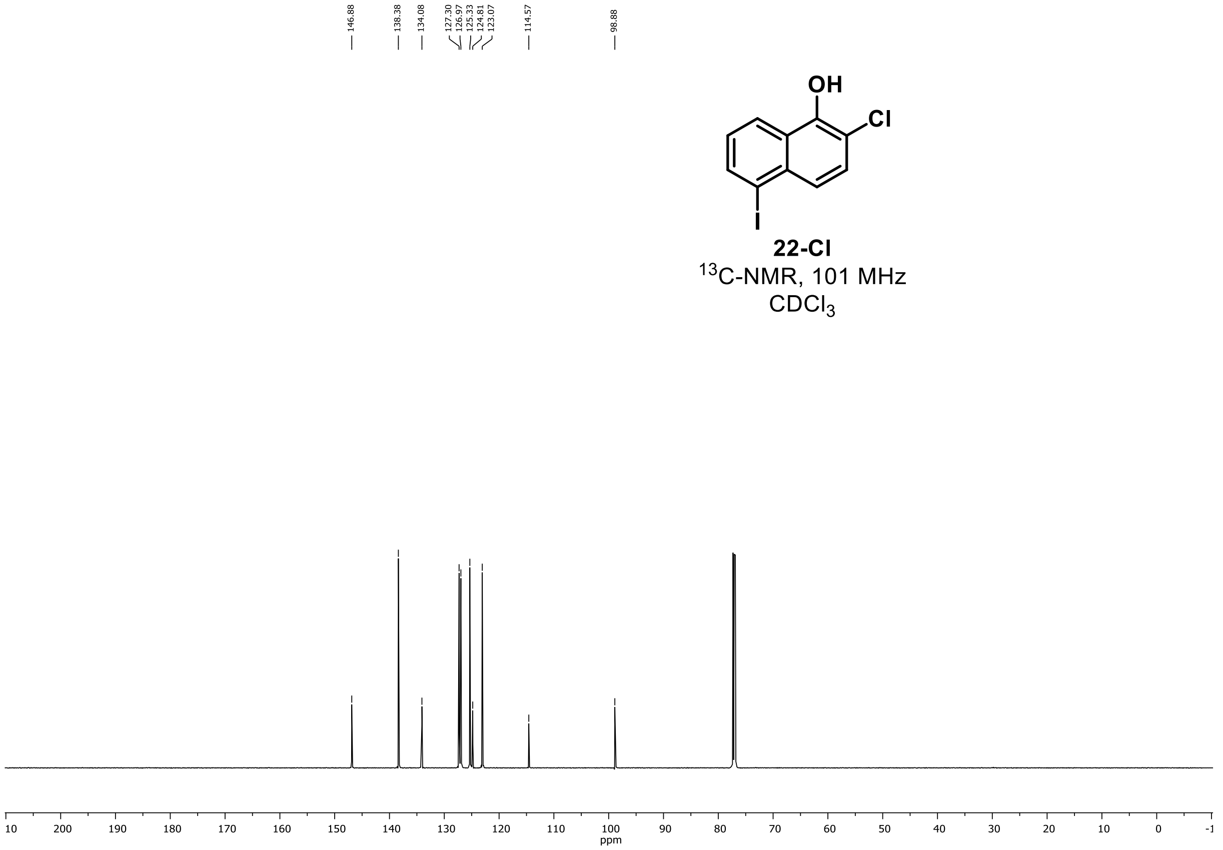

22-Cl
^{13}C-NMR, 101 MHz
$CDCl_3$

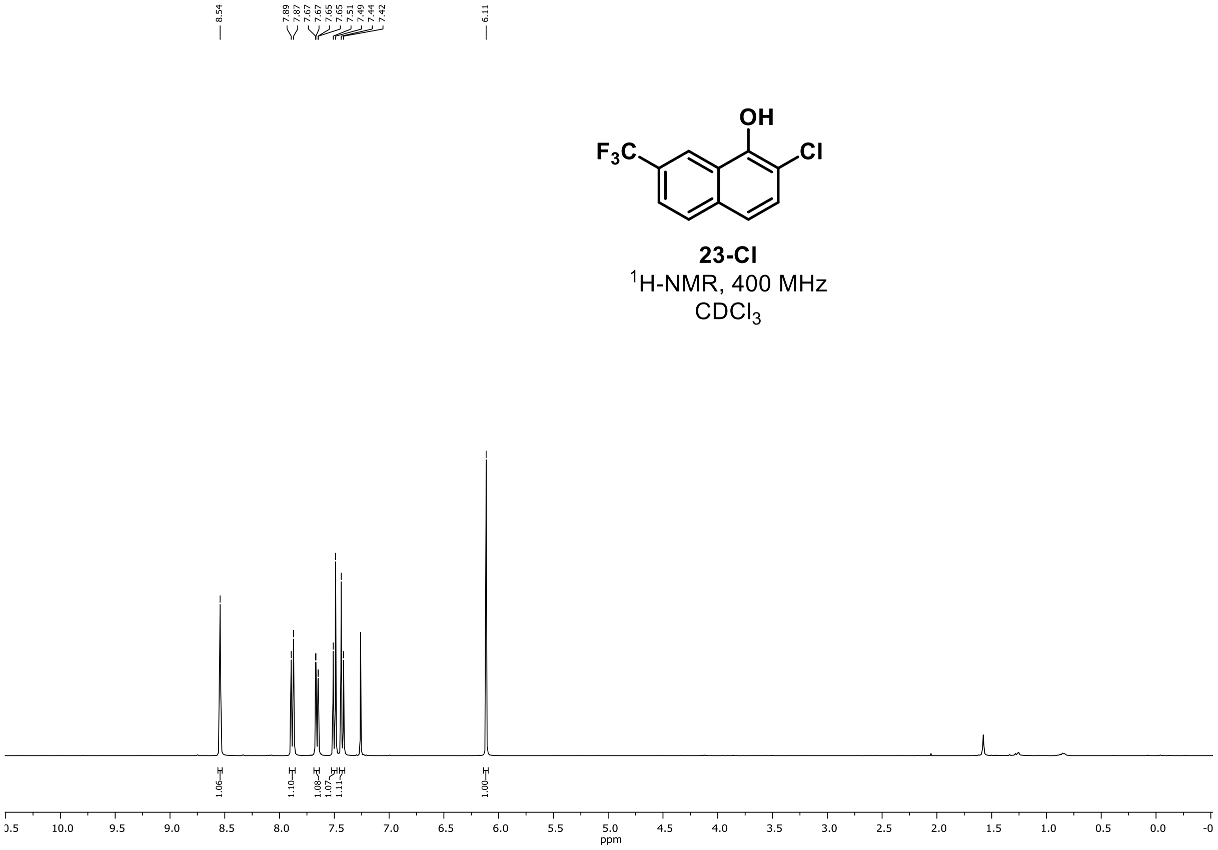
23-Cl
1H-NMR, 400 MHz
CDCl3
ppm

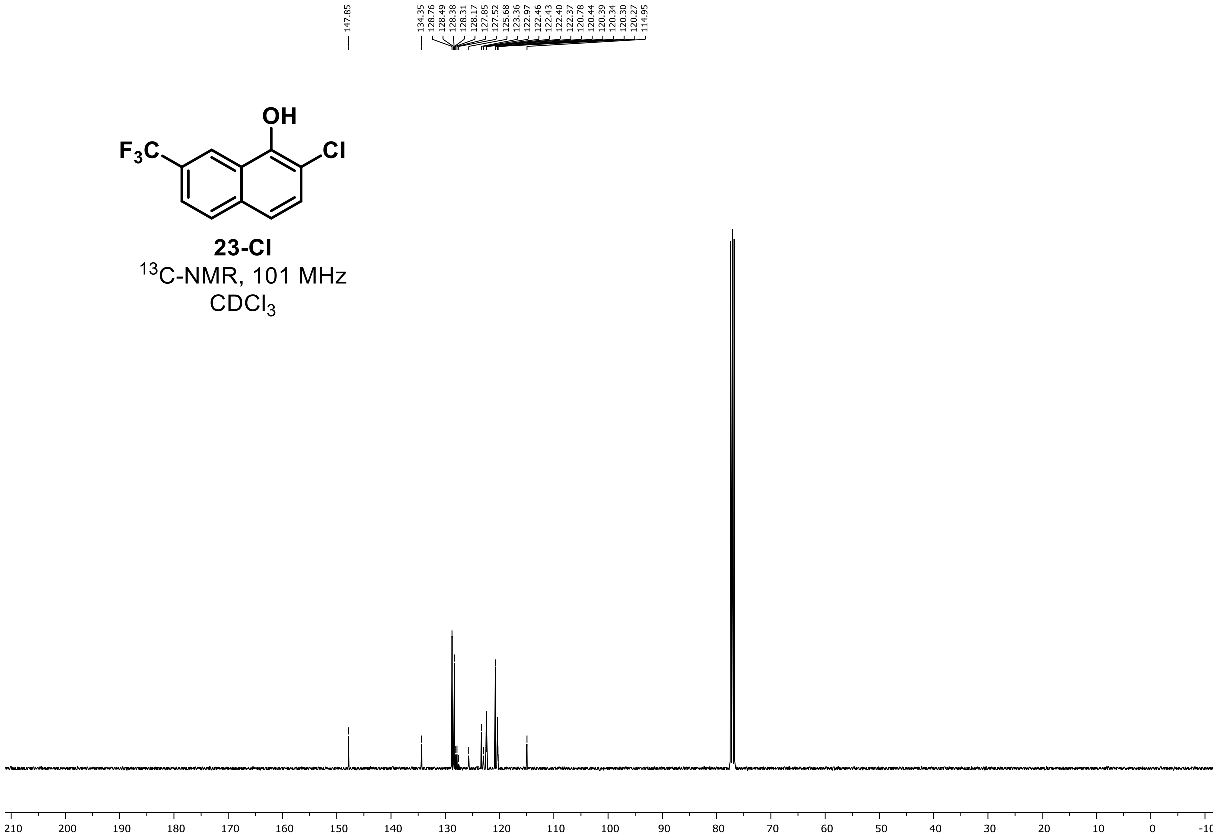
23-Cl
13C-NMR, 101 MHz
CDCl3
ppm

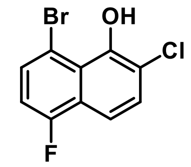

24-Cl

^{1}H-NMR, 400 MHz

$CDCl_3$

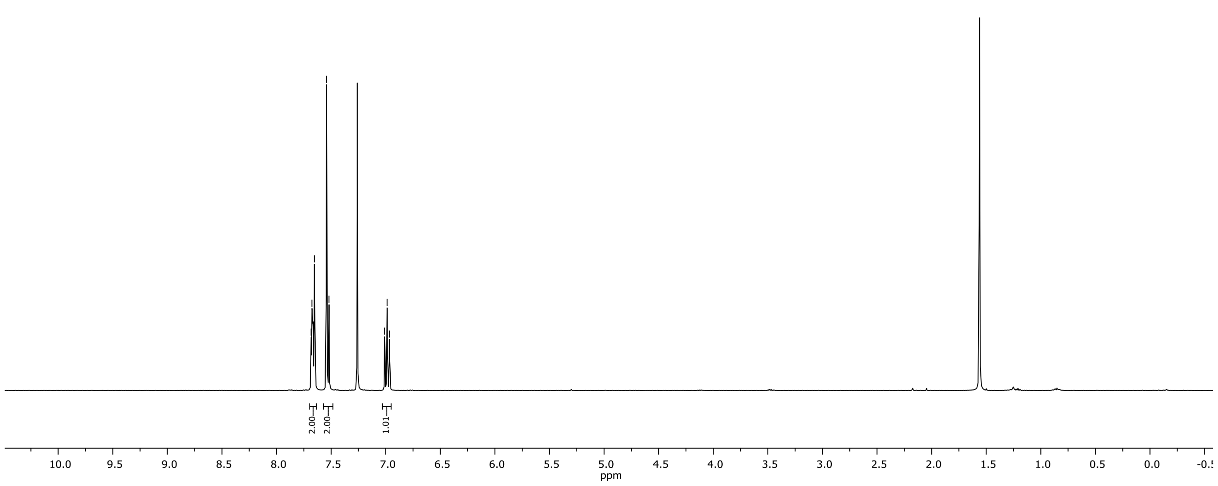

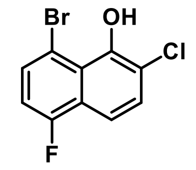

24-Cl

^{13}C-NMR, 101 MHz

$CDCl_3$

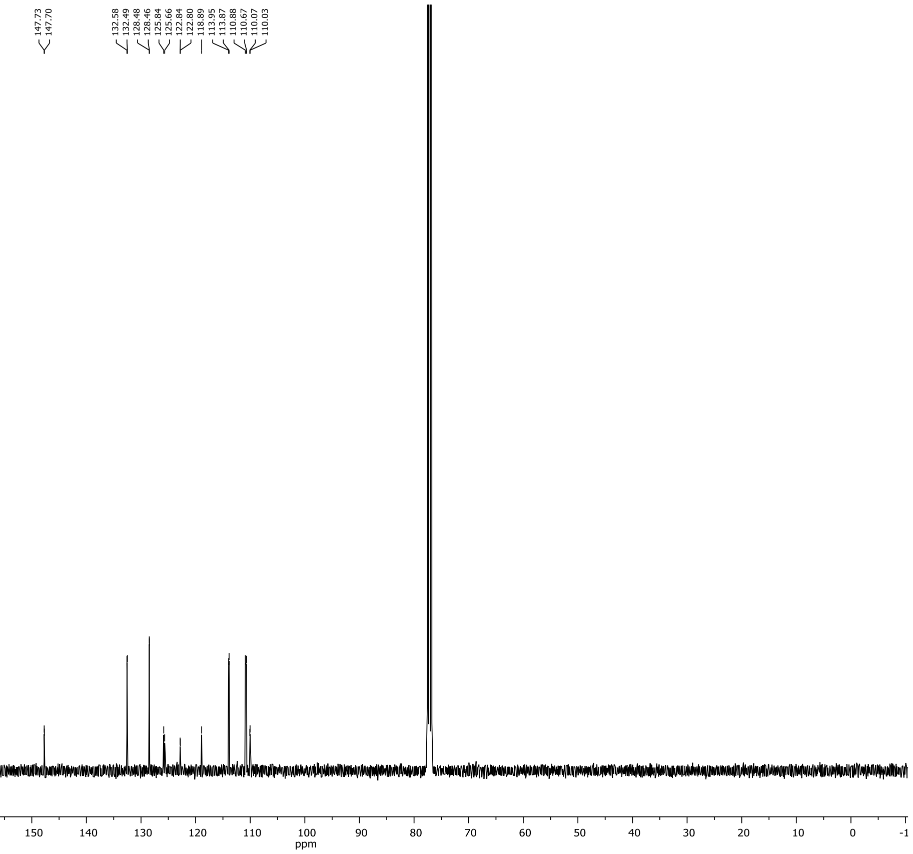

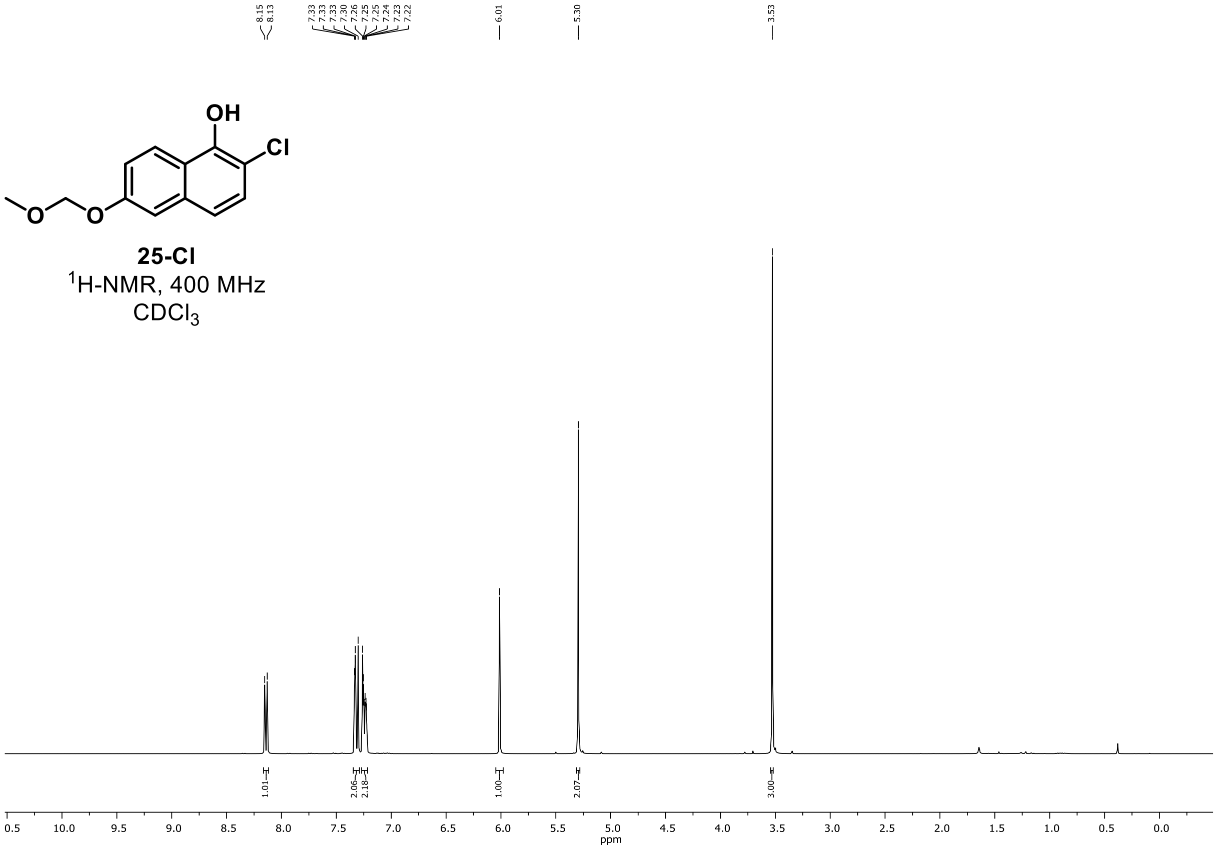
8.15
8.13
7.33
7.33
7.33
7.30
7.26
7.25
7.25
7.24
7.23
7.22
6.01
5.30
3.53
OH
Cl
O
O
25-Cl
^{1}H-NMR, 400 MHz
$CDCl_3$
1.01
2.06
2.18
1.00
2.07
3.00
ppm

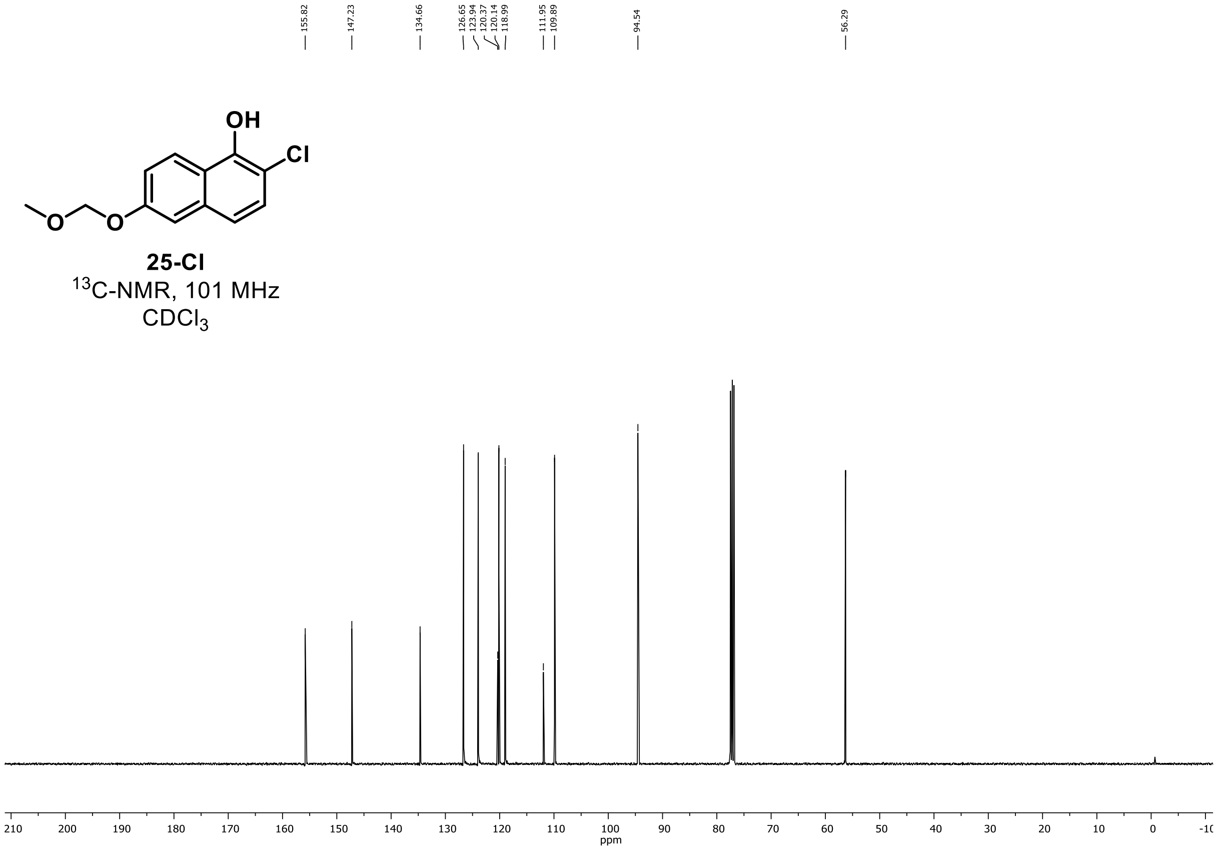
155.82
147.23
134.66
126.65
123.94
120.37
120.14
118.99
111.95
109.89
94.54
56.29
OH
Cl
O
O
25-Cl
^{13}C-NMR, 101 MHz
$CDCl_3$
ppm

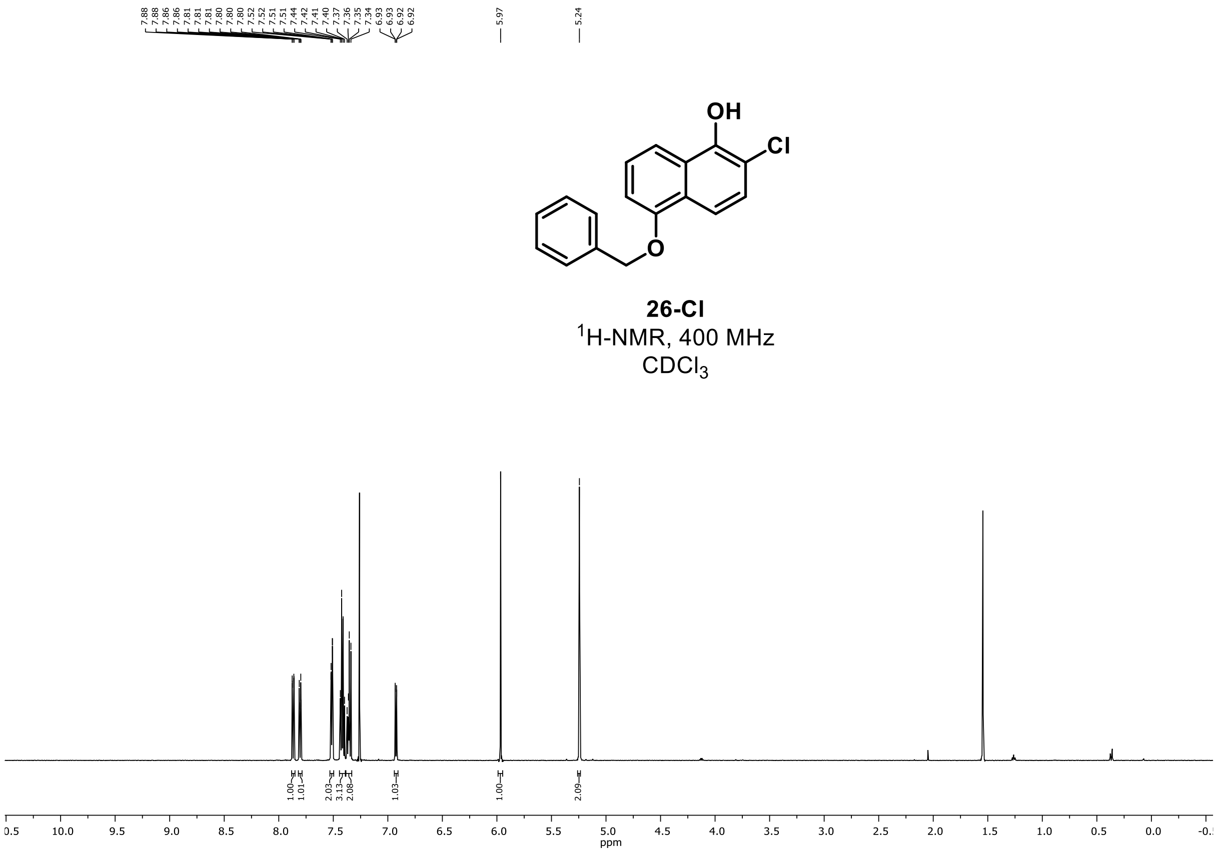
7.88 7.88 7.86 7.86 7.81 7.81 7.81 7.80 7.80 7.80 7.52 7.52 7.51 7.51 7.44 7.42 7.41 7.40 7.37 7.36 7.35 7.34 6.93 6.93 6.92 6.92
5.97
5.24
OH
Cl
O
26-Cl
^{1}H-NMR, 400 MHz
$CDCl_3$
1.00 1.01 2.03 3.13 2.08 1.03 1.00 2.09
ppm

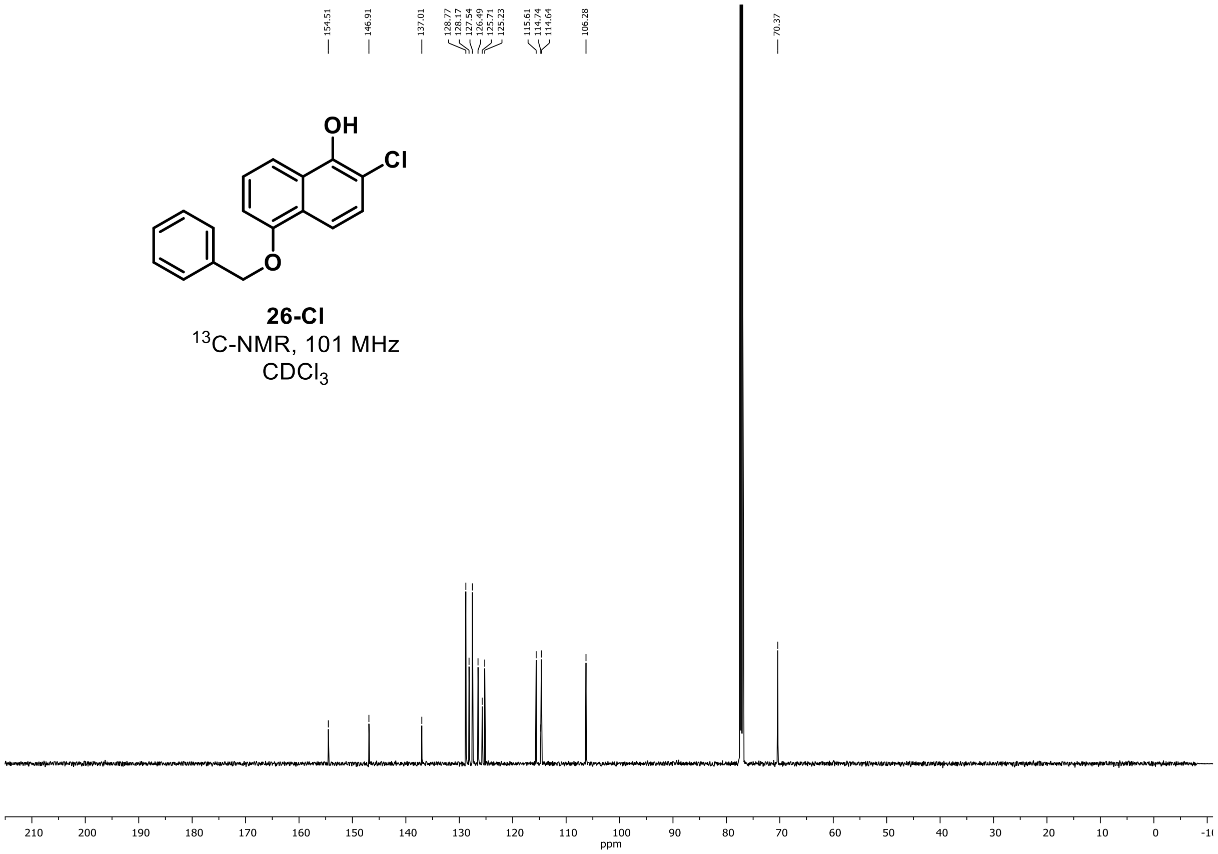
154.51
146.91
137.01
128.77 128.17 127.54 126.49 125.71 125.23
115.61 114.74 114.64
106.28
70.37
OH
Cl
O
26-Cl
^{13}C-NMR, 101 MHz
$CDCl_3$
ppm

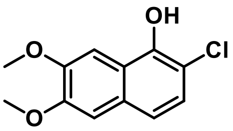

27-Cl
^{1}H-NMR, 400 MHz
$CDCl_3$

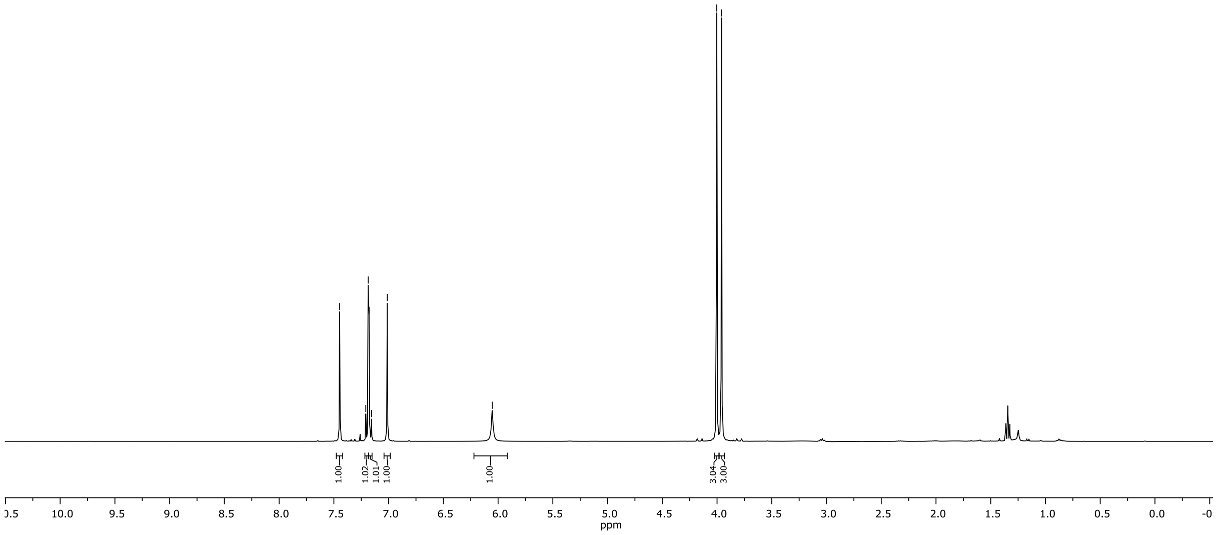

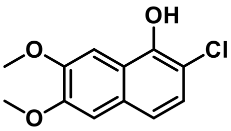

27-Cl
^{13}C-NMR, 101 MHz
$CDCl_3$

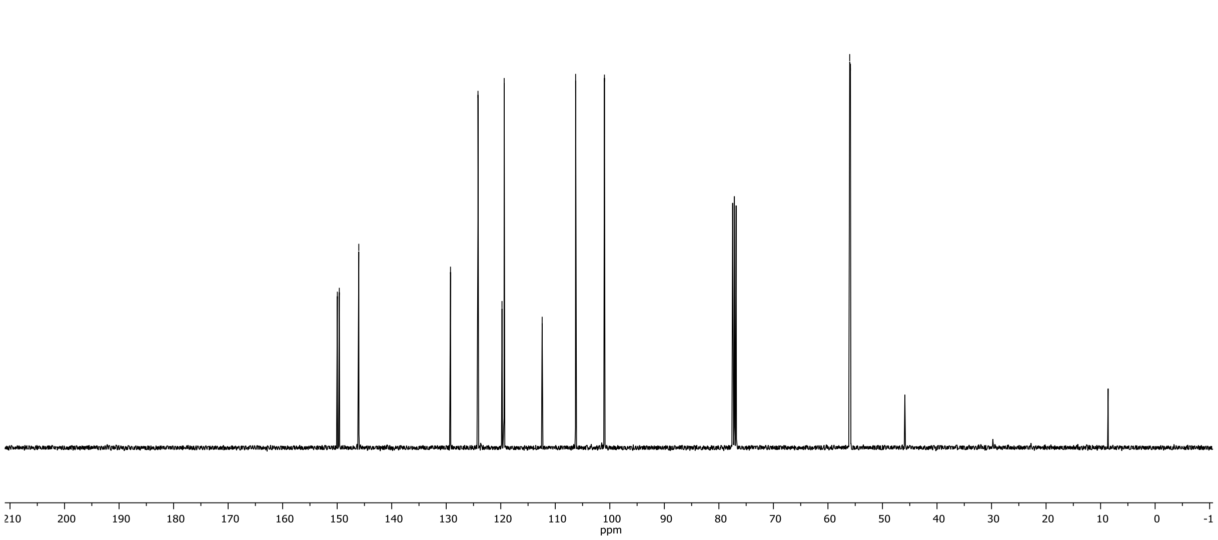

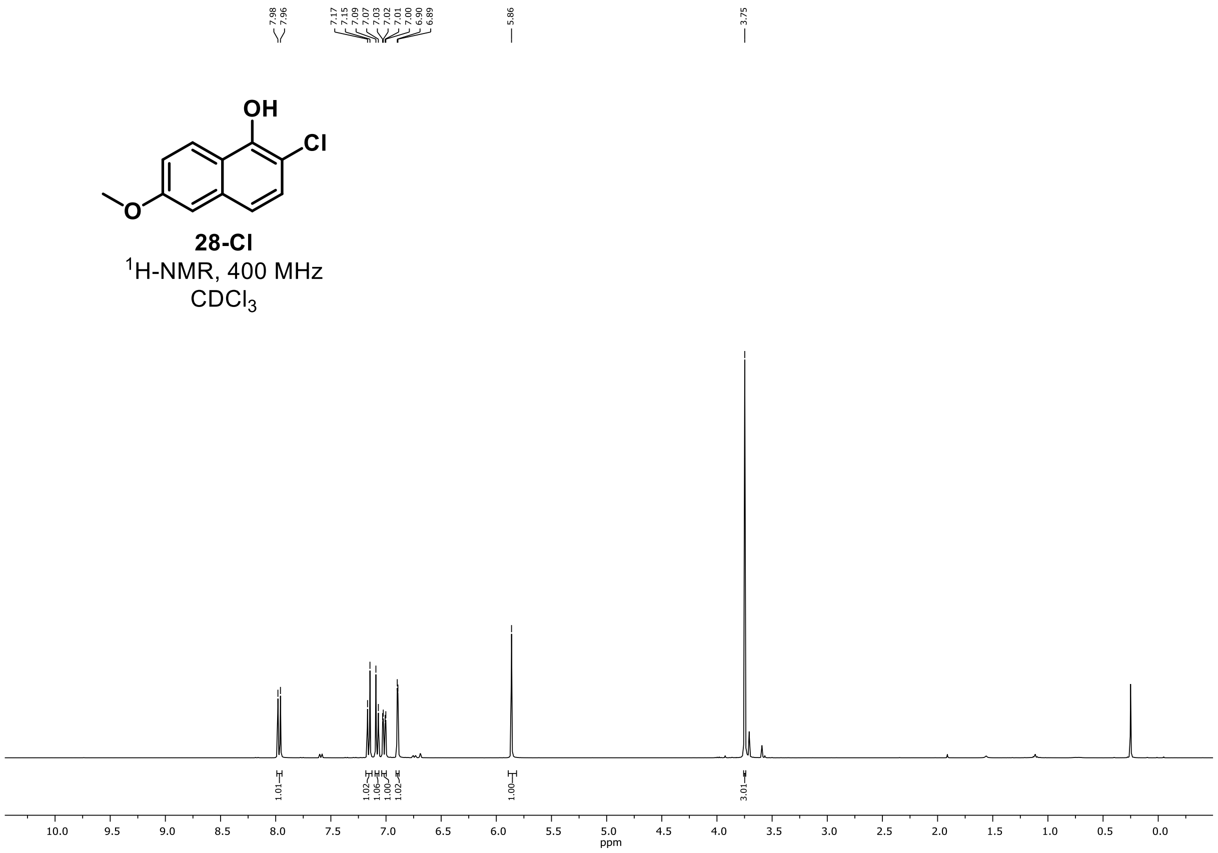

28-Cl
^{1}H-NMR, 400 MHz
$CDCl_3$

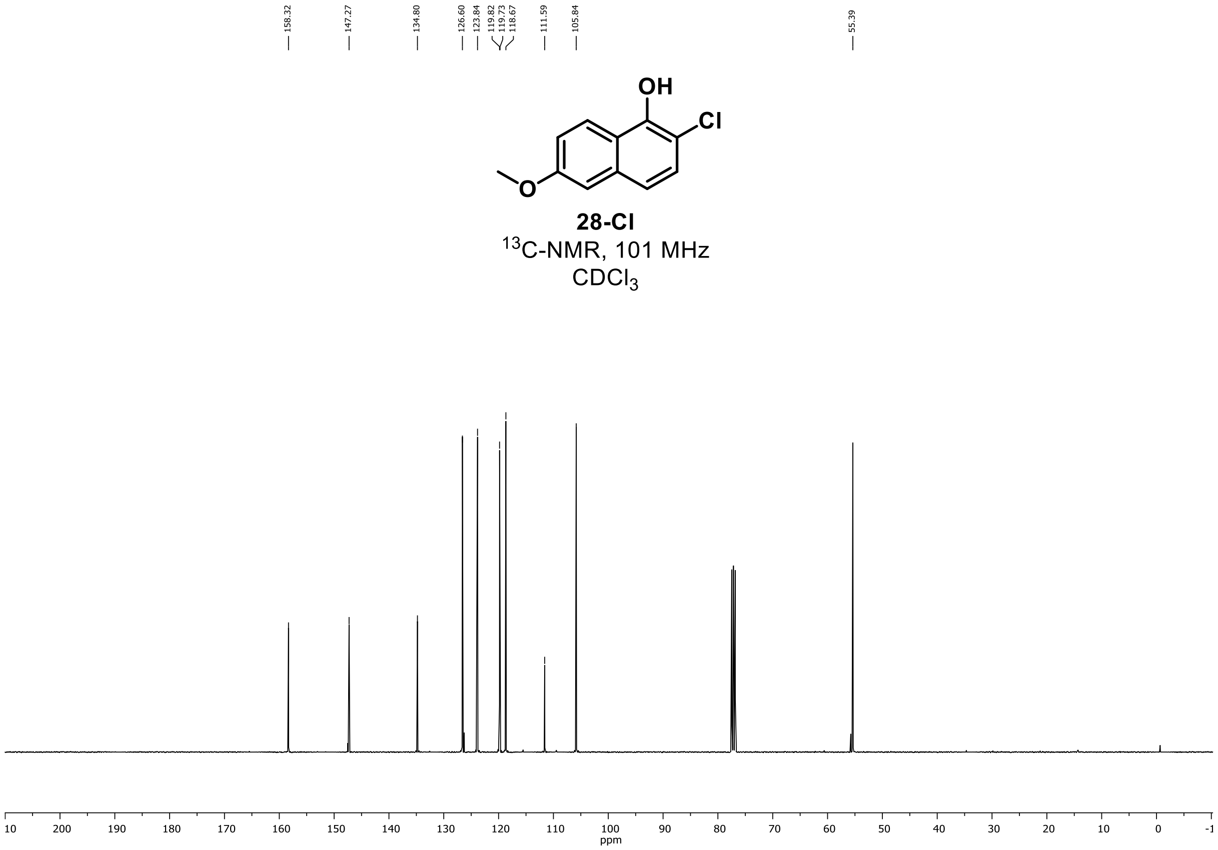

28-Cl
^{13}C-NMR, 101 MHz
$CDCl_3$

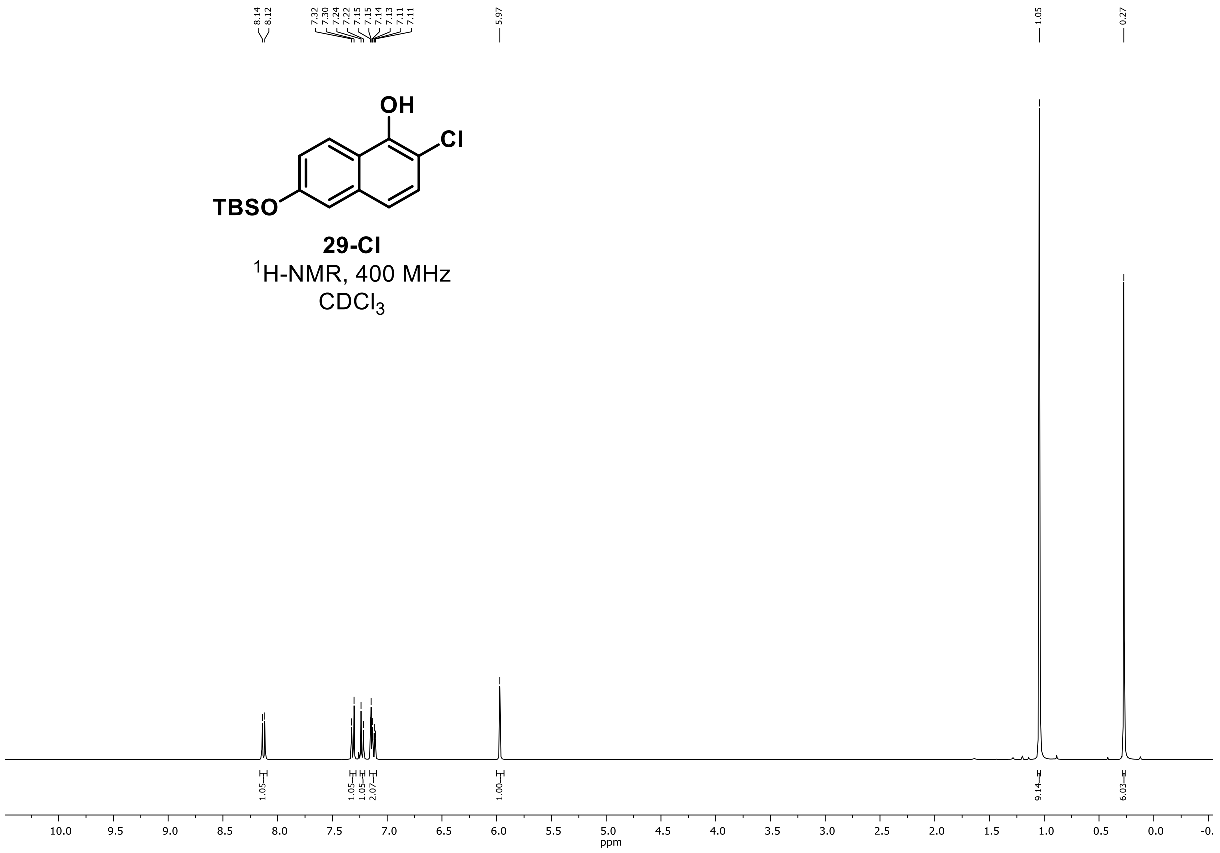
8.14
8.12
7.32
7.30
7.24
7.22
7.15
7.15
7.14
7.13
7.11
7.11
5.97
1.05
0.27
OH
Cl
TBSO
29-Cl
1H-NMR, 400 MHz
CDCl3
1.05
1.05
1.05
2.07
1.00
9.14
6.03
ppm

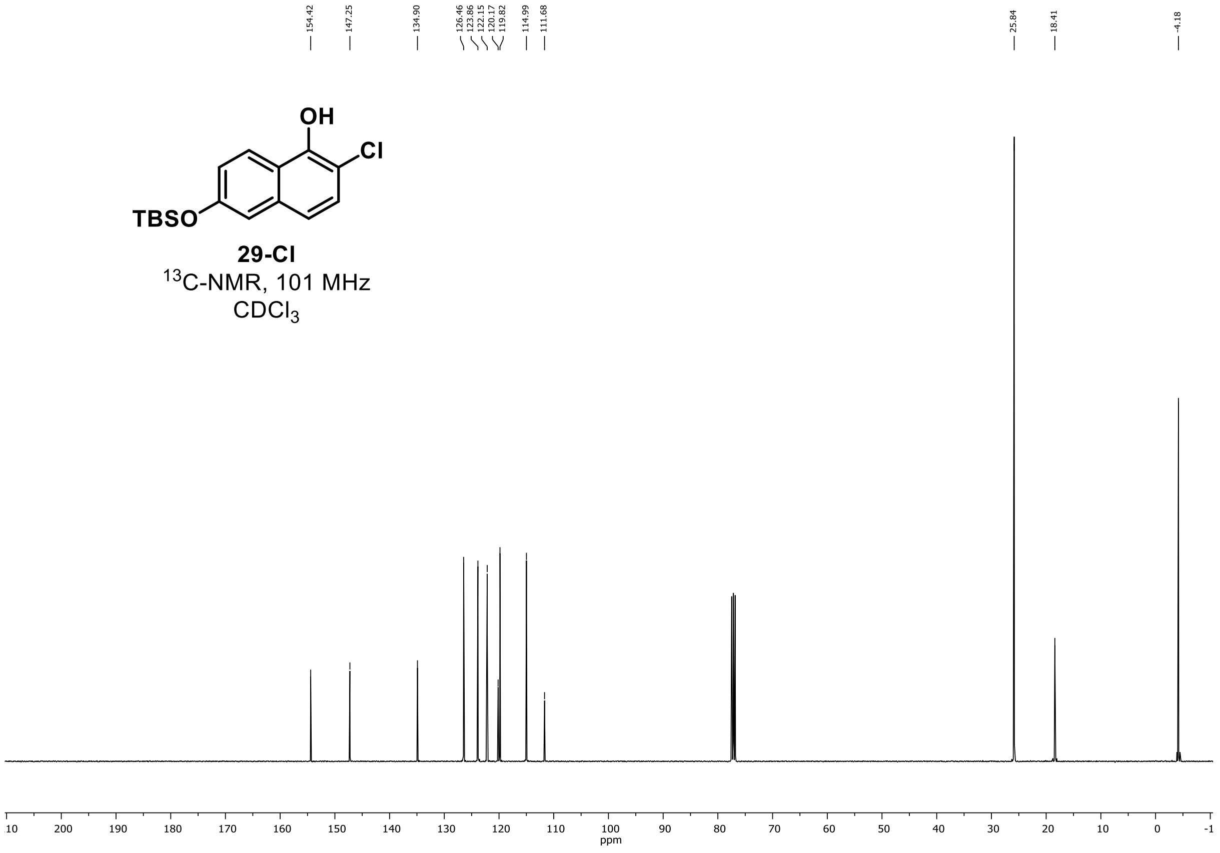
154.42
147.25
134.90
126.46
123.86
122.15
120.17
119.82
114.99
111.68
25.84
18.41
-4.18
OH
Cl
TBSO
29-Cl
13C-NMR, 101 MHz
CDCl3
ppm

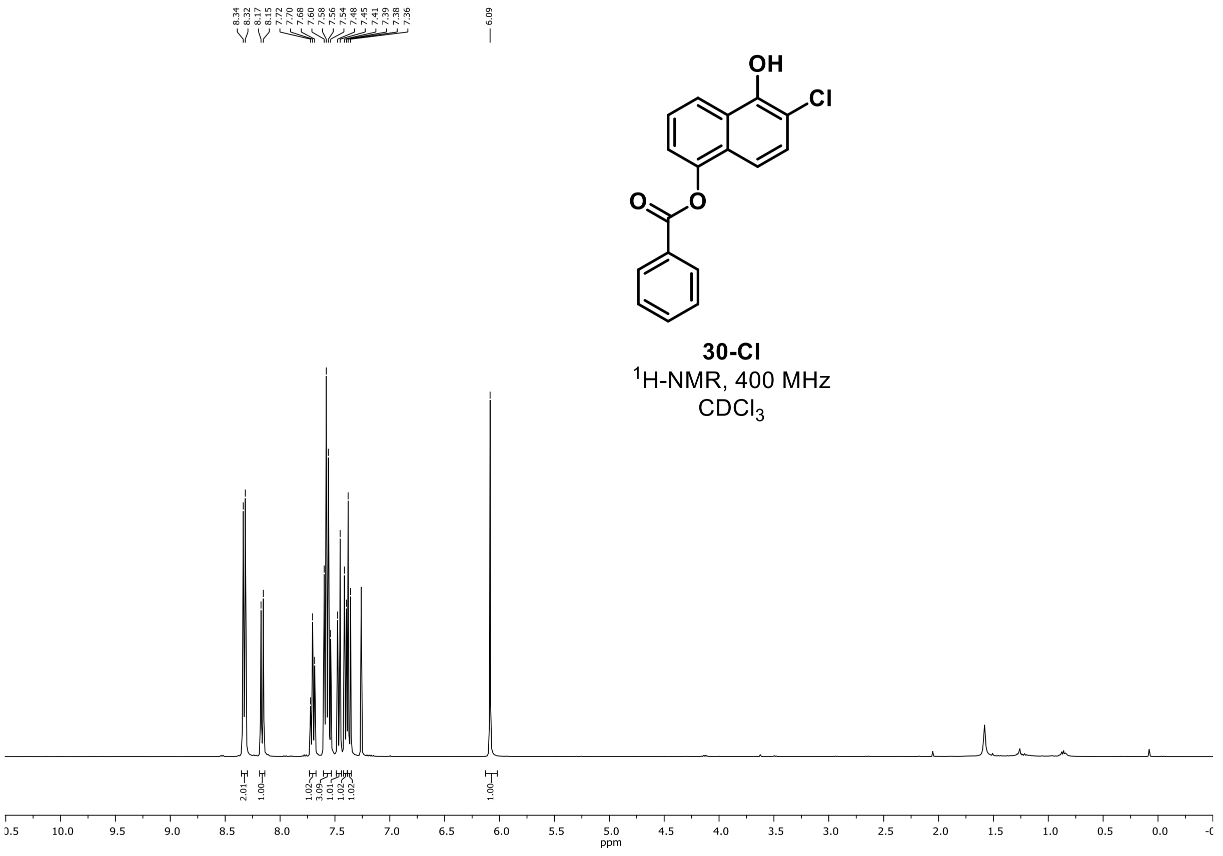

30-Cl
^{1}H-NMR, 400 MHz
$CDCl_3$

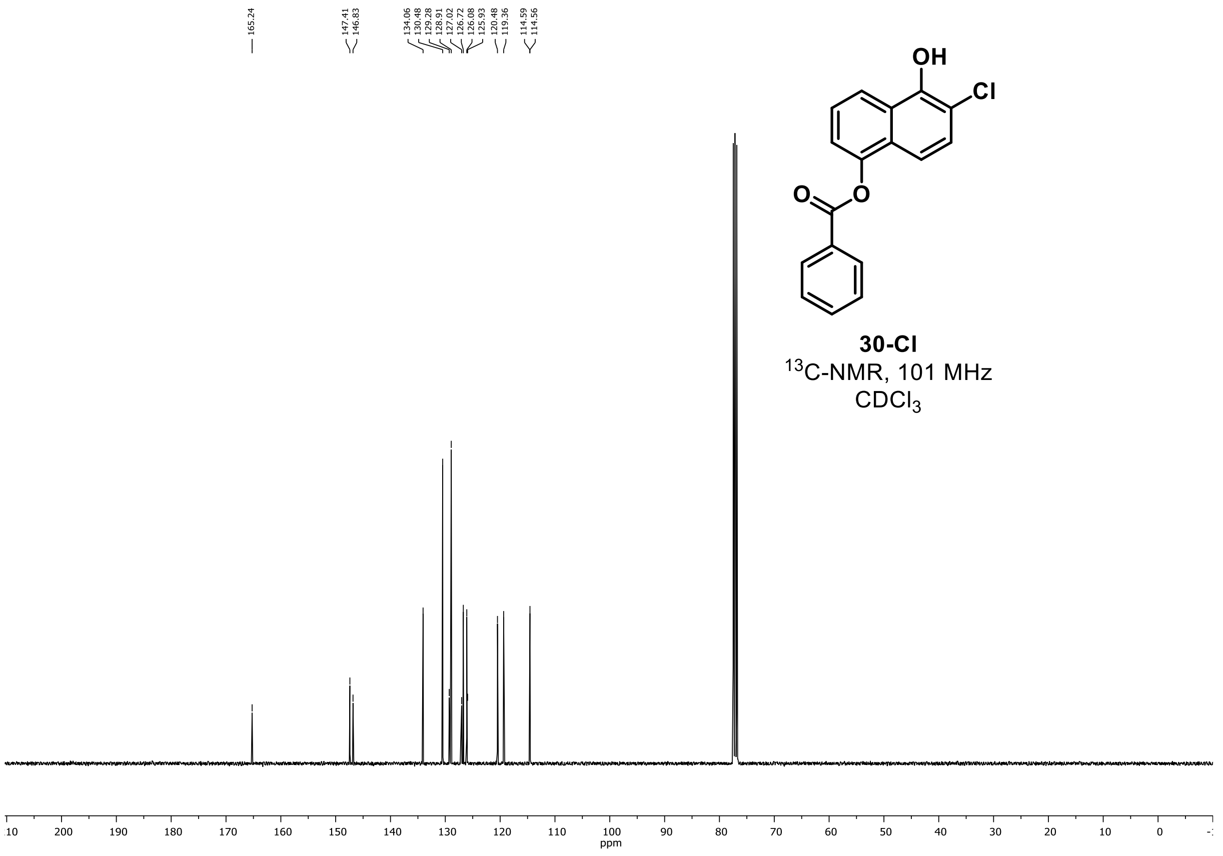

30-Cl
^{13}C-NMR, 101 MHz
$CDCl_3$

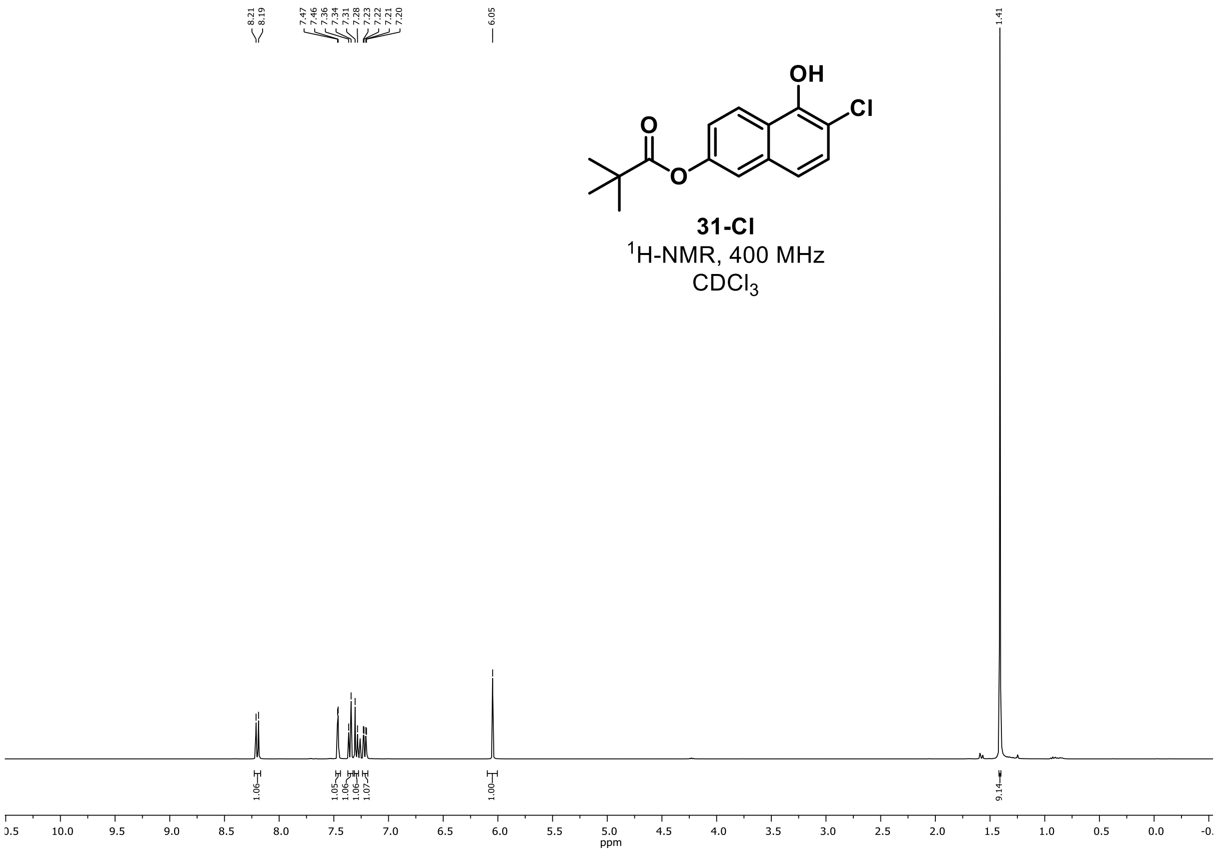
8.21
8.19
7.47
7.46
7.36
7.34
7.31
7.28
7.23
7.22
7.21
7.20
6.05
1.41
OH
Cl
O
O
31-Cl
1H-NMR, 400 MHz
CDCl3
1.06
1.05
1.06
1.06
1.07
1.00
9.14
ppm

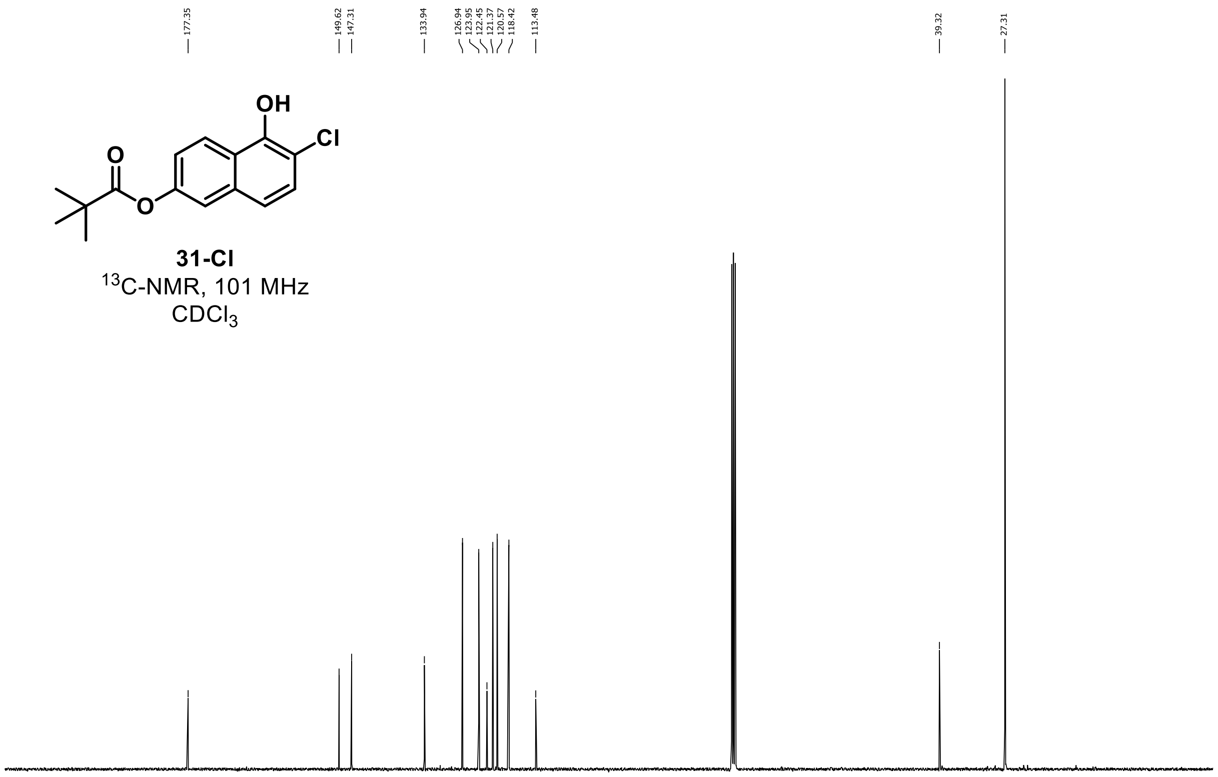
177.35
149.62
147.31
133.94
126.94
123.95
122.45
121.37
120.57
118.42
113.48
39.32
27.31
OH
Cl
O
O
31-Cl
13C-NMR, 101 MHz
CDCl3
ppm

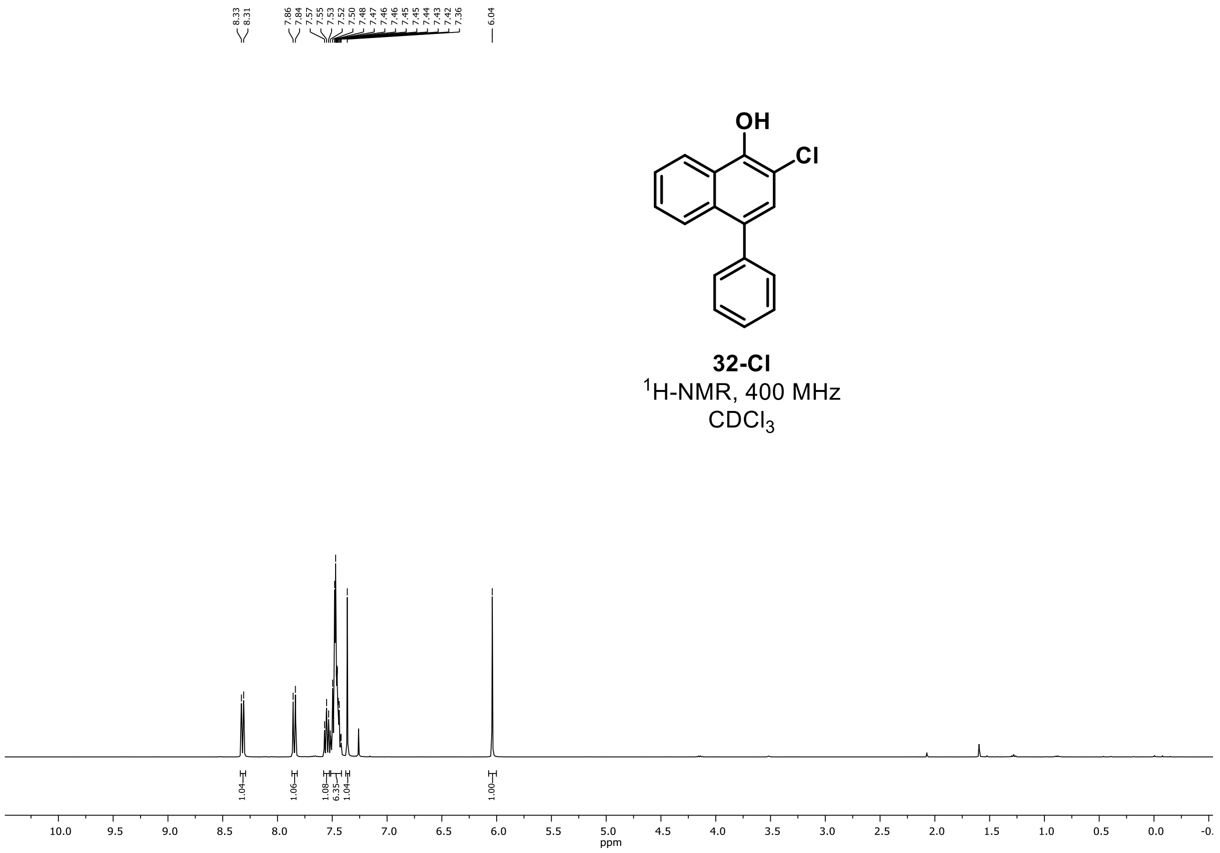
8.33
8.31
7.86
7.84
7.57
7.55
7.53
7.52
7.50
7.48
7.47
7.46
7.46
7.45
7.45
7.44
7.43
7.42
7.36
6.04
OH
Cl
32-Cl
^{1}H-NMR, 400 MHz
$CDCl_3$
1.04
1.06
1.08
6.35
1.04
1.00
ppm

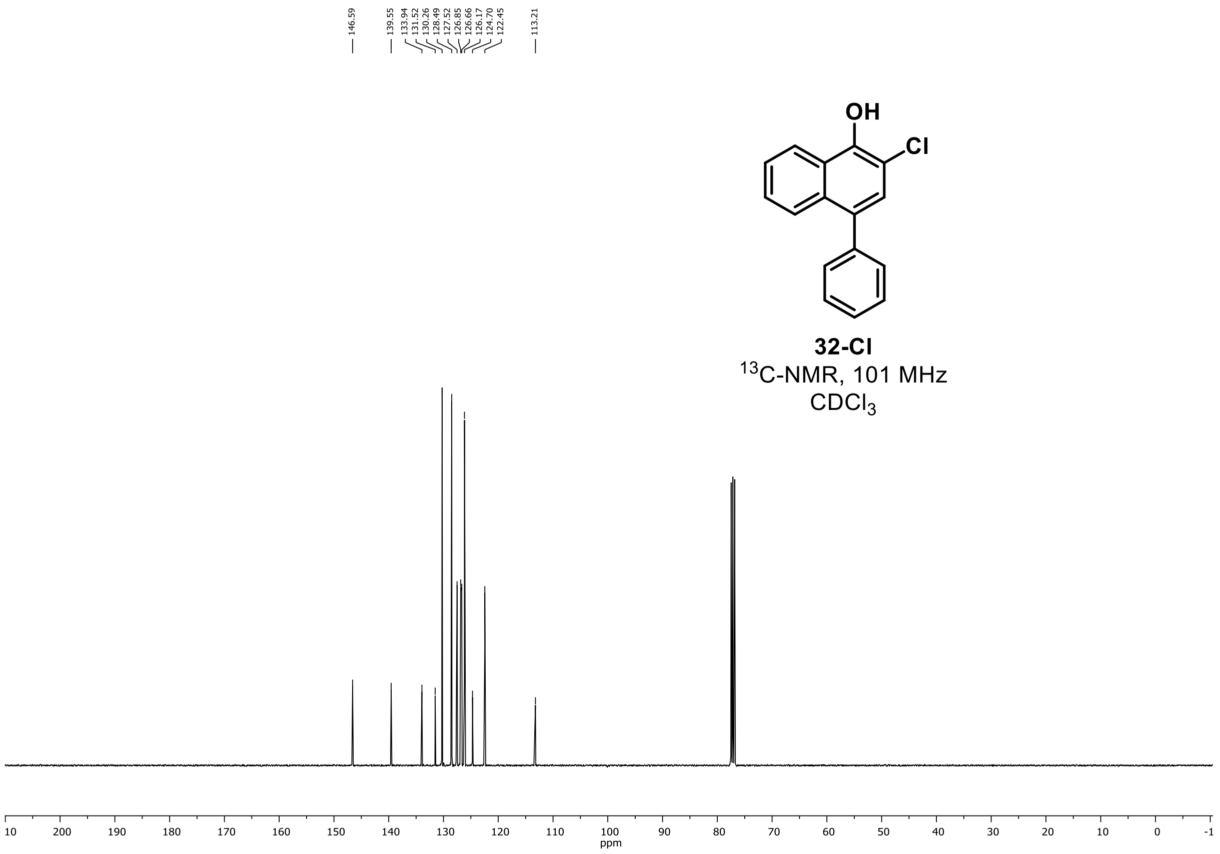
146.59
139.55
133.94
131.52
130.26
128.49
127.52
126.85
126.66
126.17
124.70
122.45
113.21
OH
Cl
32-Cl
^{13}C-NMR, 101 MHz
$CDCl_3$
ppm

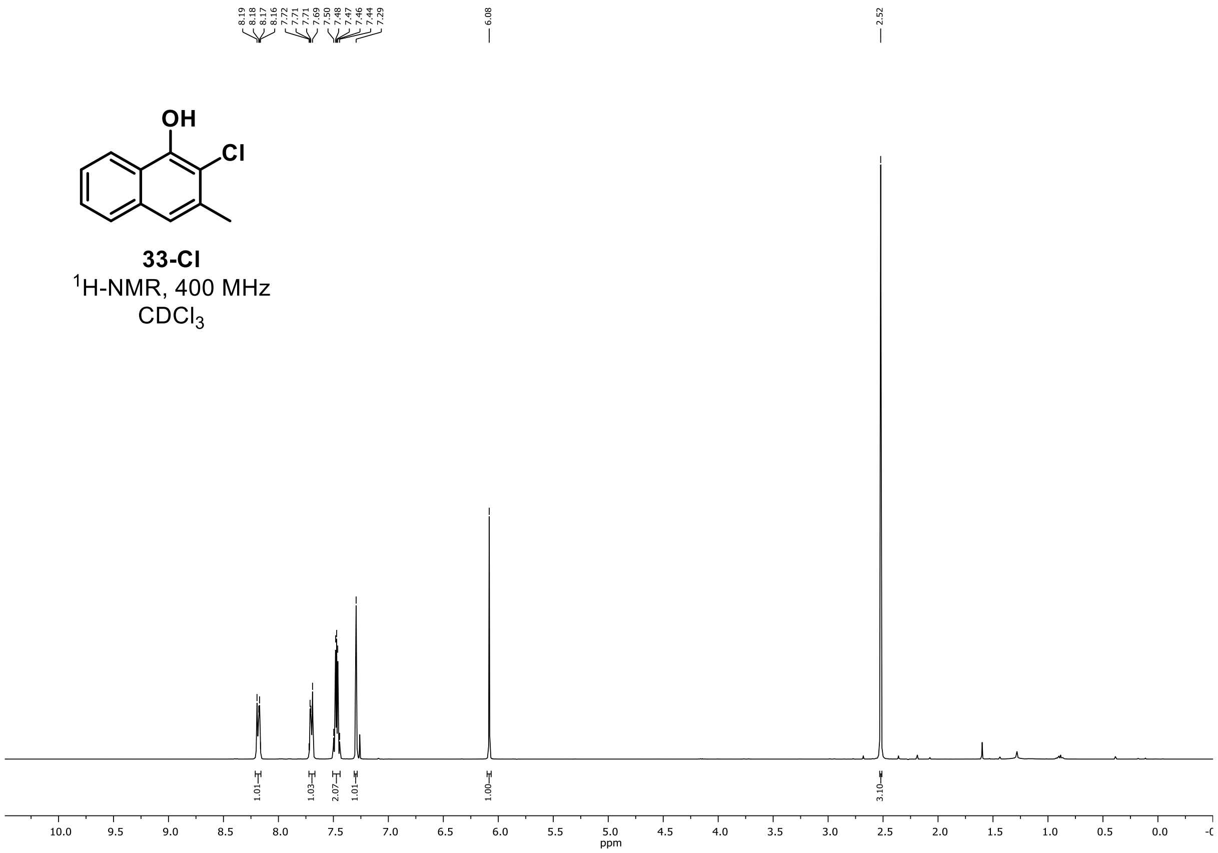
OH
Cl
33-Cl
¹H-NMR, 400 MHz
CDCl3
8.19 8.18 8.17 8.16 7.72 7.71 7.71 7.69 7.50 7.48 7.47 7.46 7.44 7.29
6.08
2.52
1.01 1.03 2.07 1.01 1.00 3.10
10.0 9.5 9.0 8.5 8.0 7.5 7.0 6.5 6.0 5.5 5.0 4.5 4.0 3.5 3.0 2.5 2.0 1.5 1.0 0.5 0.0
ppm

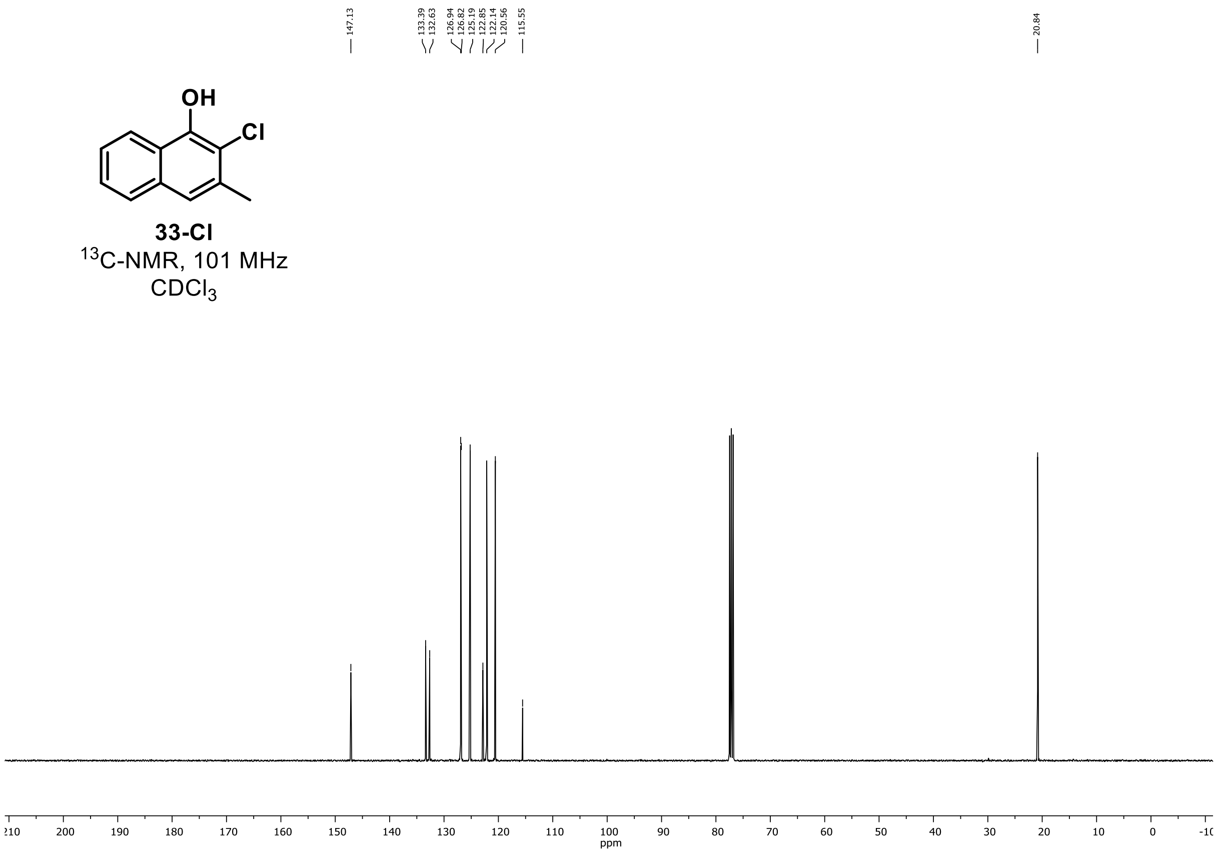
OH
Cl
33-Cl
¹³C-NMR, 101 MHz
CDCl3
147.13
133.39 132.63
126.94 126.82 125.19 122.85 122.14 120.56
115.55
20.84
200 190 180 170 160 150 140 130 120 110 100 90 80 70 60 50 40 30 20 10 0
ppm

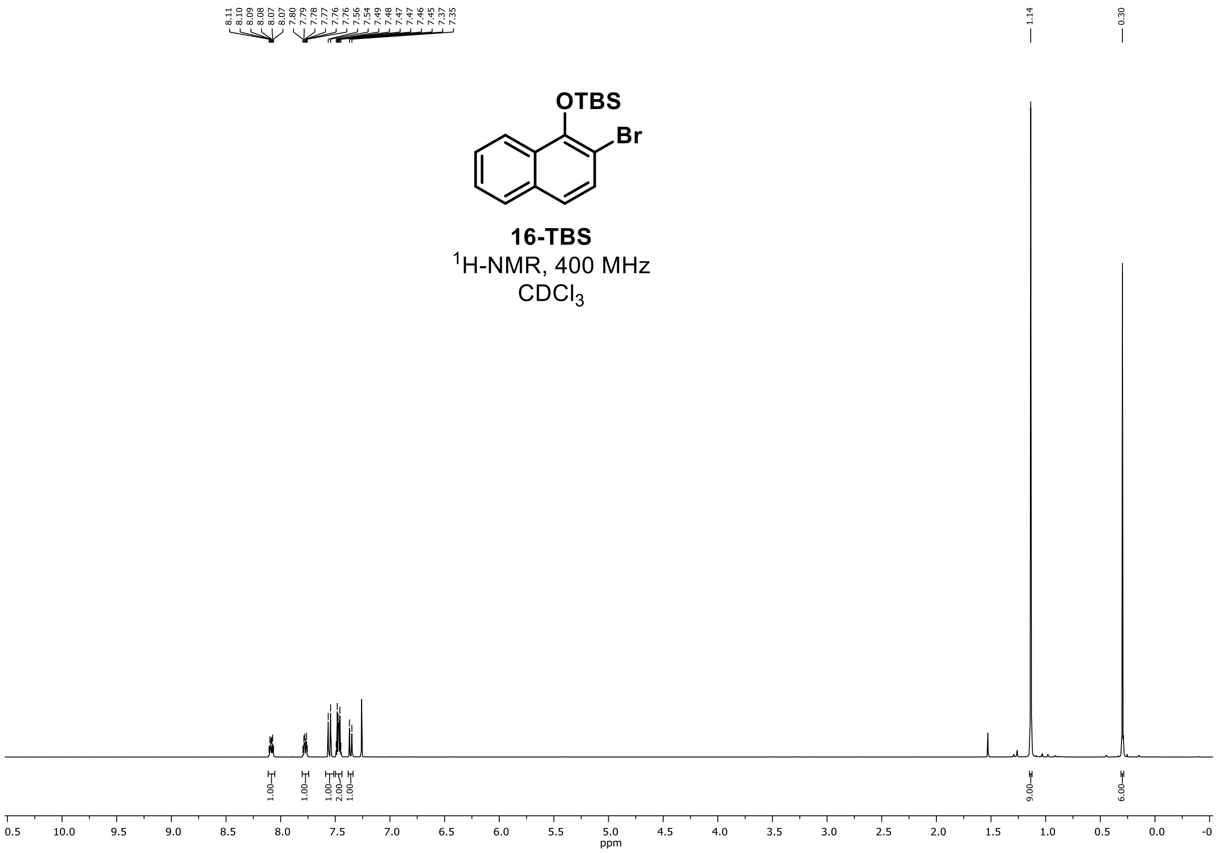
1.14
0.30
OTBS
Br
16-TBS
1H-NMR, 400 MHz
CDCl3
ppm

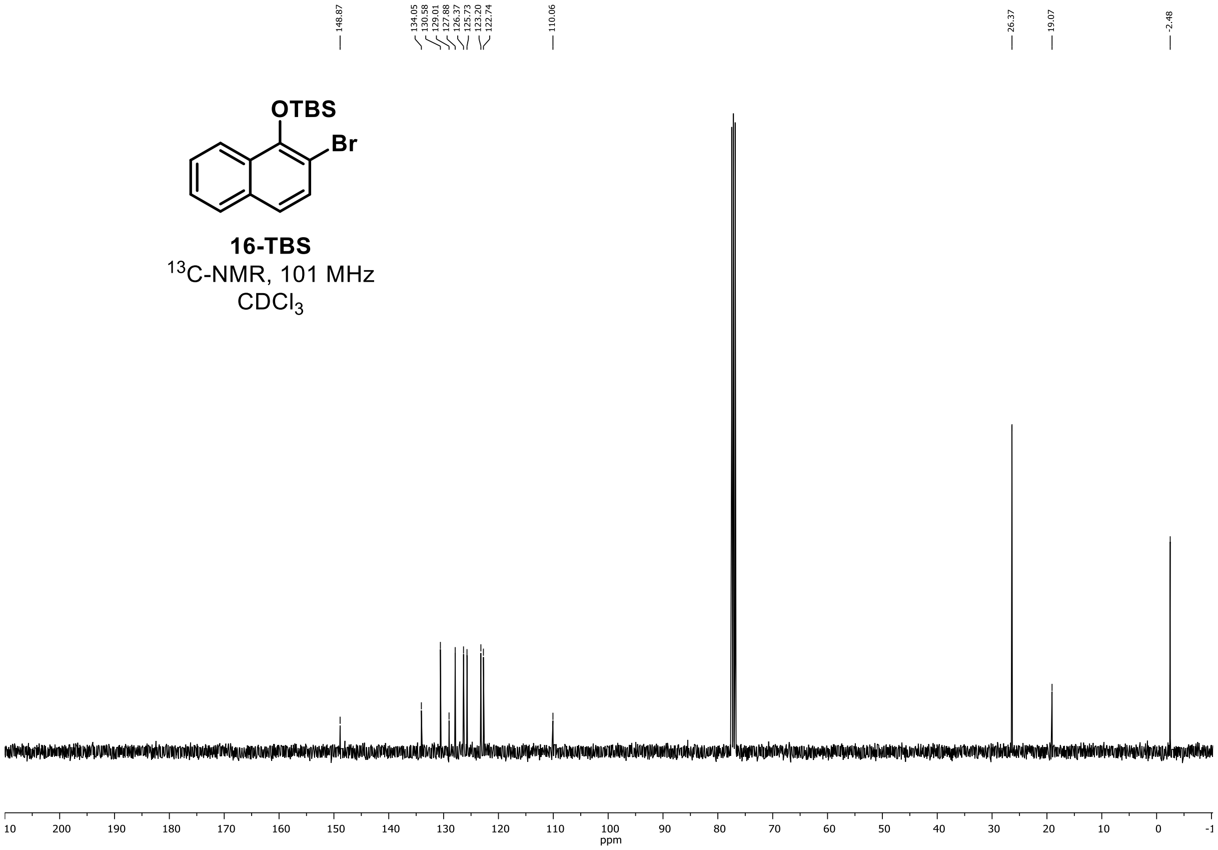
148.87
134.05
130.58
129.01
127.88
126.37
125.73
123.20
122.74
110.06
26.37
19.07
-2.48
OTBS
Br
16-TBS
13C-NMR, 101 MHz
CDCl3
ppm

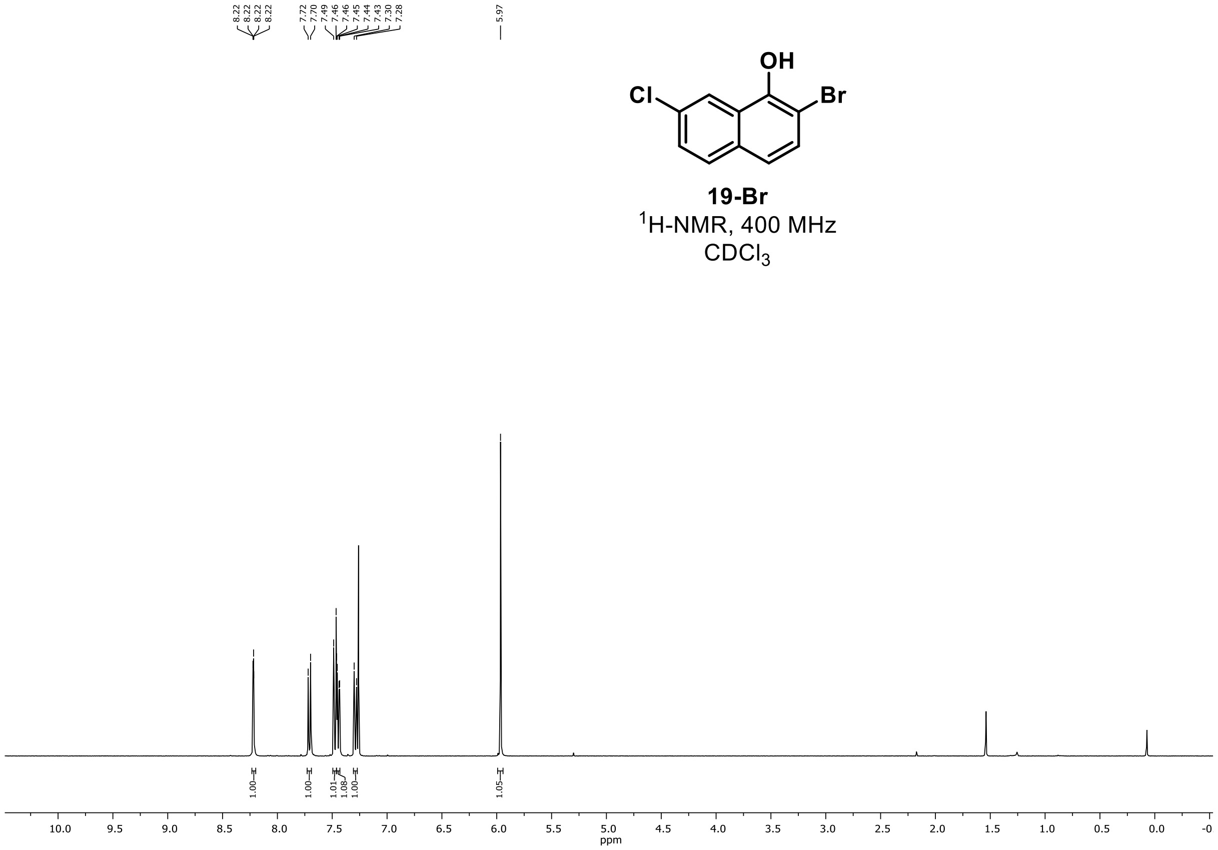

OH
Cl
Br
19-Br
1H-NMR, 400 MHz
CDCl3
ppm

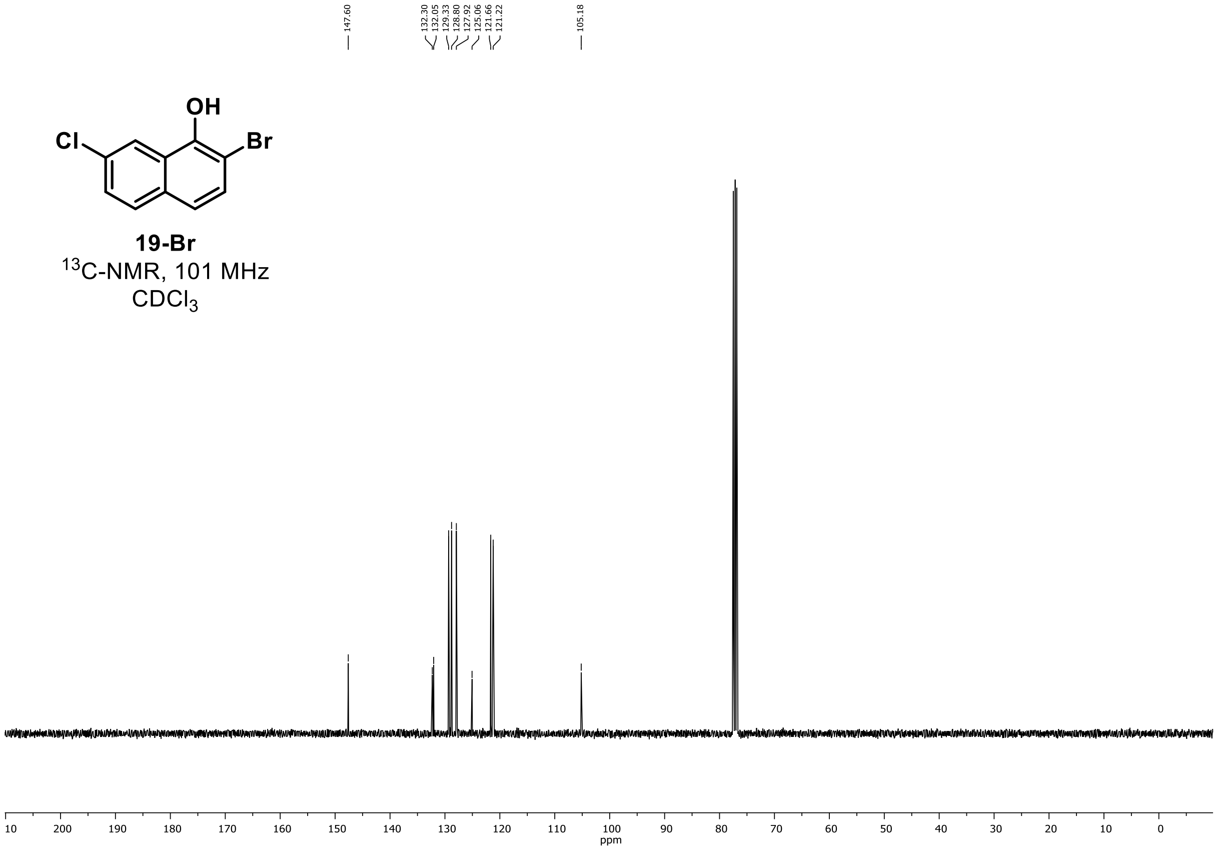

OH
Cl
Br
19-Br
13C-NMR, 101 MHz
CDCl3
ppm

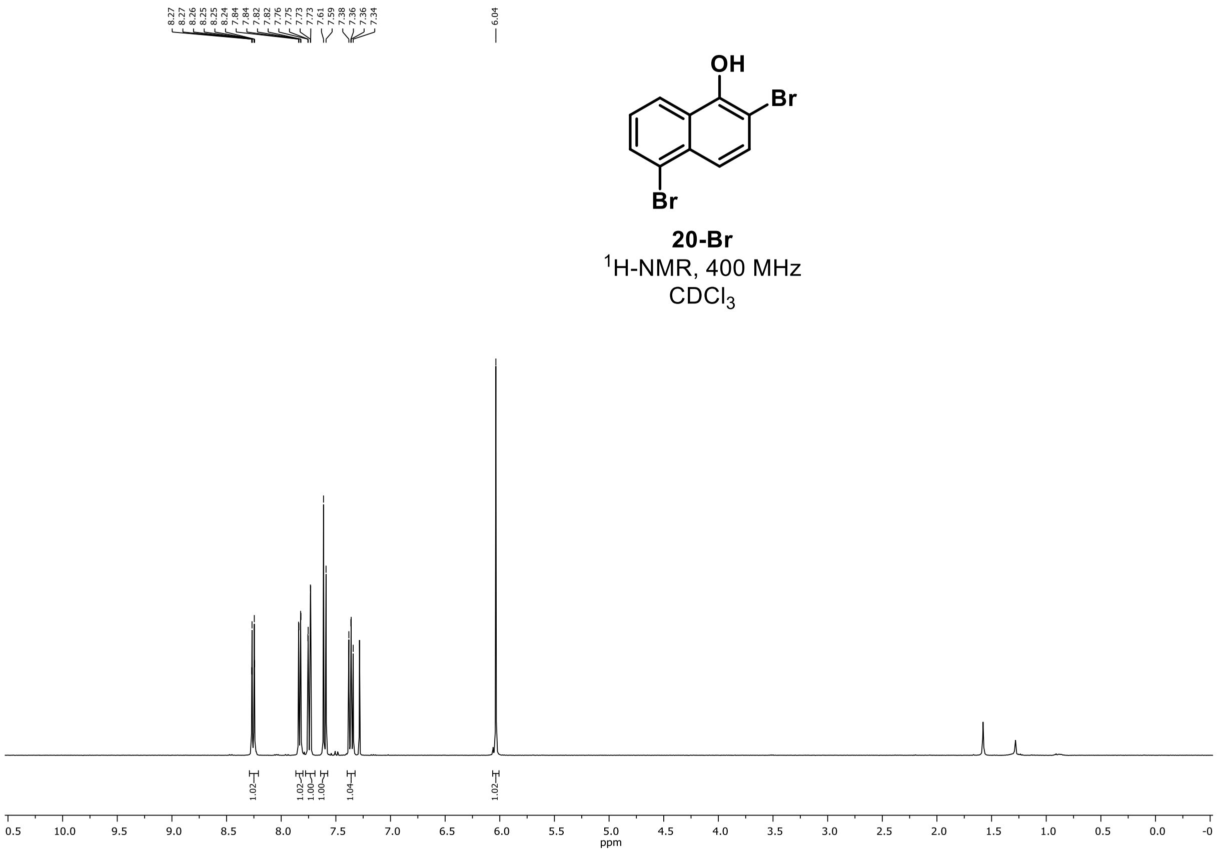
8.27 8.27 8.26 8.25 8.25 8.24 7.84 7.84 7.82 7.82 7.76 7.75 7.73 7.73 7.61 7.59 7.38 7.36 7.36 7.34
6.04
OH
Br
Br
20-Br
^{1}H-NMR, 400 MHz
$CDCl_3$
1.02
1.02
1.00
1.00
1.04
1.02
ppm

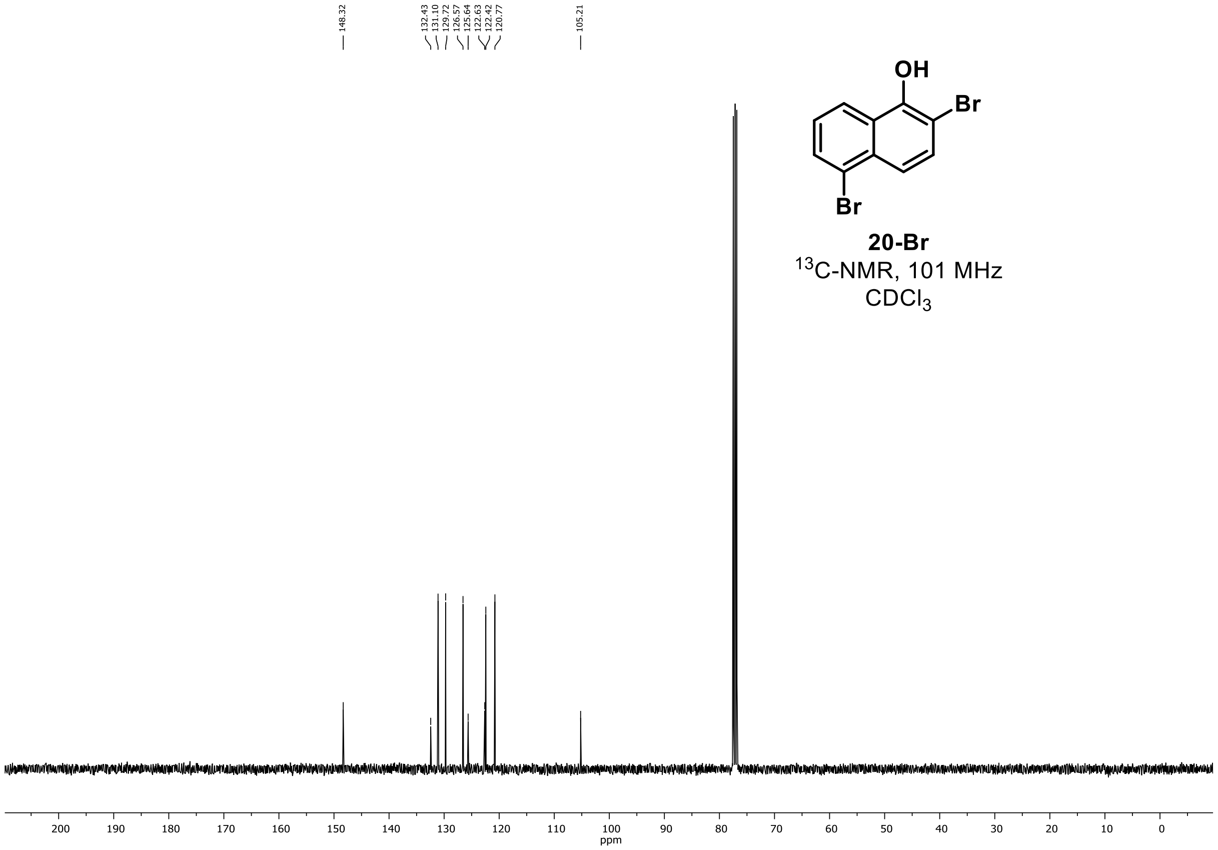
148.32
132.43 131.10 129.72 126.57 125.64 122.63 122.42 120.77
105.21
OH
Br
Br
20-Br
^{13}C-NMR, 101 MHz
$CDCl_3$
ppm

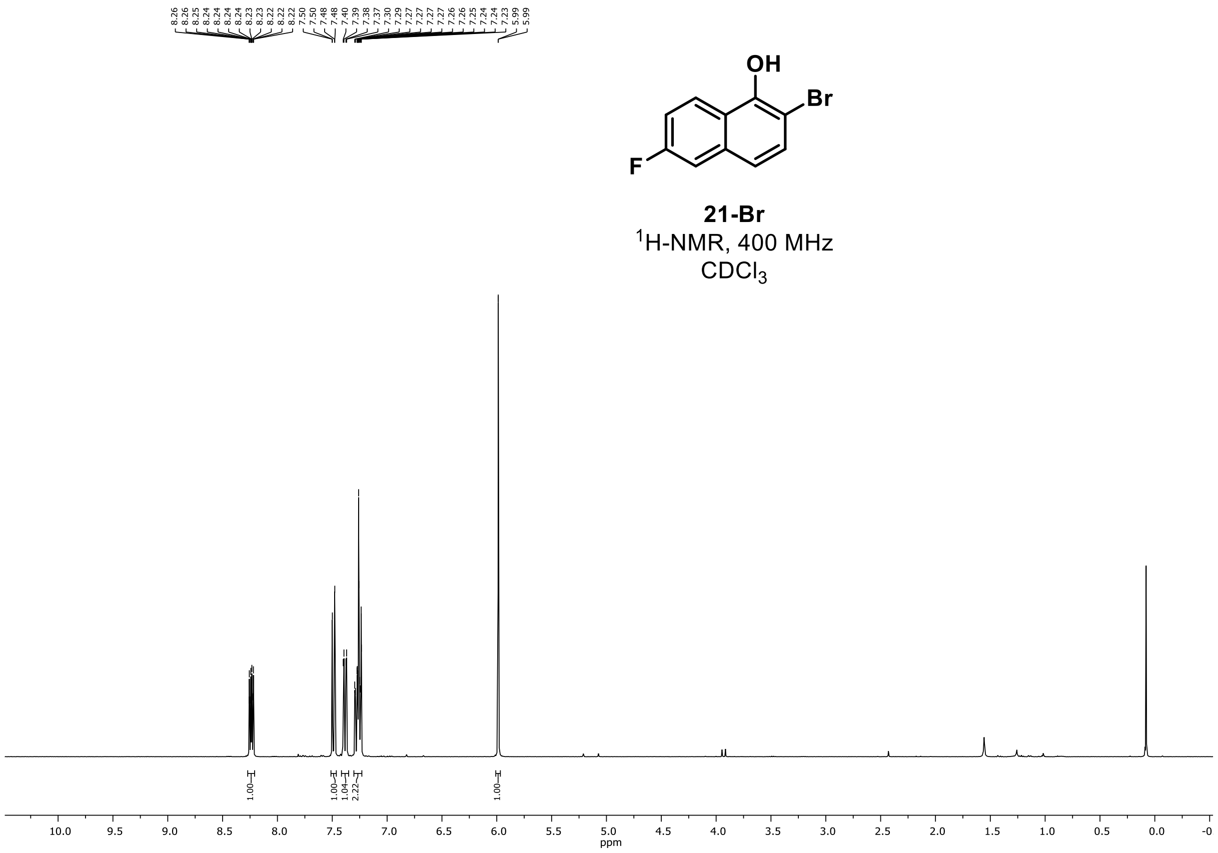

21-Br
^{1}H-NMR, 400 MHz
$CDCl_3$

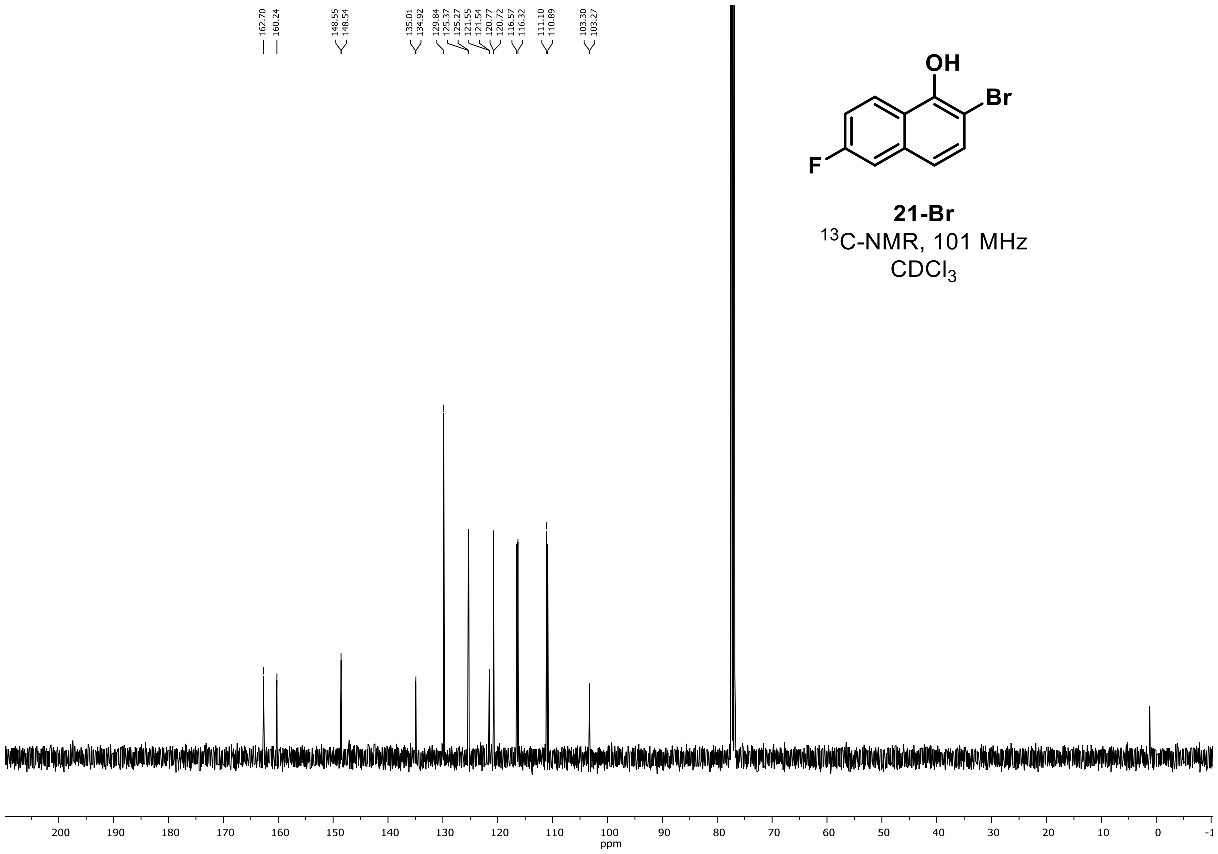

21-Br
^{13}C-NMR, 101 MHz
$CDCl_3$

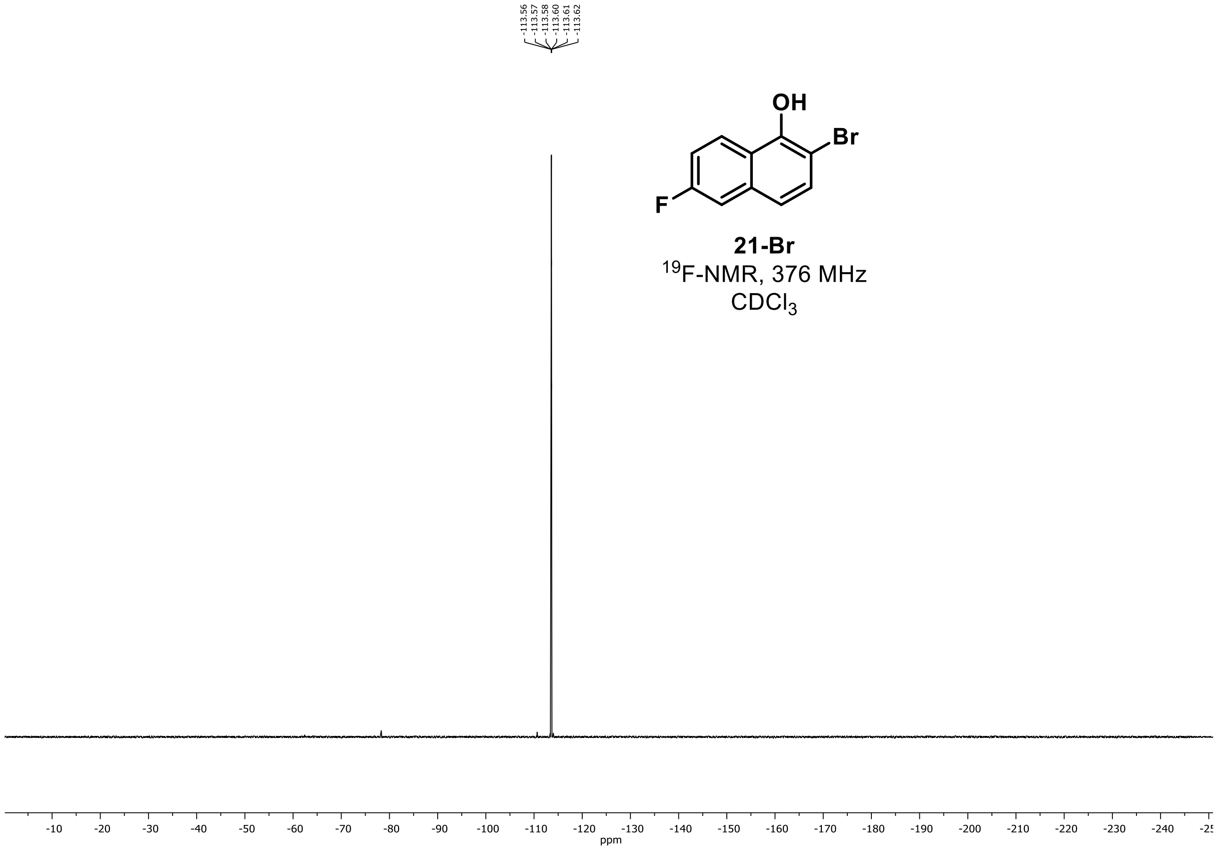
-113.56
-113.57
-113.58
-113.60
-113.61
-113.62
OH
Br
F
21-Br
^{19}F-NMR, 376 MHz
$CDCl_3$
-10 -20 -30 -40 -50 -60 -70 -80 -90 -100 -110 -120 -130 -140 -150 -160 -170 -180 -190 -200 -210 -220 -230 -240
ppm

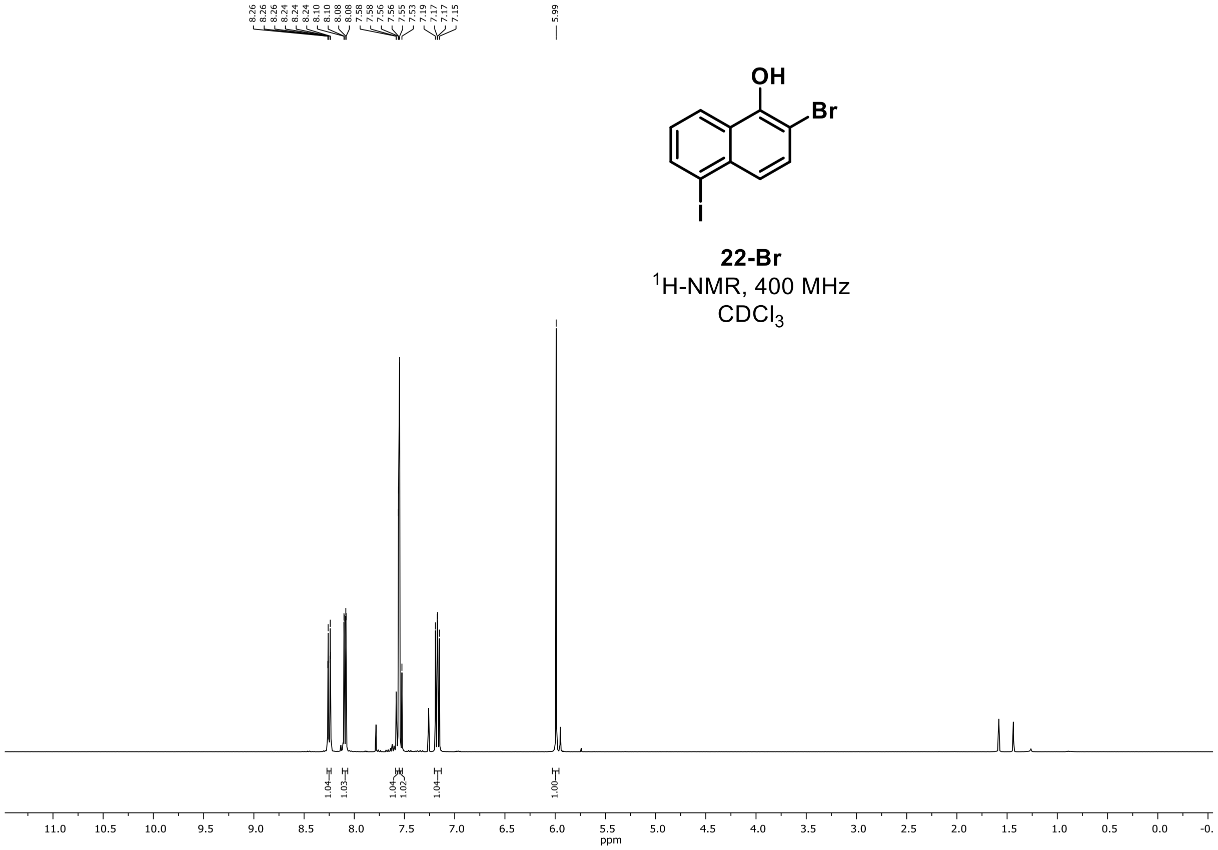
8.26
8.26
8.26
8.24
8.24
8.24
8.10
8.10
8.08
8.08
7.58
7.58
7.56
7.56
7.55
7.53
7.19
7.17
7.17
7.15
5.99
OH
Br
I
22-Br
^{1}H-NMR, 400 MHz
$CDCl_3$
1.04
1.03
1.04
1.02
1.04
1.00
11.0 10.5 10.0 9.5 9.0 8.5 8.0 7.5 7.0 6.5 6.0 5.5 5.0 4.5 4.0 3.5 3.0 2.5 2.0 1.5 1.0 0.5 0.0
ppm

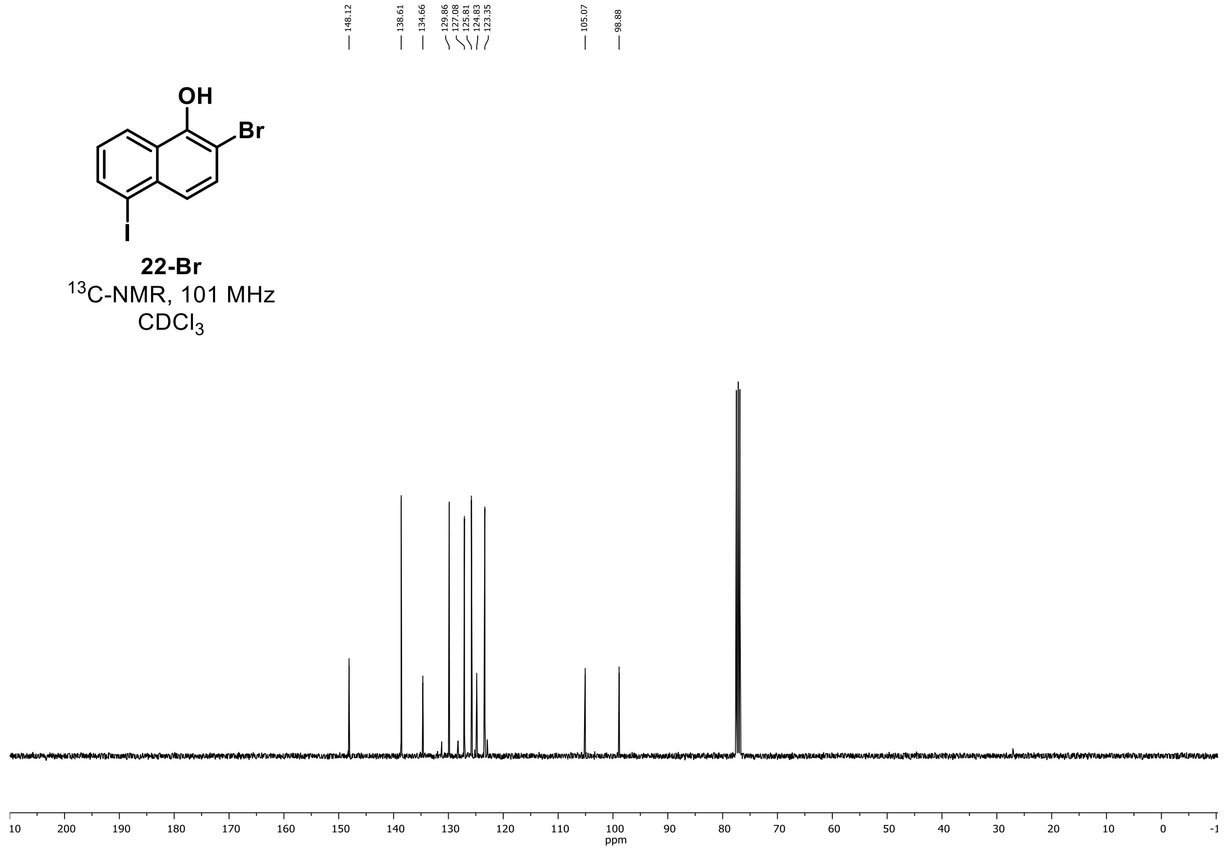

22-Br
^{13}C-NMR, 101 MHz
$CDCl_3$

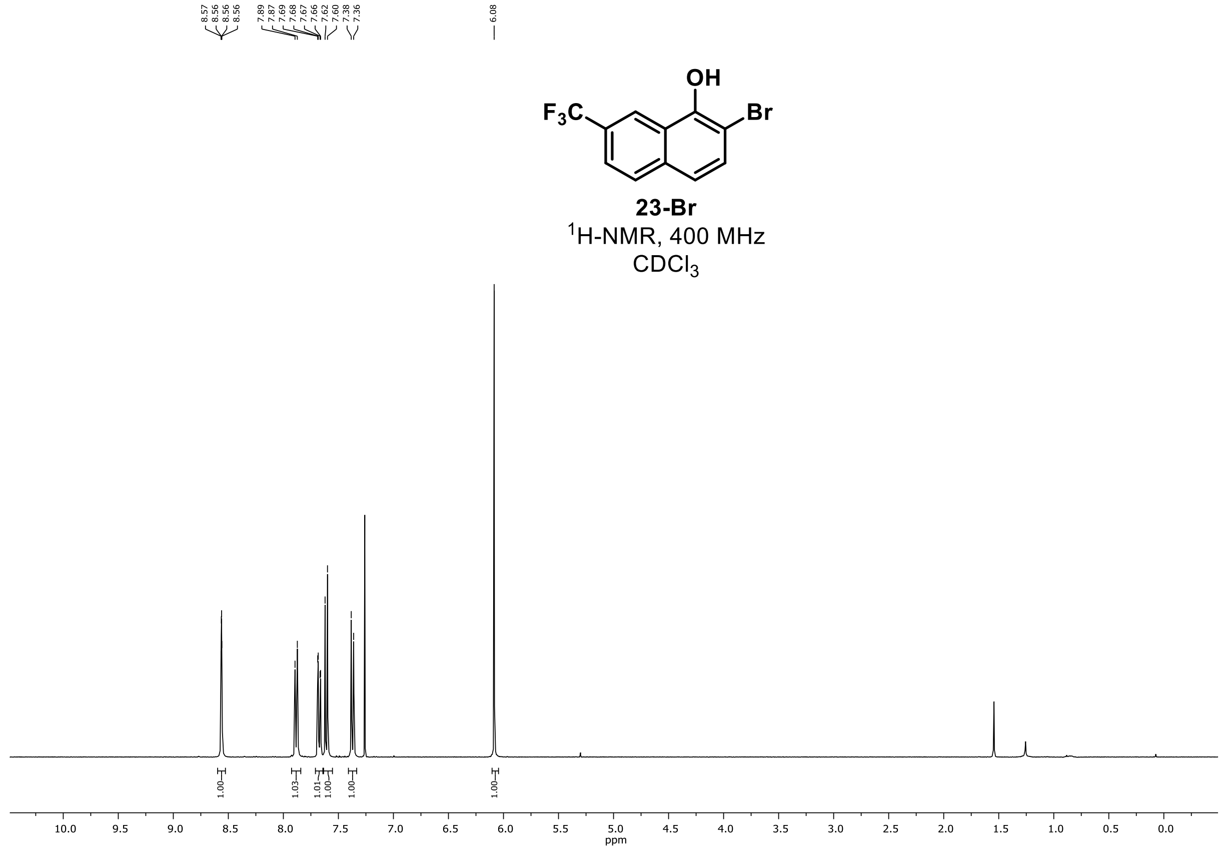

23-Br
^{1}H-NMR, 400 MHz
$CDCl_3$

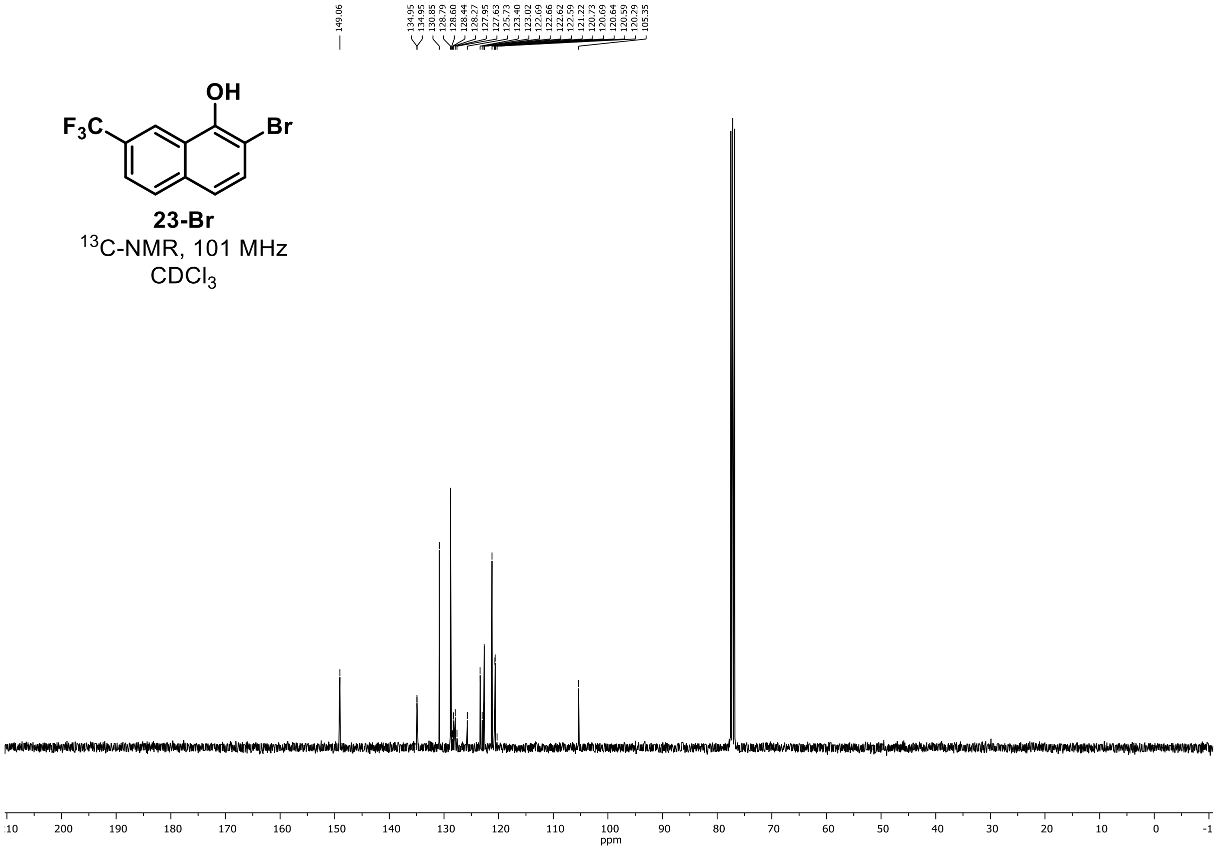
149.06
134.95 134.95 130.85 128.79 128.60 128.44 128.27 127.95 127.63 125.73 123.40 123.02 122.69 122.66 122.62 122.59 121.22 120.73 120.69 120.64 120.59 120.29 105.35
OH
F_3C
Br
23-Br
^{13}C-NMR, 101 MHz
$CDCl_3$
ppm

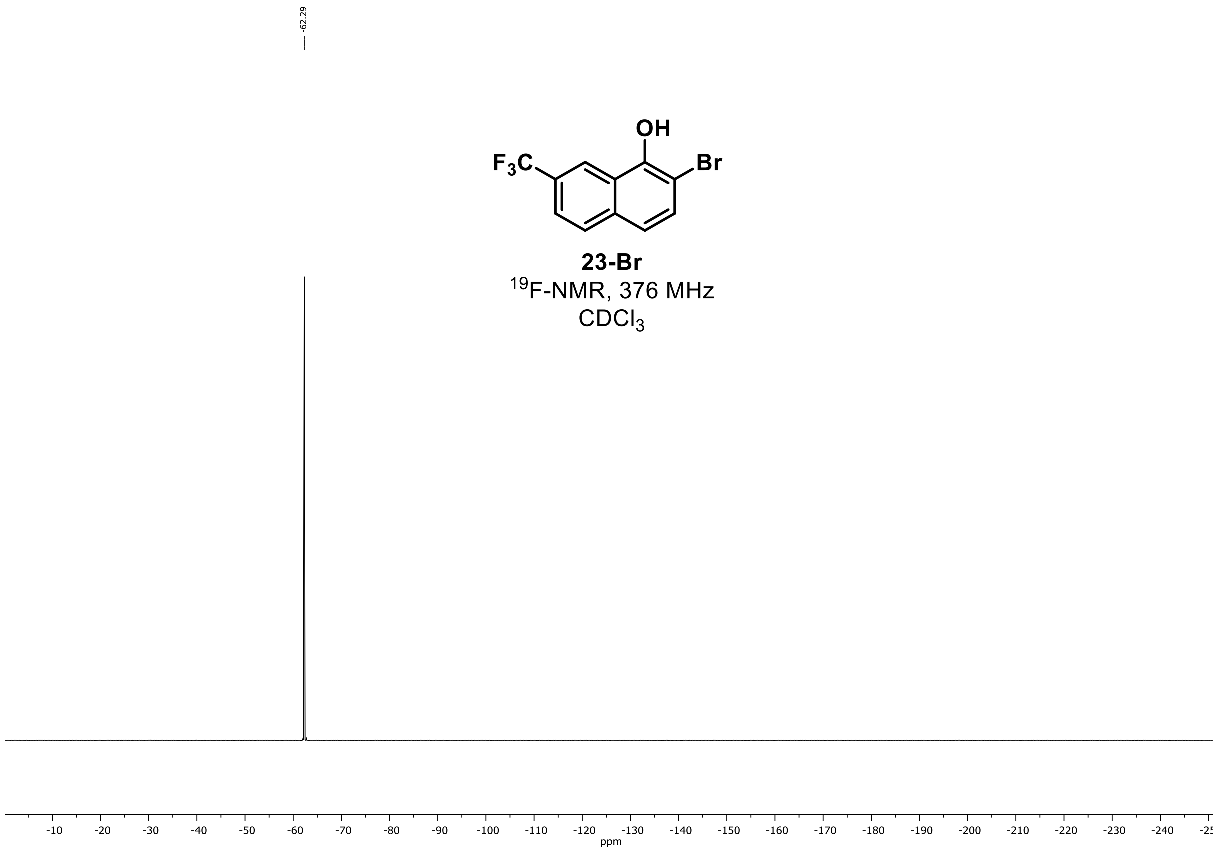
-62.29
OH
F_3C
Br
23-Br
^{19}F-NMR, 376 MHz
$CDCl_3$
ppm

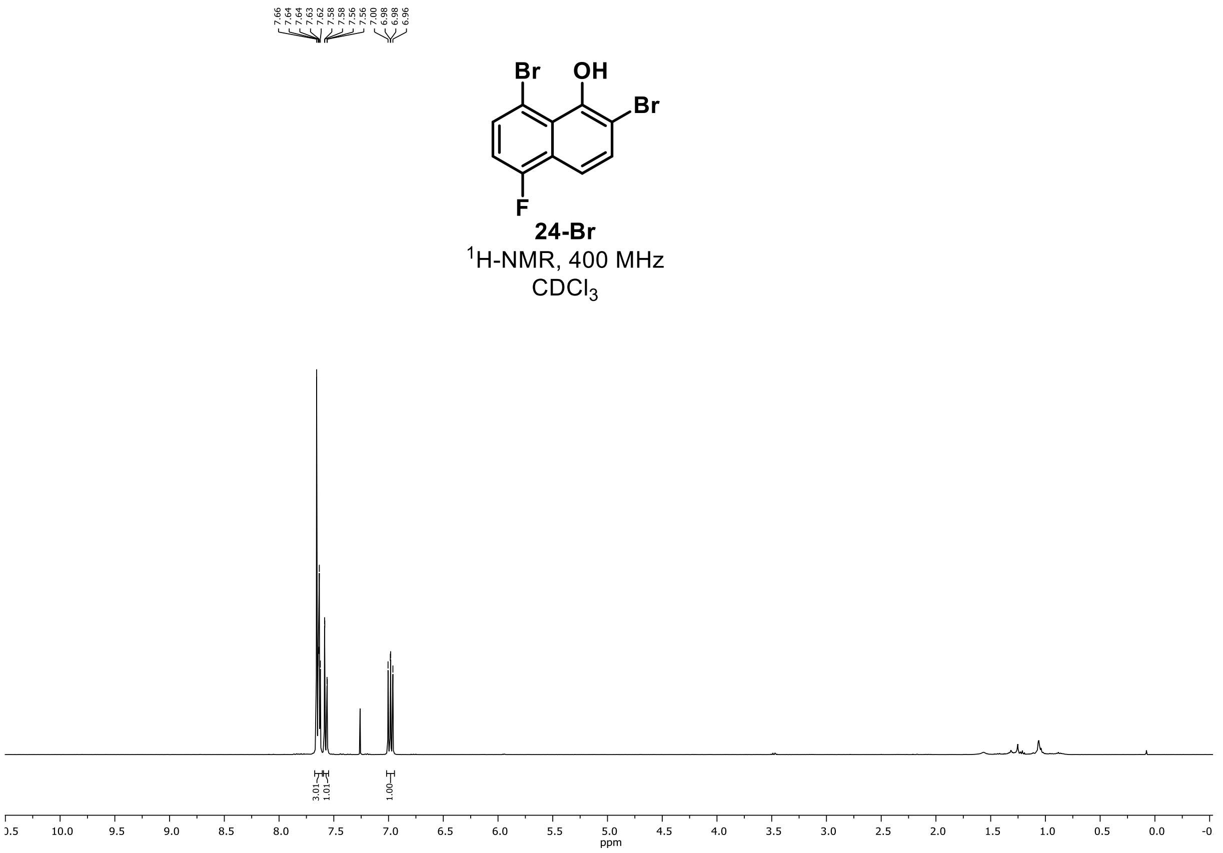
7.66
7.64
7.64
7.63
7.62
7.58
7.58
7.56
7.56
7.00
6.98
6.98
6.96
Br
OH
Br
F
24-Br
^{1}H-NMR, 400 MHz
$CDCl_3$
3.01
1.01
1.00
ppm

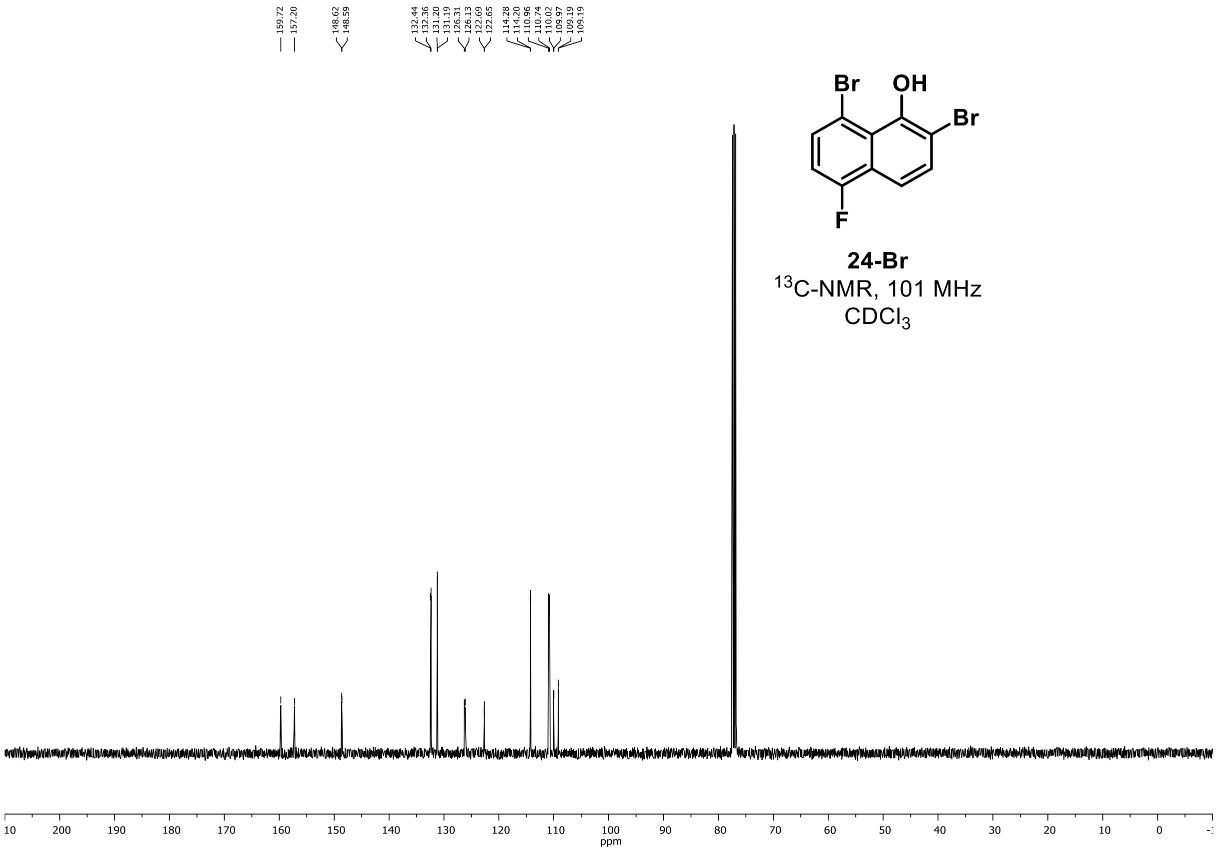
159.72
157.20
148.62
148.59
132.44
132.36
131.20
131.19
126.31
126.13
122.69
122.65
114.28
114.20
110.96
110.74
110.02
109.97
109.19
109.19
Br
OH
Br
F
24-Br
^{13}C-NMR, 101 MHz
$CDCl_3$
ppm

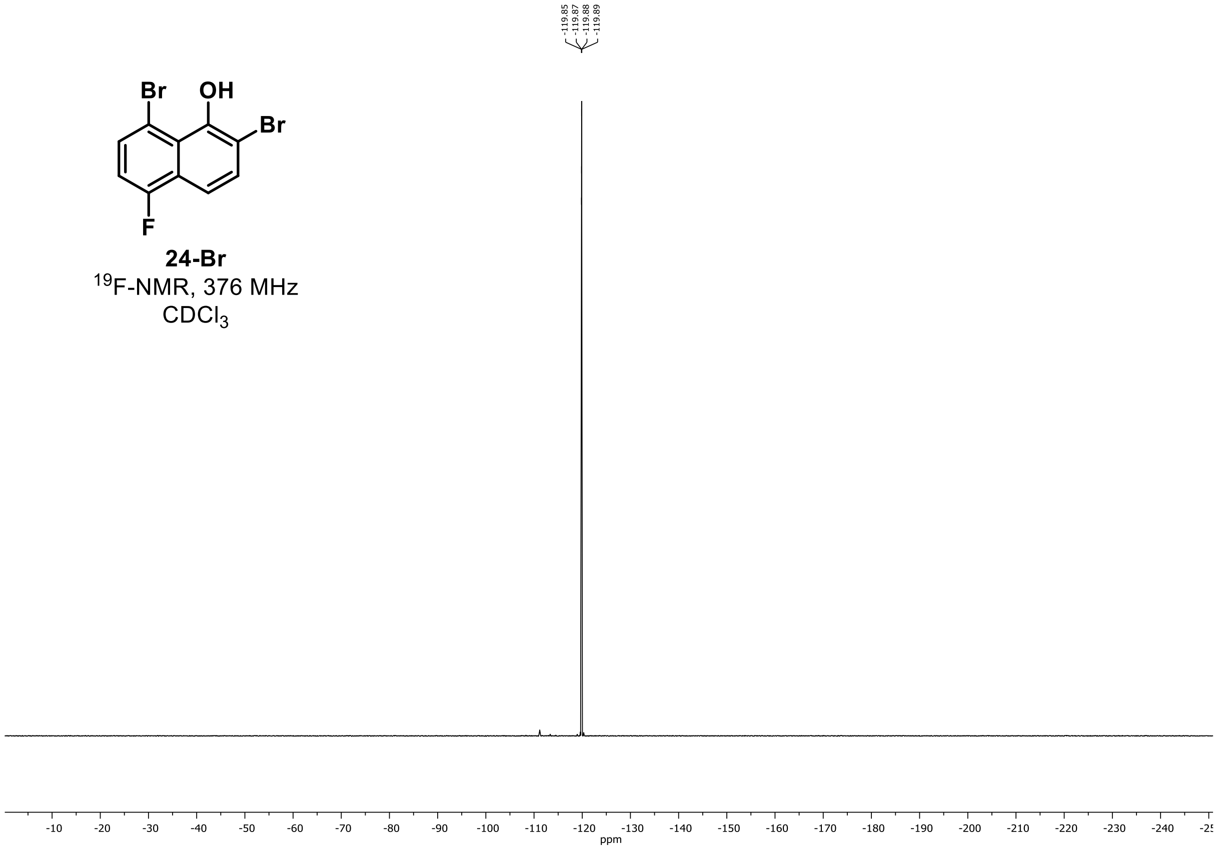
-119.85
-119.87
-119.88
-119.89
Br
OH
Br
F
24-Br
19F-NMR, 376 MHz
CDCl3
-10 -20 -30 -40 -50 -60 -70 -80 -90 -100 -110 -120 -130 -140 -150 -160 -170 -180 -190 -200 -210 -220 -230 -240
ppm

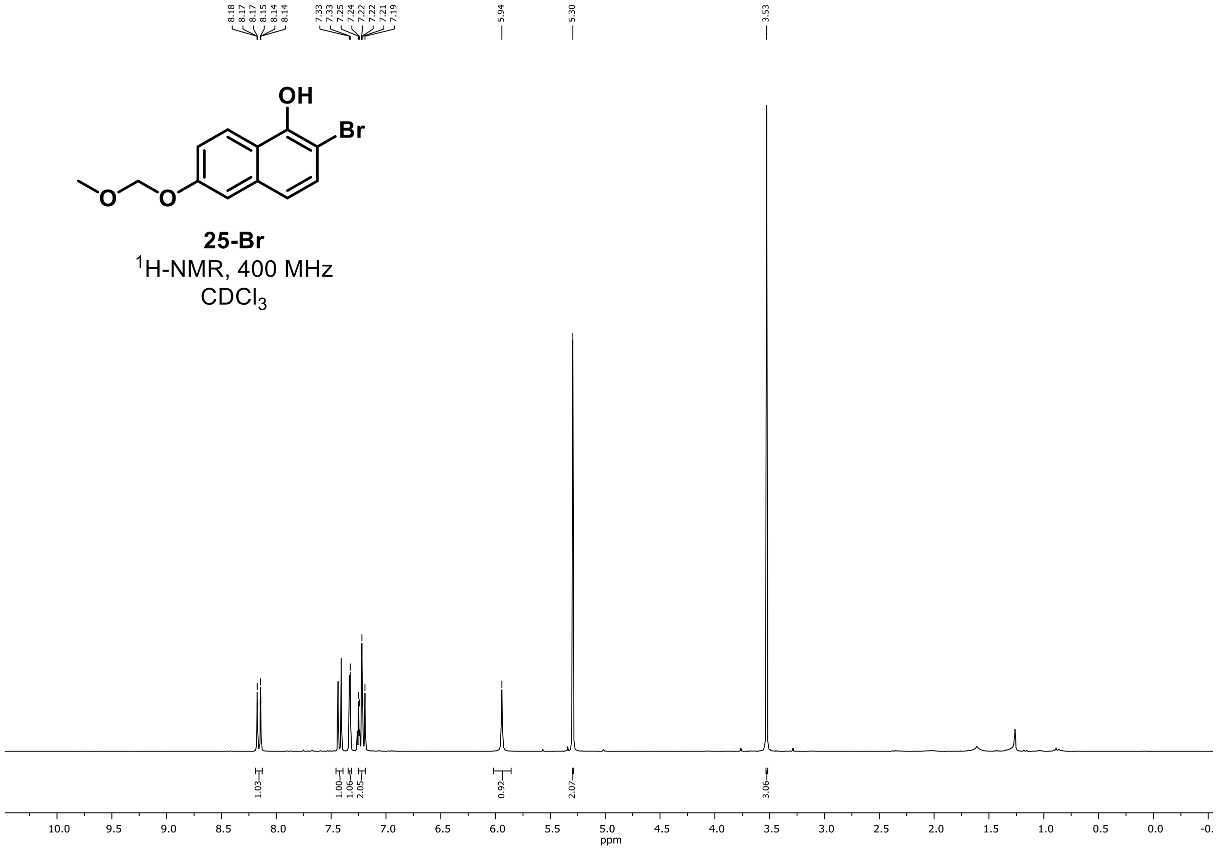
8.18
8.17
8.17
8.15
8.14
8.14
7.33
7.33
7.25
7.24
7.22
7.22
7.21
7.19
5.94
5.30
3.53
OH
Br
O
O
25-Br
1H-NMR, 400 MHz
CDCl3
1.03
1.00
1.06
2.05
0.92
2.07
3.06
10.0 9.5 9.0 8.5 8.0 7.5 7.0 6.5 6.0 5.5 5.0 4.5 4.0 3.5 3.0 2.5 2.0 1.5 1.0 0.5 0.0
ppm

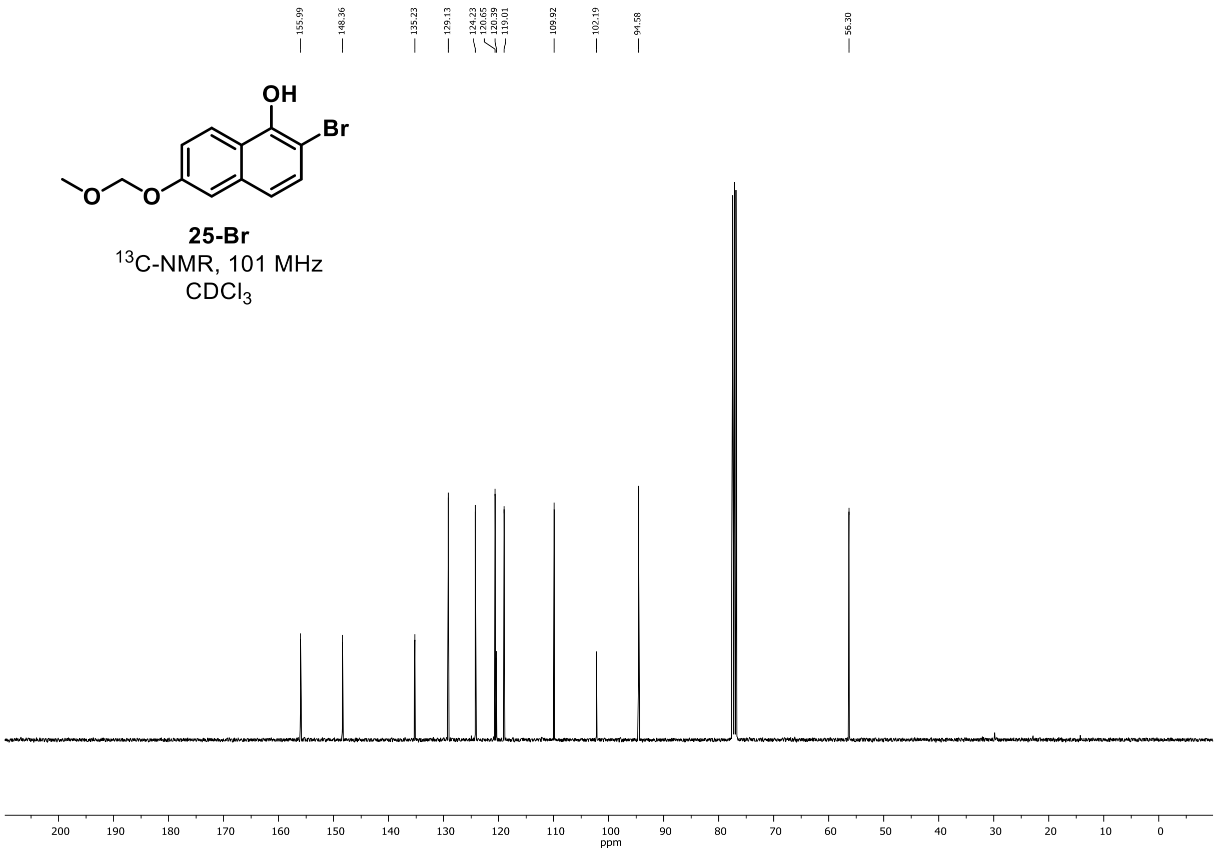
155.99
148.36
135.23
129.13
124.23
120.65
120.39
119.01
109.92
102.19
94.58
56.30
OH
Br
O
O
25-Br
^{13}C-NMR, 101 MHz
$CDCl_3$
ppm

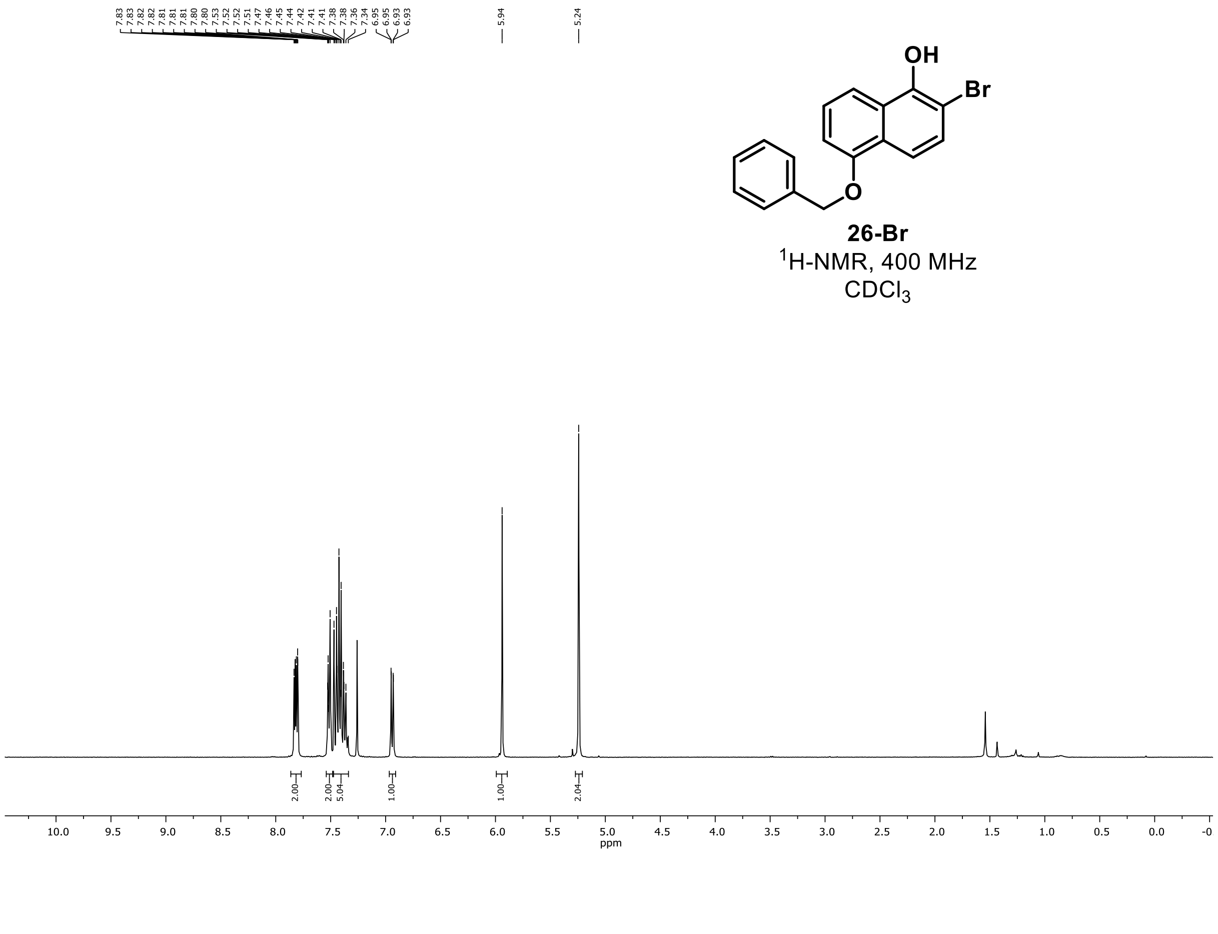
5.94
5.24
OH
Br
O
26-Br
^{1}H-NMR, 400 MHz
$CDCl_3$
2.00
2.00
5.04
1.00
1.00
2.04
ppm

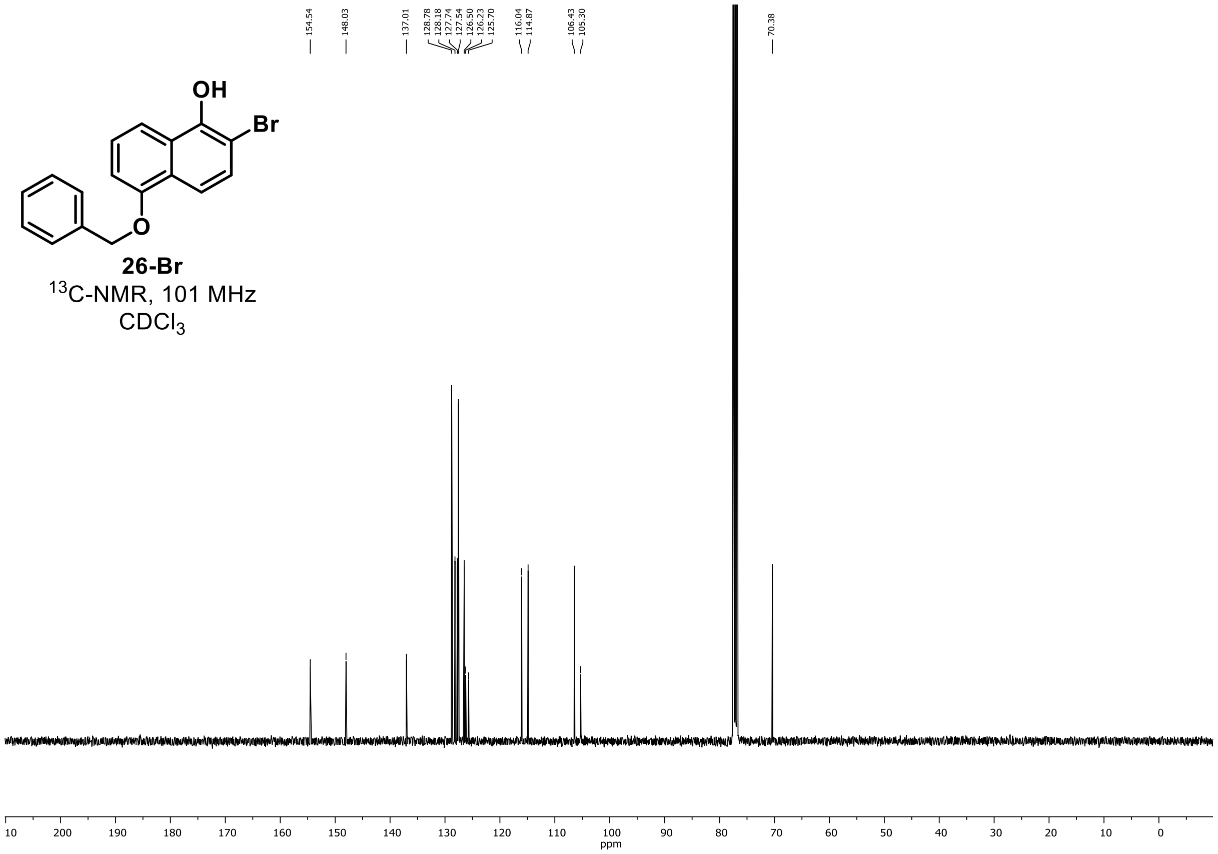
154.54
148.03
137.01
128.78
128.18
127.74
127.54
126.50
126.23
125.70
116.04
114.87
106.43
105.30
70.38
OH
Br
O
26-Br
^{13}C-NMR, 101 MHz
$CDCl_3$
ppm

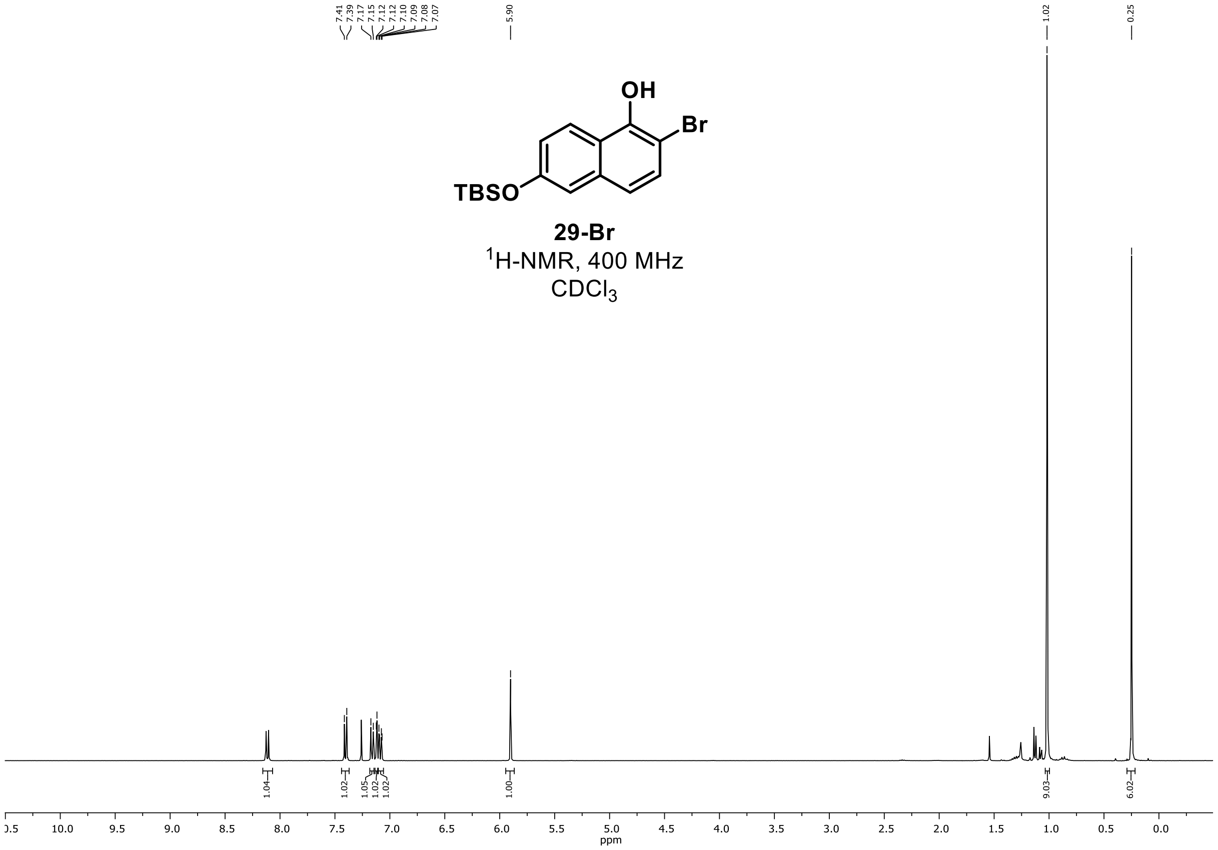
7.41
7.39
7.17
7.15
7.12
7.12
7.10
7.09
7.08
7.07
5.90
1.02
0.25
OH
Br
TBSO
29-Br
^{1}H-NMR, 400 MHz
$CDCl_3$
1.04
1.02
1.05
1.02
1.02
1.00
9.03
6.02
ppm

29-Br
^{13}C-NMR, 101 MHz
$CDCl_3$

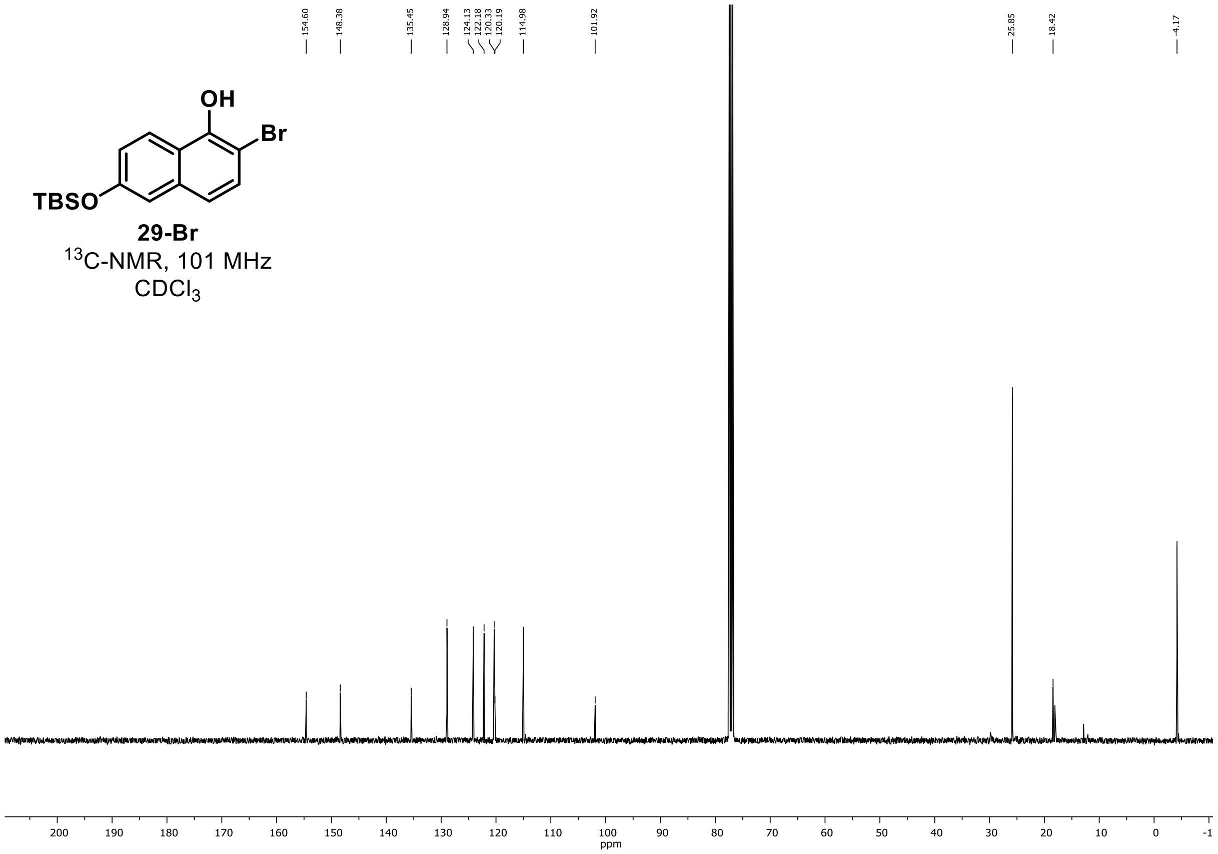

S1-Br
^{1}H-NMR, 400 MHz
$CDCl_3$

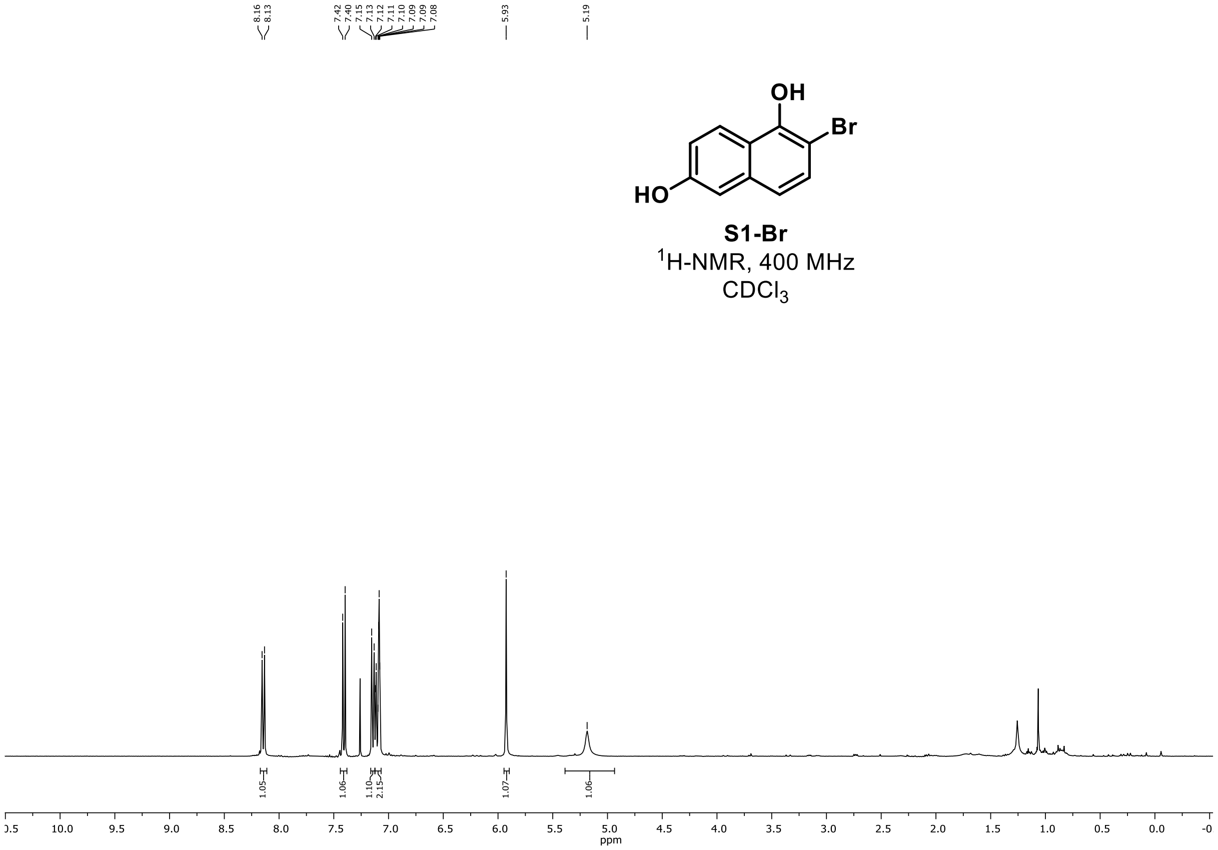

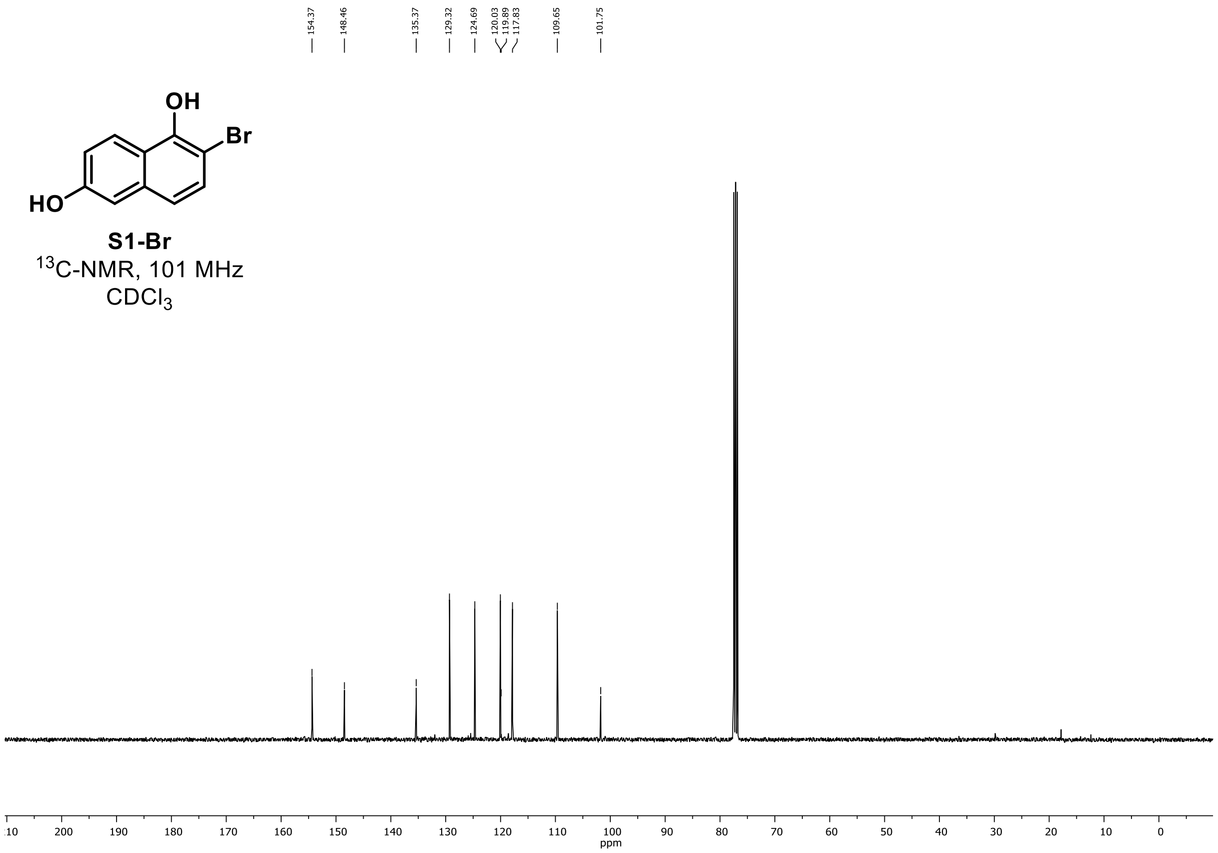
154.37
148.46
135.37
129.32
124.69
120.03
119.89
117.83
109.65
101.75
OH
Br
HO
S1-Br
^{13}C-NMR, 101 MHz
CDCl$_3$
ppm

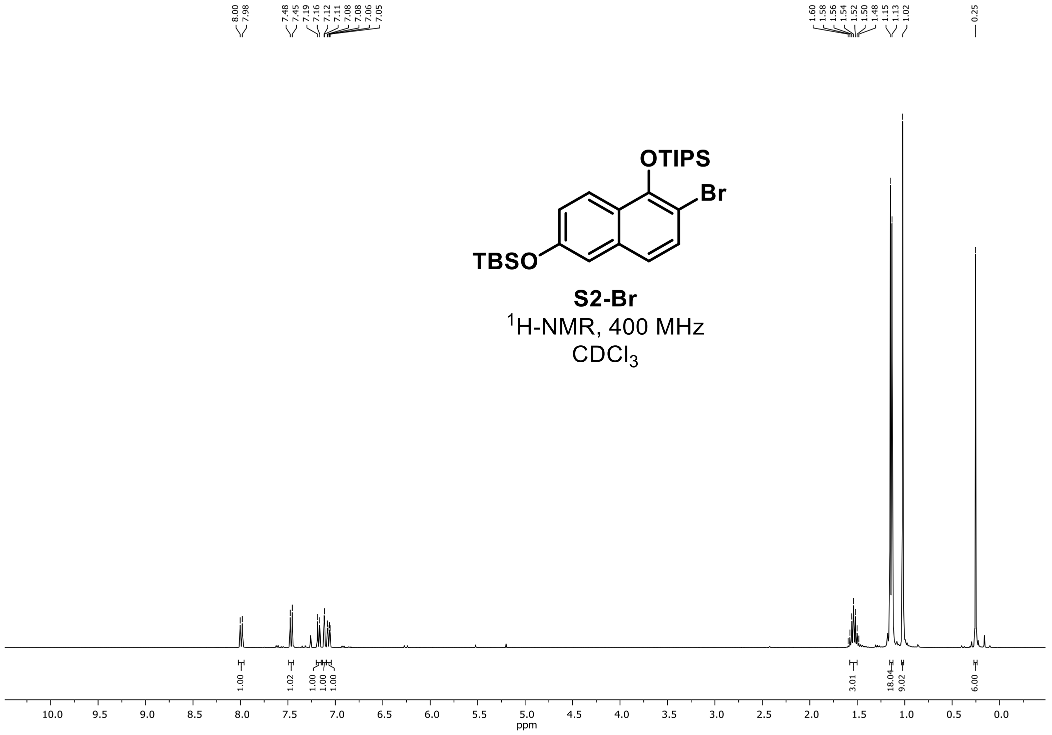
8.00
7.98
7.48
7.45
7.19
7.16
7.12
7.11
7.08
7.06
7.05
1.60
1.58
1.56
1.54
1.52
1.50
1.48
1.15
1.13
1.02
0.25
OTIPS
Br
TBSO
S2-Br
^{1}H-NMR, 400 MHz
CDCl$_3$
1.00
1.02
1.00
1.00
1.00
3.01
18.04
9.02
6.00
ppm

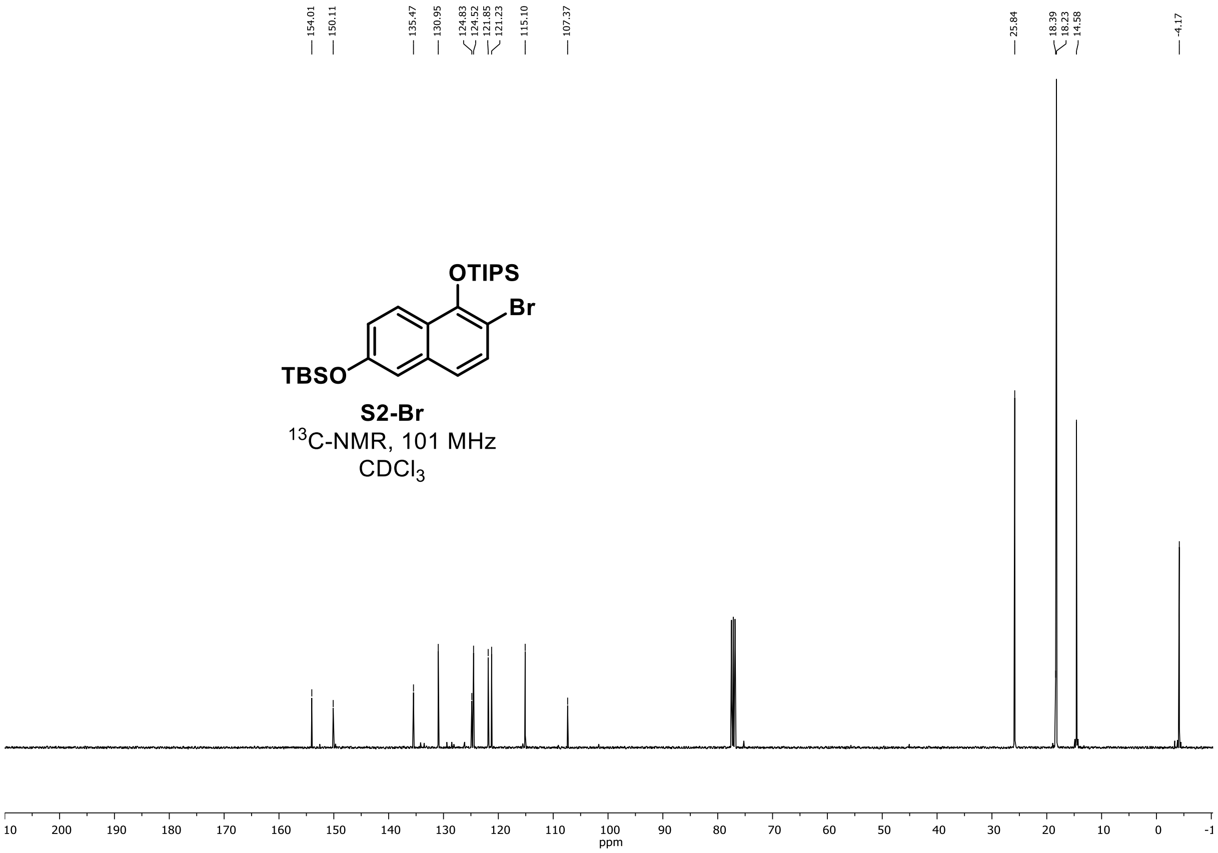
154.01
150.11
135.47
130.95
124.83
124.52
121.85
121.23
115.10
107.37
25.84
18.39
18.23
14.58
-4.17
OTIPS
Br
TBSO
S2-Br
13C-NMR, 101 MHz
CDCl3
ppm

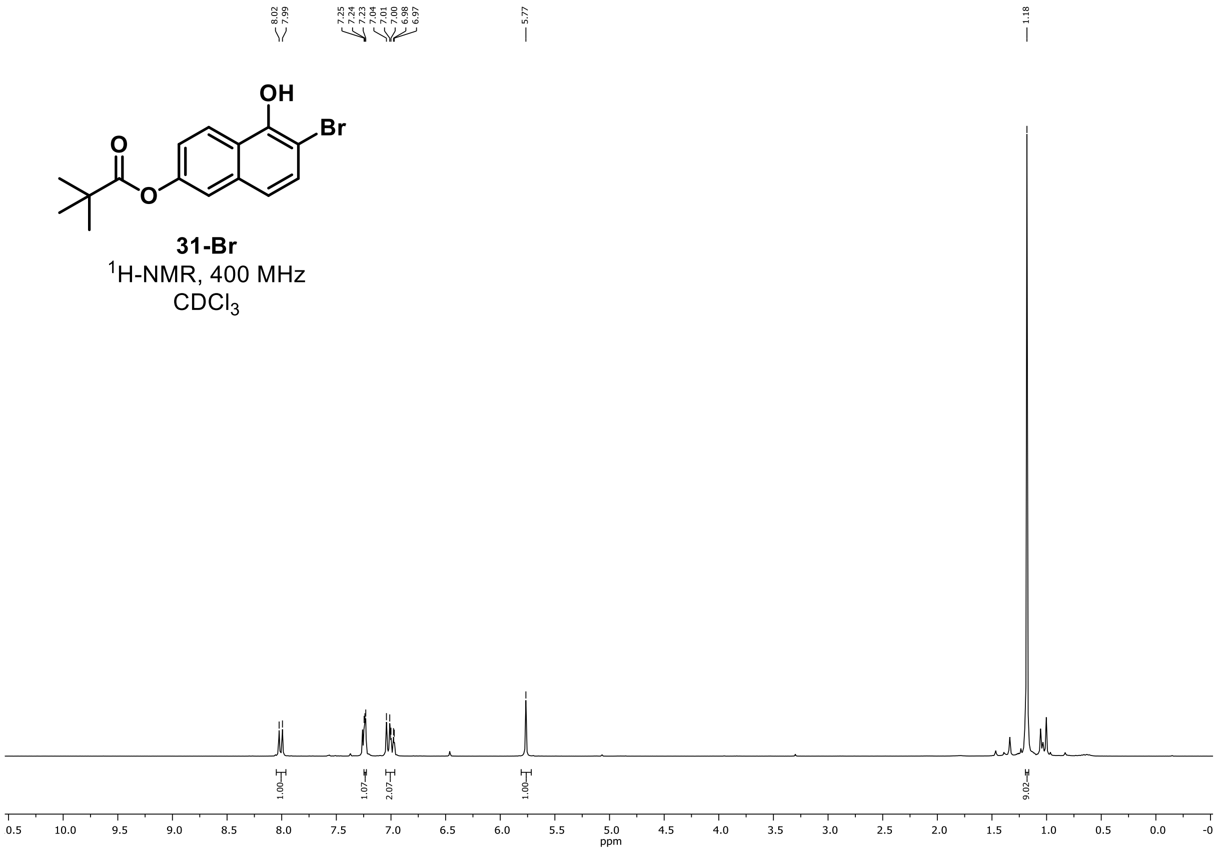
8.02
7.99
7.25
7.24
7.23
7.04
7.01
7.00
6.98
6.97
5.77
1.18
OH
Br
O
O
31-Br
1H-NMR, 400 MHz
CDCl3
1.00
1.07
2.07
1.00
9.02
ppm

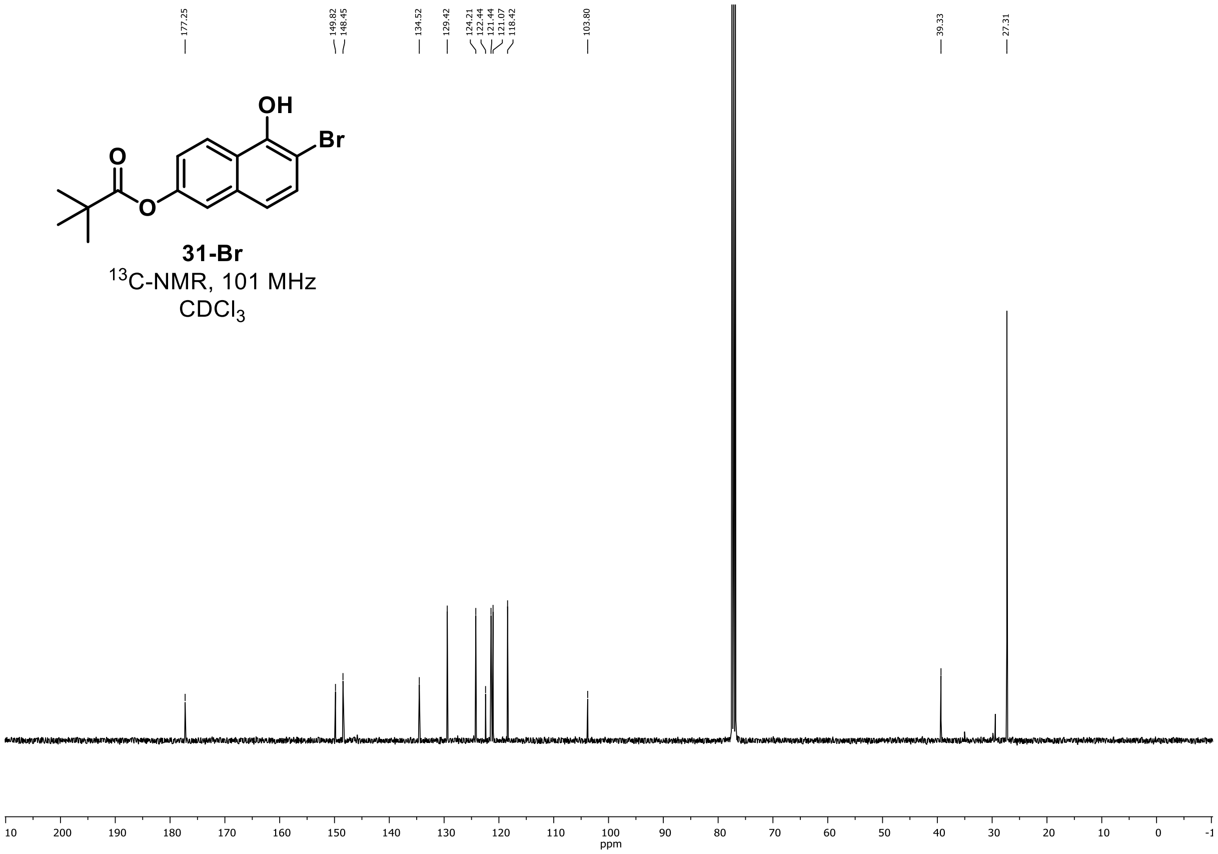
177.25
149.82
148.45
134.52
129.42
124.21
122.44
121.44
121.07
118.42
103.80
39.33
27.31
OH
Br
O
O
31-Br
13C-NMR, 101 MHz
CDCl3
ppm

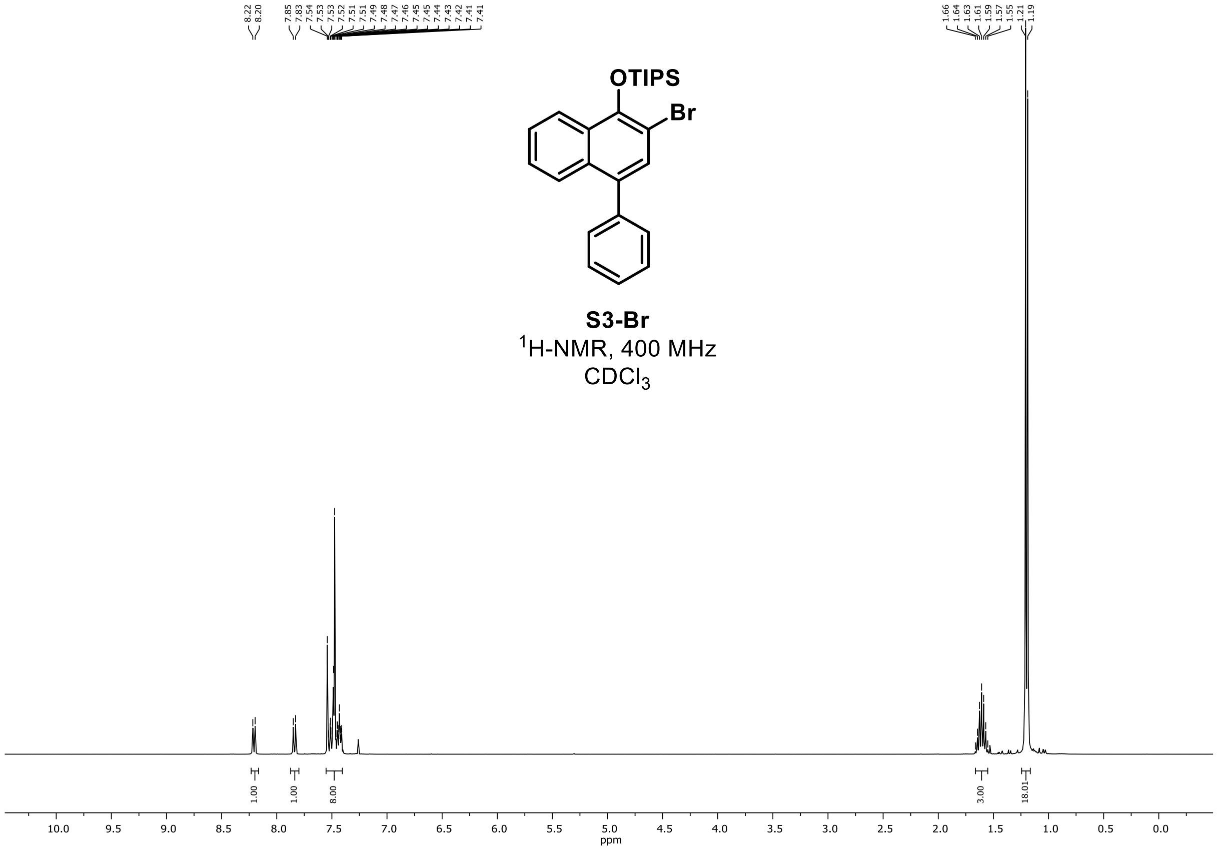
8.22
8.20
7.85
7.83
7.54
7.53
7.53
7.52
7.51
7.51
7.49
7.48
7.47
7.46
7.45
7.45
7.44
7.43
7.42
7.41
7.41
1.66
1.64
1.63
1.61
1.59
1.57
1.55
1.21
1.19
OTIPS
Br
S3-Br
1H-NMR, 400 MHz
CDCl3
1.00
1.00
8.00
3.00
18.01
ppm

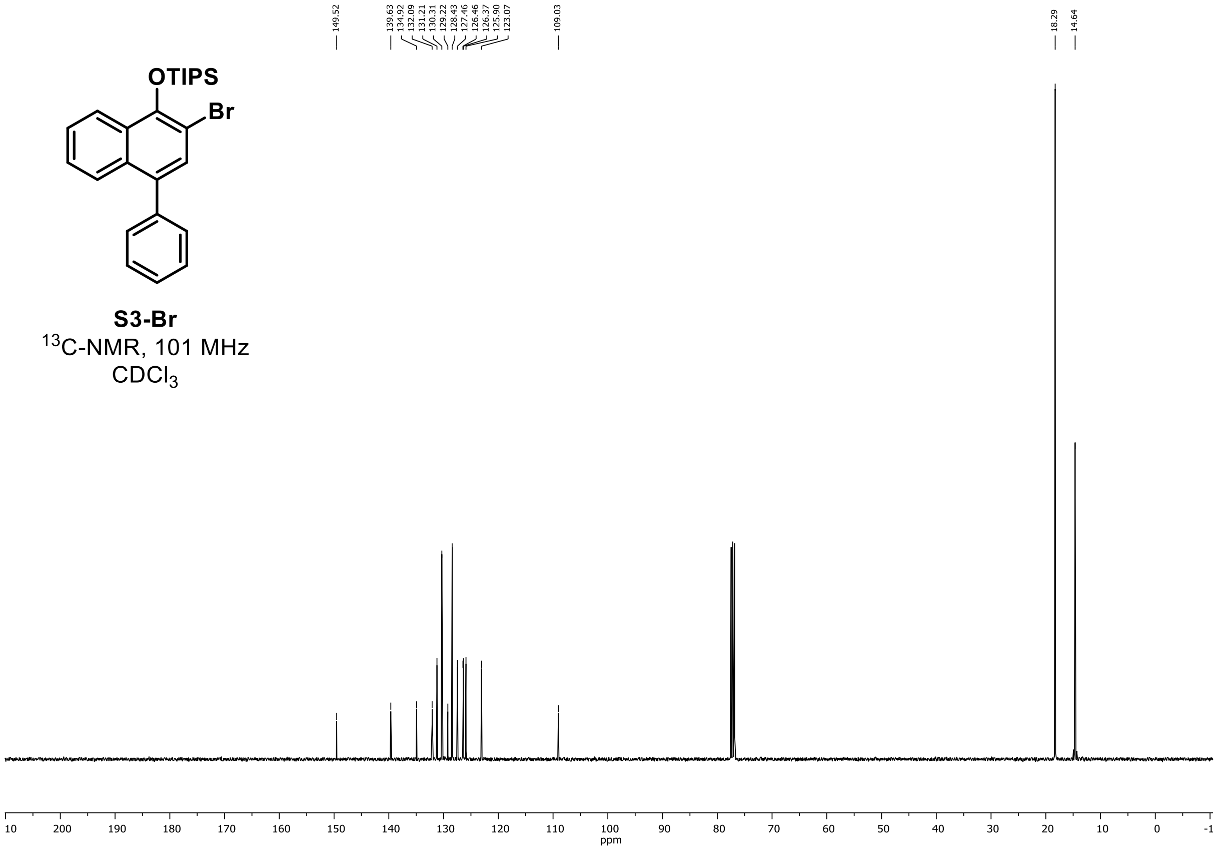

S3-Br
^{13}C-NMR, 101 MHz
$CDCl_3$

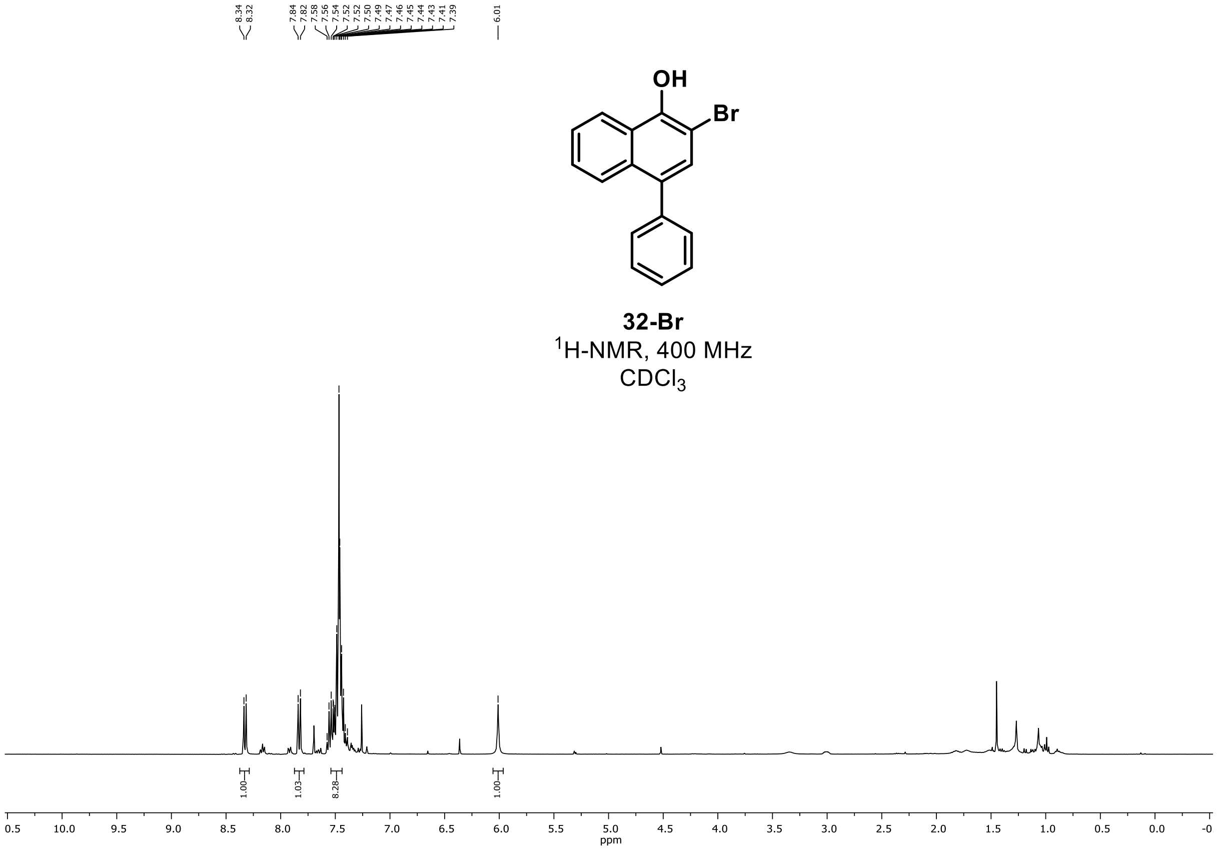

32-Br
^{1}H-NMR, 400 MHz
$CDCl_3$

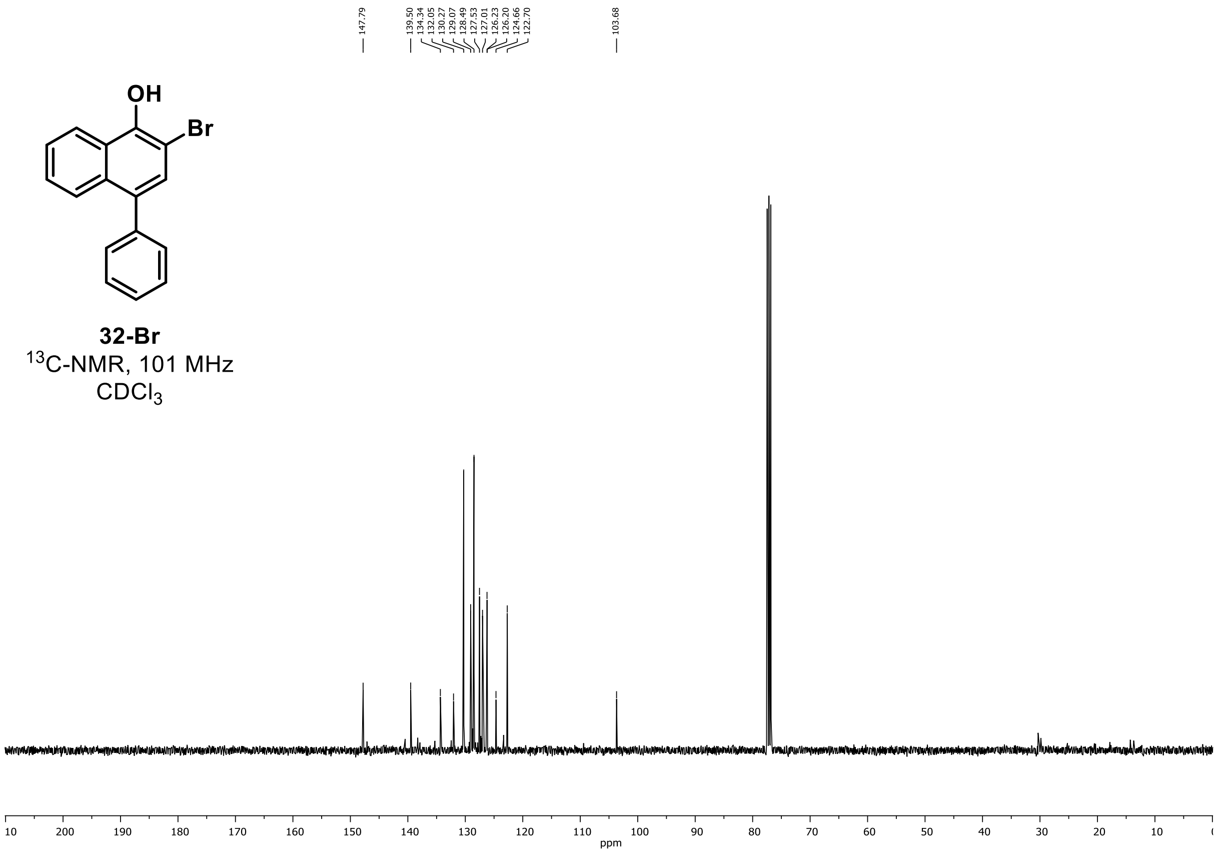

32-Br
^{13}C-NMR, 101 MHz
$CDCl_3$

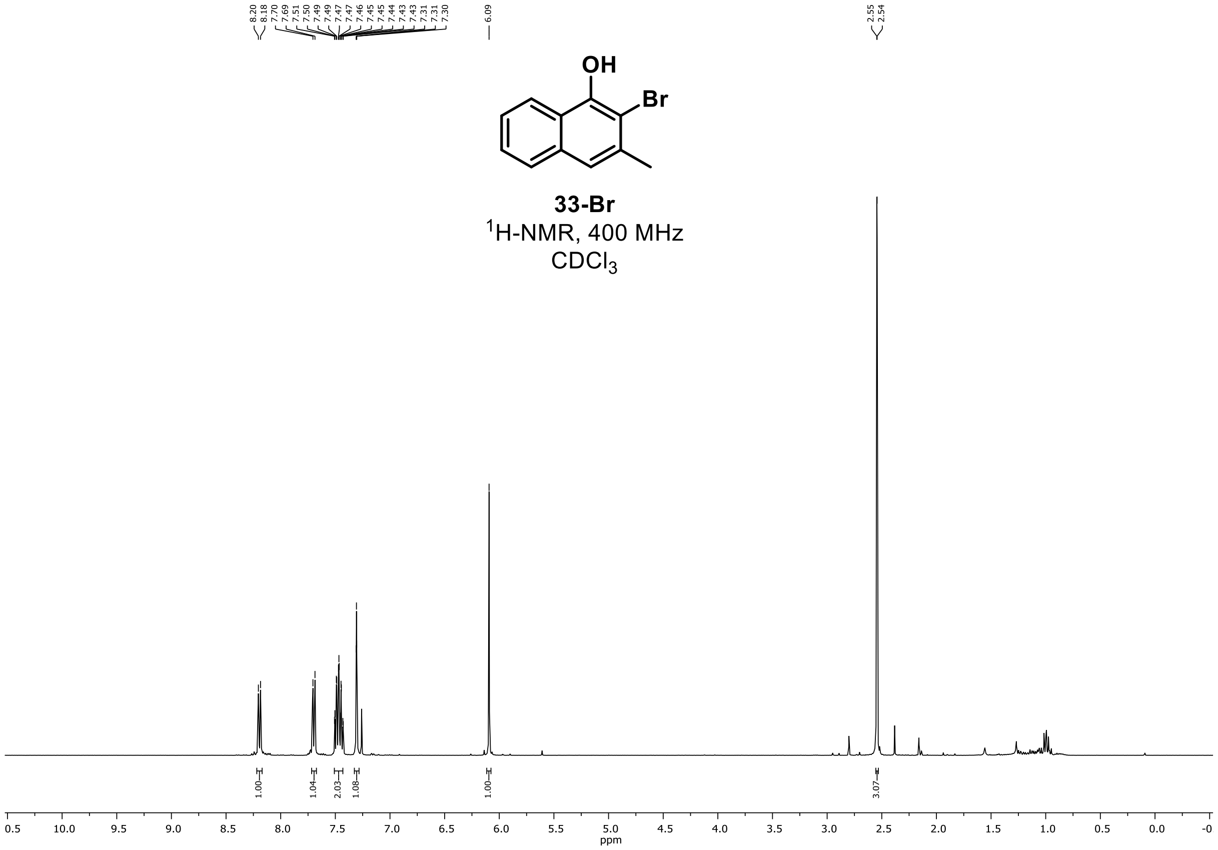

33-Br
^{1}H-NMR, 400 MHz
$CDCl_3$

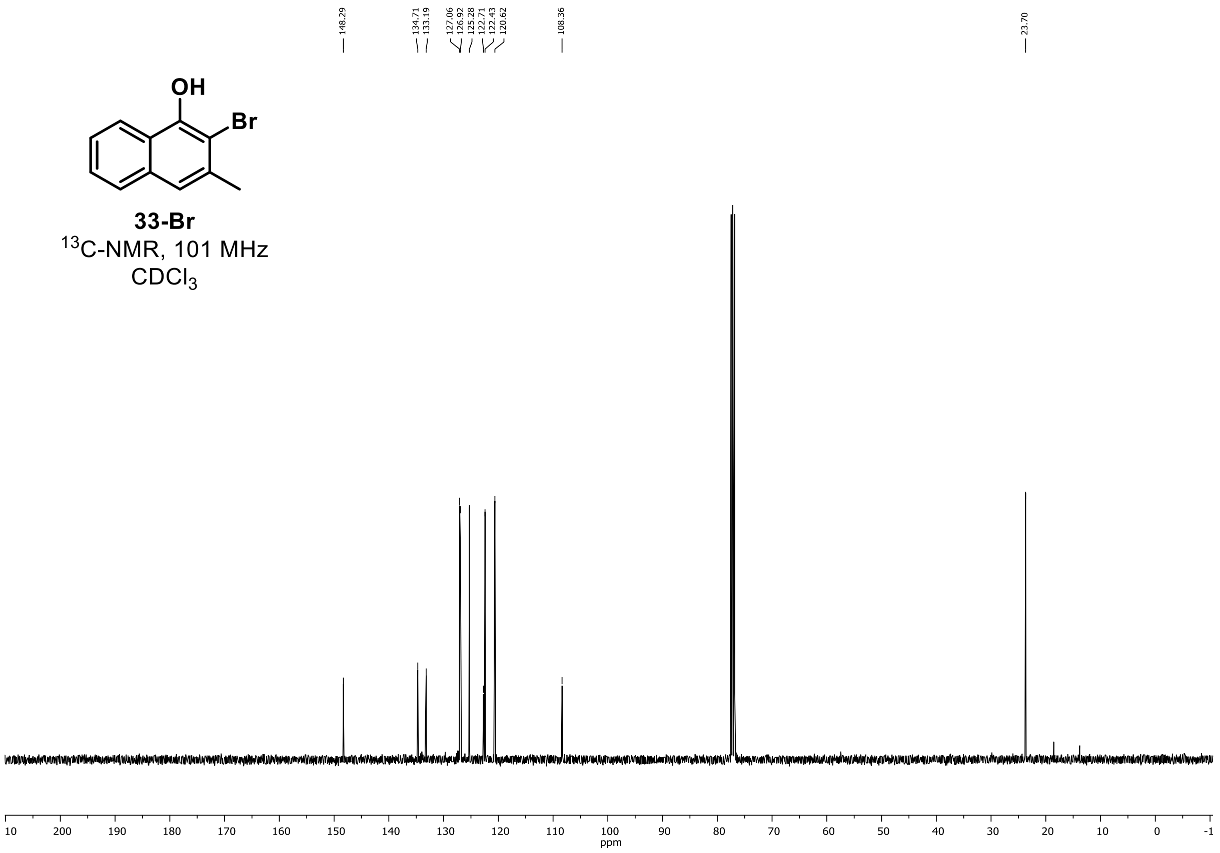
148.29
134.71
133.19
127.06
126.92
125.28
122.71
122.43
120.62
108.36
23.70
OH
Br
33-Br
^{13}C-NMR, 101 MHz
$CDCl_3$
ppm

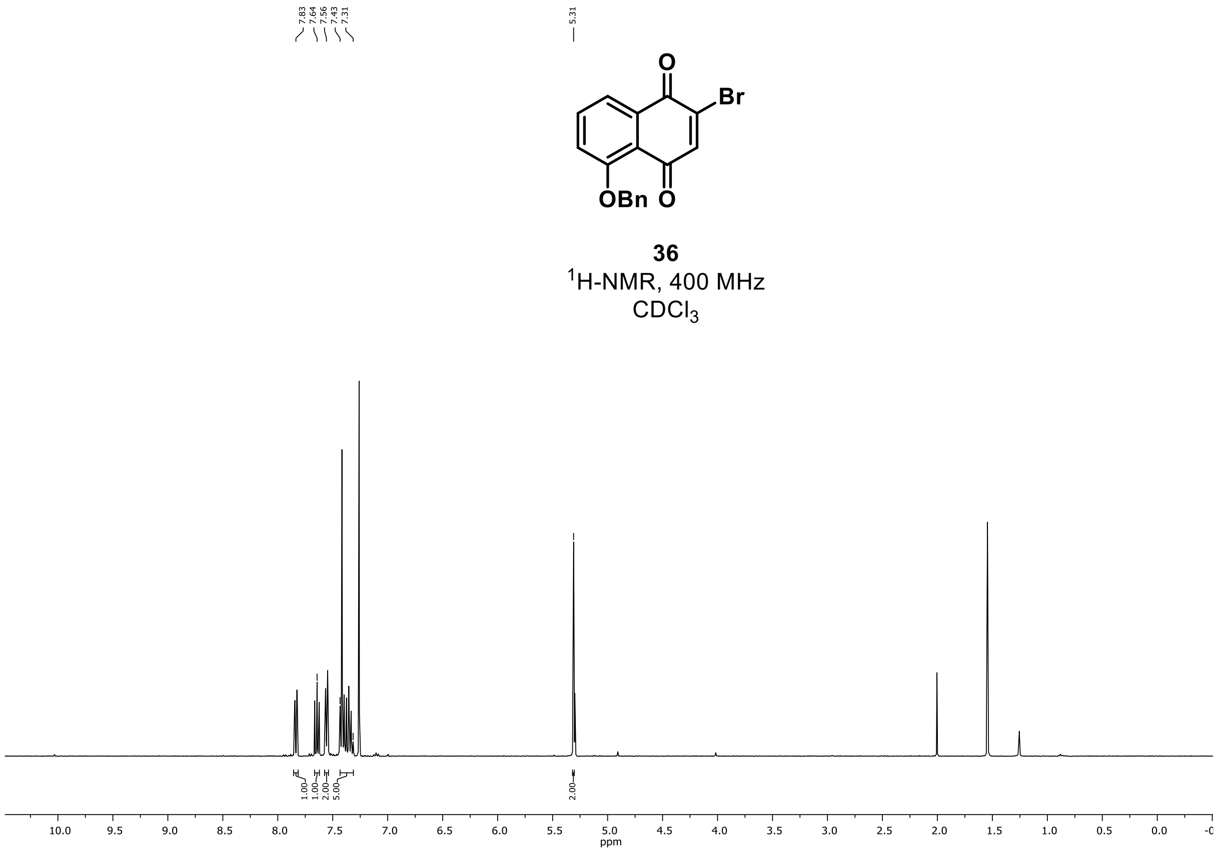
7.83
7.64
7.56
7.43
7.31
5.31
O
Br
OBn O
36
^{1}H-NMR, 400 MHz
$CDCl_3$
1.00
1.00
2.00
5.00
2.00
ppm

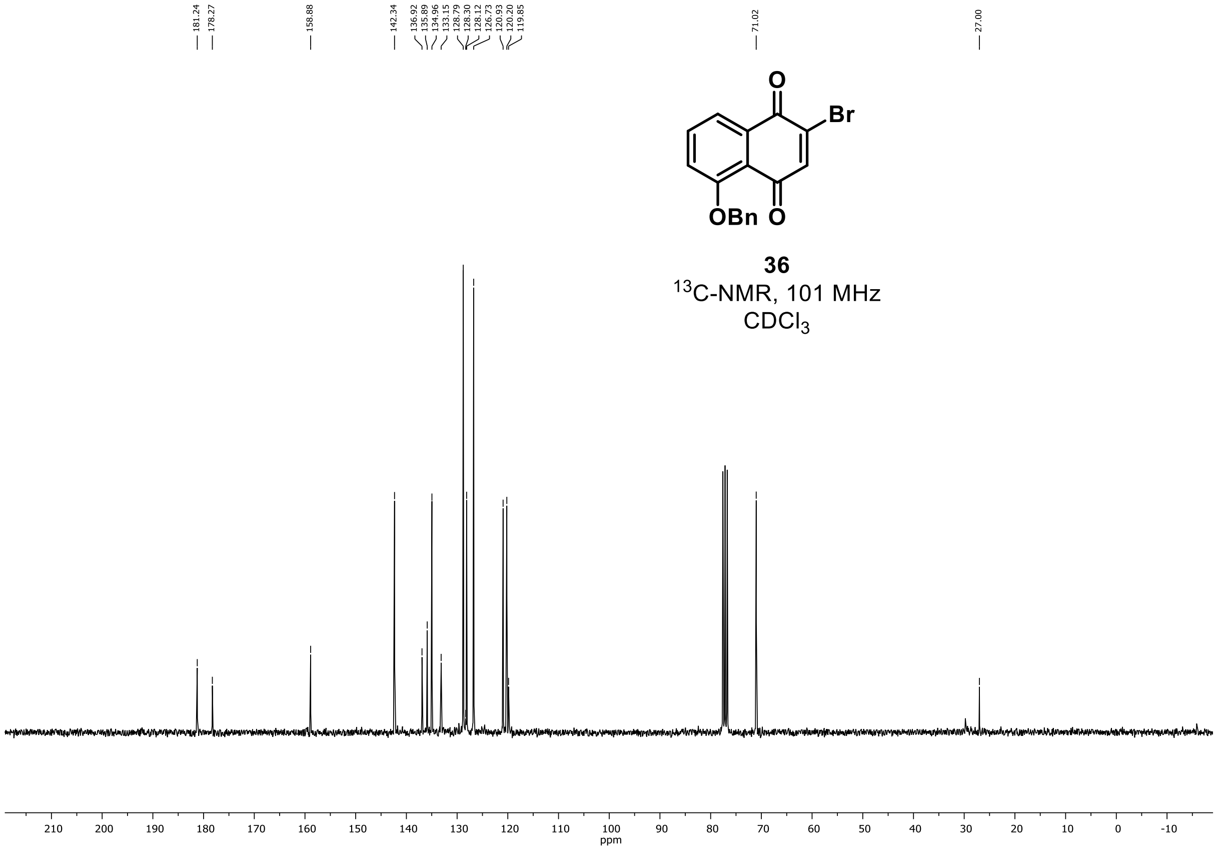
181.24
178.27
158.88
142.34
136.92
135.89
134.96
133.15
128.79
128.30
128.12
126.73
120.93
120.20
119.85
71.02
27.00
O
Br
OBn O
36
^{13}C-NMR, 101 MHz
$CDCl_3$
ppm

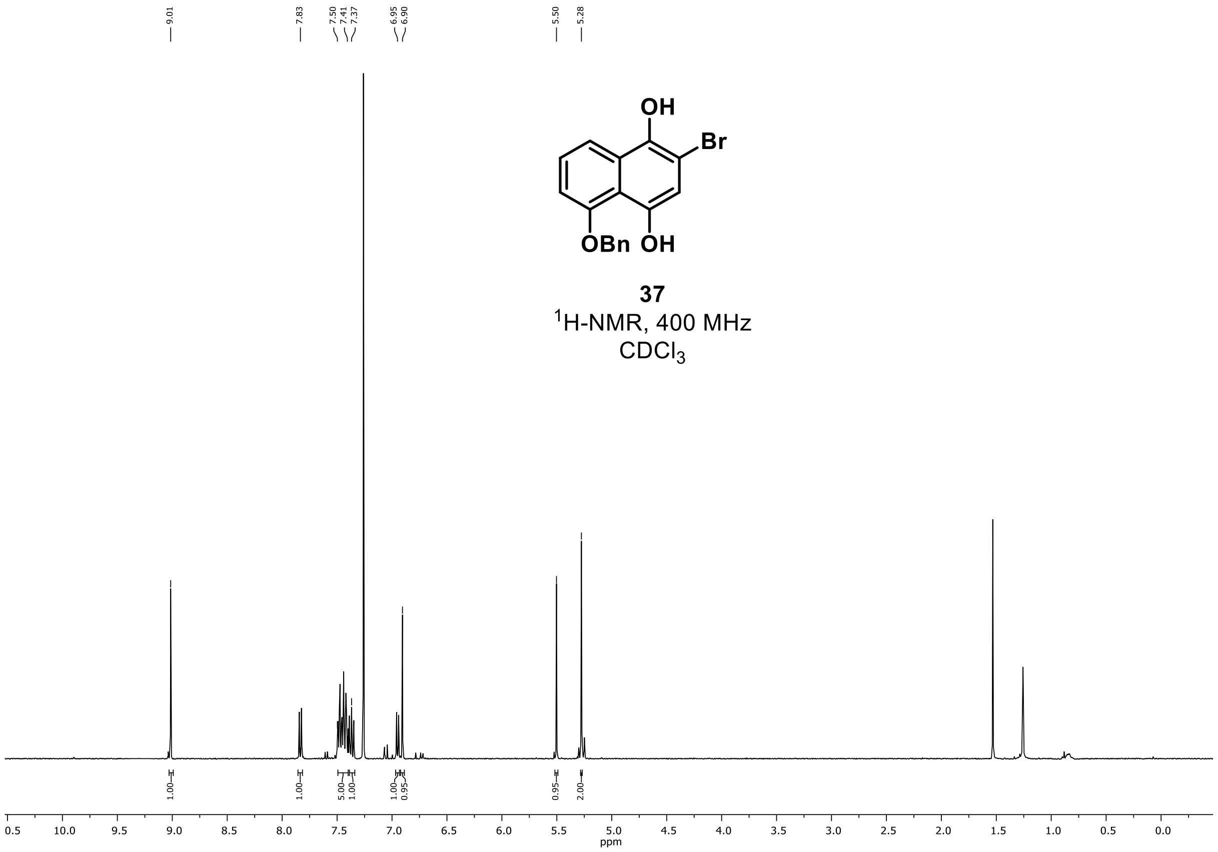
9.01
7.83
7.50
7.41
7.37
6.95
6.90
5.50
5.28
OH
Br
OBn OH
37
^{1}H-NMR, 400 MHz
$CDCl_3$
1.00
1.00
5.00
1.00
1.00
0.95
0.95
2.00
ppm

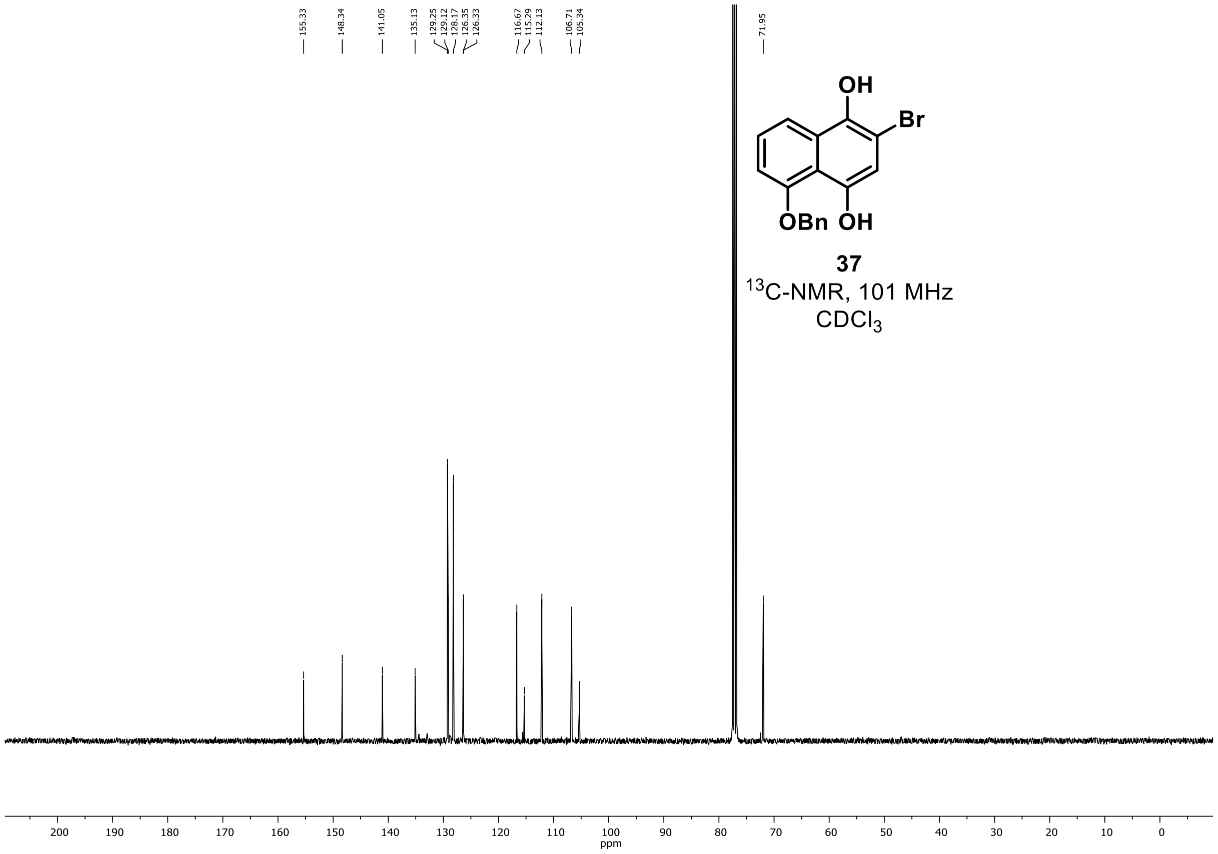
OH
Br
OBn OH
37
13C-NMR, 101 MHz
CDCl3
ppm

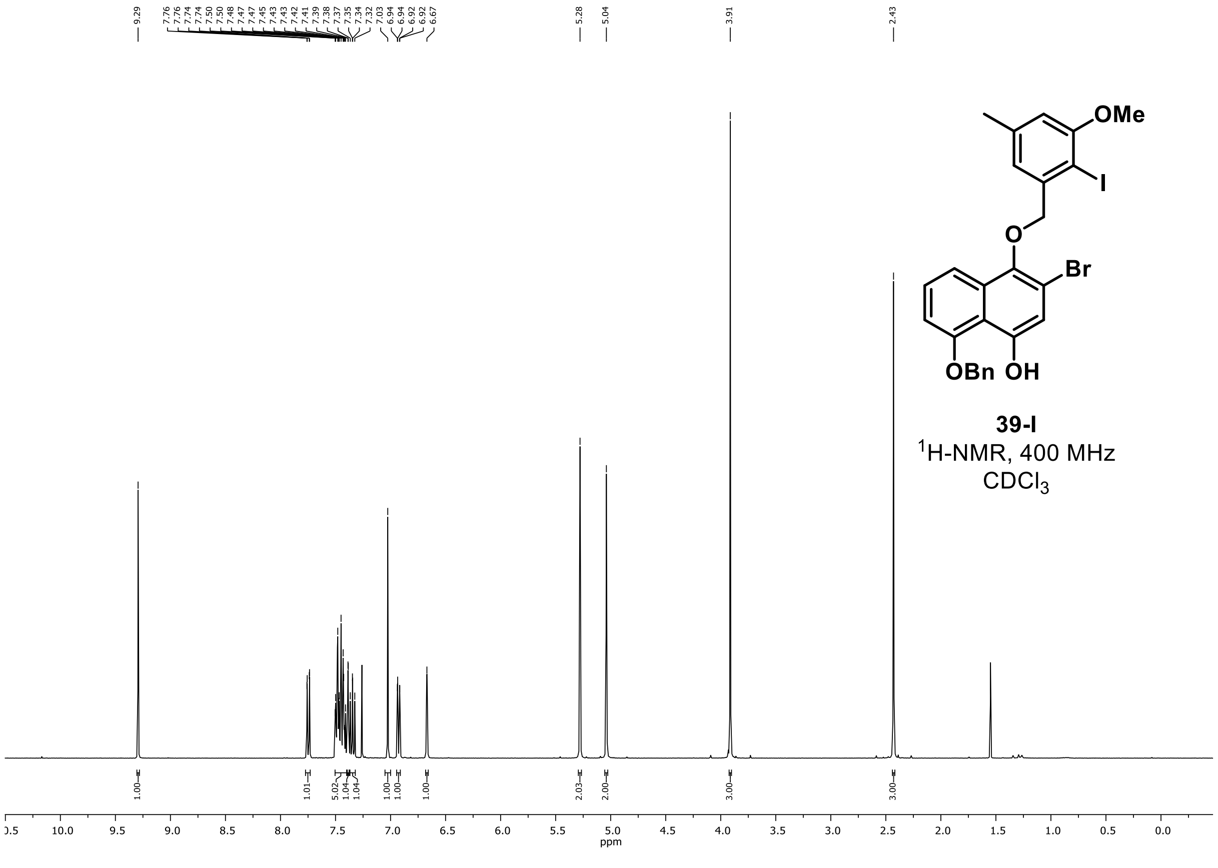
OMe
I
O
Br
OBn OH
39-I
1H-NMR, 400 MHz
CDCl3
ppm

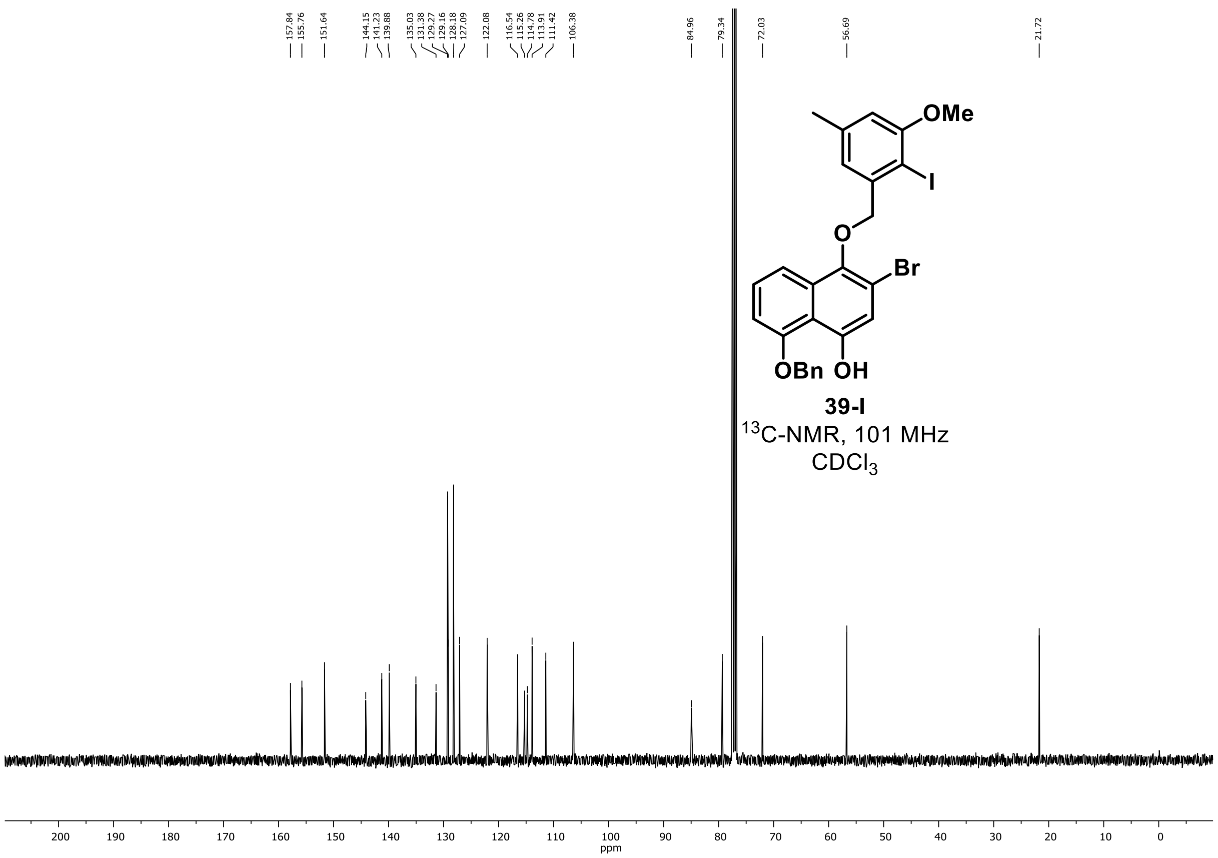
OMe
I
O
Br
OBn OH
39-I
^{13}C-NMR, 101 MHz
$CDCl_3$
ppm

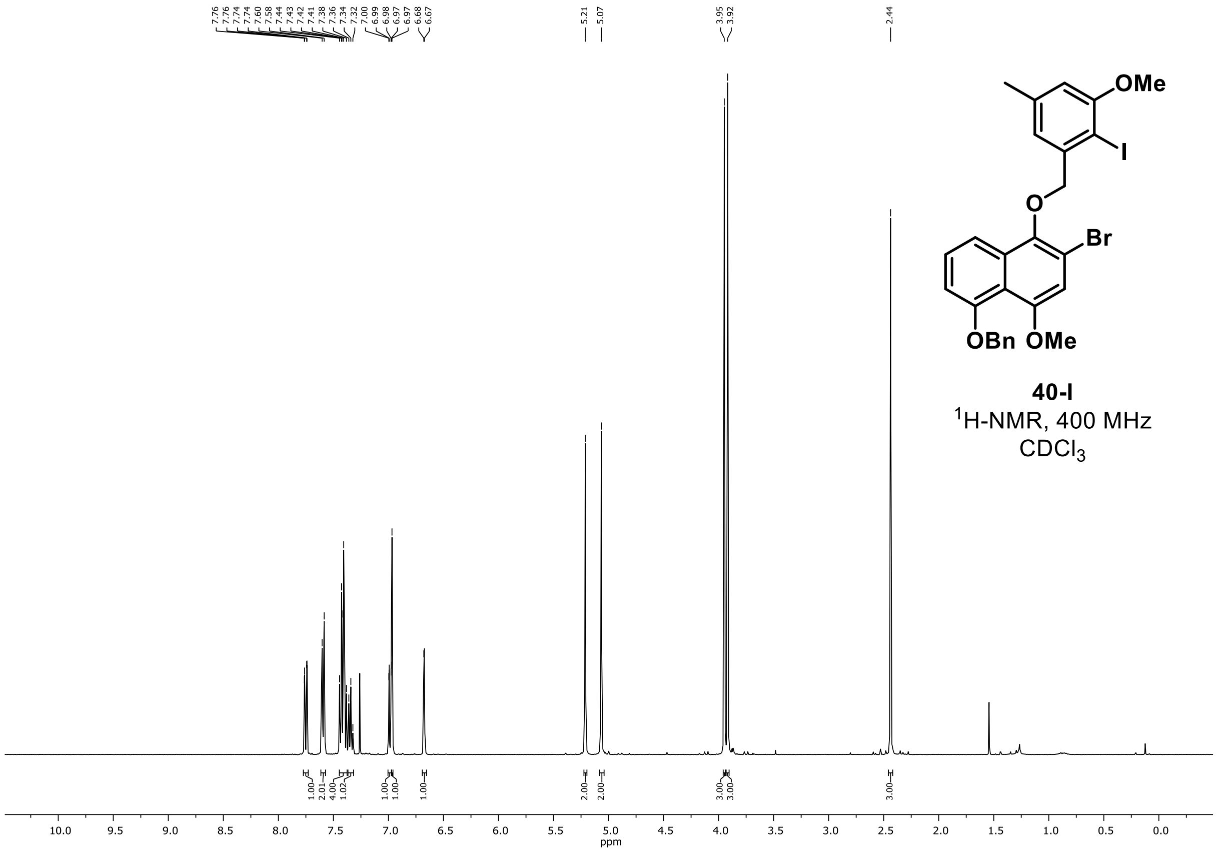
OMe
I
O
Br
OBn OMe
40-I
^{1}H-NMR, 400 MHz
$CDCl_3$
ppm

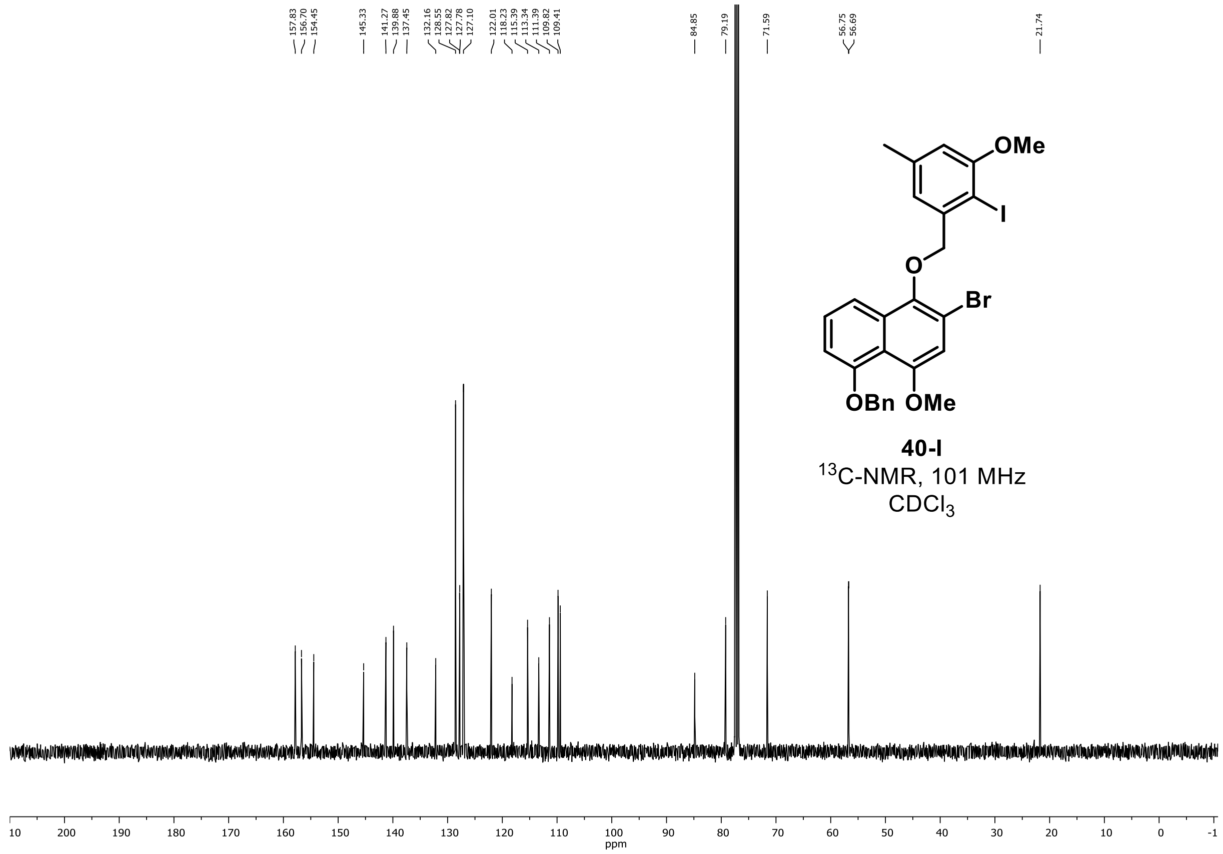
157.83
156.70
154.45
145.33
141.27
139.88
137.45
132.16
128.55
127.82
127.78
127.10
122.01
118.23
115.39
113.34
111.39
109.82
109.41
84.85
79.19
71.59
56.75
56.69
21.74
OMe
I
O
Br
OBn OMe
40-I
13C-NMR, 101 MHz
CDCl3
ppm

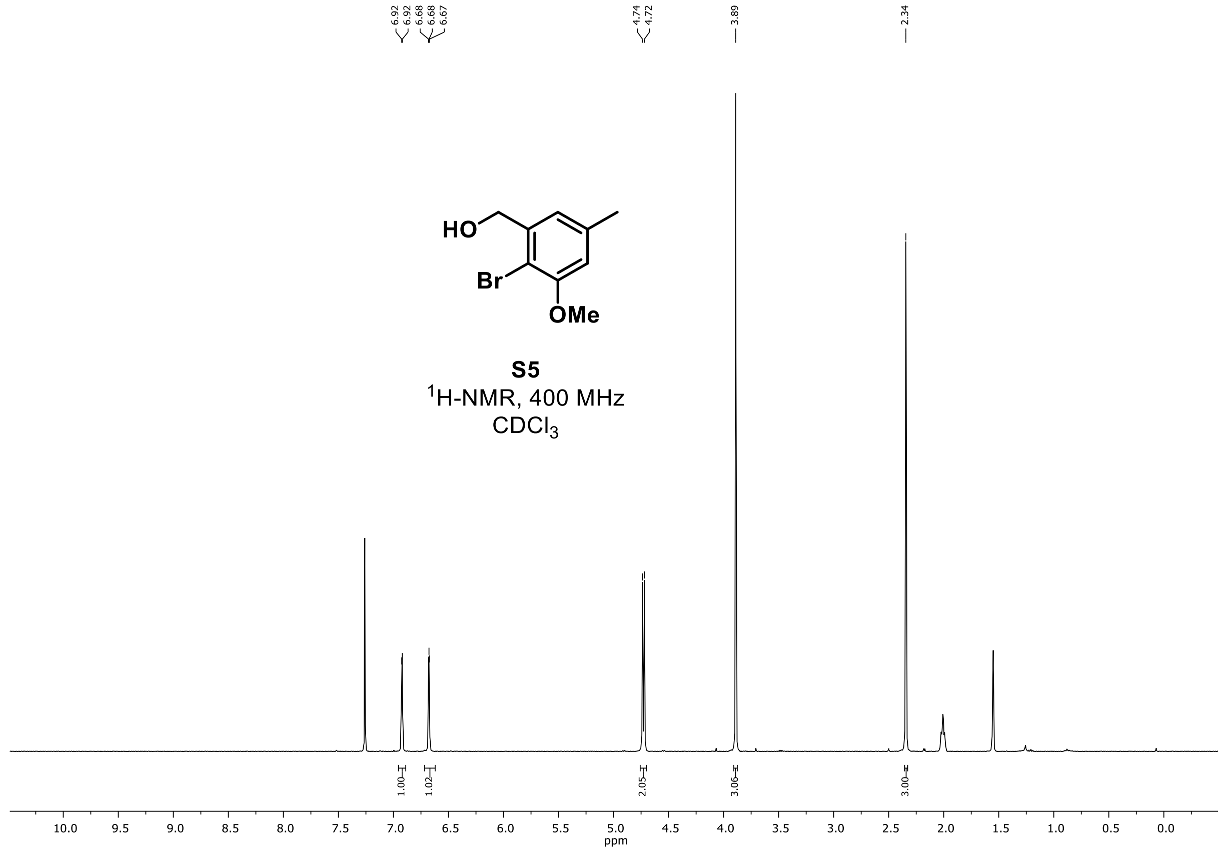
6.92
6.92
6.68
6.68
6.67
4.74
4.72
3.89
2.34
HO
Br
OMe
S5
1H-NMR, 400 MHz
CDCl3
1.00
1.02
2.05
3.06
3.00
ppm

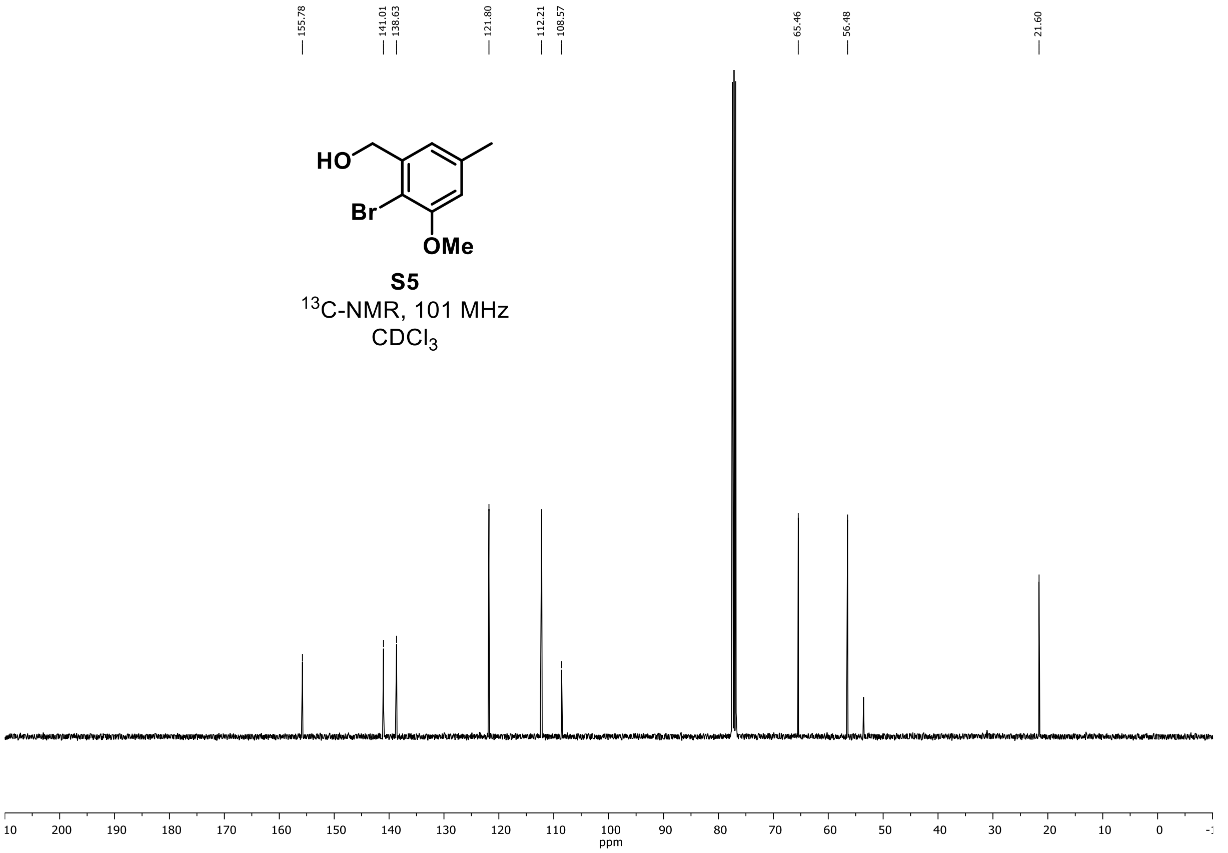
155.78
141.01
138.63
121.80
112.21
108.57
65.46
56.48
21.60
HO
Br
OMe
S5
13C-NMR, 101 MHz
CDCl3
ppm

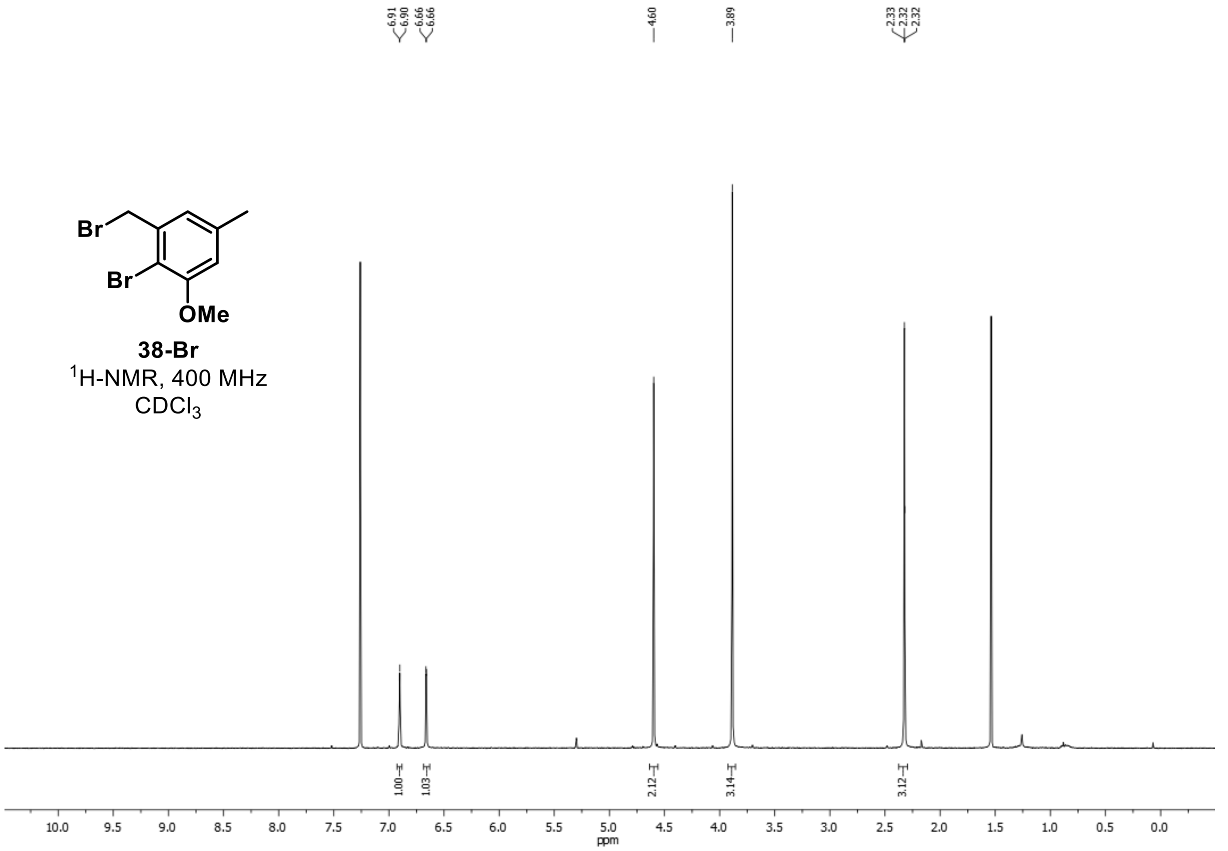
6.91
6.90
6.66
6.66
4.60
3.89
2.33
2.32
2.32
Br
Br
OMe
38-Br
1H-NMR, 400 MHz
CDCl3
1.00
1.03
2.12
3.14
3.12
ppm

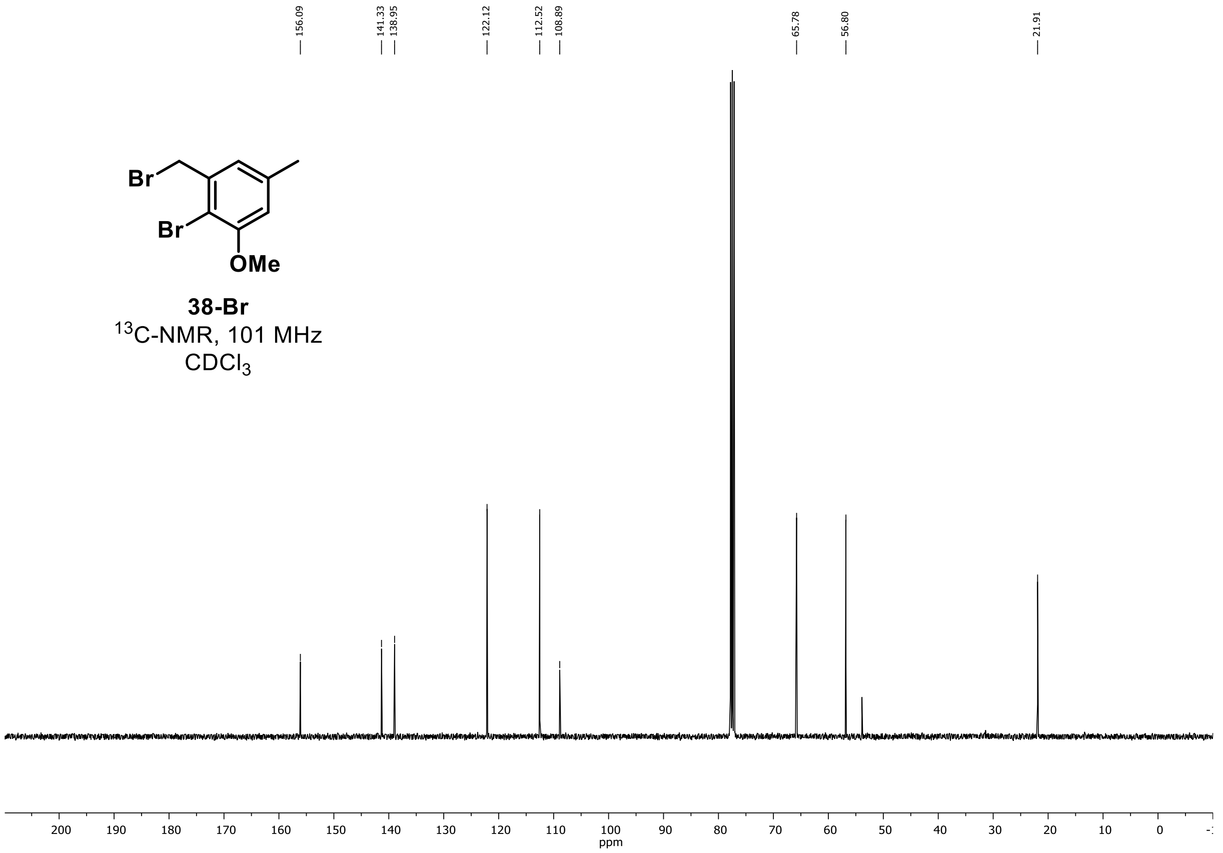
156.09
141.33
138.95
122.12
112.52
108.89
65.78
56.80
21.91
Br
Br
OMe
38-Br
13C-NMR, 101 MHz
CDCl3
ppm

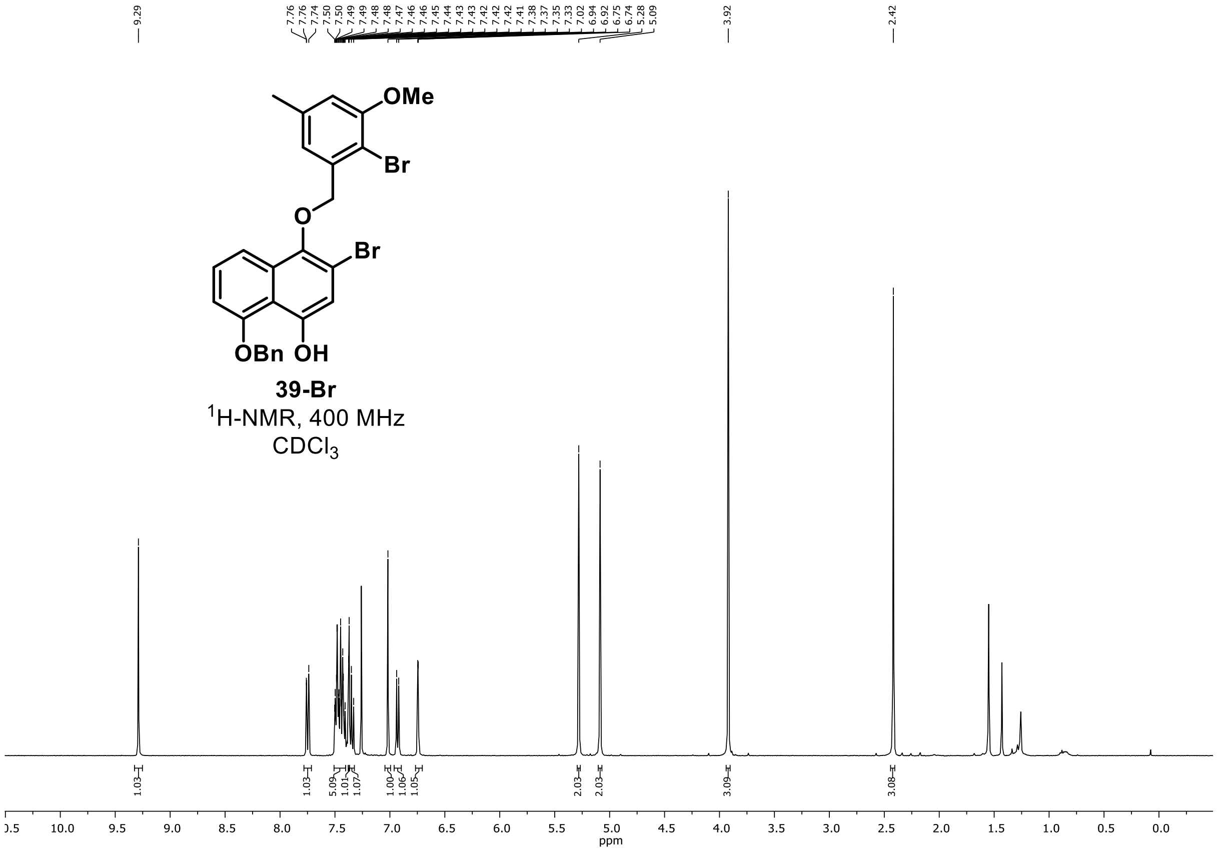
9.29
3.92
2.42
OMe
Br
O
Br
OBn OH
39-Br
1H-NMR, 400 MHz
CDCl3
ppm

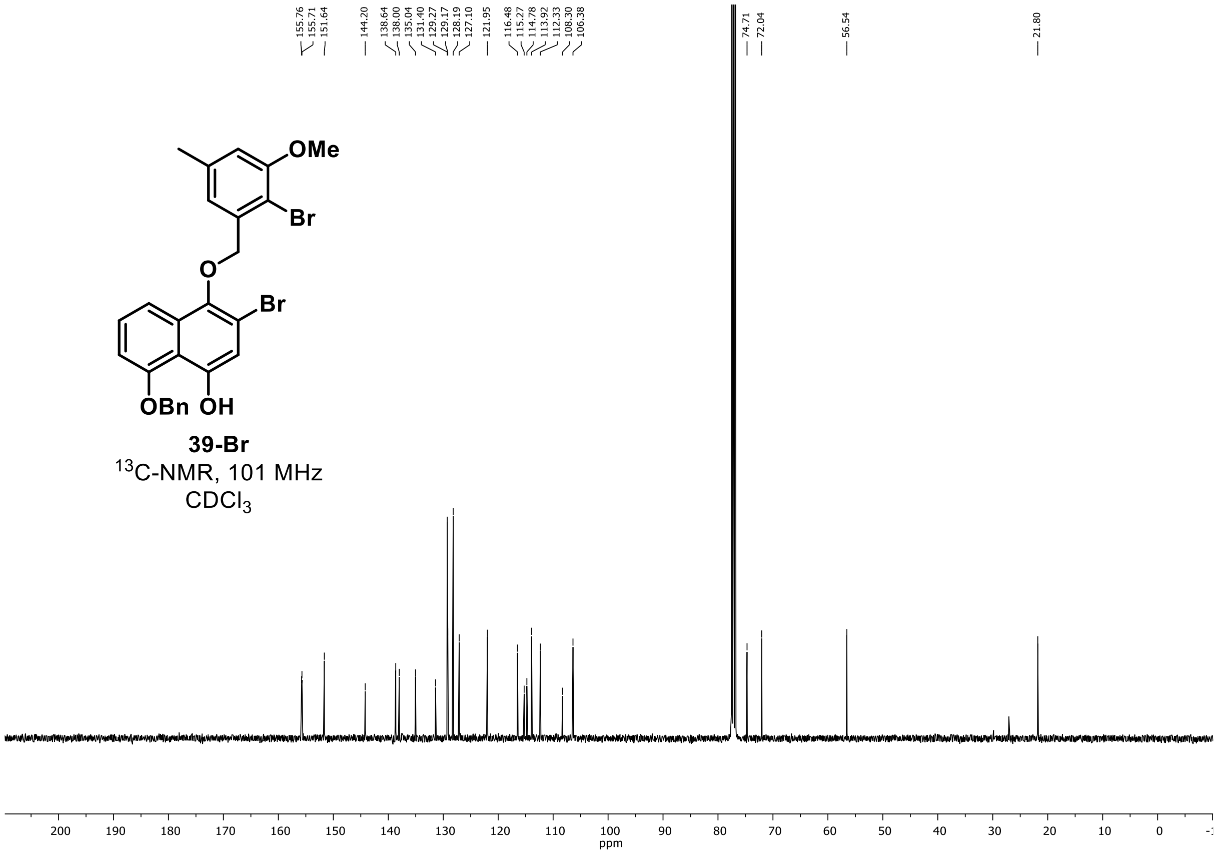

39-Br
^{13}C-NMR, 101 MHz
$CDCl_3$

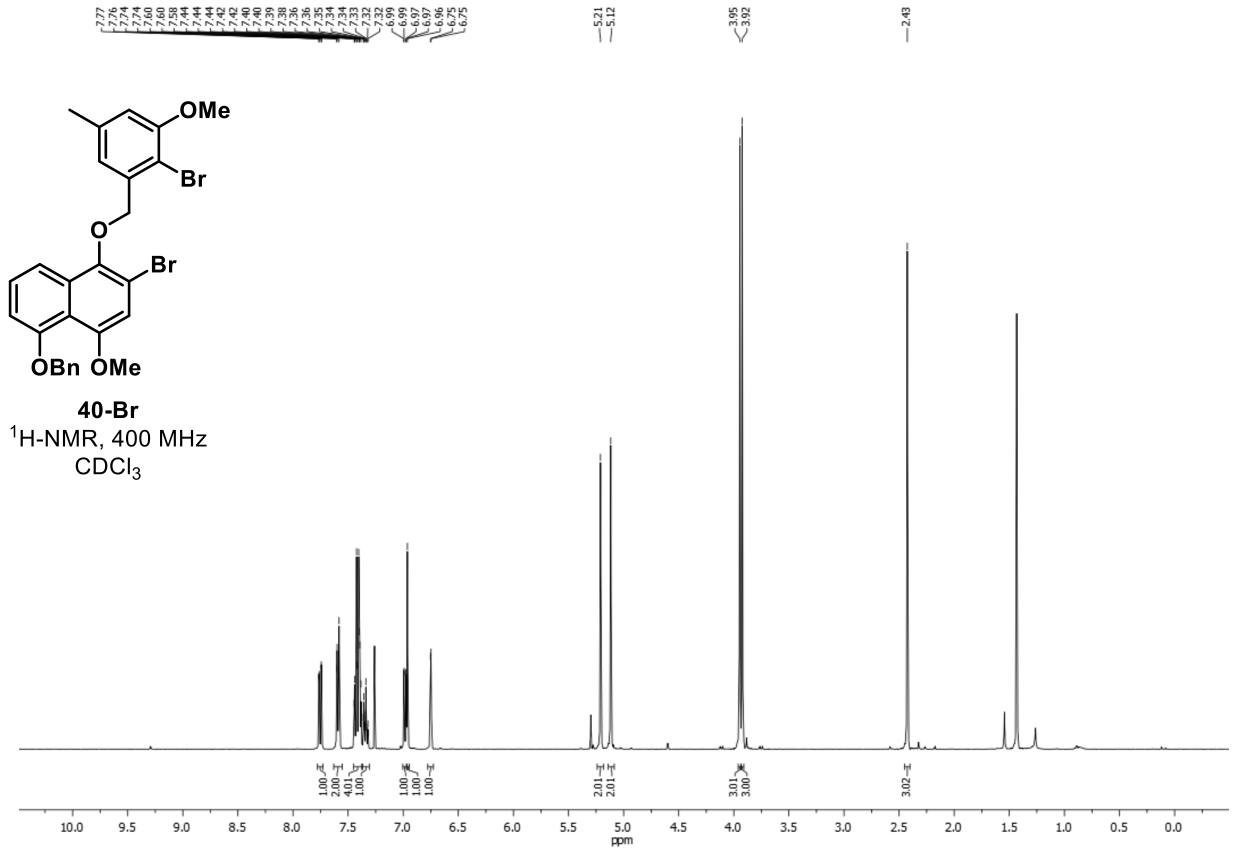

40-Br
^{1}H-NMR, 400 MHz
$CDCl_3$

40-Br
^{13}C-NMR, 101 MHz
$CDCl_3$

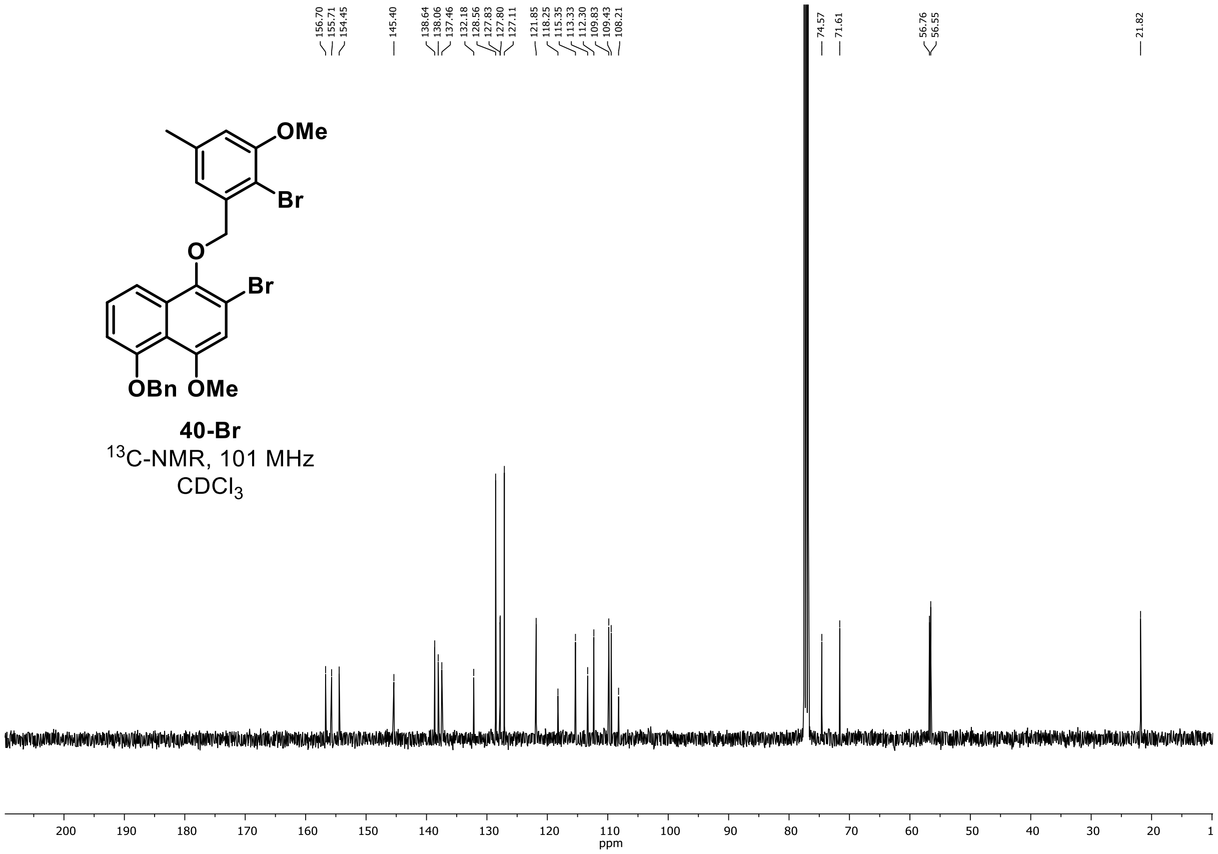

41a
^{1}H-NMR, 400 MHz
$CDCl_3$

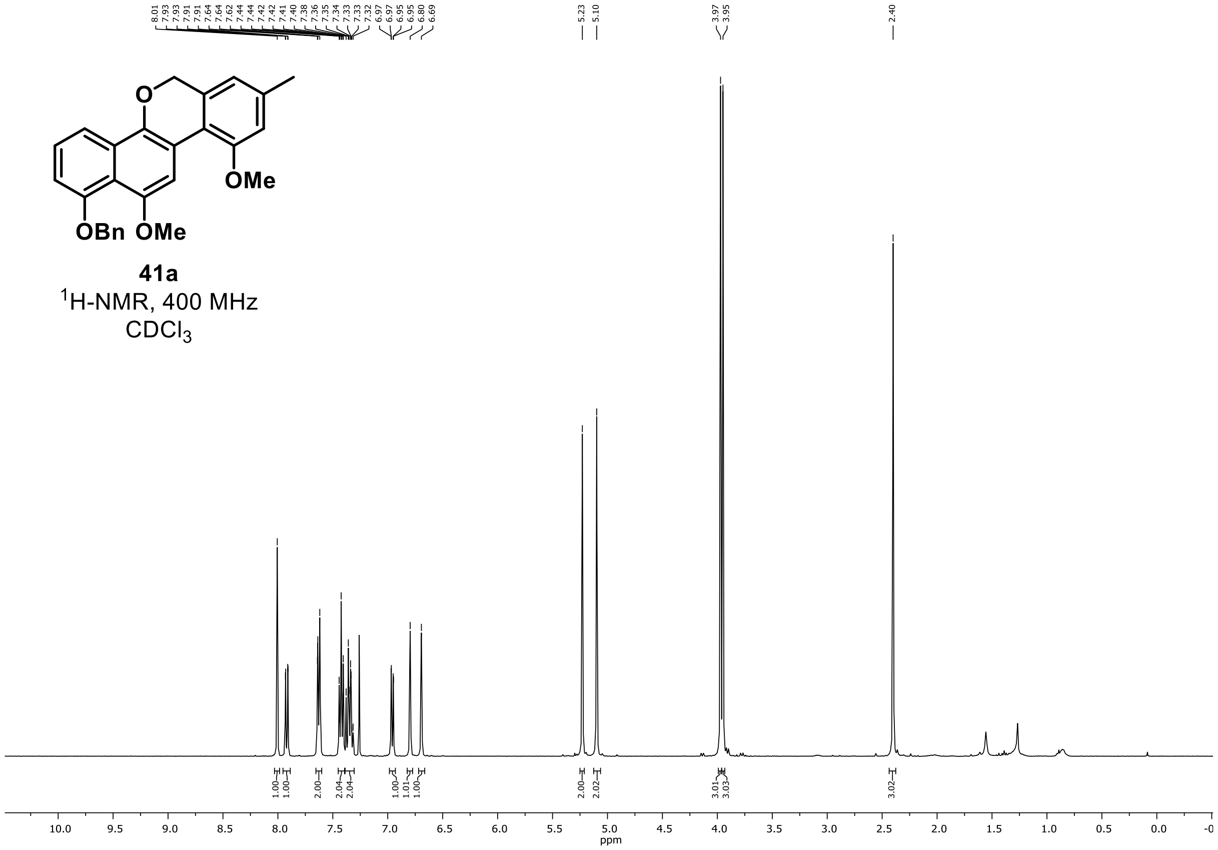

41a

^{13}C-NMR, 101 MHz

$CDCl_3$

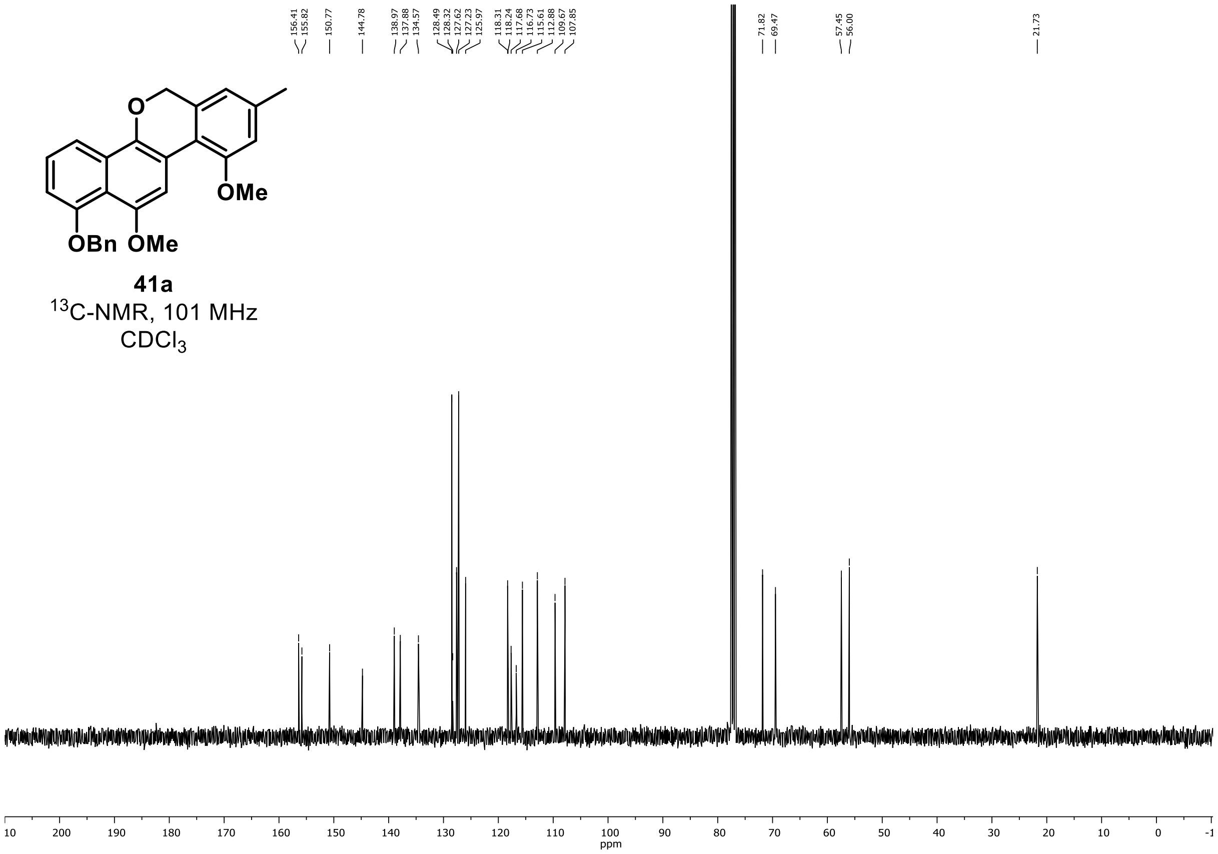

41b

^{1}H-NMR, 400 MHz

$CDCl_3$

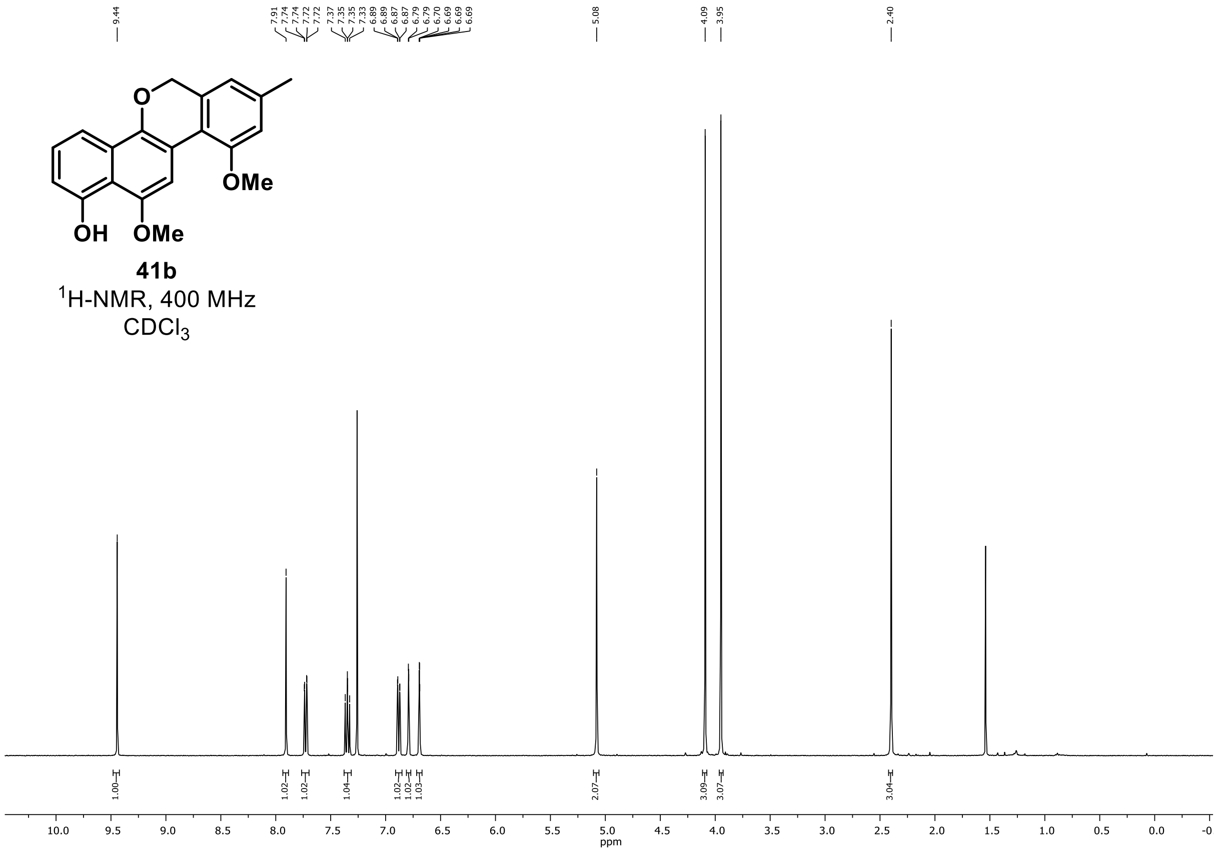

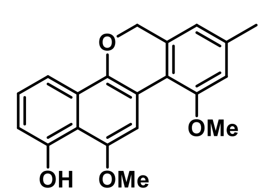

41b
^{13}C-NMR, 101 MHz
$CDCl_3$

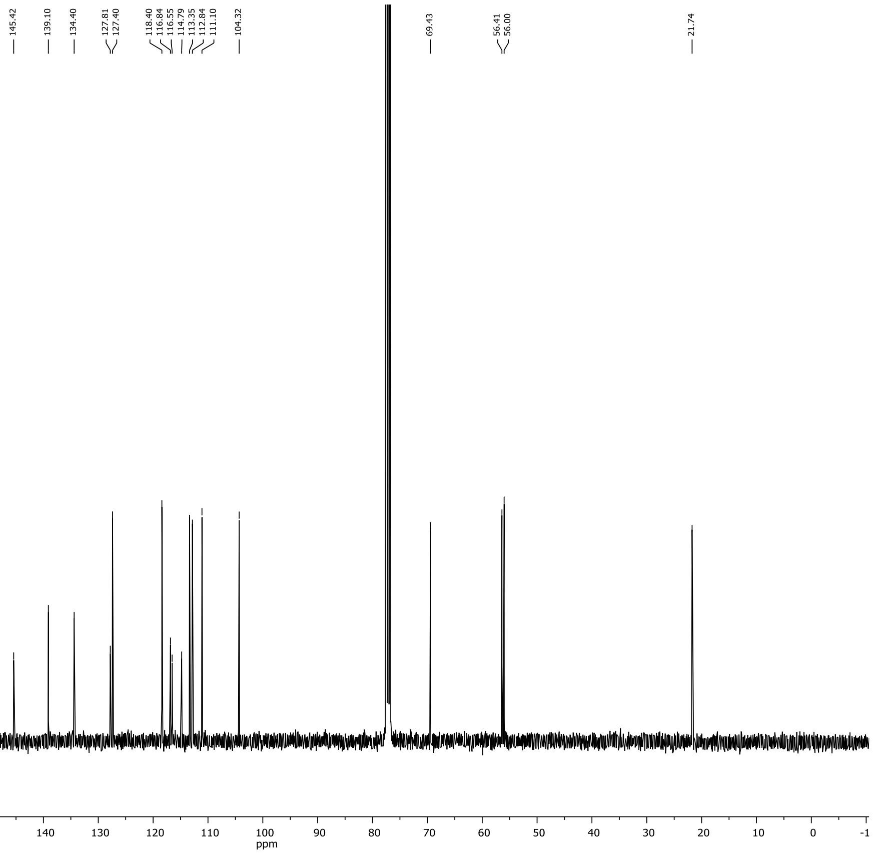

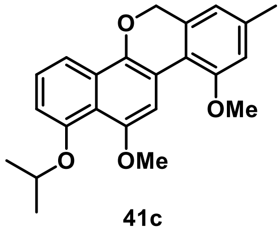

41c
^{1}H-NMR, 400 MHz
$CDCl_3$

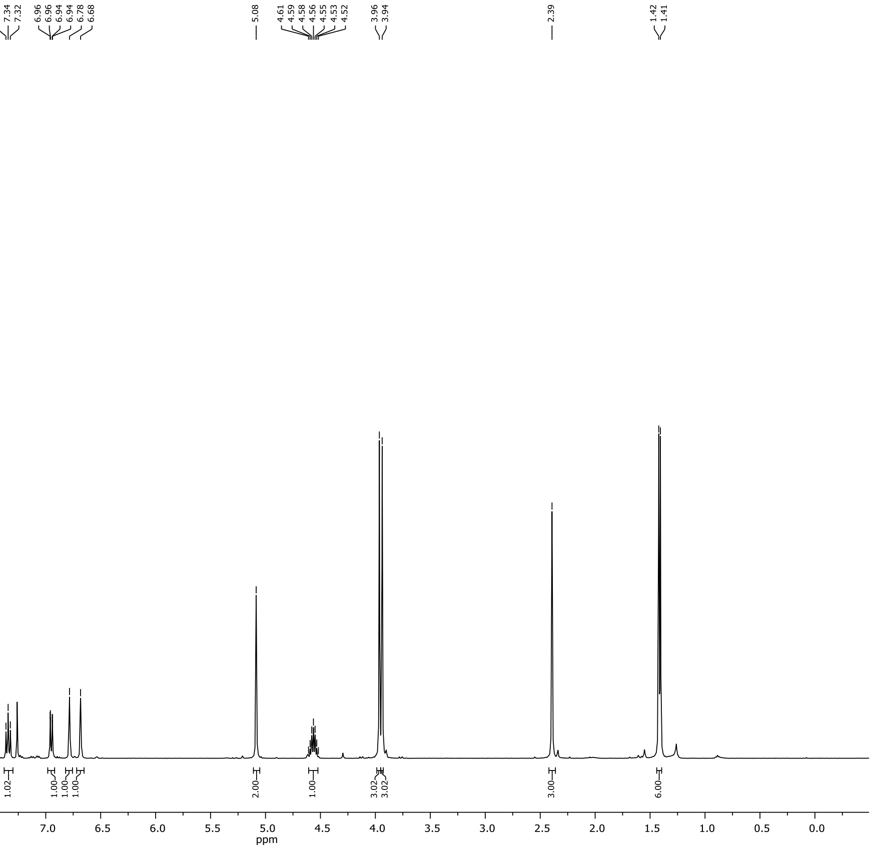

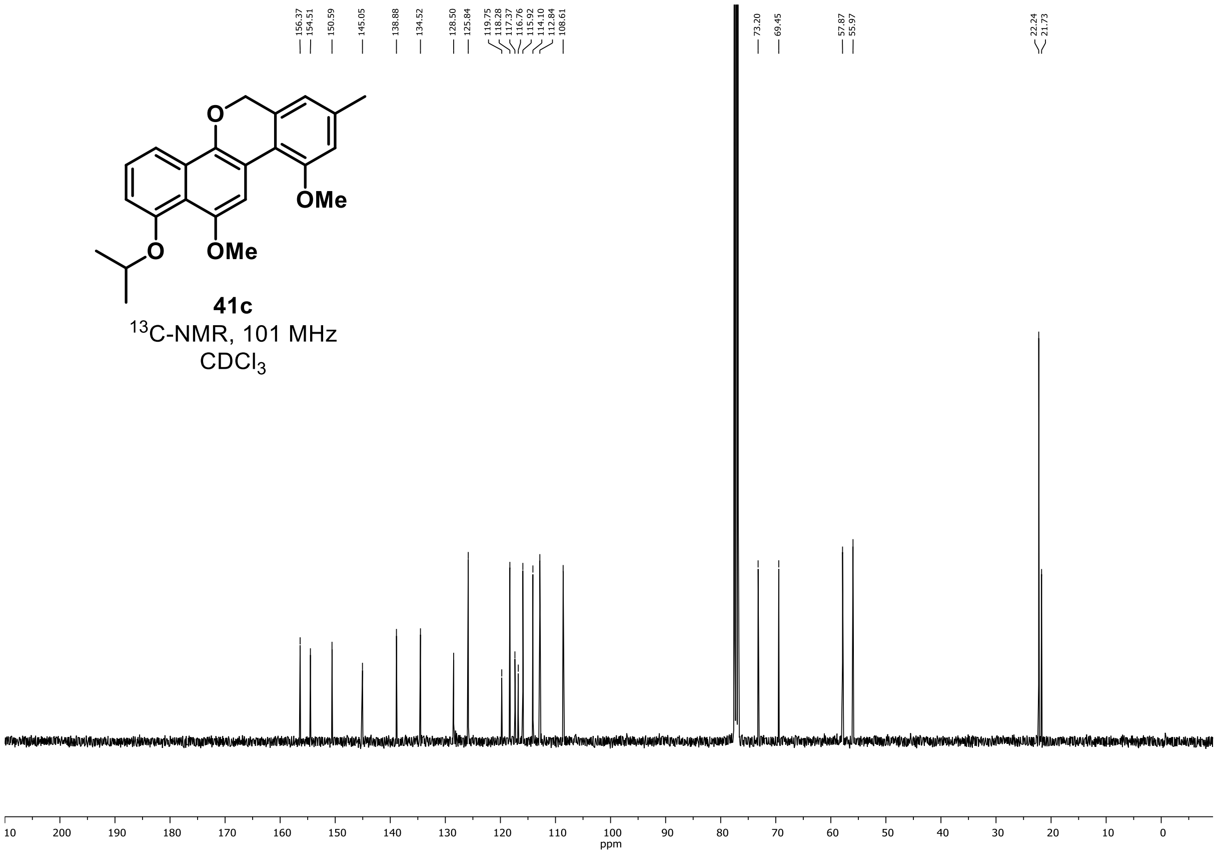
156.37
154.51
150.59
145.05
138.88
134.52
128.50
125.84
119.75
118.28
117.37
116.76
115.92
114.10
112.84
108.61
73.20
69.45
57.87
55.97
22.24
21.73
OMe
O
OMe
41c
^{13}C-NMR, 101 MHz
$CDCl_3$
ppm

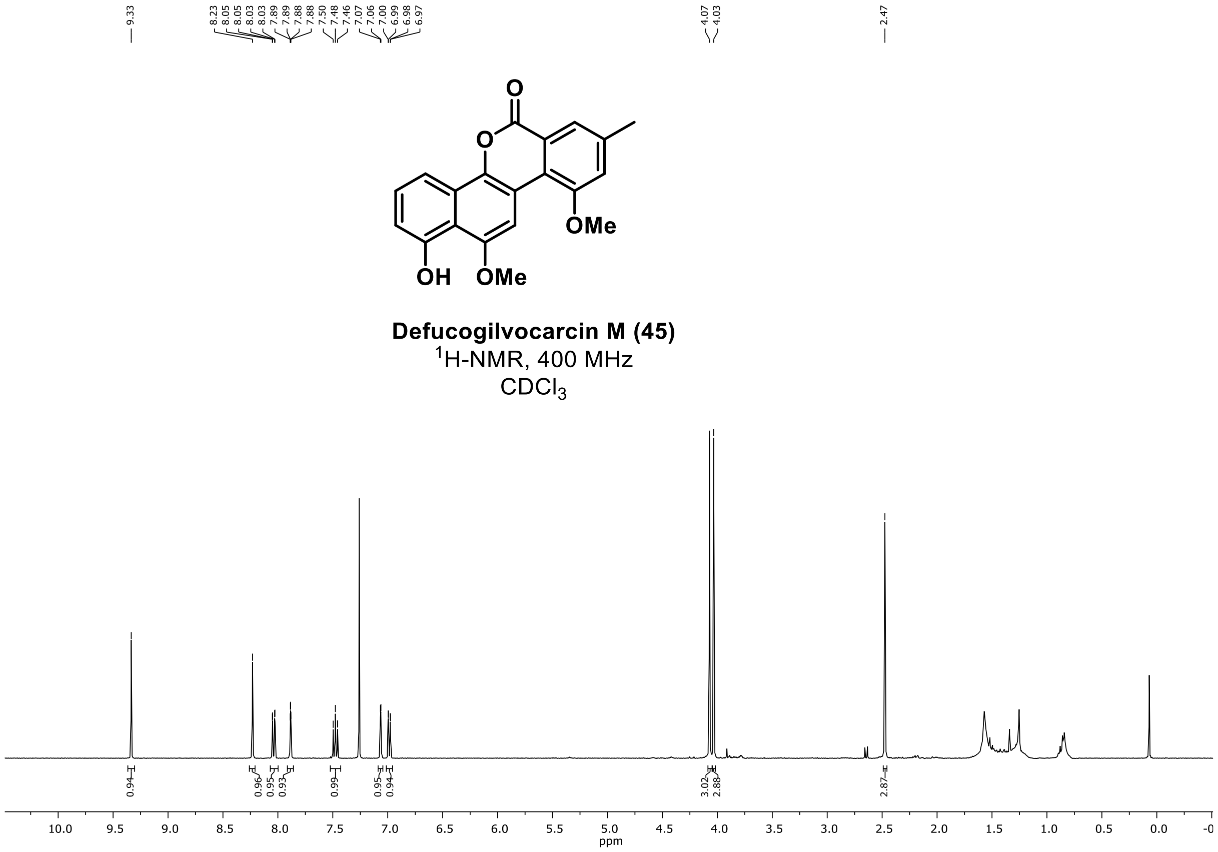
9.33
8.23
8.05
8.05
8.03
8.03
7.89
7.89
7.88
7.88
7.50
7.48
7.46
7.07
7.06
7.00
6.99
6.98
6.97
4.07
4.03
2.47
O
O
OMe
OH
OMe
Defucogilvocarcin M (45)
^{1}H-NMR, 400 MHz
$CDCl_3$
0.94
0.96
0.95
0.93
0.99
0.95
0.94
3.02
2.88
2.87
ppm

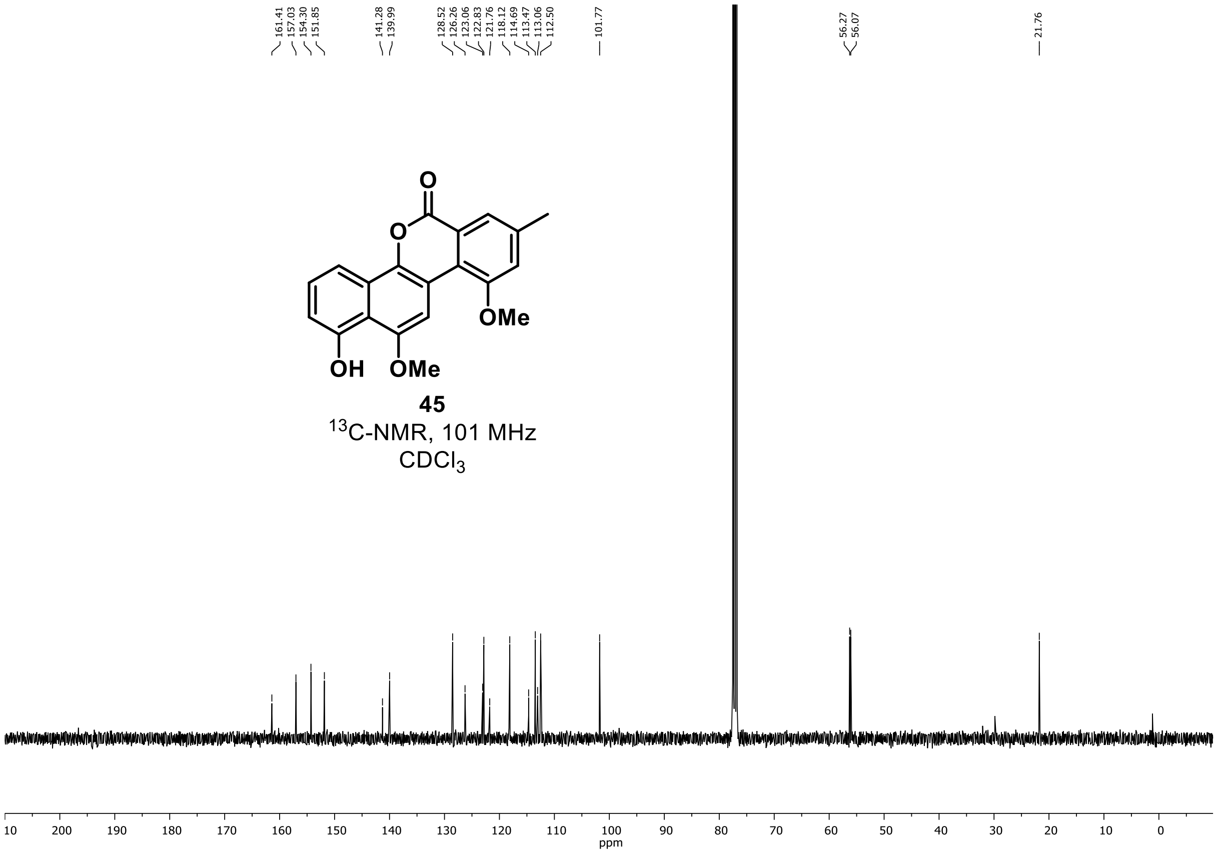
OH
OMe
OMe
45
^{13}C-NMR, 101 MHz
$CDCl_3$
161.41
157.03
154.30
151.85
141.28
139.99
128.52
126.26
123.06
122.83
121.76
118.12
114.69
113.47
113.06
112.50
101.77
56.27
56.07
21.76
ppm

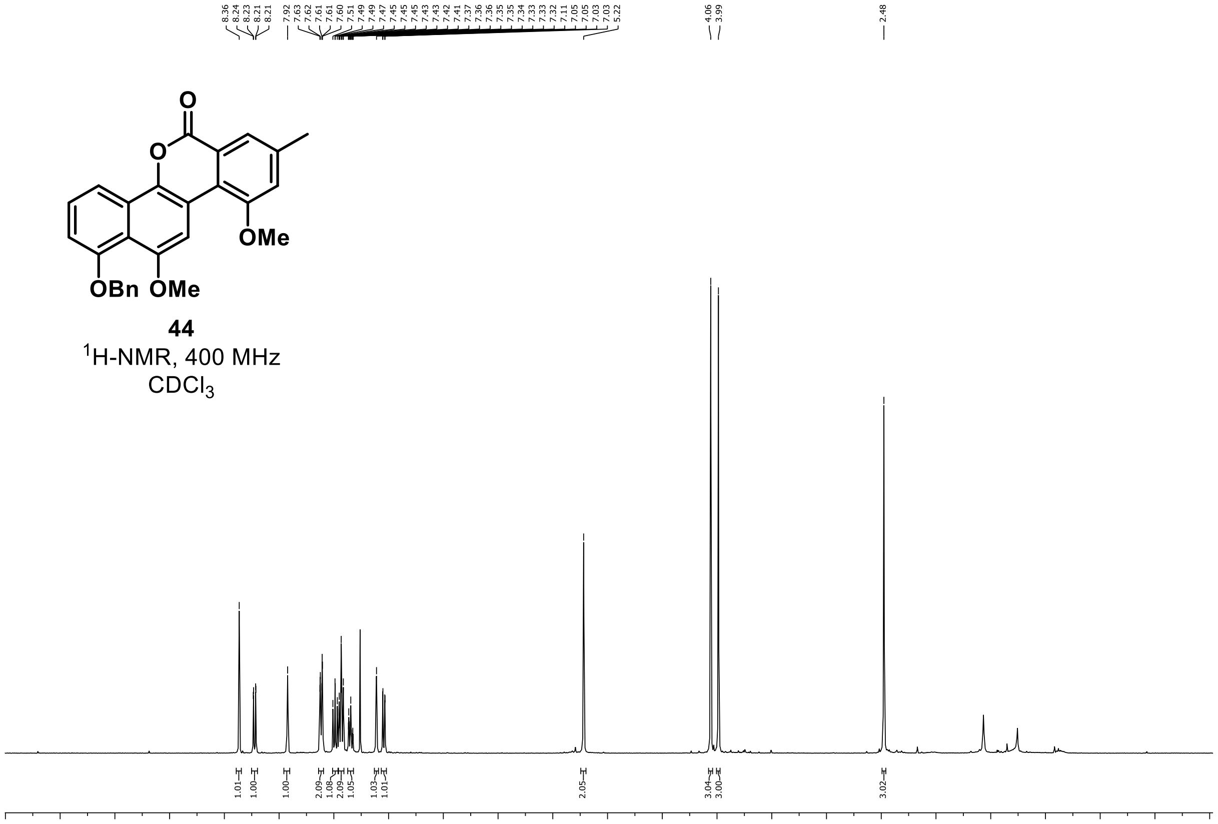
OBn
OMe
OMe
44
^{1}H-NMR, 400 MHz
$CDCl_3$
ppm

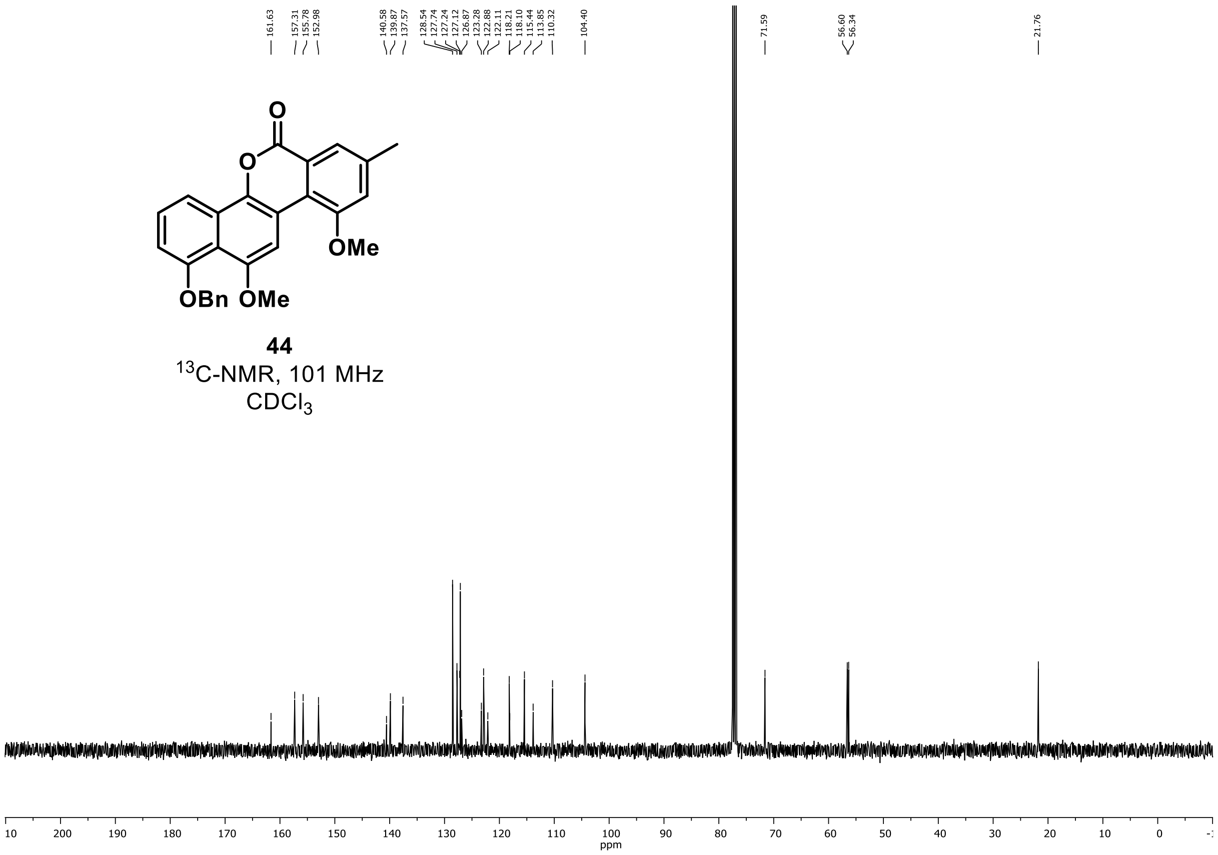
OBn OMe
OMe
44
13C-NMR, 101 MHz
CDCl3
ppm

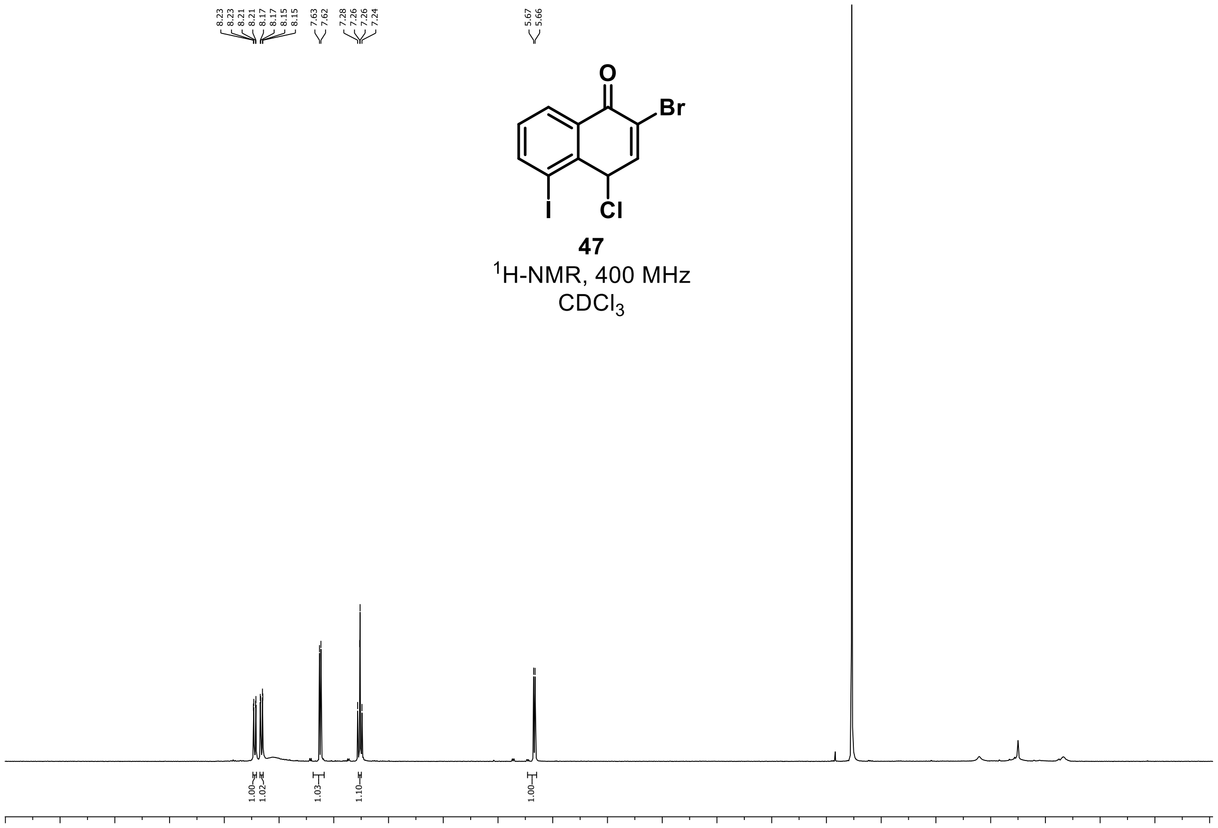
Br
I
Cl
47
1H-NMR, 400 MHz
CDCl3
ppm

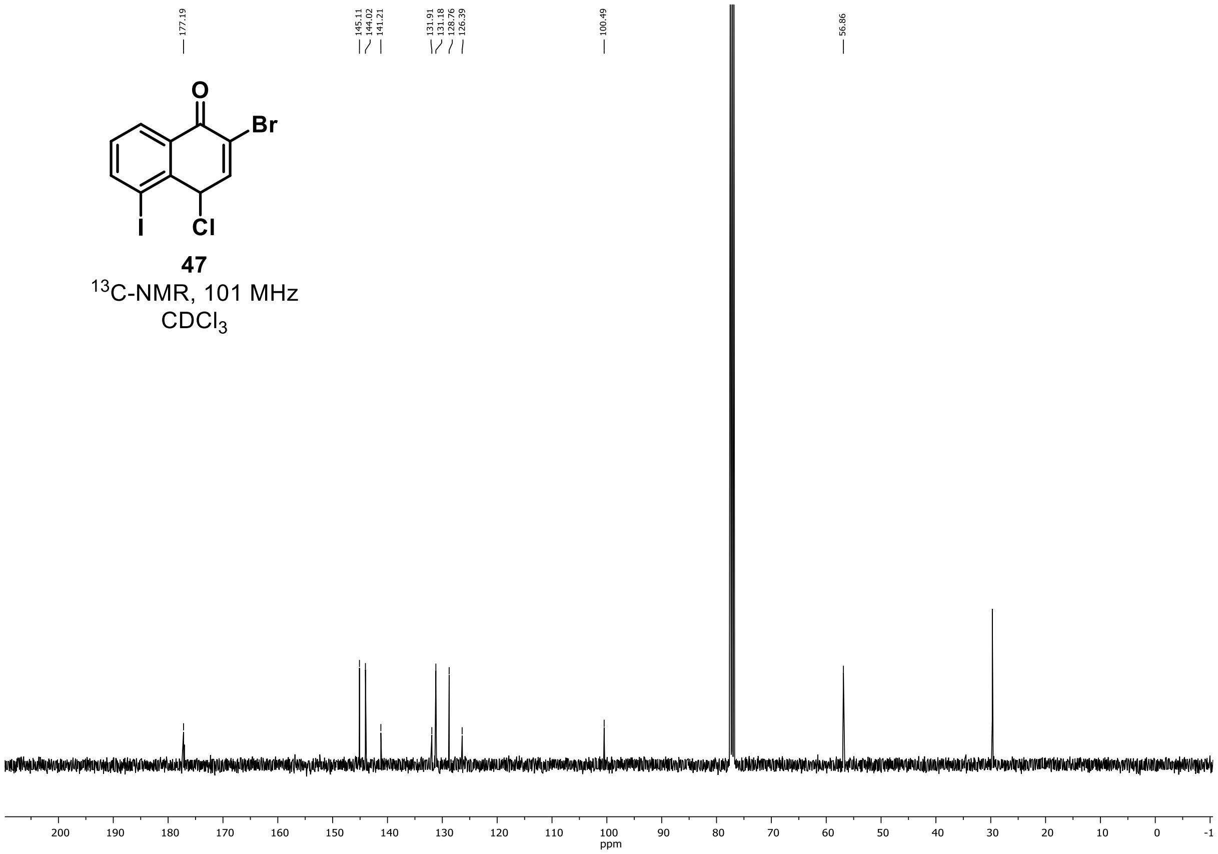
177.19
145.11
144.02
141.21
131.91
131.18
128.76
126.39
100.49
56.86
O
Br
I
Cl
47
^{13}C-NMR, 101 MHz
$CDCl_3$
ppm

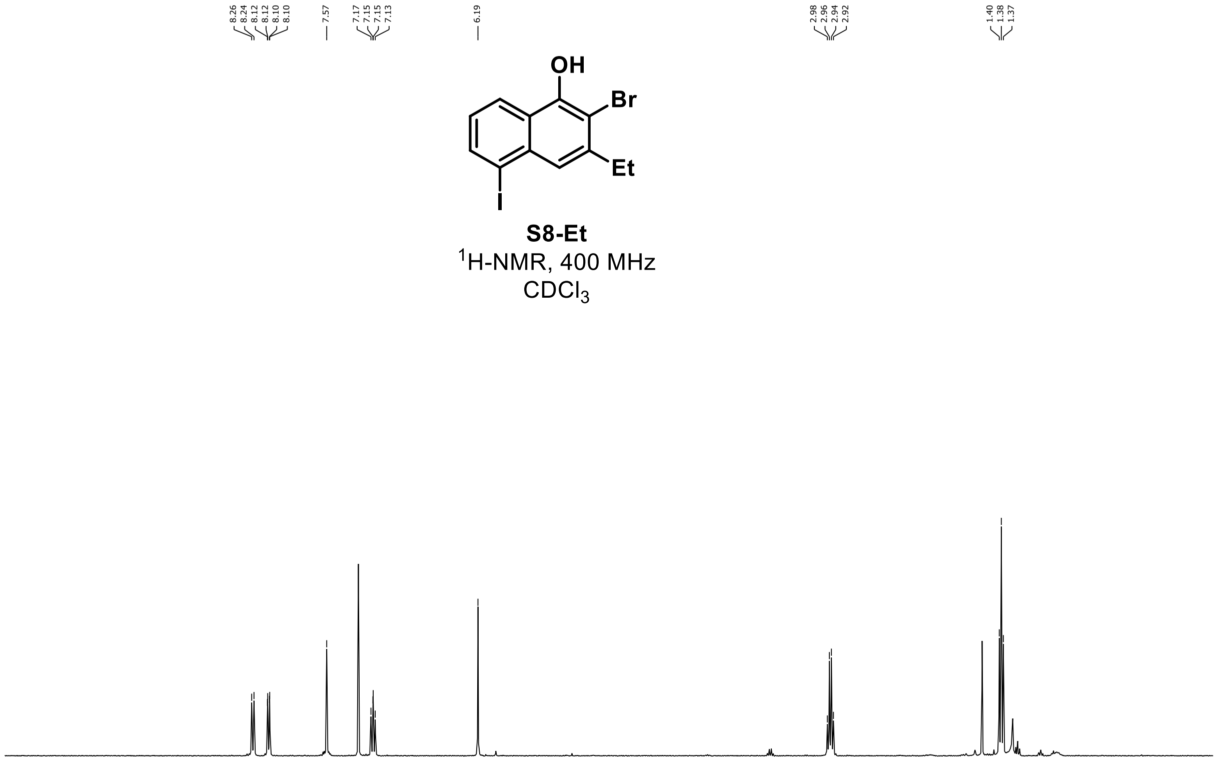
8.26
8.24
8.12
8.12
8.10
8.10
7.57
7.17
7.15
7.15
7.13
6.19
2.98
2.96
2.94
2.92
1.40
1.38
1.37
OH
Br
Et
I
S8-Et
^{1}H-NMR, 400 MHz
$CDCl_3$
1.02
1.01
1.04
1.05
1.00
2.09
3.06
ppm

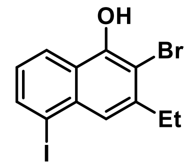

S8-Et
^{13}C-NMR, 101 MHz
$CDCl_3$

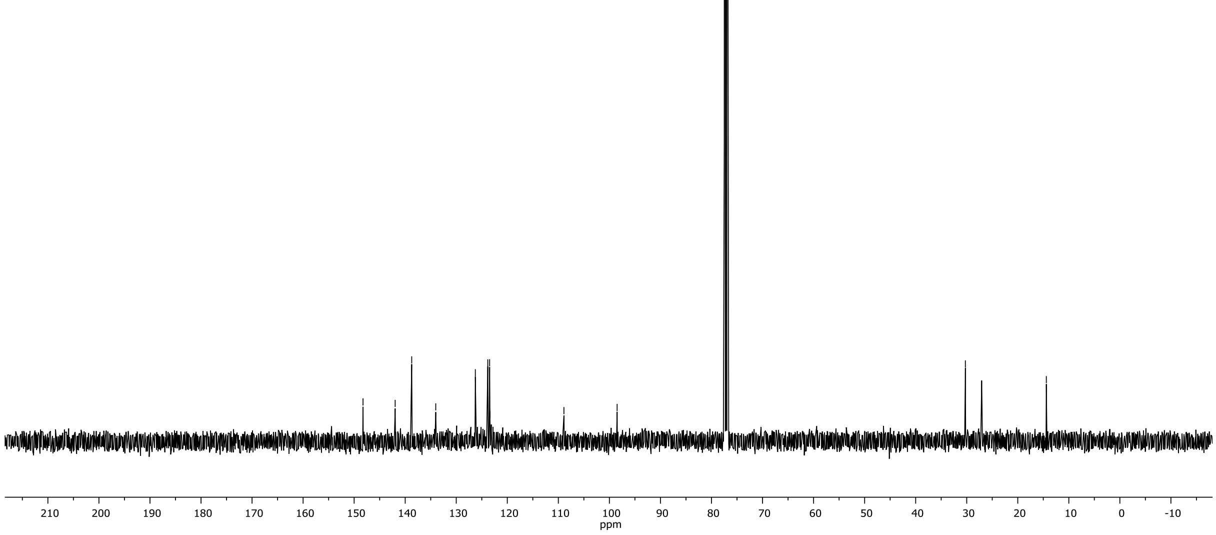

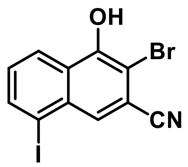

S8-CN
^{1}H-NMR, 400 MHz
CD_2Cl_2

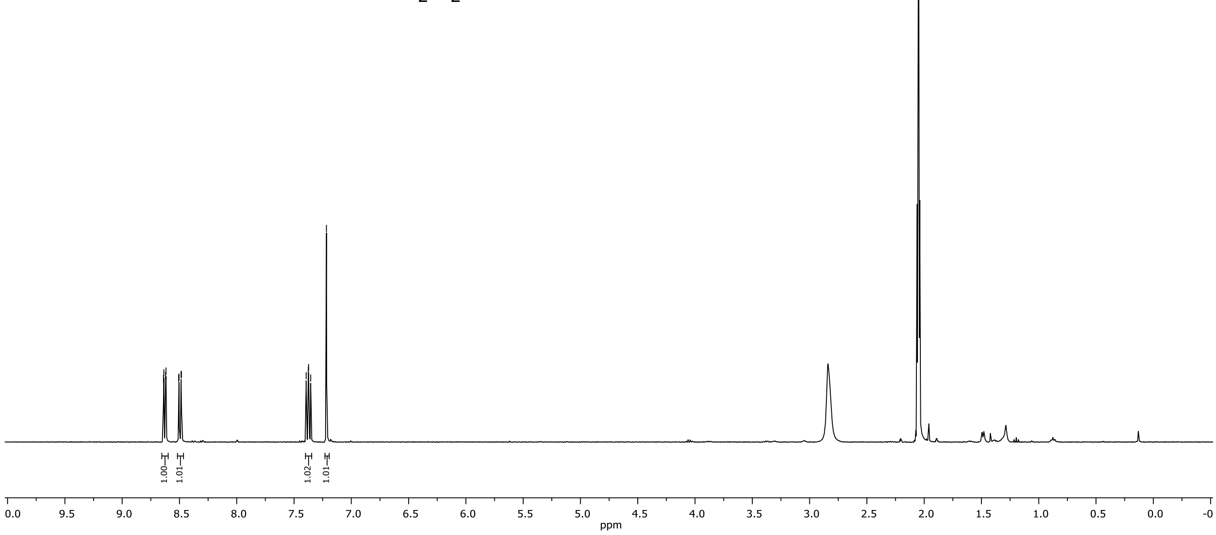

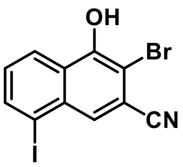

S8-CN
^{13}C-NMR, 101 MHz
CD_2Cl_2

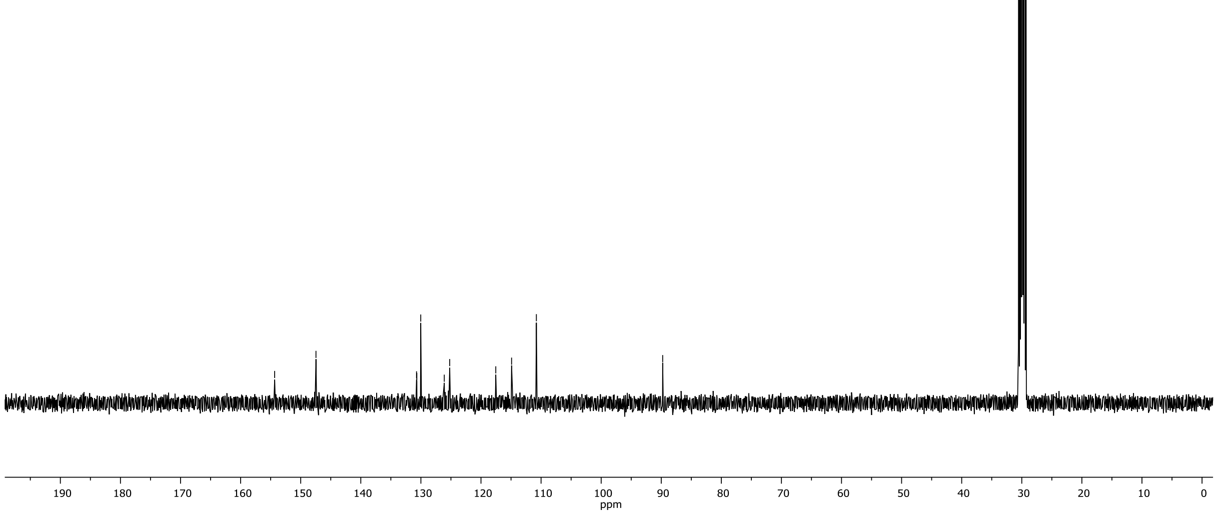

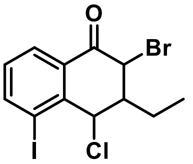

S9-Et
^{1}H-NMR, 400 MHz
$CDCl_3$

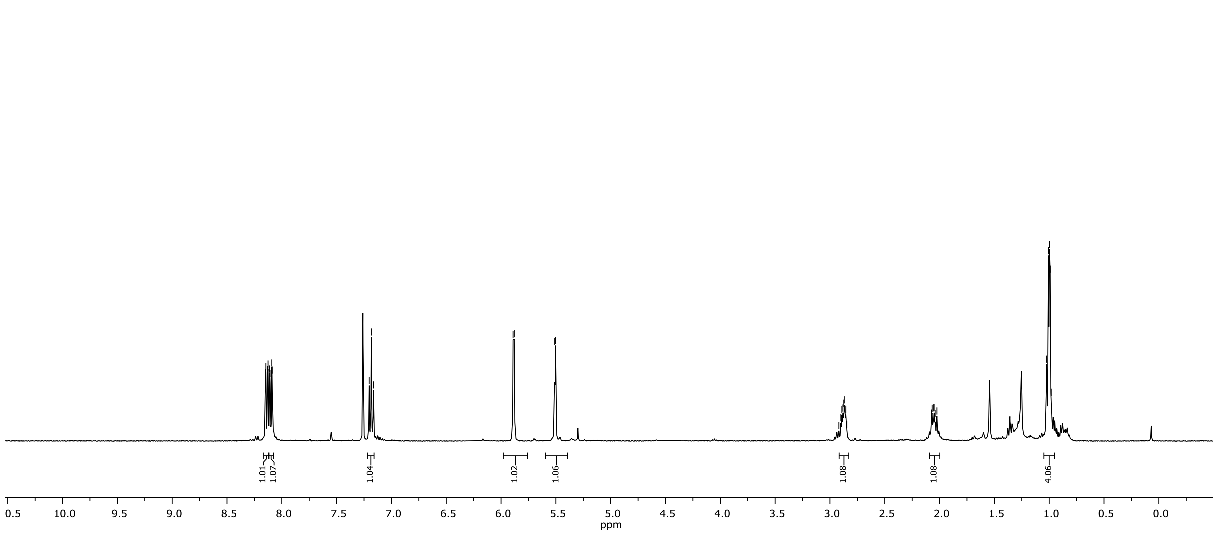

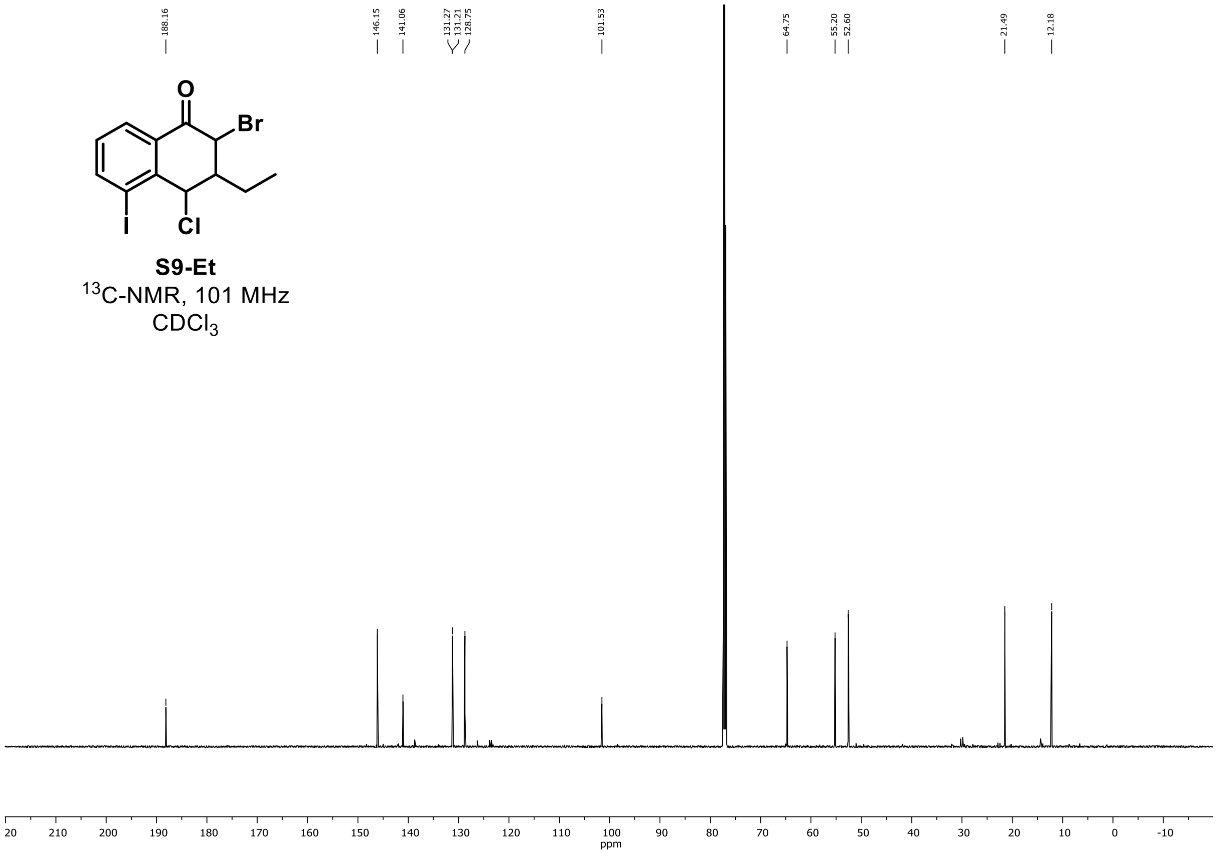
188.16
146.15
141.06
131.27
131.21
128.75
101.53
64.75
55.20
52.60
21.49
12.18
S9-Et
^{13}C-NMR, 101 MHz
$CDCl_3$
ppm

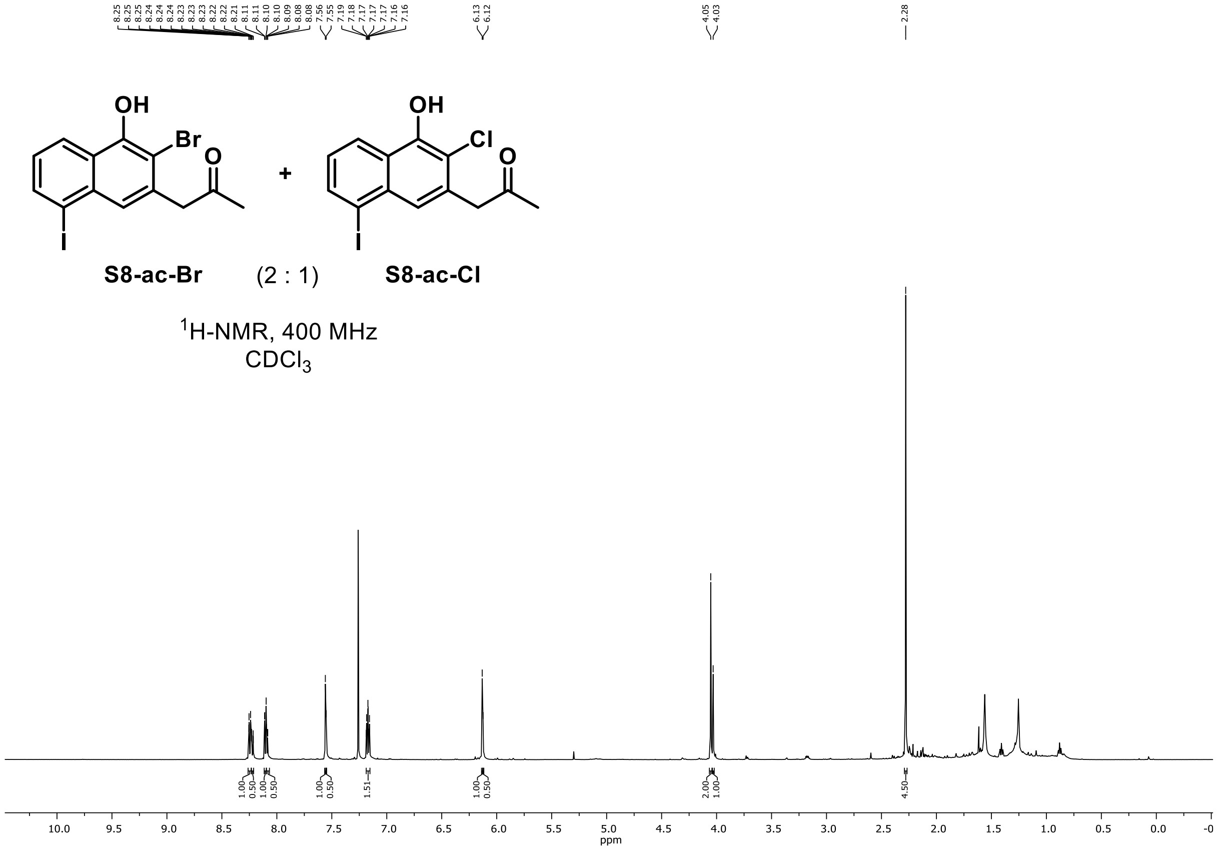
8.25 8.25 8.24 8.24 8.24 8.23 8.23 8.23 8.22 8.22 8.21 8.21 8.11 8.11 8.10 8.10 8.09 8.09 8.08 8.08 7.56 7.55 7.19 7.18 7.17 7.17 7.17 7.17 7.16 7.16
6.13 6.12
4.05 4.03
2.28
S8-ac-Br
(2 : 1)
S8-ac-Cl
^{1}H-NMR, 400 MHz
$CDCl_3$
ppm

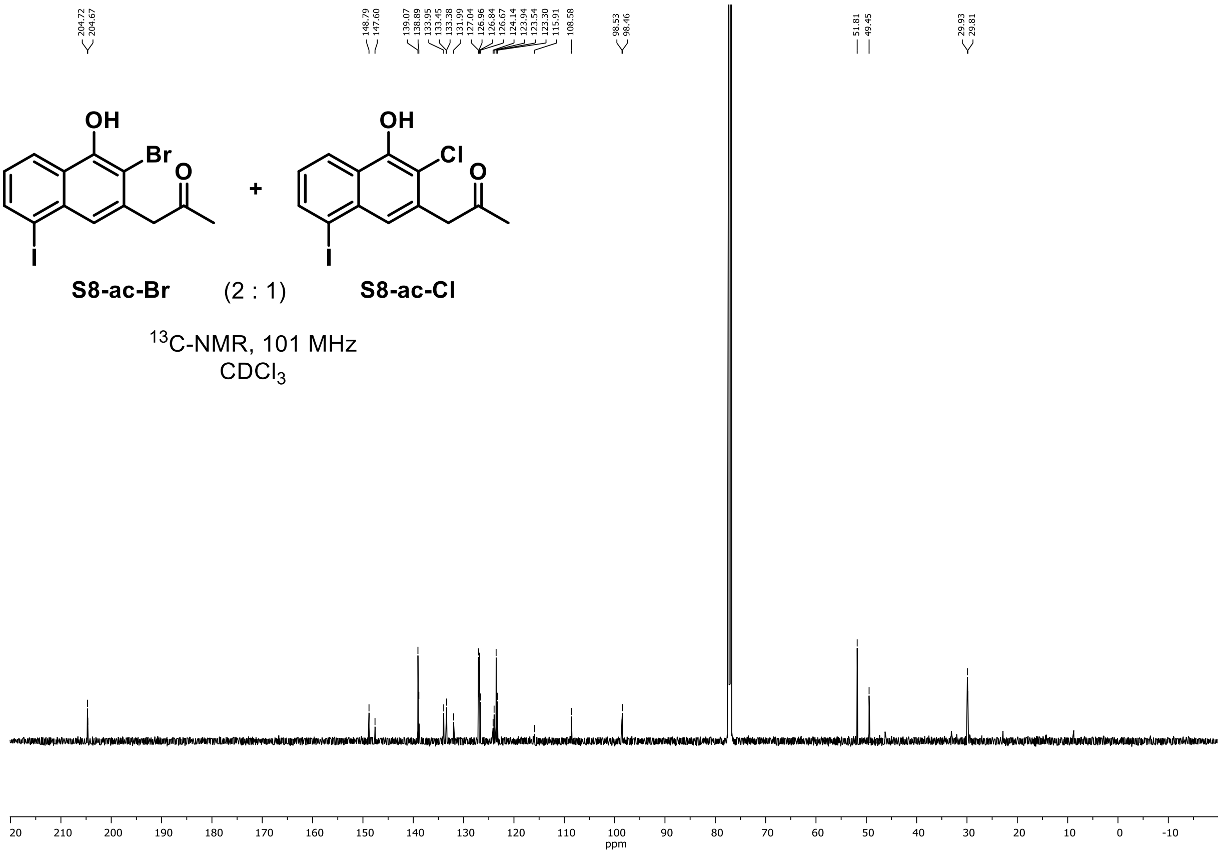
204.72
204.67
148.79
147.60
139.07
138.89
133.95
133.45
133.38
131.99
127.04
126.96
126.84
126.67
124.14
123.94
123.54
123.30
115.91
108.58
98.53
98.46
51.81
49.45
29.93
29.81
OH
Br
O
Cl
I
+
S8-ac-Br
(2 : 1)
S8-ac-Cl
13C-NMR, 101 MHz
CDCl3
ppm

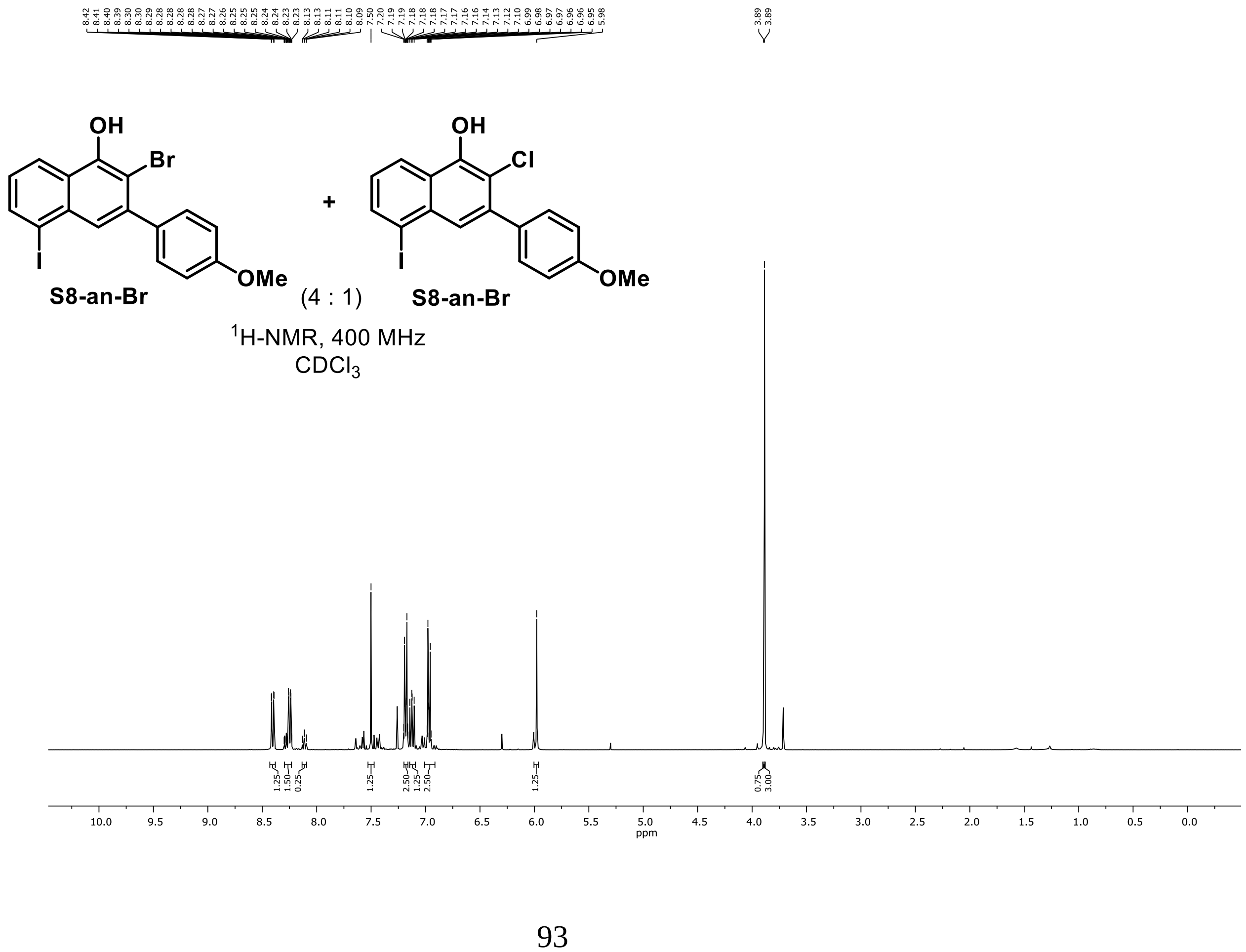
3.89
3.89
OH
Br
Cl
I
OMe
+
S8-an-Br
(4 : 1)
S8-an-Br
1H-NMR, 400 MHz
CDCl3
ppm

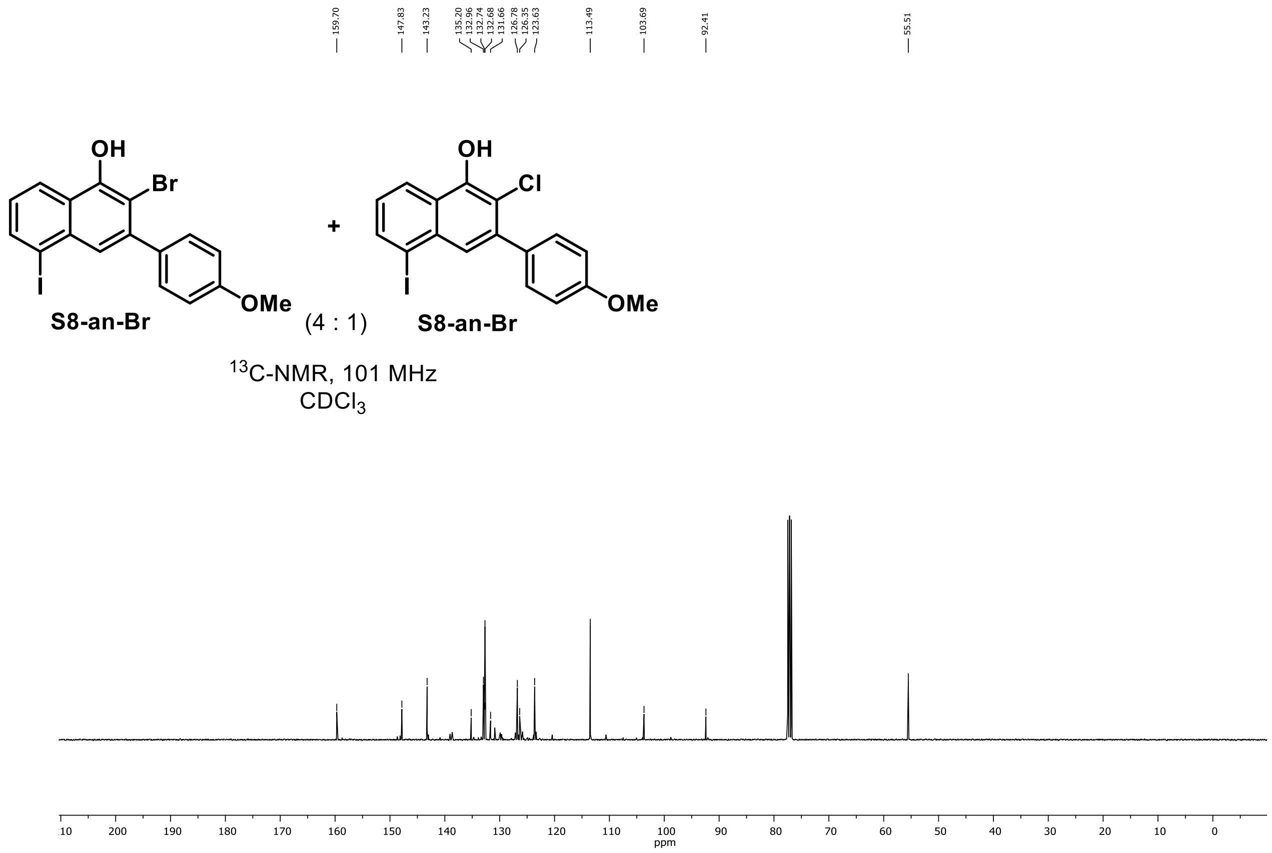

6. References

[1] W. Clark Still, Michael Kahn, and Abhijit Mitra, *J. Org. Chem.* **1978**, *43*, 2923–2925.

[2] Kofron, W. G.; Baclawski, L. M., *J. Org. Chem.* **1976**, *41*, 1879–1880.

[3] Lai, Yao-Hsuan; Mondal, Soumik; Su, Hsin-Tzu; Huang, Sheng-Cih; Wu, Mine-Hsine; Huang; Yang Lauderdale, Tsai-Ling; Song, Jen-Shin; Shia, Kak-Shan; Mong, Kwok-Kong Tony, *RSC Advances*, **2021**, *11*, 9426–9432.

[4] a) Akhmetova, N. E.; Shteingarts, V. D.; Yakobson, G. G, Izvestiya Akademii Nauk SSSR, Seriya Khimicheskaya, **1970**, 705 – 707. b) M. Funatsu, N. M. Green, and B. Witkop, *J. Am. Chem. Soc.* **1964**, 86, 1846–1848.
